# Nicotinamide modulates gut microbial metabolic potential and accelerates recovery in mild-to-moderate COVID-19

**DOI:** 10.1038/s42255-025-01290-1

**Published:** 2025-05-12

**Authors:** Stefan Schreiber, Georg H. Waetzig, Víctor A. López-Agudelo, Corinna Geisler, Kristina Schlicht, Sina Franzenburg, Romina di Giuseppe, Daniel Pape, Thomas Bahmer, Michael Krawczak, Elisabeth Kokott, Josef M. Penninger, Oliver Harzer, Jan Kramer, Tammo von Schrenck, Felix Sommer, Helena U. Zacharias, COVit-2 Study Group, Stefan Schreiber, Georg H. Waetzig, Víctor A. López-Agudelo, Corinna Geisler, Kristina Schlicht, Sina Franzenburg, Daniel Pape, Thomas Bahmer, Michael Krawczak, Elisabeth Kokott, Josef M. Penninger, Oliver Harzer, Jan Kramer, Felix Sommer, Helena U. Zacharias, Bernd Bokemeyer, Romina di Giuseppe, Wolfram Gronwald, Danielle M. M. Harris, Katharina Hartmann, Tim Hollstein, Peter J. Oefner, Sandra Plachta-Danielzik, Florian Tran, Tammo von Schrenck, Belén Millet Pascual-Leone, Sofia K. Forslund, Jan Heyckendorf, Konrad Aden, Regina Hollweck, Matthias Laudes, Philip Rosenstiel, Belén Millet Pascual-Leone, Sofia K. Forslund, Jan Heyckendorf, Konrad Aden, Regina Hollweck, Matthias Laudes, Philip Rosenstiel

**Affiliations:** 1 Department of Internal Medicine I, University Hospital Schleswig-Holstein, Kiel, Germany; 2 Institute of Clinical Molecular Biology, Kiel University and University Hospital Schleswig-Holstein, Kiel, Germany; 3 CONARIS Research Institute AG, Kiel, Germany; 4 Institute of Diabetes and Clinical Metabolic Research, Kiel University and University Hospital Schleswig-Holstein, Kiel, Germany; 5 Competence Network Intestinal Diseases, Kiel, Germany; 6 Institute of Medical Informatics and Statistics, Kiel University and University Hospital Schleswig-Holstein, Kiel, Germany; 7 Helmholtz Centre for Infection Research, Braunschweig, Germany; 8 Department of Medical Genetics, Life Science Institute, University of British Columbia, Vancouver, British Columbia Canada; 9 Eric Kandel Institute, Department of Laboratory Medicine, Medical University of Vienna, Vienna, Austria; 10 Bioscientia Healthcare GmbH, Ingelheim, Germany; 11 LADR Laboratory Group Dr. Kramer & Colleagues, Geesthacht, Germany; 12 Labor Dr. von Froreich, Hamburg, Germany; 13 Peter L. Reichertz Institute for Medical Informatics of TU Braunschweig and Hannover Medical School, Hannover Medical School, Hannover, Germany; 14 Department of Infectious Diseases, Respiratory Medicine and Critical Care, Charité-Universitätsmedizin Berlin, a corporate member of Freie Universität Berlin and Humboldt-Universität zu Berlin, Berlin, Germany; 15 Experimental and Clinical Research Center, Max Delbrück Center for Molecular Medicine and Charité-Universitätsmedizin Berlin, Berlin, Germany; 16 Charité-Universitätsmedizin Berlin, Freie Universität Berlin and Humboldt-Universität zu Berlin, Berlin, Germany; 17 Max Delbrück Center for Molecular Medicine in the Helmholtz Association (MDC), Berlin, Germany; 18 DZHK (German Centre for Cardiovascular Research), Berlin, Germany; 19 Novustat GmbH, Wollerau, Switzerland; 20 Institute of Functional Genomics, University of Regensburg, Regensburg, Germany; 21 Institute of Human Nutrition and Food Science, Kiel University, Kiel, Germany

**Keywords:** Translational research, Viral infection, Molecular medicine, Metabolism

## Abstract

Cellular NAD^+^ depletion, altered tryptophan metabolism and gut microbiome dysbiosis are associated with disease progression and unfavourable clinical outcomes in COVID-19. Here, we show that supplementing tryptophan metabolism with nicotinamide alleviates COVID-19 symptoms. We evaluate a 4-week intervention with a novel nicotinamide formulation (1,000 mg) in a prospective, double-blind, randomized, placebo-controlled trial in 900 symptomatic outpatients with PCR-proven COVID-19. In the primary analysis population of participants at risk for severe COVID-19, 57.6% of those receiving nicotinamide and 42.6% receiving placebo recover from their performance drop at week 2 (*P* = 0.004). Nicotinamide is also beneficial for returning to normal activities (*P* = 0.009). Effects on gut metagenomic signatures parallel clinical efficacy, suggesting that nicotinamide influences COVID-19-associated faecal microbiome changes. After 6 months, responders to nicotinamide in acute COVID-19 show fewer post-COVID symptoms than placebo responders (*P* = 0.010). No relevant safety signals are observed. Overall, our results show that nicotinamide leads to faster recovery of physical performance and modulates COVID-19-associated faecal microbiome changes.

## Abstract (web summary)

In a prospective, double-blind, randomized, placebo-controlled trial, supplementation with a combination of conventional and gut-targeted nicotinamide was safe and effective in ameliorating COVID-19 symptoms.

## Main

COVID-19 remains a large global disease burden, causing a substantial loss in work productivity even in the post-pandemic phase. Respiratory symptoms are often linked to a sharp drop in physical performance and the inability to perform normal activities. Despite a strong reduction in overall mortality due to vaccination and antiviral treatments, there is a large unmet need for an effective, broad, symptomatic intervention.

Nicotinamide is required to generate oxidized nicotinamide adenine dinucleotide (NAD^+^), a coenzyme central to cellular energy metabolism. However, NAD^+^ availability is diminished in viral infections, particularly in COVID-19 (refs. ^1,2^). NAD^+^ can be synthesized from the essential amino acid tryptophan through a de novo pathway in which the nicotinamide base is newly generated. Nicotinic acid, nicotinamide riboside and nicotinamide also serve as NAD^+^ precursors in enzymatic salvage pathways for NAD^+^ regeneration. Cells continuously synthesize NAD^+^ because it functions both as a recyclable coenzyme and as a substrate for NAD^+^-consuming enzymes, for example sirtuins^3^.

Notably, elevated tryptophan catabolism, indicated by high levels of kynurenine, an essential intermediate in the de novo NAD^+^ synthesis pathway, characterizes acute inflammation during SARS-CoV-2 infection^4–6^. The degradation of tryptophan results from increased activity of tryptophan-catabolizing enzymes, such as indoleamine 2,3-dioxygenase-1, and not only is associated with COVID-19 severity^4–6^, but also has been observed in other infectious diseases, including community-acquired bacterial pneumonia^7^ and viral infections^8,9^. Additionally, tryptophan absorption depends on the presence of angiotensin-converting enzyme-2 on the intestinal epithelium, which is also the entry point for SARS-CoV-2 (refs. ^5,10^).

COVID-19 is closely linked to disruptions of the gut microbiome, characterized by reduced microbial diversity and a decline in beneficial bacterial species^11–14^. These imbalances are associated with increased inflammation and immune dysregulation, and are assumed to contribute to more severe disease outcomes, for example by licensing immune responses through microbe-derived metabolites^15–17^.

We have previously shown that tryptophan supports gut microbiome homeostasis^18^ and that nicotinamide supplementation exerts strong, microbiota-dependent anti-inflammatory effects in a colitis model^18,19^. In mice, gut-targeted nicotinamide showed a dose-dependent anti-inflammatory effect, surpassing the benefits of systemic supplementation^19^. Given that impaired tryptophan cometabolism is associated with gut microbial dysbiosis in people with COVID-19 (ref. ^17^), topical nicotinamide might modulate the gut microbiome and improve outcomes of SARS-CoV-2 infections, complementing its systemic antiviral benefits^1,20^.

Hence, we developed a pharmaceutical pH-dependent matrix tablet formulation with ingredients approved for use in both food and pharmaceuticals (DRKS00023384, NCT05258474). This formulation is designed to release nicotinamide in the lower small intestine and colon, ensuring systemic supply of nicotinamide and targeting more distal parts of the intestinal tract, including the microbiota.

This study reports the results of two randomized controlled trials, a smaller pilot experiment (COVit-1; DRKS00021214) using conventional nicotinamide tablets, and the larger COVit-2 trial, which combined conventional and gut-targeted nicotinamide tablets in outpatients within 7 days of testing PCR-positive for SARS-CoV-2.

## Results

**Extended Data Fig. 1:**
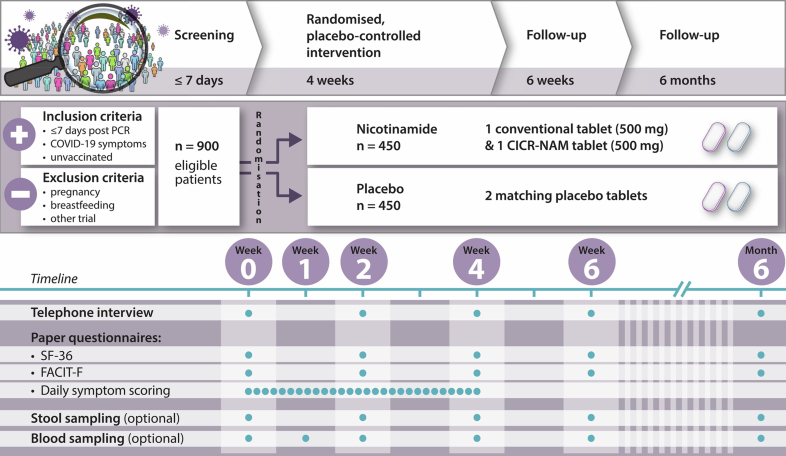
Design of the COVit-2 trial. Details on trial procedures and design are available in Supplementary Section 3.1.

The 4-week intervention in the pilot trial COVit-1 indicated faster restoration of physical performance (18 of 23 participants receiving nicotinamide versus 12 of 23 in the control group at week 2) and time to complete resolution of symptoms (Supplementary Section 1). The results provided the impetus for the COVit-2 trial, described in the following sections (details on trial procedures and design are available in Extended Data Fig. 1 and Supplementary Section 3.1).

**Extended Data Fig. 2:**
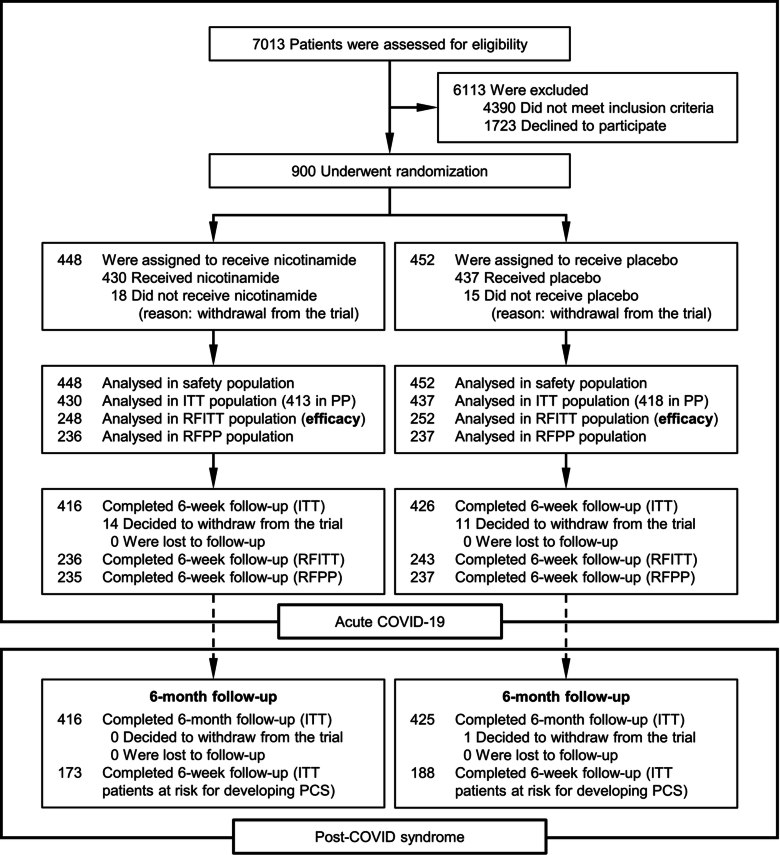
CONSORT diagram for COVit-2. ITT, intention-to-treat; PP, per-protocol; RFITT, participants with at least one risk factor for severe COVID-19; RFPP, participants from RFITT with per-protocol compliance. The main reasons for exclusion of screened subjects were exceedance of the 7-day time window after the first positive PCR test, complete lack of symptoms, rejection of the trial by the subject, or vaccination against SARS-CoV-2. Refusal of potential subjects to participate occurred during the multi-step registration and verification process, usually after they had fully understood the comprehensive requirements of the trial. Reasons for withdrawal from the trial were mostly non-compliance and worsening of COVID-19.

Screening of 7,013 individuals for COVit-2 resulted in randomization of 900 participants (safety population: 448 assigned to nicotinamide, 452 to placebo). Of these, 867 received the investigational product, and 500 (248 receiving nicotinamide, 252 placebo) qualified for the risk factor intention-to-treat (RFITT) population for primary analysis (Extended Data Fig. 2).

The analysis populations in COVit-2 were similar with respect to demographic and clinical characteristics at baseline (intention-to-treat (ITT) population, *n* = 867: Table 1; RFITT population, *n* = 500: Supplementary Table 3). A total of 97.1% of the participants in the ITT population (*n* = 842) and 95.8% of those in the RFITT population (*n* = 479) completed the 4-week intervention period and the follow-up at week 6. Only one participant left the trial between week 6 and the follow-up after 6 months. The trial had a low drop-out rate (4.2% of the 500 participants analysed in the RFITT population, with 479 completing the 6-week follow-up), and 98.5% of participants (472 of 479) finished the full 6-week trial, adhering to the protocol. Although efficacy analyses for acute COVID-19 had been planned to include only the RFITT population, safety data were obtained from the entire cohort (*n* = 900). At the 6-month follow-up, subgroups of participants at risk for developing post-COVID syndrome (PCS) and responders to the intervention were analysed in addition to the primary ITT population (Supplementary Section 3.4).

**Table 1:** Demographic and clinical characteristics of the participants at baseline (ITT population)

	Nicotinamide (*n* = 430)	Placebo (*n* = 437)	Total (*n* = 867)
Median age (range) at randomization in years	37 (18–75)	38 (18–70)	37 (18–75)
Sex, no. of participants (%)			
Male	166 (38.6)	179 (41.0)	345 (39.8)
Female	264 (61.4)	258 (59.0)	522 (60.2)
Race or ethnic group, no. of participants (%)			
White	411 (95.6)	413 (94.5)	824 (95.0)
Other	18 (4.2)	24 (5.5)	42 (4.8)
Not reported	1 (0.2)	0 (0.0)	1 (0.1)
Body mass index (BMI) (mean ± s.d.)	24.9 ± 4.8	25.7 ± 4.8	25.3 ± 4.8
Risk factors for severe COVID-19, no. (%)			
Age ≥ 60 years	22 (5.1)	20 (4.6)	42 (4.8)
BMI ≥ 30 and/or type 2 diabetes	58 (13.5)	73 (16.7)	131 (15.1)
Cardiovascular diseases, high blood pressure or stroke	63 (14.7)	60 (13.7)	123 (14.2)
Asthma, chronic obstructive pulmonary disease or other chronic lung disease	41 (9.5)	52 (11.9)	93 (10.7)
Current or former smoker	165 (38.4)	171 (39.1)	336 (38.8)
Other risk factors	21 (4.9)	15 (3.4)	36 (4.2)
At least one risk factor (RFITT population)	248 (57.7)	252 (57.7)	500 (57.7)

### Efficacy

**Fig. 1:**
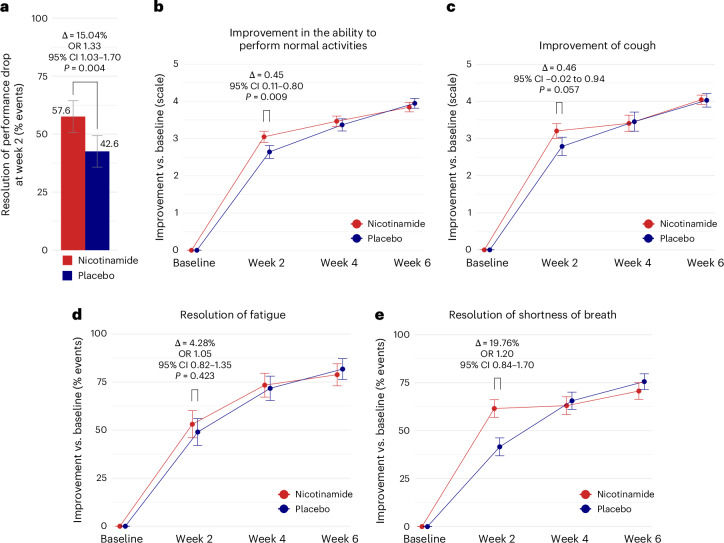
Clinical endpoints. **a**, The primary endpoint (RFITT population) was a significant difference in resolution of performance drop at week 2 in the 379 participants reporting the symptom at baseline (nicotinamide: *n* = 191 (73 males, 118 females); placebo: *n* = 188 (77 males, 111 females)). One hundred ten participants responded to nicotinamide at week 2 (48 males, 62 females) and 80 to placebo (34 males, 46 females). Data are shown as relative frequency ± s.d. Two-sided Fisher’s exact test, adjusted for hierarchical testing. OR, odds ratio; CI, confidence interval. **b**–**e**, Secondary endpoints (RFITT population). Data are shown as mean ± s.e. (**b**,**c**) or relative frequency ± s.d. (**d**,**e**). **b**, Significant improvement in the ability to perform normal activities at week 2 in the 198 participants with baseline scores of >3 (nicotinamide: *n* = 103 (41 males, 62 females); placebo: *n* = 95 (34 males, 61 females)): 3.07 ± 0.12 with nicotinamide (males: 3.34 ± 0.17, females: 2.89 ± 0.16), 2.62 ± 0.13 with placebo (males: 3.00 ± 0.19, females: 2.41 ± 0.16). Two-sided *t*-test of contrast within a mixed model for repeated measures (MMRM), adjusted for hierarchical testing. **c**, Improvement of cough at week 2 in the 77 participants with baseline scores of >3 (nicotinamide: *n* = 44 (17 males, 27 females); placebo: *n* = 33 (8 males, 25 females)): 3.22 ± 0.16 with nicotinamide (males: 3.31 ± 0.21, females: 3.17 ± 0.22), 2.76 ± 0.18 with placebo (males: 3.09 ± 0.31, females: 2.66 ± 0.23). Two-sided *t*-test of contrast within MMRM, adjusted for hierarchical testing. **d**, Among the 397 participants reporting fatigue at baseline (nicotinamide: *n* = 199 (82 males, 117 females); placebo: *n* = 198 (78 males, 120 females)), 105 responded to nicotinamide at week 2 (48 males, 57 females) and 96 to placebo (45 males, 51 females). Two-sided Fisher’s exact test, adjusted for hierarchical testing. **e**, Among the 182 participants reporting shortness of breath at baseline (nicotinamide: *n* = 92 (36 males, 56 females); placebo: *n* = 90 (28 males, 62 females)), 56 responded to nicotinamide at week 2 (24 males, 32 females) and 37 to placebo (14 males, 23 females). Exploratory *P* value from two-sided, post-hoc, unadjusted Fisher’s exact test: *P* = 0.012. Details regarding symptoms and risk factors are available in Supplementary Tables 8 and 9 and Supplementary Sections 3.2 and 3.3. Source data

**Extended Data Fig. 3:**
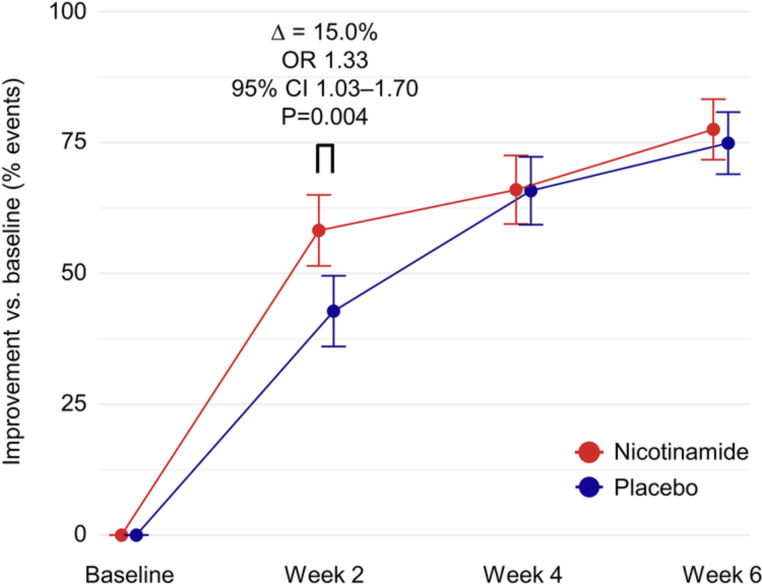
Primary endpoint: resolution of performance drop in the RFITT population from baseline to week 6. A significantly different resolution of performance drop was seen in the 379 participants reporting the symptom at baseline (nicotinamide: *n* = 191 [73 males, 118 females]; placebo: *n* = 188 [77 males, 111 females]). One hundred ten participants responded to nicotinamide at week 2 (48 males, 62 females) and 80 participants to placebo (34 males, 46 females). Graphs represent relative frequency ± s.d. Two-sided Fisher exact test, adjusted for hierarchical testing. Source data

By week 2, 110 of 191 participants with reduced physical performance at baseline (57.6%) receiving nicotinamide and 80 of 188 participants receiving placebo (42.6%) had recovered from their decline in physical performance (absolute difference, 15.04 percentage points; odds ratio, 1.33; 95% confidence interval, 1.03 to 1.70; *P* = 0.004) (Fig. 1a and Extended Data Fig. 3). The number needed to treat was seven.

**Extended Data Fig. 4:**
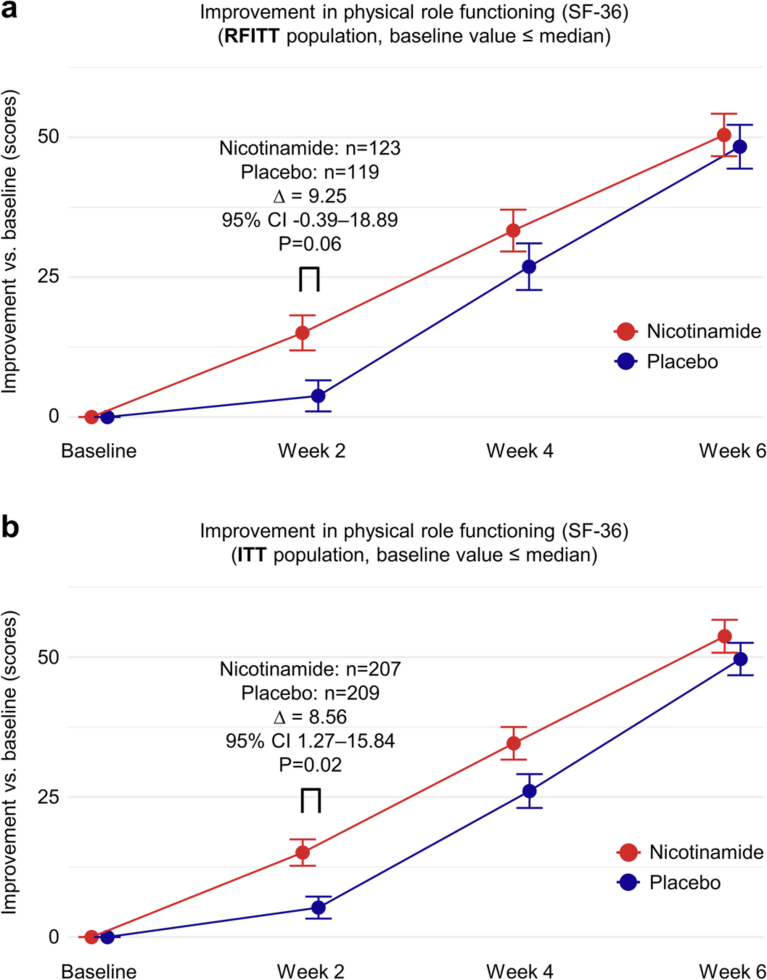
Physical role functioning (SF-36 questionnaire) from baseline to week 6. **a**, Primary analysis population RFITT. **b**, ITT population. For details on trial populations, see Supplementary Section 3.4. Only subjects with severe complaints (baseline values ≤ median) were included in the analyses (RFITT: nicotinamide: *n* = 123 [49 males, 74 females], placebo: *n* = 119 [45 males, 74 females]; ITT: nicotinamide: *n* = 207 [79 males, 128 females], placebo: *n* = 209 [74 males, 135 females]. Graphs represent mean ± s.e. Two-sided t-test of contrasts within a mixed model for repeated measures, adjusted for multiple timepoints. The questions from SF-36 V 1.0 related to physical role functioning were as follows: ‘During the past 4 weeks, have you had any of the following problems with your work or other regular daily activities as a result of your physical health? Cut down the amount of time you spent on work or other activities; accomplished less than you would like; were limited in the kind of work or other activities; had difficulty performing the work or other activities (for example, it took extra effort)’. Source data

**Extended Data Fig. 5:**
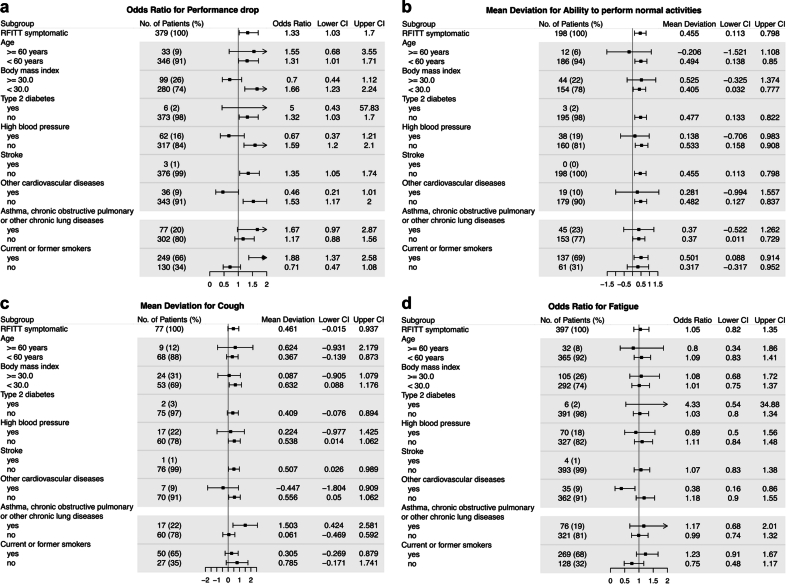
Subgroup analysis of primary and key secondary endpoints in the RFITT population at week 2. **a**, Resolution of performance drop (primary endpoint). The number of participants indicates those included in the analysis due to the presence of performance drop at baseline. **b**, Improvement in the ability to perform normal activities (first key secondary endpoint). The number of participants indicates those included in the analysis due to the presence of a sufficiently severe reduction in the ability to perform normal activities (a value of >3 on the 6-point complaint scale) at baseline. **c**, Improvement in cough (second key secondary endpoint). The number of participants indicates those included in the analysis due to the presence of a sufficiently severe cough (a value of >3 on the 6-point complaint scale) at baseline. **d**, Resolution of fatigue (third key secondary endpoint). The number of participants indicates those included in the analysis due to the presence of fatigue at baseline. For details regarding symptoms and risk factors, see Supplementary Sections 3.2–3.4. Forest plot graphs represent odds ratios (**a**, **d**) or mean deviations (**b**, **c**) and the respective lower/upper 95% confidence intervals (CI). Source data

**Extended Data Fig. 6:**
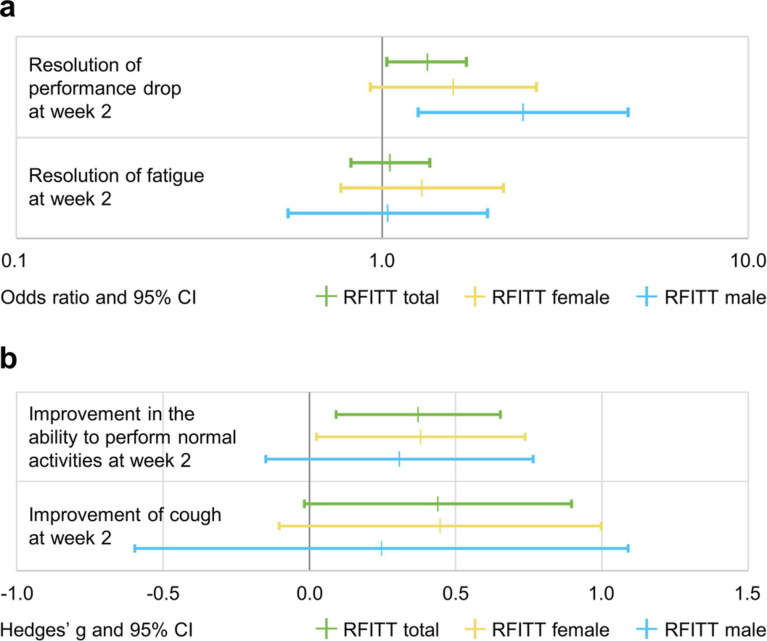
Sex-specific analysis of primary and key secondary endpoints in the RFITT population at week 2. **a**, Binary symptoms. Forest plot graphs represent odds ratios and the respective lower/upper 95% confidence intervals (CI). Resolution of performance drop: nicotinamide: *n* = 191 (73 males, 118 females); placebo: *n* = 188 (77 males, 111 females). Resolution of fatigue: nicotinamide: *n* = 199 (82 males, 117 females); placebo: *n* = 198 (78 males, 120 females). The number of participants indicates those included in the analysis due to the presence of performance drop or fatigue at baseline, respectively. **b**, Complaint scale. Forest plot graphs represent Hedges’ g and lower/upper CI. Improvement in the ability to perform normal activities: nicotinamide: *n* = 103 (41 males, 62 females); placebo: *n* = 95 (34 males, 61 females). Improvement of cough: nicotinamide: *n* = 44 (17 males, 27 females); placebo: *n* = 33 (8 males, 25 females). The number of participants indicates those included in the analysis due to the presence of a sufficiently severe reduction in the ability to perform normal activities or a sufficiently severe cough (a value of >3 on the 6-point complaint scale) at baseline, respectively. Source data

The trial also met the first of three prespecified key secondary endpoints. By week 2, participants taking nicotinamide had recovered their ability to perform normal activities significantly faster than those taking the placebo (absolute difference, 0.45 scale points; 95% confidence interval, 0.11 to 0.80; *P* = 0.009) (Fig. 1b). The difference in recovery from severe cough (absolute difference, 0.46 scale points; 95% confidence interval, −0.02 to 0.94; *P* = 0.057) (Fig. 1c) was of borderline statistical significance only in per-protocol participants (RFPP population; *P* = 0.049). For the third key secondary endpoint, the resolution of fatigue, the observed difference did not achieve statistical significance (Fig. 1d). Only a small number of participants in both groups reported severe fatigue at week 2 (as indicated by the descriptive statistics of the SF-36 and FACIT-F questionnaires; Supplementary Tables 4 and 5). Therefore, no additional endpoints were formally statistically tested, although trends suggested greater effectiveness of nicotinamide over placebo for shortness of breath (Fig. 1e) and the ‘physical role functioning’ subscale of the SF-36 questionnaire (Extended Data Fig. 4). Exploratory subgroup analyses of the primary and three key secondary endpoints suggested that individuals with a history of lung disease or smoking might specifically benefit from nicotinamide (Extended Data Fig. 5), but there were no sex-dependent differences (Extended Data Fig. 6 and Supplementary Tables 6–13). In exploratory sex-specific analyses of symptomatic males and females in the RFITT population, significant effects on recovery from performance drop (in males) and improved ability to perform normal activities (in females), both at week 2, were retained despite the reduced sample sizes (Supplementary Tables 8–13). Further data covering primary, secondary and exploratory endpoints are provided in the Supplementary Sections 2.2 and 2.3).

### Metabolic response of the gut microbiome

To assess gut microbiota shifts induced by the intervention, we analysed longitudinal faecal samples using 16S rDNA phylogenomics (*n* = 70; 280 samples) and metagenomics (*n* = 18; 72 samples) across four timepoints. Stool sampling was optional (details in the Supplementary Section 3.5). No significant differences in participant characteristics between intervention arms were observed (Supplementary Tables 16 and 17).

**Fig. 2:**
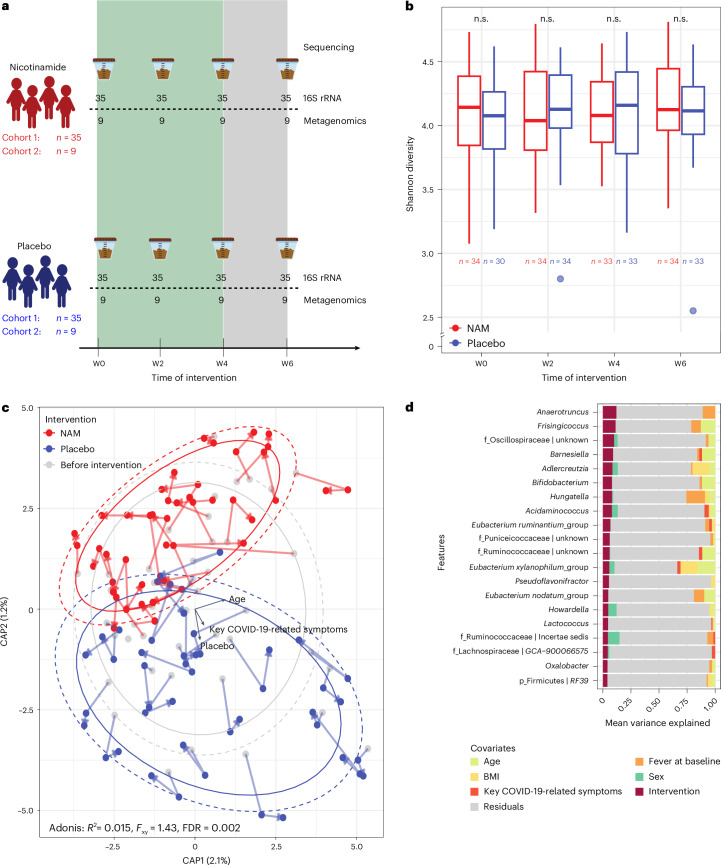
Gut microbiome characterization in COVit-2 trial participants. **a**, Stool samples from 88 participants were collected at baseline (week (W) 0), during intervention (weeks 2 and 4; nicotinamide (NAM) or placebo) and at follow-up (week 6). Cohort 1 included 35 participants per group (NAM: 25 females, 10 males; placebo: 24 females, 11 males), and cohort 2 included 9 participants per group (NAM: 4 females, 5 males; placebo: 5 females, 4 males). Samples underwent 16S rRNA (*n* = 280) and shotgun metagenomics (*n* = 72) sequencing. **b**, α-diversity analysis (Shannon index at amplicon sequence variant level) of 16S rRNA data showed no significant (n.s.) differences across intervention groups or timepoints (two-sided Wilcoxon rank-sum test; likelihood ratio test on linear mixed-effect models; *n* per group is depicted below each box plot). Box plots show the median (centre line), interquartile range (IQR, box), 1.5 × IQR (whiskers) and outliers (points). **c**, Microbiota shifts (Aitchison distance, 16S rRNA) were examined using constraint-based principal coordinates analysis in participants with key COVID-19-related symptoms. Significant differences emerged between nicotinamide and placebo at week 2 and week 4 (*n* = 45 per intervention; PERMANOVA, *R*^2^ = 0.015, *F*_*xy*_ = 1.43, false discovery rate (FDR) = 0.002) but not at baseline (week 0) (NAM: n = 24; placebo: n = 23; PERMANOVA, *R*^2^ = 0.018, *F*_*xy*_ = 0.82, FDR = 0.99; Supplementary Fig. 4). Dots indicate individual samples, and arrows represent trajectories (baseline → week 2 → week 4). Ellipses show sample distributions per intervention group (solid line: 70% confidence; dashed line: 80% confidence; assuming multivariate normality). Black arrows show the impact of key COVID-19-related symptoms, intervention and age on microbiota dissimilarity, proportional to their correlation. Placebo and key COVID-19-related symptoms had similar effects. FDR: Benjamini–Hochberg-corrected *P* values. **d**, Variance partition analysis of the top 20 microbial genera that show highest variation at week 2 and week 4 in the 16S data (*n* = 67 per intervention). The bar plot shows the mean variance explained for the top 20 microbial genera, with variance attributed to covariates including age (light green), body mass index (BMI) (yellow), key COVID-19-related symptoms (red), fever at baseline (orange), sex (dark green), intervention (purple) and residuals (grey). Prefixes in genus labels denote higher taxonomic ranks: f_, family; p_, phylum. Only samples with at least 5,000 reads were included. For additional metagenomics-based variance partition analyses at the taxonomical level, see Supplementary Fig. 7 and Supplementary Table 21. Source data

We first analysed compositional changes using phylogenomic 16S rRNA data (Fig. 2). Analysis of α-diversity (within-sample diversity) did not show significant longitudinal or cross-sectional differences (Fig. 2b and Supplementary Fig. 3), indicating that the richness and evenness of the bacterial communities were not drastically affected by the nicotinamide intervention. Between-sample diversity analysis (β-diversity) revealed that there was a significant difference in participants receiving nicotinamide compared with those receiving placebo (PERMANOVA on between-sample Aitchison distances for intervention groups (*R*^2^ = 0.015, false discovery rate (FDR) = 0.002)), but not at baseline (*R*^2^ = 0.018, FDR = 0.99); however, the effect size was small (Fig. 2c and Supplementary Tables 18 and 19). Notably, the severity of COVID-19 symptoms and assignment to the placebo intervention group were correlated in their effects on the direction of β-diversity changes (Fig. 2c). We used variance partition analysis^21^ to assess how clinical covariates (for example, age, sex or bacterial genera) influence gut microbiome variation by intervention group. In this analysis, we found that—despite the significant differences between study arms—the contribution of individual taxa to shifts in β-diversity was subtle, suggesting considerable heterogeneity in the intervention effect at the taxonomic level (Fig. 2d and Supplementary Table 20).

**Fig. 3:**
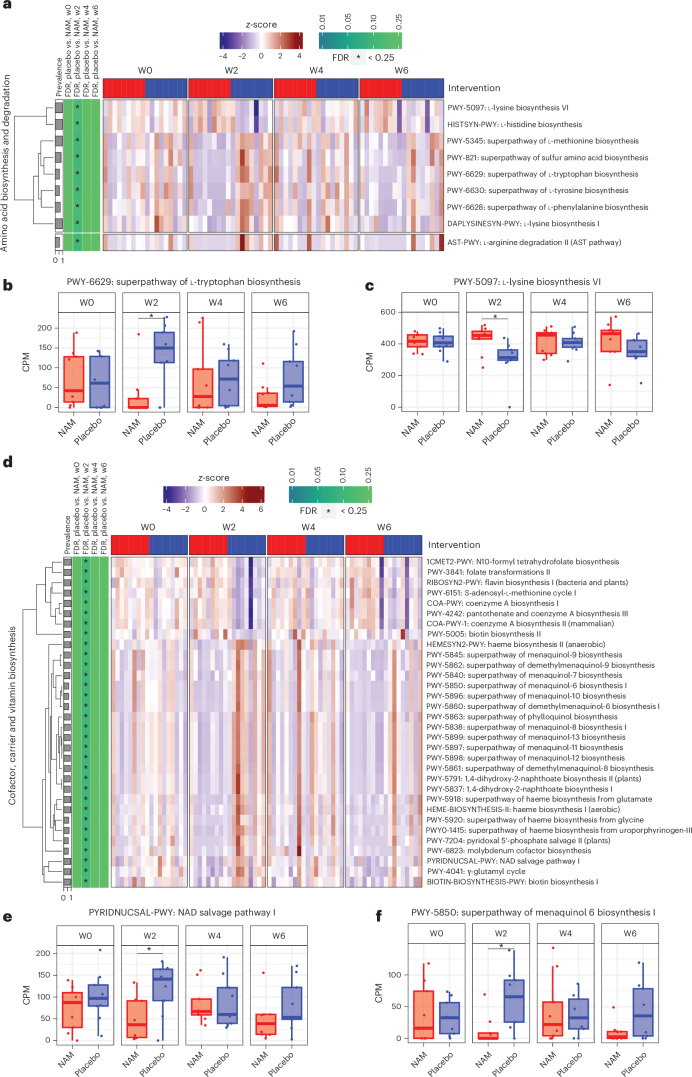
The metabolic potential of the faecal microbial communities is modified by nicotinamide. **a**, Heatmap of changes in significant amino-acid-related pathways found during a cross-sectional comparison of nicotinamide (NAM) versus placebo over time (*n* = 9 participants per group). There was an increase in tryptophan biosynthesis in participants receiving placebo compared with participants receiving nicotinamide at week 2. For each cell, colours indicate the *z*-score of the pathway abundance per sample, asterisks denote the significance of Benjamini–Hochberg-corrected *P* values (false discovery rate (FDR) < 0.25), and prevalence represents the percentage of non-zero features used in the comparison. **b**, Longitudinal plot of the counts per million (CPM) abundances of the tryptophan biosynthesis pathway (*n* = 9 per group; two-sided Wilcoxon rank-sum test, *P = 0.026, corrected for multiple comparisons). **c**, Longitudinal plot of the CPM abundances of the l-lysine biosynthesis pathway (*n* = 9 per group; two-sided Wilcoxon rank-sum test, *P = 0.014, corrected for multiple comparisons). **d**, Heatmap of changes in significant cofactor, carrier and vitamin-biosynthesis-related pathways found during a cross-sectional comparison of nicotinamide versus placebo over time (*n* = 9 per group). Similar to **a**, colours of cells indicate the *z*-score of the pathway abundance per sample, asterisks denote the significance of Benjamini–Hochberg-corrected *P* values (FDR < 0.25) and prevalence represents the percentage of non-zero features used in the comparison. **e**, Longitudinal plot of the CPM abundances of the NAD^+^ salvage pathway (*n* = 9 per group; two-sided Wilcoxon rank-sum test, *P = 0.024, corrected for multiple comparisons). **f**, Longitudinal plot of the CPM abundances of the menaquinol-6 biosynthesis pathway (*n* = 9 per group; two-sided Wilcoxon rank-sum test, *P = 0.013, corrected for multiple comparisons). Box plots show the median (centre line), IQR (box), 1.5 × IQR (whiskers) and outliers (points). Source data

We next aimed to understand the underlying functional differences using metagenomic pathway profiling. First, we inferred the presence and abundance of microbial taxa and community functions using the MetaPhlAn 3.0 and HUMAnN 3.0 (ref. ^22^) from metagenomics data. We found that, at week 2, the placebo group exhibited increased microbial biosynthesis pathways for tryptophan, phenylalanine, methionine and lysine, as well as enhanced redox and NAD^+^ salvage pathways, compared with the nicotinamide group. This suggests a relative deficiency in NAD^+^ de novo and tryptophan biosynthesis in participants receiving placebo, an effect prevented by nicotinamide supplementation (Fig. 3 and Supplementary Table 22).

**Fig. 4:**
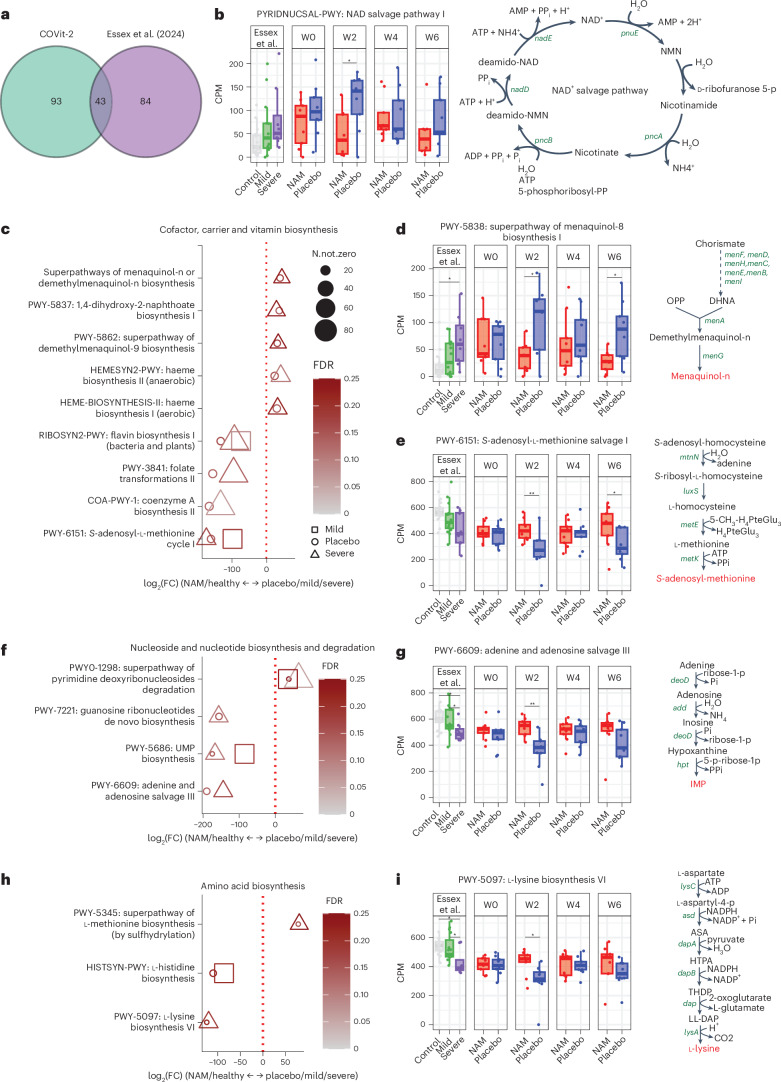
Differences in the functional potential of the gut microbiota between nicotinamide- versus placebo-receiving COVit-2 trial participants and healthy controls versus people with mild or severe COVID-19. **a**, Venn diagram showing 43 overlapping pathways between the COVit-2 trial cohort (green) and the public dataset from Essex et al^17^. (purple) among 220 significant pathways (false discovery rate (FDR) < 0.25). **b**, PYRIDNUCSAL-PWY pathway (NAD^+^ salvage pathway I) activity in healthy controls and patients with mild or severe COVID-19 (from ref. ^17^), and longitudinal samples (week (W) 0–6) from nicotinamide (NAM)- and placebo-receiving COVit-2 trial participants. The pathway was enriched in severe COVID-19 and in placebo participants. pnuE, NAD^+^ pyrophosphatase; pncA, nicotinamidase; pncB, nicotinate phosphoribosyltransferase; nadD, nicotinate-nucleotide adenyltransferase; nadE, NAD^+^ synthetase; Pi, phosphate; PPi, pyrophosphate. **c**, Differential pathway abundance plot for cofactor, carrier and vitamin biosynthesis pathways, comparing nicotinamide-receiving or healthy individuals (NAM/healthy) with placebo-receiving individuals or patients with mild or severe COVID-19 (placebo/mild/severe), respectively. **d**, PWY-5838 (superpathway of menaquinol-8 biosynthesis) abundances, enriched in the placebo and severe groups. **e**, PWY-6151 (*S*-adenosyl-l-methionine cycle I) abundances is enriched in nicotinamide-receiving individuals and in healthy individuals over time. **f**, Differential pathway abundance plot for nucleotide biosynthesis and degradation pathways, showing enriched pathways in NAM/healthy versus placebo/mild/severe groups. **g**, PWY-6609 (adenine and adenosine salvage III) abundances, enriched in the nicotinamide-receiving and healthy groups. **h**, Differential pathway abundance plot for amino acid biosynthesis pathways, showing pathways enriched in NAM/healthy versus placebo/mild/severe groups. **i**, PWY-5097 (l-lysine biosynthesis VI) abundances, enriched in the NAM, healthy and mild groups. Dot plots (**c**, **f**, **h**) represent significantly different pathways from MaasLin2 output (Supplementary Section 3.5), where log_2_(fold change (FC)) indicates enrichment in NAM/healthy (negative values) or placebo/mild/severe (positive values) groups. Symbol size reflects the number of samples in which the pathway was detected (N.not.zero), and the FDR significance is shown in the colour gradient. Box plots (**b**, **d**, **e**, **g**, **i**) show the median (centre line), IQR (box), 1.5 × IQR (whiskers) and outliers (points) of counts per million (CPM) abundance of pathways across healthy (*n* = 15), mild (*n* = 15) and severe (*n* = 8) groups from Essex et al.^17^, as well as nicotinamide (*n* = 9) and placebo (*n* = 9) groups from COVit-2 (two-sided Wilcoxon rank-sum test, *P < 0.05, **P < 0.01, corrected for multiple comparisons). Right panels in **b**, **d**, **e**, **g** and **i** show metabolic maps and key genes of the pathways. Source data

To further explore the potential influence of nicotinamide on COVID-19-associated gut microbiota changes, we compared our cohort with an independent gut microbiome dataset^17^. In that study, stool samples were collected from patients hospitalized with mild or severe COVID-19 and from uninfected matched control individuals. We analysed baseline and longitudinal samples from the public cohort to infer microbiome function, comparing key pathways altered by COVID-19 severity with those affected by nicotinamide versus placebo in our trial (for details, see Supplementary Section 3.5). We found an overlap of 43 pathways, mainly involved in cofactor, amino acid and nucleoside or nucleotide metabolism (Supplementary Fig. 8 and Supplementary Table 23). These pathways showed similar effect sizes when comparing healthy individuals versus those with COVID-19, and nicotinamide- versus placebo-receiving participants in the COVit-2 trial (Fig. 4). This finding suggests that nicotinamide intervention shifts the functional potential of gut microbiomes of people with COVID-19 towards that of healthy individuals, supporting the idea that nicotinamide protects against microbiota dysbiosis linked to COVID-19.

### Post-COVID syndrome

**Extended Data Fig. 7:**
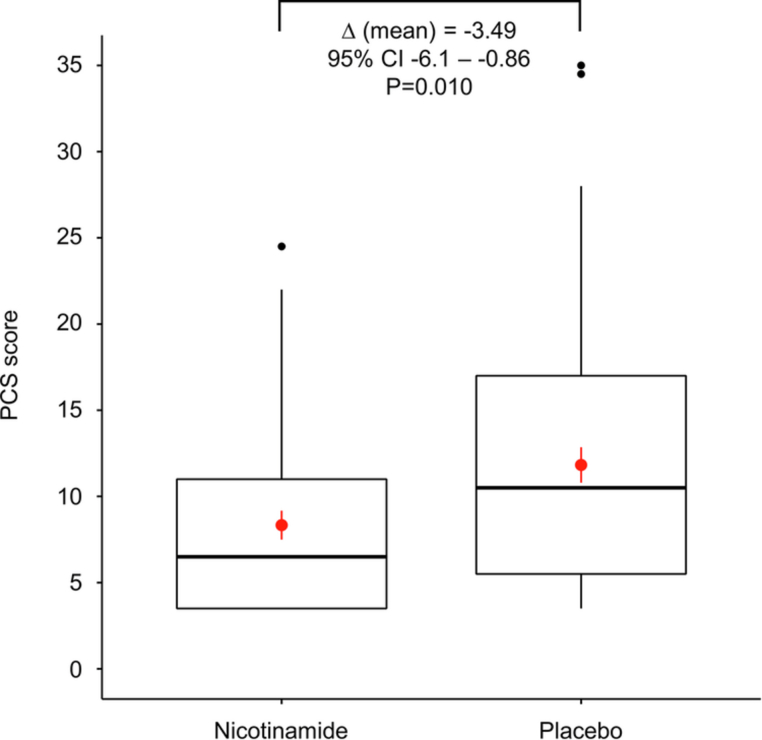
6-month follow-up for post-COVID syndrome (PCS) in responders to nicotinamide in the acute phase who were at risk for developing PCS. The box plots depict the PCS scores of 105 participants at risk for developing PCS with a PCS score >0, who had shown improvement in the primary endpoint or one of the three key secondary endpoints in the acute phase of the disease (nicotinamide: *n* = 48 [19 males, 29 females]; placebo: *n* = 57 [15 males, 42 females]). The median PCS score was 6.5 (quartile [Q]1: 3.5, Q3: 11.0) in participants who had received nicotinamide (males: 6.5 [3.5; 11.5], females: 6.5 [3.5; 11.0]) and 10.5 (Q1: 5.5, Q3: 17.0) in participants who had received placebo (males: 10.5 [5.25; 11.0], females: 10.75 [6.5; 17.0]). The mean PCS score ± s.e. was 8.33 ± 0.84 with nicotinamide (males: 8.37 ± 1.34, females: 8.31 ± 1.09) and 11.82 ± 1.03 with placebo (males: 9.67 ± 1.37, females: 12.60 ± 1.29). Box plots show the median (center line), interquartile range (IQR, box), 1.5x IQR (whiskers) and outliers (points). The means (red points) ± standard error (red whiskers) are shown within the box. Two-sided, unadjusted t-test for independent groups. Source data

The low severity of COVID-19 in the trial was associated with a low rate of PCS, as determined by the PCS score^23^ at the 6-month follow-up. The PCS score ranges from 0 to 59, with higher values indicating more severe PCS^23^. In the ITT population, only 47 participants in the nicotinamide arm and 51 in the placebo arm reached the threshold for moderate to severe PCS^23^. The mean PCS score was 2.95 (s.d., 5.91) in the nicotinamide arm and 3.19 ± 6.55 in the placebo arm (absolute difference, −0.24; 95% confidence interval, −1.1 to 0.61; *P* = 0.817). An exploratory analysis focused on participants at risk for developing PCS (nicotinamide: PCS score 3.97 ± 6.95; placebo: 4.81 ± 7.84; absolute difference, −0.85; 95% confidence interval, −2.4 to 0.69; *P* = 0.610) and on participants at risk who had shown improvement in the primary endpoint or one of the three key secondary endpoints in the acute phase of the disease (Supplementary Section 3.2). In the latter subgroup, a significant benefit of nicotinamide was also observed in participants with PCS (nicotinamide: *n* = 48, PCS score 8.33 ± 0.84; placebo: *n* = 57, PCS score 11.82 ± 1.03; absolute difference: −3.49; 95% confidence interval, −6.1 to −0.86; *P* = 0.010) (Extended Data Fig. 7).

### Safety

In the safety population, 1,798 adverse events (AEs) occurred in 317 (70.8%) of participants receiving nicotinamide, and 1,732 AEs occurred in 297 (65.7%) of participants receiving placebo (*P* = 0.115; Supplementary Table 25). Most AEs occurred early during the trial and were due to the onset or worsening of COVID-19-related symptoms. Notably, there were no significant differences between the two groups in this regard. Thus, these AEs are likely to have reflected the study set-up, with an early recruitment of participants during the incremental phase of the underlying infection. A trend towards a higher overall incidence of cumulative gastrointestinal AEs in the nicotinamide arm (25.2% versus 17.7% with placebo; unadjusted *P* = 0.007; without single gastrointestinal symptoms explaining this observation) is in line with the known side-effect profile of nicotinamide, for which abdominal discomfort has been described (AEs of special interest). These were mild and did not require further treatment. Sixteen participants were examined in an emergency department but were not hospitalized, seven were hospitalized (one with low-flow oxygen) and none died. All serious AEs were classified as unlikely to be related to the intervention, and their frequencies were highly similar between the two groups (Supplementary Tables 26–29).

## Discussion

COVID-19 is characterized by its large impact on health-related quality of life through a substantial symptom burden involving reduced physical performance, an inability to perform normal activities, airway symptoms and fatigue^24^. In the prospective, double-blind, randomized, placebo-controlled COVit-2 trial, we found that an intervention with nicotinamide (1,000 mg) in a combination of ileocolonic and systemic exposure leads to faster recovery from main COVID-19 symptoms. By week 2, recovery from reduced physical performance was 57.6% with the nicotinamide intervention and 42.6% with placebo intervention, resulting in a number needed to treat of seven.

The binary primary endpoint ‘performance drop’—a key symptom of COVID-19—affects people with the disease regardless of their disease course and physical fitness^25,26^. It has been widely documented, despite variability in its measurement^27^, as a multifactorial and sensitive metric for detecting impairments reported by individuals with COVID-19. The closely related key secondary endpoint ‘ability to perform normal activities’ and the secondary endpoint ‘shortness of breath’ support the validity of the measure. Notably, not all secondary endpoints, including resolution of fatigue by FACIT-F, were met, but FACIT-F is not validated for use in post-viral sequelae. Although return to work could not be measured owing to the prevailing quarantine regulations at the time of the trial (that is, taking participants out of contact until full recovery), we suggest that a faster regain of physical performance would also have translated into restoration of work productivity.

Our trial is in line with other observations showing that ‘real world’ mild-to-moderate COVID-19 results in a low frequency of clinically relevant PCS. However, in our population, we demonstrate a continued benefit of the intervention in participants at risk for developing PCS who had shown improvement while taking nicotinamide during acute COVID-19.

The COVit-2 trial recruited outpatients on the basis of SARS-CoV-2 test results through a network of laboratories. Although the recruitment strategy provided insights into ‘real world’ COVID-19 during a period when virtually all infections were caused by the Alpha (B.1.1.7) and Delta (B.1.617.2) variants of SARS-CoV-2 in Germany, it also resulted in a population with mild-to-moderate disease according to the World Health Organization’s scale of COVID-19 severity^28^. Therefore, hospitalization rates were low (seven participants), and progression to severe COVID-19 was observed in only one individual. Fatigue was generally mild and transient, with most participants in both trial arms having already returned to normal at week 2, which is in line with a recent, large and representative sample from the healthy German population (FACIT-F: 43.5 ± 8.3)^29^. Therefore, we regarded performance drop, despite its multifactorial nature, as a better endpoint than fatigue to measure the effects of early interventions in mild-to-moderate COVID-19.

In the COVit-2 population, AEs occurred in approximately 68% of participants. Most AEs occurred early and were due to the onset or worsening of COVID-19 symptoms. Notably, there was no significant difference in the overall frequency of AEs between the nicotinamide and placebo groups. Thus, these AEs are likely to reflect the study set-up, which involved early recruitment of participants during the incremental phase of COVID-19. Although nicotinamide is a vitamin that is generally recognized as safe and has a tolerable upper intake level of 900 mg day^–1^, our findings suggest that ileocolonic delivery resulting in high mucosal exposure does not substantially alter the known side-effect profile. Indeed, a phase I study including serial ileocolonoscopies in healthy participants (DRKS00023384, NCT05258474) did not find any mucosal irritation. Importantly, nicotinamide has no known substantial interactions with other drugs that might be administered to treat severe COVID-19.

Nicotinamide, a NAD^+^ precursor, has key functions in metabolism and cellular immunity, for example in enabling the function of poly-ADP-ribosyltransferases (PARPs) and suppressing viral replication in infected cells^1,2^. SARS-CoV-2 infection disturbs PARP expression patterns and depletes cellular NAD^+^ levels, which compromises antiviral defence^1,2,20^. The antiviral properties of nicotinamide have been described for diverse viruses, including human immunodeficiency virus^30^. Various publications on COVID-19 pathophysiology have suggested that nicotinamide and related substances should be examined as an intervention to replenish NAD^+^ in COVID-19 (refs. ^1,20,31^).

Our analyses of faecal microbiota changes align with findings from other studies^32–35^ reporting a complex dysbiosis associated with COVID-19. Although we observed only subtle shifts and higher heterogeneity in microbial taxa, it is important to note that many studies focused on severe cases, in which microbiota alterations are influenced by factors such as antibiotic use and invasive procedures^36^. Notably, our finding that microbiota changes are more pronounced at the functional level than in terms of gut taxonomic shifts is supported by another study investigating milder COVID-19 cases^37^. It also highlights the concept of ‘functional microbiota guilds,’ which exhibit consistent functional signals despite considerable taxonomic heterogeneity between individuals^38^. Preclinical studies clearly demonstrate the importance of the gut–lung axis in shaping protective immunity against respiratory infections, for example through circulating short-chain fatty acid levels (reviewed in ref. ^5^). Furthermore, evidence has been presented that the application of other gut microbiota-modulation principles, for example probiotics, might improve COVID-19 outcomes^39^. Our observation of gut microbiota shifts was derived from a representative, yet smaller, subcohort of the COVit-2 trial population, and might reflect both direct and indirect effects of nicotinamide, for example through modulation of the immune response or altered gut motility^1,3^. Although we cannot definitively establish a causal relationship, our observations of intervention-induced functional shifts in the gut microbiota following administration of placebo versus nicotinamide, particularly in relation to the healthy–mild–severe COVID-19 trajectory (for example, in amino acid and energy metabolic pathways) in a second cohort of patients with COVID-19 (ref. ^17^), support the hypothesis that nicotinamide might exert a beneficial impact through local intestinal mechanisms. Neither the observed effects on the microbiota nor the clinical effects were statistically different between non-smokers and smokers, the latter of whom might have systematic long-term metabolic adaptations affecting both host and microbiome systems^40,41^.

The trial has several limitations. Owing to the recruitment period, participants were almost exclusively infected with the Alpha (B.1.1.7) and Delta (B.1.617.2) variants. The remote nature of the trial and the quarantine rules did not allow us to measure lung function parameters and work-activity profiles. Given that we did not expect different results in vaccinated individuals and sought to minimize the need to account for additional covariates (for example, number of vaccinations, vaccine type), the COVit-2 trial, like many other studies, included only non-vaccinated participants, even as vaccines gradually became available. Although we cannot formally exclude the notion that SARS-CoV-2 variants or vaccination could have an influence on the observed effects, we anticipate from the general nature of the underlying distortion in NAD^+^–tryptophan homeostasis that our results could be extrapolated to other scenarios, for example current virus variants. Conclusions about PCS are limited because severe PCS cases were rare in the trial.

The clinical efficacy observed in the larger COVit-2 trial, which involved both conventional and gut-targeted nicotinamide release, aligns with the findings of the pilot trial COVit-1 in 56 participants. In COVit-1, differences between conventional nicotinamide tablets and an inactive comparator were, however, larger. The recruitment strategy for COVit-1, which involved physician practices instead of diagnostic laboratories, might have resulted in a participant population with more severe disease and higher rates of fever and pain than the cohort in COVit-2. We intentionally selected a combined intervention approach (conventional and gut-targeted) to provide a comprehensive evaluation of nicotinamide’s effects while ensuring baseline systemic availability. We acknowledge that this approach limits our ability to draw direct conclusions about the standalone effects of the novel gut-targeted formulation. Nevertheless, both the pilot study and the COVit-2 trial showed similar signatures of efficacy with regard to physical fitness in daily life.

Given that the metabolic mechanisms affected by nicotinamide are rather general, we anticipate that the findings from COVit-2 might also relate to other tryptophan-wasting conditions, including respiratory infections with other viruses or bacteria^7–9^. Moreover, lower levels of tryptophan might independently predict disease severity and short-term adverse outcomes not only in COVID-19 (refs. ^4,6^), but also in other settings such as community-acquired pneumonia^7^, and might identify people that could benefit from an intervention with nicotinamide.

In conclusion, we demonstrate the efficacy of nicotinamide administration to alleviate physical performance drop, a key symptom of COVID-19, which aligns with previous observations of NAD^+^ depletion in viral infections, as well as tryptophan degradation and altered host–microbial cometabolism as a systemic phenomenon in acute and chronic inflammation^17,42^. Further trials and mechanistic studies are needed to differentiate between systemic and gut-targeted delivery routes to firmly establish distinct clinical efficacy and clarify the precise mechanisms of action of the two routes.

## Methods

### Participants

The protocol (Supplementary Section 5), including all amendments, was approved by the Ethics Committee of the Medical Faculty of Kiel University (file reference A107/20), and all participants provided informed consent before any study procedures (see below). COVit-1 and COVit-2 were registered at the World Health Organization primary registry German Clinical Trials Register (DRKS00021214); COVit-2 was additionally registered at ClinicalTrials.gov (NCT04751604). In the COVit-1 trial, 56 outpatients with early symptomatic COVID-19 in domestic quarantine were recruited between 6 April 2020 and 28 January 2021, and 900 outpatients were enrolled into the COVit-2 trial between 1 February 2021 and 17 January 2022. Participants were compensated for their time according to the ethics committee’s approval (up to 265.00 € for interviews and questionnaires, up to 50.00 € for stool samples and up to 120.00 € for blood samples). Vaccinated individuals were excluded to avoid confounding (*f*or example, by inhomogeneous vaccination schedules or selection by age groups that received preferential access to vaccines). Self-reported demographics, including sex assigned at birth (male or female) or the gender option ‘diverse’ (not selected by any participant), were collected at screening. Owing to the lack of evidence for a sex-specific effect of nicotinamide in COVID-19 or similar infections, neither sex nor gender were specifically considered in the design of the trial, but were analysed in an exploratory fashion. No analysis of viral subtypes was performed, but with respect to population epidemiology in Germany, participants were almost exclusively infected by the wild-type virus in the COVit-1 trial and by the Alpha (B.1.1.7) or Delta (B.1.617.2) virotypes in the COVit-2 trial^43^.

For COVit-1, participants were recruited through outpatient facilities surrounding the University Hospital Schleswig-Holstein, whereas screening for COVit-2 was performed using diagnostic laboratories at 71 sites in Germany (Supplementary Section 3.1). Inclusion criteria for the overall population were ≥18 years of age, a recent SARS-CoV-2 infection (≤7 days after first positive test) and at least one symptom of COVID-19 on the day of randomization (Supplementary Section 3.2). Most participants reported five or more symptoms at baseline. Symptom load and the number of risk factors were similar in both trial arms and over the trial period (Supplementary Section 3.3). Exclusion criteria were current participation in another interventional study, pregnancy, breast-feeding and current or anticipated hospitalization. Inclusion into the acute RFITT primary-analysis population additionally required at least one risk factor for severe COVID-19 (Supplementary Section 3.4). At the 6-month follow-up, the ITT group was the primary analysis population, in which subgroups of participants at risk for developing PCS and responders to the intervention were further analysed (Supplementary Section 3.4).

### Randomization, masking and trial procedures

The trials were performed remotely owing to the contact restrictions, which were in place during the trial time period. For details on trial procedures and design, see Supplementary Section 3.1. Participants provided electronic written informed consent, which was confirmed by telephone. Whereas COVit-1 recruited participants through advertisements, COVit-2 identified candidates by contacting all patients who received a positive SARS-CoV-2 test result at 71 sites operated by German diagnostic laboratory service providers. After confirming eligibility, participants were computer-randomized and received the interventional product and paper questionnaires (SF-36 and FACIT-F), delivered by next-day courier. Eligible participants were randomly assigned in a 1:1 ratio to daily self-administration of either 1 g day^–1^ nicotinamide (500 mg immediate-release nicotinamide and 500 mg controlled-ileocolonic-release nicotinamide (CICR-NAM, Setamer®)) or matched placebo tablets in identical primary and secondary packaging, taken with breakfast for 4 weeks. Tablets were formally released by the pharmacy of the trial sponsor, the University Hospital Schleswig-Holstein (Kiel, Germany). The intervention received by a participant was not disclosed to personnel involved in the study; personnel responsible for clinical supply and safety were unblinded. Participants and personnel involved with participant care remained blinded throughout the study, including the 6-month follow-up. Participants underwent structured telephone interviews at weeks 2, 4 and 6, as well as 6 months after baseline. Optional stool samples were collected from participants by mail (Supplementary Section 3.5).

### Clinical outcomes

The original primary clinical outcome of COVit-1 was the rate of hospital admission for a minimum of 24 h of continuous oxygen therapy. Secondary endpoints included the rates of machine ventilation, intensive care and death, as well as time to resolution of symptoms (Supplementary Section 1). Owing to the results of the pilot experiment, COVit-2 focused on participant-reported COVID-19 symptom burden in the acute primary analysis RFITT population (ITT participants with at least one risk factor for severe COVID-19; Supplementary Section 3.4). The primary endpoint was restoration of physical performance at week 2. Key secondary endpoints were an improvement of the ability to perform normal activities, resolution of cough and resolution of fatigue at week 2. All endpoints were tested in participants with the respective symptoms at baseline. Prespecified subgroup analyses were performed for key risk factors. At the 6-month follow-up, the main outcome was PCS determined by a previously established PCS score, which was derived and validated in a large and prospective German cohort^23^. For the complete list of outcomes and for details on trial populations, see Supplementary Sections 3.2 and 3.4.

### Gut microbiome analyses

In COVit-2, 16S rDNA phylogenomic and metagenomic analyses were performed to investigate the effects of nicotinamide on gut microbial community composition and on functional metabolic capabilities of the colonic microbiome stratified by pathways, gene families and enzyme categories. Stool samples from 88 participants (70 participants for 16S and 18 participants for metagenomics) were analysed at week 0 (baseline), weeks 2 and 4 (exposure to nicotinamide or placebo) and week 6 (follow-up). For a detailed description of methods and references, see Supplementary Section 3.5.

### Safety

The safety population included all randomized participants. AEs were classified into preferred terms and summarized using MedDRA version 25.1. These AEs were reported in participant interviews conducted after baseline and through ad hoc reports from participants requesting medical consultation until week 6. Serious AEs were recorded by structured interview queries, with follow-up if necessary. All hospitalizations and emergency-room visits were recorded. Serious AEs related to the underlying disease were evaluated according to the World Health Organization’s COVID-19 scale^28^. For the safety analysis of the intervention, symptoms reported during the course of the study that were not present at baseline or were increased in severity were listed, and their frequency was compared between the nicotinamide and placebo groups using unadjusted Chi-square or Fisher’s exact tests.

### Primary statistical analysis

COVit-1 included 56 outpatients recruited from the referral network of the University Hospital Schleswig-Holstein and served to establish the rationale for the larger study, COVit-2. Statistics were descriptive because the study was a pilot trial (Supplementary Section 1). The results of the COVit-1 pilot trial were kept separate from those of the main trial, COVit-2. Statistical analysis was conducted in the blinded dataset by a third-party provider with established clinical trial statistics expertise (Novustat).

The COVit-2 trial enrolled 900 participants, following the sample size assumptions detailed in Supplementary Section 3.6. A pre-planned futility analysis was conducted by the Data Management Board after 400 participants had been recruited (Supplementary Sections 3.6 and 6). The full analysis set (the ITT population) included all participants who received at least one dose of nicotinamide or placebo. The RFITT population was defined as all participants in the ITT population with at least one symptom, demographic characteristic or underlying medical condition that was previously associated with an increased risk of developing severe COVID-19. The respective per-protocol populations, PP and RFPP, excluded those who dropped out or failed to comply with the investigational product intake for at least 80% of the study duration (Supplementary Section 3.4). In the RFITT population, each analysis regarding resolution or improvement of a symptom was performed only for those participants who reported the respective symptom at baseline. In case of ordinal queries (Supplementary Section 3.2), participants with severe symptoms (a value of >3) at baseline were selected for analysis. For the FACIT-F and SF-36 questionnaires, only those with severe complaints (baseline values ≤ median) were included in the analyses.

Baseline characteristics were summarized according to trial group and overall, with the use of descriptive statistics for continuous and categorical measures.

Primary and confirmatory secondary binary endpoints were analysed by assessing changes from baseline for all weeks within each intervention group, compared using the Cochran–Mantel–Haenszel test. Post hoc analyses for each week were calculated using Fisher’s exact test, with Benjamini–Hochberg adjustment for multiple testing. The Woolfe test was performed to test for homogeneity of odds ratios across time. If significant *P* values were obtained from the Woolfe test, the Cochran–Mantel–Haenszel test would not be appropriate. In this case, Fisher’s exact tests for each timepoint were used instead of the Cochran–Mantel–Haenszel test. A continuity correction was applied for zero frequencies.

Continuous secondary outcomes (scales, SF-36 and FACIT-F) were analysed with the use of a mixed model for repeated measures (MMRM), with the change from baseline at each of the three scheduled post-baseline time points (2, 4 and 6 weeks) as the dependent variable and baseline value, intervention group, time and time–intervention interaction as independent variables (Supplementary Section 6). Statistical methods that do not involve imputation, such as the MMRM or the Chi-square test, were used for the analyses. The use of the MMRM model assumes implicitly that data are missing at random.

For time-to-event analyses, a Kaplan–Meier approach was used. The log rank test was performed to test whether time to event differed between intervention groups. A Cox proportional-hazards model was used to evaluate and estimate the impact of the intervention group. The assumption of proportional hazards was analysed before the model was applied.

The reliability of the FACIT-F and SF-36 questionnaires was assessed using Cronbach’s alpha coefficient (*α* ≥ 0.80) for internal consistency and item-to-total correlations exceeding 0.20. The average variance extracted was calculated to assess discriminant validity.

We applied multivariable generalized linear models, using a binomial family with a log link for binary endpoints and a Gaussian family for endpoints measured on a scale of complaints, to assess the impact of sex. Treatment, change from baseline, baseline value, sex and treatment–time and treatment–sex interactions were considered as independent factors. Sex-specific subgroup analyses were performed as part of the exploratory analyses. Odds ratios and 95% confidence intervals were calculated for binary endpoints, and Hedges’ *g* effect sizes including 95% confidence intervals were calculated for ordinal scales of complaint. Additional analyses were performed in accordance with the analyses of the entire RFITT population.

We assessed normality using the Shapiro–Wilk test as well as histograms, and homogeneity of variances using Levene’s test. The results of these tests confirmed that the assumptions of the statistical tests were met. No data points were excluded from the analyses, as we also used MMRM and generalized linear models, which account for all available data without explicit exclusions. Detailed descriptions of the study populations for specific analyses are available in the statistical analysis plans (Supplementary Sections 6 and 7).

All statistical analyses were performed with the use of R software (R Foundation for Statistical Computing, 2021) version 4.1.2 or higher. For further details on the statistical analyses, see Supplementary Sections 3.5, 3.6, 6 and 7.

### Exploratory statistical analysis of occurrence of PCS

For the 6-month follow-up, the PCS score^23^ served as the primary efficacy variable and was compared between the intervention groups using a *t*-test or a nonparametric Mann–Whitney *U* test. Subgroup analyses were performed to further define responders in defined risk groups. For further details on the statistical analyses, see Supplementary Section 7.

### Quality-control measures

Blinded (recruiters, interviewers, study physicians, statisticians, technicians and scientists for microbiome analysis) and unblinded (study material distribution, safety) personnel were strictly separated. All personnel completed documented formal monitored training on trial procedures, and delegation logs were adapted from good-clinical-practice guidelines. Guided standard operating procedures were regularly retrained, and detailed instructions for participants were implemented to ensure the validity of assessing participant-reported outcomes through structured telephone interviews (Supplementary Sections 3.1 and 3.2). Key interview questions were redundant, and source data entry into the database was monitored. SARS-CoV-2 test results were verified. Compliance was surveyed by remote tablet count during each interview and through specific questions (Supplementary Section 3.1). Side effects were queried and coded according to MedDRA Version 25.1 (see above).

### Reporting summary

Further information on research design is available in the Nature Portfolio Reporting Summary linked to this article.

## Supplementary information

Supplementary Information Supplementary Sections 1–7, Tables 1–29, Figs. 1–12 and References.

Reporting Summary

## Source data

Source Data Fig. 1 Statistical source data.

Source Data Fig. 2 Statistical source data.

Source Data Fig. 3 Statistical source data.

Source Data Fig. 4 Statistical source data.

Source Data Extended Data Fig. 3 Statistical source data.

Source Data Extended Data Fig. 4 Statistical source data.

Source Data Extended Data Fig. 5 Statistical source data.

Source Data Extended Data Fig. 6 Statistical source data.

Source Data Extended Data Fig. 7 Statistical source data.

## Data Availability

The COVit-1 pilot trial and the COVit-2 main trial were preregistered with a data sharing statement at the WHO primary registry German Clinical Trials Register (DRKS00021214). COVit-2 was additionally registered with ClinicalTrials.gov (NCT04751604). The trial protocol and statistical analysis plans are available in the Supplementary Information. The microbiome sequencing reads have been deposited and are available at ENA under the accession code PRJEB61276 (last accessed on 11 March 2025). The taxonomic classification of 16S rRNA data, performed using the SILVA database (version 138) is publicly available at Zenodo^44^: https://zenodo.org/records/6395539 (last accessed on 11 March 2025). Clinical data are not available for download owing privacy law according to the European Union’s General Data Protection Regulation (EU GDPR) and ethical restrictions. Specific requests by academic researchers for access to clinical data can be addressed to the corresponding author. These data include individual deidentified participant data and data sorted by sex and diversity. On the basis of such a request including a detailed analysis plan, access might be provided, subject to a decision of the Ethics Committee of the Medical Faculty of Kiel University to ensure compliance with privacy laws, data protection and requirements for consent and anonymization. Requests will be considered from the date of publication of this article. It is expected that data can be obtained within 90 days after the eventual ethics vote. Data will be available for ten years. Source data are provided with this paper.
