# Supplementary material for: Nicotinamide modulates gut microbial metabolic potential and accelerates recovery in mild-to-moderate COVID-19
Source: Nat Metab. 2025 May 12;7(6):1136–49. doi: 10.1038/s42255-025-01290-1 (PMC12198012; doi:10.1038/s42255-025-01290-1)
Supplement: Supplementary file 1 — Supplementary Sections 1–7, Tables 1–29, Figs. 1–12 and References. [file 42255_2025_1290_MOESM1_ESM.pdf]

nature metabolism

Supplementary information https://doi.org/10.1038/s42255-025-01290-1

Nicotinamide modulates gut microbial metabolic potential and accelerates recovery in mild-to-moderate COVID-19

In the format provided by the authors and unedited

SUPPLEMENT

1. Summary of the pilot trial COVit-1 3

Supplementary Table 1: Demographic and clinical characteristics of all patients at baseline (intention-to-treat population) in the pilot trial COVit-1. 3

Supplementary Fig. 1: Number of symptoms reported at baseline in the intention-to-treat population of the pilot trial COVit-1 4

Supplementary Table 2: Baseline symptoms of the intention-to-treat population of the pilot trial COVit-1 compared to symptom frequencies in the COVit-2 trial. 4

Supplementary Fig. 2*:* Results of the primary and secondary endpoints of COVit-2 in a post-hoc analysis of the COVit-1 pilot trial intention-to-treat population. 5

2. Supplementary results for COVit-2 6

2.1. Demographic and clinical characteristics at baseline 6

Supplementary Table 3: Demographic and clinical characteristics of the patients at baseline (RFITT population). 6

2.2. Supplementary clinical data (tables) 7

Supplementary Table 4: Descriptive statistics of SF-36 scales in the RFITT population. 7

Supplementary Table 5: Descriptive statistics of FACIT-F scales in the RFITT population. 9

Supplementary Table 6: Effect of sex on key binary endpoints in the RFITT population. 11

Supplementary Table 7: Effect of sex on key ordinal endpoints in the RFITT population. 11

Supplementary Table 8: Effects of nicotinamide on COVID-19 symptoms (change to baseline) in the entire RFITT population (binary symptoms). 12

Supplementary Table 9: Effects of nicotinamide on COVID-19 symptoms (change to baseline) in males of the RFITT population (binary symptoms). 16

Supplementary Table 10: Effects of nicotinamide on COVID-19 symptoms (change to baseline) in females of the RFITT population (binary symptoms). 20

Supplementary Table 11: Effects of nicotinamide on COVID-19 symptoms (change to baseline) in the entire RFITT population (complaint scale). 24

Supplementary Table 12: Effects of nicotinamide on COVID-19 symptoms (change to baseline) in males of the RFITT population (complaint scale). 25

Supplementary Table 13: Effects of nicotinamide on COVID-19 symptoms (change to baseline) in females of the RFITT population (complaint scale). 26

Supplementary Table 14: Effects of nicotinamide on SF-36 scales (change to baseline) in the RFITT population. 27

Supplementary Table 15: Effects of nicotinamide on FACIT-F scales (change to baseline) in the RFITT population. 28

2.3. Supplementary clinical data (text) 29

2.4. Supplementary data from gut microbiome analyses 30

Supplementary Fig. 3: α-diversity metrics in relation to the intervention. 31

Supplementary Fig. 4 | Principal Coordinate Analysis (PCoA) based on Bray-Curtis distances showing microbial community composition changes in patients with key COVID-19-related symptoms. 32

Supplementary Fig. 5: Variance contribution of baseline characteristics. 34

Supplementary Fig. 6: Longitudinal analysis of α-diversity in relation to fever in patients with key COVID-19-related symptoms. 35

Supplementary Fig. 7 | Variance Partition Analysis (VPA) of microbial genera and comparison of 50 top genera identified in 16S rRNA gene sequencing and shotgun metagenomics. 36

Supplementary Fig. 8 | 43 functional pathways overlap in the gut microbiota of healthy control individuals vs. patients with mild or severe COVID-19 in the public dataset from Essex et al. and nicotinamide (NAM)-receiving vs. placebo-receiving participants in COVit-2. 37

Supplementary Table 16: Demographic and clinical characteristics at baseline of the 16S phylogenomic subcohort (n=70)........ 38

Supplementary Table 17: Demographic and clinical characteristics of the metagenomics subcohort (n=18).. 38

Supplementary Table 18: PERMANOVA results of the influence of age, key COVID-19-related symptoms and intervention on the gut microbial composition of the 16S phylogenomic subcohort (n=70) at week 2 and week 4. 39

Supplementary Table 19: PERMANOVA results of the influence of age and intervention on the gut microbial composition of the 16S phylogenomic subcohort (n=70) at baseline. 39

Supplementary Table 20: Proportion of variation explained by each feature and confounder in the 16S phylogenomic subcohort (n=70) at weeks 2 and 4, based on 16S data........ 40

Supplementary Table 21: Proportion of variation explained by each feature and confounder in the metagenomics subcohort (n=18) at weeks 2 and 4, based on metagenomics data........ 48

Supplementary Table 22: Maaslin2 output of the significantly differentially abundant pathways found in the nicotinamide vs. placebo comparisons in the metagenomics subcohort (n=18). Only significant features were found at week 2 (FDR<0.25). 52

Supplementary Table 23: Maaslin2 output of overlapping pathways between the Essex et al. cohort (n=38) and the COVit-2 metagenomics subcohort (n=18). 59

Supplementary Table 24: Mean variance contribution of patients' covariates at baseline. 64

2.5. Safety data 65

Supplementary Table 25: Adverse events sorted by MedDRA System Organ Class and Preferred Term. 65

Supplementary Table 26: Severe COVID-19, Serious Adverse Events and emergency treatment in the safety population at week 0 (baseline). 72

Supplementary Table 27: Severe COVID-19, Serious Adverse Events and emergency treatment in the safety population at week 2. 72

Supplementary Table 28: Severe COVID-19, Serious Adverse Events and emergency treatment in the safety population at week 4. 73

Supplementary Table 29: Severe COVID-19, Serious Adverse Events and emergency treatment in the safety population at week 6. 73

3. Supplementary information on methods for COVit-2 74

3.1. Trial procedures and design of COVit-2 74

3.2. Lists of COVID-19 symptoms queried by telephone interviews in COVit-2........ 76

3.3. Symptoms and risk factors at baseline in COVit-2 77

Supplementary Fig. 9: Number of symptoms reported at baseline in the RFITT population. 77

Supplementary Fig. 10: Intensity of reduced ability to perform normal activities reported at baseline in the RFITT population. 77

Supplementary Fig. 11: Intensity of cough reported at baseline in the RFITT population. 78

Supplementary Fig. 12: Number of risk factors for severe COVID-19 reported at baseline in the RFITT population. 78

3.4. Analysis populations and risk factor subgroups in COVit-2 79

3.5. Methods for gut microbiome analyses in COVit-2 81

3.6. Sample size and futility analysis in COVit-2 83

4. Supplementary references 84

5. Trial protocols 85

6. Statistical analysis plan for acute COVID-19 148

7. Statistical analysis plan for the 6-month follow-up 184

1. Summary of the pilot trial COVit-1

The pilot trial COVit-1 was a double-blind, randomized, controlled, monocentric trial conducted at the Department of Internal Medicine I of the University Hospital Schleswig-Holstein (Kiel, Germany). The protocol (see section 5 of this Supplement) including all amendments was approved by the Ethics Committee of the Medical Faculty of Kiel University (file reference A107/20) and all patients gave informed consent prior to any study procedures (see below). COVit-1 and COVit-2 were registered with the WHO primary registry German Clinical Trials Register (DRKS00021214). The trial was conducted remotely (while patients were confined to domestic quarantine) using the same procedures as described for COVit-2, but without biosampling. The trial population of COVit-1 is slightly different from the population of COVit-2 due to the recruitment of participants from physician's practices in the referral network of the University Hospital Schleswig-Holstein. In contrast to recruiting via diagnostic laboratories as in COVit-2, this may have resulted in a more severely affected patient population.

In the COVit-1 trial, 56 outpatients mainly from the Kiel area with early symptomatic COVID-19 in domestic quarantine were recruited from 06 April 2020 to 28 January 2021. The trial was double blind and patients were randomized 1:1 to either receive orally 1,000 mg of nicotinamide (NicoPel®, Derma Enzinger, Ainring, Germany) or 245 mg silica (Salus Pharma, Bruckmühl, Germany). The study intervention was taken once daily with breakfast in the morning, for 4 weeks (28 days).

Inclusion criteria were a laboratory-confirmed SARS-CoV-2 infection, COVID-19 symptoms in the respiratory or gastrointestinal tract and an age of ≥ 18 years. There were no formal exclusion criteria. Supplementary Table 1 shows the baseline characteristics of the COVit-1 trial population (intention-to-treat, ITT) in the same format as used for COVit-2 (Table 1). Virus variants were not systematically determined, but the timeframe of recruitment of COVit-1 comprised mostly the wildtype strain of the pandemic, as the Alpha variant (B.1.1.7) was first detected in the recruitment area in the second week of January, 2021.

Supplementary Table 1: Demographic and clinical characteristics of all patients at baseline (intention-to-treat population) in the pilot trial COVit-1.

Characteristics	Nicotinamide (n=28)	Control (n=28)	Total (n=56)
Median age (range) at randomization – yr	40 (18–69)	46 (20–58)	42 (18–69)
Sex – no. of patients (%)			
Male	11 (39.3)	10 (35.7)	21 (37.5)
Female	17 (60.7)	18 (64.3)	35 (62.5)
Race or ethnic group – no. of patients (%)			
White	28 (100)	25 (89.3)	53 (94.6)
Other	0 (0)	3 (10.7)	3 (5.4)
Not reported	0 (0)	0 (0)	0 (0)
Body mass index (BMI) (mean ± SD)	26.1 (4.5)	26.3 (6.6)	26.2 (5.6)
Risk factors for severe COVID-19 – no. (%)			
Age ≥ 60 yr	4 (14.3)	0 (0)	4 (7.1)
BMI ≥ 30 and/or type 2 diabetes	4 (14.3)	5 (17.9)	9 (16.1)
Cardiovascular diseases, high blood pressure or stroke	7 (25.0)	5 (17.9)	12 (21.4)
Asthma, COPD or other chronic lung diseases	6 (21.4)	0 (0)	6 (10.7)
Current or former smokers	18 (64.3)	23 (82.1)	41 (73.2)
Other risk factors	10 (35.7)	11 (39.3)	21 (37.5)
At least one risk factor	26 (92.9)	27 (96.4)	53 (94.6)

As shown in Supplementary Table 1, 26/28 (93%) in the nicotinamide and 27/28 (96%) of the patients in the control group of the COVit-1 trial had at least one risk factor for severe COVID-19, although this was not an inclusion criterion. In COVit-2, patients with at least one risk factor for severe COVID-19 were less frequent. The RFITT population of the COVit-2 trial comprised 248/430 (58%) of such patients in the nicotinamide group and 252/437 (also 58%) in the placebo group (cf. Table 1, Supplementary Table 3 and Extended Data Fig. 2). This difference in patient attraction was probably due to the different recruitment strategies: physician recruitment in COVit-1, enlisting participants who had shown up at local therapeutic facilities including the emergency rooms but then remained outpatients, is contrasted by COVit-2 with recruitment of patients who freshly received a positive test result for SARS-CoV-2 infection from a diagnostic laboratory (see section 3.1 of this Supplement).

The patients in the COVit-1 ITT population (n=56) showed a different symptomatology compared to the patients in the COVit-2 ITT (n=867) with significantly more fever and pain at baseline (Supplementary Table 2). Interestingly, the number of symptoms per patient at baseline was only slightly higher in COVit-1 (Supplementary Fig. 1) in comparison with COVit-2 (Supplementary Fig. 9). The recruitment period of COVit-2 from 01 February 2021 until 17 January 2022 covered the end of the Alpha (B.1.1.7) wave, the entire Delta (B.1.617.2) wave and the beginning of the first Omicron (B.1.1.529) wave in Germany.

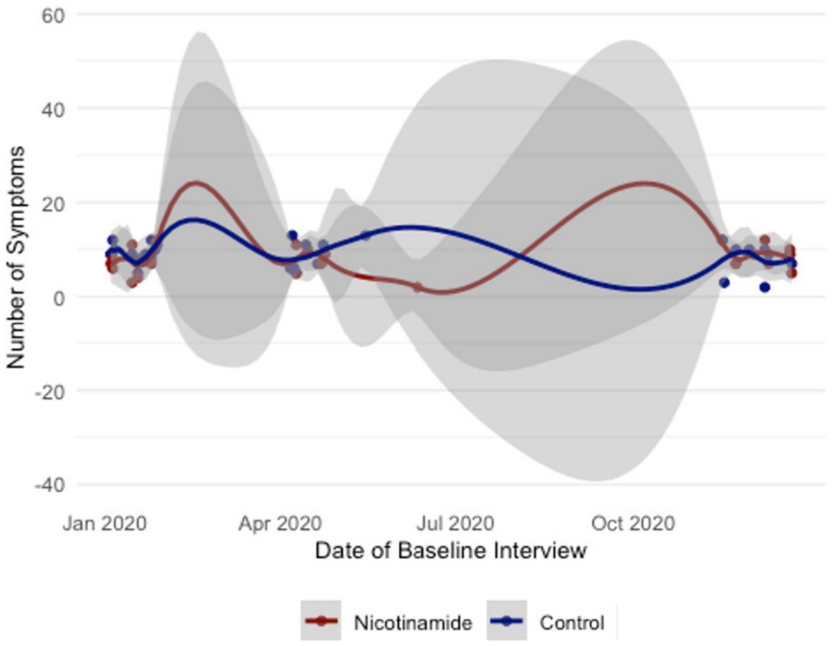

Supplementary Fig. 1: Number of symptoms reported at baseline in the intention-to-treat population of the pilot trial COVit-1.
Each dot represents one patient (n=56). Lines were produced with locally weighted scatterplot (LOESS) smoothing in R. Grey shades are 95% CI bounds of LOESS smoothing.

Supplementary Table 2: Baseline symptoms of the intention-to-treat population of the pilot trial COVit-1 compared to symptom frequencies in the COVit-2 trial.

Symptoms	Nicotinamide (n=28) no. (%)	Control (n=28) no. (%)	P*	COVit-1 ITT (n=56) no. (%)	COVit-2 ITT (n=867) no. (%)	P**
Fatigue	23 (82.1)	26 (92.9)	0.422	49 (87.5)	663 (76.5)	0.0696
Performance drop	23 (82.1)	23 (82.1)	1.000	46 (82.1)	617 (71.2)	0.0913
**Fever*** **	**14 (50.0)**	**17 (60.7)**	**0.420**	**31 (55.4)**	**117 (13.5)**	**<0.0001**
Shortness of breath	8 (28.6)	18 (35.7)	0.567	26 (46.4)	268 (30.9)	0.0182
Cough	20 (71.4)	16 (57.1)	0.265	36 (64.3)	426 (49.1)	0.0378
Cough with sputum	7 (25.0)	9 (32.1)	0.554	16 (28.6)	312 (36.0)	0.3138
Head cold (rhinorrhea)	22 (78.6)	22 (78.6)	1.000	44 (78.6)	634 (73.1)	0.4366
Sore throat	10 (35.7)	21 (75.0)	0.003	31 (55.4)	334 (38.5)	0.0160
**Muscle pain**	**14 (50.0)**	**11 (39.3)**	**0.420**	**25 (44.6)**	**250 (28.8)**	**0.0156**
**Joint pain**	**10 (35.7)**	**13 (46.4**	**0.415**	**23 (41.1)**	**177 (20.4)**	**0.0007**
**Chest pain**	**7 (25.0)**	**11 (39.3)**	**0.252**	**18 (32.1)**	**156 (18.0)**	**0.0130**
**Headache**	**22 (78.6)**	**24 (85.7)**	**0.729**	**46 (82.1)**	**398 (45.9)**	**<0.0001**
Diarrhea	8 (28.6)	5 (17.9)	0.342	13 (23.2)	117 (13.5)	0.0486
Vomiting	1 (3.6)	5 (17.9)	0.193	6 (10.7)	5 (0.6)	<0.0001
Loss of appetite	12 (42.9)	17 (60.7)	0.181	29 (51.8)	414 (47.8)	0.5832
Impaired sense of smell	17 (60.7)	14 (50.0)	0.420	31 (55.4)	425 (49.0)	0.4087
Impaired sense of taste	18 (64.3)	14 (50.0)	0.280	32 (57.1)	395 (45.6)	0.0984

* COVit-1: nicotinamide vs. control; Chi-square test (one-sided) or Fisher exact test (two-sided), unadjusted.
** COVit vs. COVit-2, Fisher exact test (two-sided, unadjusted).
*** Missing temperature for 1 patient allocated to nicotinamide in COVit-1.
Bold, fever and pain symptomatology.

COVit-1 was started in the first weeks of the pandemic, when the number of patients requiring hospitalization and the numbers of infections were expected rise significantly. The planned primary endpoint of the COVit-1 pilot trial was the frequency of hospital admission in order to receive at least 24 h of continuous oxygen therapy, and secondary endpoints included frequencies of machine ventilation, intensive care, death as well as time to resolution of symptoms. As blinded frequency analyses showed that the primary endpoint did not occur in a sufficiently high frequency, because the COVID-19 disease courses did not result in enough intensive care admissions and deaths, the trial was stopped. Symptom-related endpoints were observed in frequencies necessary for a statistical analysis, and complete resolution of symptoms after 2 weeks was defined as the principal goal for an exploratory, post-hoc

statistical analysis. Therefore, the COVit-1 trial was a pilot experiment. Protocol amendments were approved by the Ethics Committee and recruitment seamlessly continued with COVit-2 becoming a trial with a second patient population. Endpoints were adapted to the evolving real-world clinical situation, the number of recruitment sites was increased, and molecular analyses were included. The populations in COVit-1 and COVit-2 are completely separated and were not analyzed together.

Symptom frequencies and intensities at week 2 compared to baseline were more strongly reduced in the nicotinamide group than in the control group. The results for the post-hoc endpoints (cf. COVit-2 primary and key secondary endpoints) are shown for the COVit-1 ITT population (n=56) in Supplementary Fig. 2. Of note, complete resolution of symptoms after 2 weeks was substantially higher in the nicotinamide group (35.7% of patients) than in the control group (14.3%). The 35 female participants were main contributors to the difference between the nicotinamide and control interventions (P=0.043). Taken together, the results from COVit-1 warranted investigation of nicotinamide versus placebo in a larger and more definite trial.

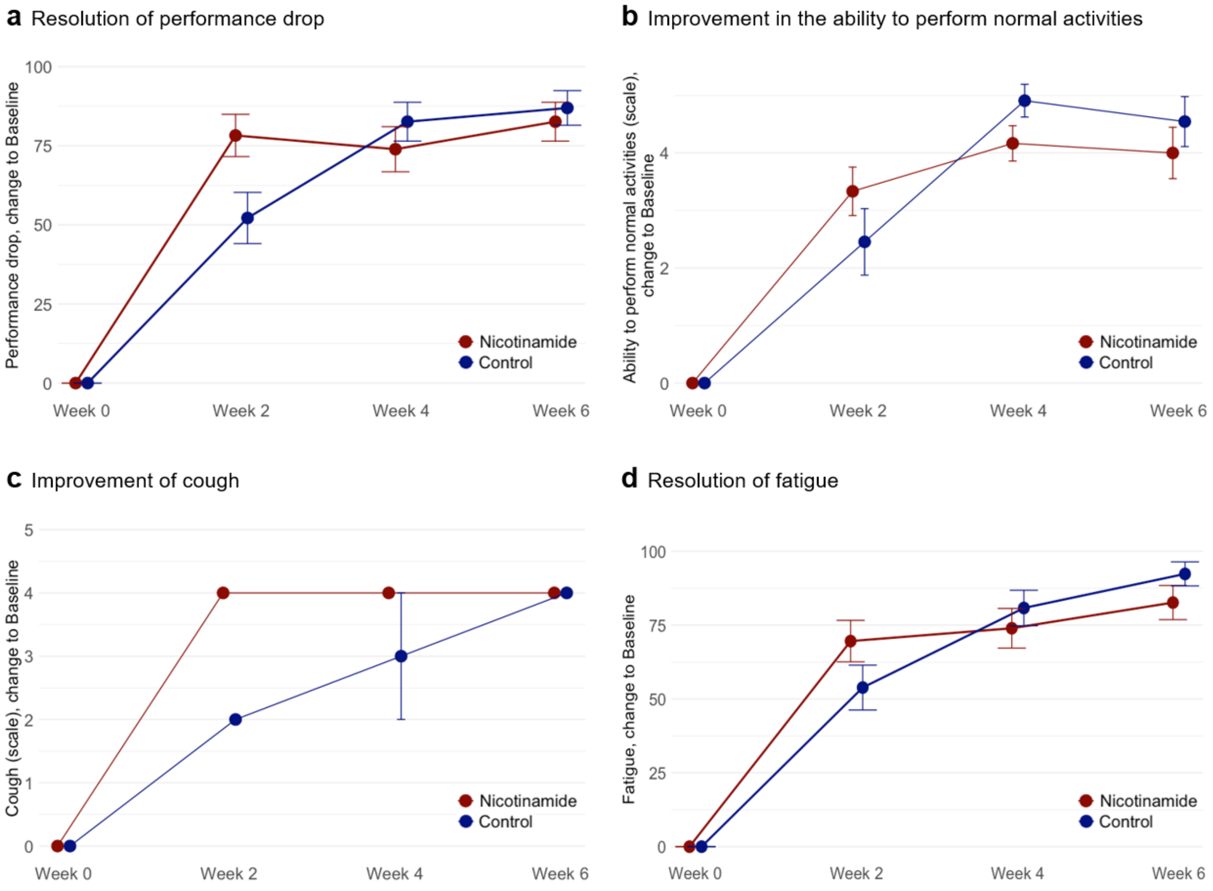

Supplementary Fig. 2*:* Results of the primary and secondary endpoints of COVit-2 in a post-hoc analysis of the COVit-1 pilot trial intention-to-treat population.
Statistically significant differences (post-hoc) were observed for the resolution of performance drop (panel **a**, P=0.034) and the improvement in the ability to perform normal activities (panel **b**, P=0.045). In panel **c**, a standard error is only calculated for week 4 in the control group due to the low number of patients with severe cough at baseline (1 patient receiving nicotinamide and 2 patients receiving inert control, with scale improvements of -4 and -2 at week 4 compared to baseline, respectively). For the numbers of patients with the respective symptoms at baseline, see Supplementary Table 2. Graphs represent relative frequency ± standard deviation (**a**, **d**) or mean ± standard error (**b**, **c**).

2. Supplementary results for COVit-2

2.1. Demographic and clinical characteristics at baseline

Supplementary Table 3: Demographic and clinical characteristics of the patients at baseline (RFITT population).

Characteristics	Nicotinamide (n=248)	Placebo (n=252)	Total (n=500)
Median age (range) at randomization – yr	38 (18–75)	39 (18–70)	39 (18–75)
Sex – no. of patients (%)			
Male	101 (40.7)	108 (42.9)	209 (41.8)
Female	147 (59.3)	144 (57.1)	291 (58.2)
Race or ethnic group – no. of patients (%)			
White	238 (96.0)	243 (96.4)	481 (96.2)
Other	10 (4.0)	9 (3.6)	19 (3.8)
Not reported	0 (0)	0 (0.0)	0 (0)
Body mass index (BMI) (mean ± SD)	26.3 ± 5.5	27.2 ± 5.4	26.8 ± 5.5
Risk factors for severe COVID-19 – no. (%)			
Age ≥ 60 yr	22 (8.9)	20 (7.9)	42 (8.4)
BMI ≥ 30 and/or type 2 diabetes	58 (23.4)	73 (29.0)	131 (26.2)
Cardiovascular diseases, high blood pressure or stroke	63 (25.4)	60 (23.8)	123 (24.6)
Asthma, chronic obstructive pulmonary disease or other chronic lung diseases	41 (16.5)	52 (20.6)	93 (18.6)
Current or former smokers	165 (66.5)	171 (67.9)	336 (67.2)
Other risk factors	21 (8.5)	15 (6.0)	36 (7.2)

The primary analysis population RFITT consists of patients with at least one risk factor for severe COVID-19 (see section 3.4 of this Supplement).

2.2. Supplementary clinical data (tables)

Supplementary Table 4: Descriptive statistics of SF-36 scales in the RFITT population.

Time	Scale	Nicotinamide (n=248)		Placebo (n=252)		Total (n=500)	
		Mean	Standard deviation	Mean	Standard deviation	Mean	Standard deviation
Week 0	Physical functioning	53.50	27.50	51.19	26.80	52.09	27.21
	Role functioning/physical	48.68	45.19	53.04	44.43	50.73	44.83
	Role functioning/emotional	69.87	40.93	70.95	40.26	69.69	40.83
	Energy/fatigue	50.72	21.20	52.17	21.66	50.97	21.52
	Emotional well-being	67.42	18.09	69.20	17.78	67.77	18.10
	Social functioning	62.20	30.23	63.32	29.81	62.64	29.99
	Pain	64.46	29.13	64.51	30.33	64.02	29.78
	General health	60.02	18.31	59.82	18.38	59.72	18.37
	Health change	23.80	23.02	23.36	25.24	23.42	24.03
Week 2	Physical functioning	75.25	25.03	74.13	26.17	74.11	25.85
	Role functioning/physical	34.25	40.99	28.97	38.78	31.55	40.04
	Role functioning/emotional	57.21	44.82	56.23	44.99	56.92	44.93
	Energy/fatigue	44.48	22.43	44.02	20.67	43.97	21.57
	Emotional well-being	65.62	18.73	66.29	18.69	65.55	18.84
	Social functioning	51.20	29.36	50.18	28.06	50.48	28.71
	Pain	58.49	27.88	55.68	28.18	57.04	28.09
	General health	63.91	18.65	62.53	18.99	62.74	19.02
	Health change	33.17	22.10	33.06	23.16	32.90	22.62
Week 4	Physical functioning	86.19	19.11	85.28	20.32	84.96	20.40
	Role functioning/physical	51.84	43.17	47.20	43.78	48.89	43.62
	Role functioning/emotional	68.11	42.13	66.04	42.97	66.52	42.87
	Energy/fatigue	53.25	22.66	52.83	21.19	52.77	21.90
	Emotional well-being	69.75	18.82	70.55	19.05	69.96	18.87
	Social functioning	64.18	27.56	64.66	25.70	64.06	26.89
	Pain	72.93	25.15	69.47	26.36	70.94	25.77
	General health	68.11	19.11	65.33	18.69	66.53	18.96
	Health change	39.78	20.58	40.65	21.81	40.08	21.10

Time	Scale	Nicotinamide (n=248)		Placebo (n=252)		Total (n=500)	
		Mean	Standard deviation	Mean	Standard deviation	Mean	Standard deviation
Week 6	Physical functioning	90.67	15.24	90.74	17.32	90.50	16.28
	Role functioning/physical	69.07	40.06	67.52	40.20	67.77	40.19
	Role functioning/emotional	77.88	35.47	75.55	38.21	76.16	37.26
	Energy/fatigue	62.12	21.55	61.64	20.63	61.56	21.10
	Emotional well-being	75.42	17.54	75.36	17.89	75.21	17.68
	Social functioning	79.03	22.28	76.29	23.58	77.27	23.11
	Pain	86.30	20.21	81.23	23.64	83.31	22.36
	General health	70.36	19.57	69.42	18.86	69.86	19.08
	Health change	45.55	20.15	46.73	21.42	46.13	20.67

The higher the scores, the better the quality of life. The range for each of the eight SF-36 multi-item scales (except for health change) is 0 – 100. For details on scoring and reference ranges, see https://www.rand.org/health-care/surveys_tools/mos/36-item-short-form/scoring.html (last accessed on 11-Mar-2025).

Supplementary Table 5: Descriptive statistics of FACIT-F scales in the RFITT population.

Time	Scale	Nicotinamide (n=248)		Placebo (n=252)		Total (n=500)	
		Mean	Standard deviation	Mean	Standard deviation	Mean	Standard deviation
Week 0	Physical well-being	15.96	5.70	14.86	5.86	15.29	5.93
	Social/family well-being	22.35	4.47	21.97	4.56	22.30	4.51
	Emotional well-being	18.16	3.86	18.29	4.30	18.13	4.15
	Functional well-being	13.34	5.69	12.97	5.85	13.03	5.80
	Fatigue	23.33	12.30	22.59	11.98	22.99	12.21
	Trial Outcome Index	52.63	20.92	50.42	20.54	51.37	21.08
	FACT G Score	69.82	13.70	68.09	14.49	68.67	14.21
	FACIT-F General Score	93.15	24.01	90.68	24.08	91.57	24.35
Week 2	Physical well-being	22.69	5.35	22.03	5.29	22.29	5.40
	Social/family well-being	22.60	4.18	21.74	4.27	22.11	4.46
	Emotional well-being	20.32	3.46	20.03	3.62	19.99	3.62
	Functional well-being	17.20	5.97	17.28	5.91	17.05	6.17
	Fatigue	35.35	13.24	34.68	12.41	34.61	12.82
	Trial Outcome Index	75.24	23.20	73.98	21.72	74.43	22.71
	FACT G Score	82.80	14.54	81.07	15.29	81.63	15.39
	FACIT-F General Score	118.15	26.65	115.75	26.24	116.50	26.91
Week 4	Physical well-being	25.26	3.68	24.79	4.27	24.88	4.24
	Social/family well-being	22.83	4.80	22.39	4.52	22.56	4.78
	Emotional well-being	21.27	3.09	21.18	3.18	21.08	3.27
	Functional well-being	20.33	5.64	20.11	5.42	19.97	5.84
	Fatigue	41.47	10.05	41.32	9.96	41.08	10.42
	Trial Outcome Index	87.06	17.96	86.22	18.17	86.08	19.00
	FACT G Score	89.68	14.08	88.47	13.66	88.51	14.49
	FACIT-F General Score	131.16	22.87	129.79	22.32	129.69	23.56

Time	Scale	Nicotinamide		Placebo		Total	
		Mean	Standard deviation	Mean	Standard deviation	Mean	Standard deviation
Week 6	Physical well-being	26.16	2.98	26.11	3.46	26.02	3.31
	Social/family well-being	23.35	4.62	22.77	4.52	23.03	4.67
	Emotional well-being	21.75	2.68	21.70	3.17	21.61	2.95
	Functional well-being	21.67	5.36	22.06	5.24	21.71	5.37
	Fatigue	44.30	9.00	44.23	9.68	44.10	9.18
	Trial Outcome Index	92.13	15.97	92.40	17.15	92.02	16.38
	FACT G Score	92.92	12.92	92.64	13.52	92.60	13.28
	FACIT-F General Score	137.22	20.75	136.87	22.04	136.94	21.09

The higher the scores, the better the quality of life. The ranges of the FACIT-F scales are as follows:

Scale	Range
Physical well-being	0 – 28
Social/family well-being	0 – 28
Emotional well-being	0 – 24
Functional well-being	0 – 28
Fatigue	0 – 52
Trial Outcome Index	0 – 108
FACT G Score	0 – 108
FACIT-F General Score	0 – 160

For details on scoring and reference ranges, see https://www.facit.org/measures/FACIT-F (last accessed on 11-Mar-2025).

Supplementary Table 6: Effect of sex on key binary endpoints in the RFITT population.

	**Performance drop (primary endpoint)**		**Fatigue (3rd key secondary endpoint)**	
	**Odds ratio**	**P value**	**Odds ratio**	**P value**
Female vs. male	1.30	0.392	0.98	0.957
Treatment:Sex	0.96	0.827	1.26	0.311

Male: n=209; female: n=291. Generalized logistic regression model, Wald test, two-sided, unadjusted.

Supplementary Table 7: Effect of sex on key ordinal endpoints in the RFITT population.

	**Ability to perform normal activities (1st key secondary endpoint)**		**Cough (2nd key secondary endpoint)**	
	**β**	**P value**	**β**	**P value**
Female vs. male	0.11	0.338	-0.04	0.596
Treatment:Sex	-0.02	0.750	0.03	0.512

Male: n=209; female: n=291. Mixed model for repeated measures, Wald test, two-sided, unadjusted.

When extending existing models for primary and key secondary endpoints by sex and treatment-sex interaction terms, no significant effects can be observed. Supplementary Tables 6 and 7 show odds ratio or regression coefficient (β), respectively, and corresponding P values of sex-specific dependent factors.

Supplementary Table 8: Effects of nicotinamide on COVID-19 symptoms (change to baseline) in the entire RFITT population (binary symptoms).

Symptoms	Time	Nicotinamide	Placebo	Nicotinamide				Placebo				Difference			Odds Ratio			Woolfe test			Cochran-Mantel-Haenszel test			Two-sided Fisher exact test*		
		n total	n total	Resolution vs. baseline		Persisting symptom		Resolution vs. baseline		Persisting symptom		Diff.	95% CI		OR	95% CI		X^2	df	P	χ2MH	df	P	Week 2	Week 4	Week 6
				n	%	n	%	n	%	n	%		LCL	UCL		LCL	UCL							P	P	P
Performance drop	Week 2	191	188	110	57.59%	81	42.41%	80	42.55%	108	57.45%	15.04%	5.08%	24.99%	1.33	1.03	1.70	4.09	2	0.130	4.69	2	0.030	0.004	0.914	0.548
	Week 4	191	188	126	65.97%	65	34.03%	123	65.43%	65	34.57%	0.54%	-9.02%	10.10%												
	Week 6	191	188	148	77.49%	43	22.51%	140	74.47%	48	25.53%	3.02%	-5.58%	11.62%												
Fatigue	Week 2	199	198	105	52.76%	94	47.24%	96	48.48%	102	51.52%	4.28%	-5.55%	14.11%	1.05	0.82	1.35	1.24	2	0.538	0.10	2	0.750	0.423	0.737	0.532
	Week 4	199	198	146	73.37%	53	26.63%	142	71.72%	56	28.28%	1.65%	-7.13%	10.43%												
	Week 6	199	198	156	78.39%	43	21.61%	161	81.31%	37	18.69%	-2.92%	-10.81%	4.97%												
Fever	Week 2	25	37	25	100.00%	0	0.00%	37	100.00%	0	0.00%	n/a														
	Week 4	25	37	25	100.00%	0	0.00%	37	100.00%	0	0.00%	n/a														
	Week 6	25	37	25	100.00%	0	0.00%	37	100.00%	0	0.00%	n/a														
Chills	Week 2	24	29	24	100.00%	0	0.00%	29	100.00%	0	0.00%	n/a														
	Week 4	24	29	24	100.00%	0	0.00%	29	100.00%	0	0.00%	n/a														
	Week 6	24	29	24	100.00%	0	0.00%	29	100.00%	0	0.00%	n/a														
Shortness of breath	Week 2	92	90	56	60.87%	36	39.13%	37	41.11%	53	58.89%	19.76%	5.52%	34.00%	1.20	0.84	1.70	6.73	2	0.035	0.85	2	0.358	0.012	0.759	0.506
	Week 4	92	90	58	63.04%	34	36.96%	59	65.56%	31	34.44%	-2.51%	-16.43%	11.40%												
	Week 6	92	90	65	70.65%	27	29.35%	68	75.56%	22	24.44%	-4.90%	-17.76%	7.96%												
Whistling / Wheezing breathing	Week 2	28	34	25	89.29%	3	10.71%	32	94.12%	2	5.88%	-4.83%	-18.75%	9.09%	1.10	0.24	5.16	1.08	2	0.582	0.02	2	0.900	0.650	>0.999	>0.999
	Week 4	28	34	28	100.00%	0	0.00%	33	97.06%	1	2.94%	2.94%	-2.74%	8.62%												
	Week 6	28	34	28	100.00%	0	0.00%	33	97.06%	1	2.94%	2.94%	-2.74%	8.62%												
Cough	Week 2	116	119	84	72.41%	32	27.59%	80	67.23%	39	32.77%	5.19%	-6.53%	16.90%	1.01	0.68	1.50	2.73	2	0.255	0.00	2	0.966	0.398	0.212	0.797
	Week 4	116	119	94	81.03%	22	18.97%	104	87.39%	15	12.61%	-6.36%	-15.66%	2.94%												
	Week 6	116	119	109	93.97%	7	6.03%	110	92.44%	9	7.56%	1.53%	-4.90%	7.96%												
Cough with sputum production	Week 2	93	95	72	77.42%	21	22.58%	74	77.89%	21	22.11%	-0.48%	-12.39%	11.43%	0.71	0.42	1.20	2.29	2	0.318	1.33	2	0.250	>0.999	0.061	0.719
	Week 4	93	95	79	84.95%	14	15.05%	89	93.68%	6	6.32%	-8.74%	-17.50%	0.02%												
	Week 6	93	95	89	95.70%	4	4.30%	92	96.84%	3	3.16%	-1.14%	-6.56%	4.28%												

Symptoms	Time	Nicotinamide	Placebo	Nicotinamide				Placebo				Difference			Odds Ratio			Woolfe test			Cochran-Mantel-Haenszel test			Two-sided Fisher exact test*		
		n total	n total	Resolution vs. baseline		Persisting symptom		Resolution vs. baseline		Persisting symptom		Diff.	95% CI		OR	95% CI		X^2	df	P	χ2MH	df	P	Week 2	Week 4	Week 6
				n	%	n	%	n	%	n	%		LCL	UCL		LCL	UCL							P	P	P
Rhinitis/rhinorrhea	Week 2	184	178	143	77.72%	41	22.28%	126	70.79%	52	29.21%	6.93%	-2.06%	15.92%	1.54	1.08	2.19	0.23	2	0.893	5.24	2	0.022	0.149	0.096	0.569
	Week 4	184	178	164	89.13%	20	10.87%	147	82.58%	31	17.42%	6.55%	-0.61%	13.71%												
	Week 6	184	178	179	97.28%	5	2.72%	171	96.07%	7	3.93%	1.22%	-2.48%	4.91%												
Sore throat	Week 2	78	93	72	92.31%	6	7.69%	81	87.10%	12	12.90%	5.21%	-3.81%	14.23%	1.29	0.63	2.61	0.77	2	0.680	0.27	2	0.603	0.323	>0.999	>0.999
	Week 4	78	93	71	91.03%	7	8.97%	85	91.40%	8	8.60%	-0.37%	-8.90%	8.15%												
	Week 6	78	93	77	98.72%	1	1.28%	92	98.92%	1	1.08%	-0.21%	-3.47%	3.05%												
Hoarseness	Week 2	76	92	67	88.16%	9	11.84%	86	93.48%	6	6.52%	-5.32%	-14.16%	3.52%	0.89	0.38	2.08	3.86	2	0.145	0.00	2	0.967	0.281	0.129	0.452
	Week 4	76	92	75	98.68%	1	1.32%	86	93.48%	6	6.52%	5.21%	-0.45%	10.86%												
	Week 6	76	92	75	98.68%	1	1.32%	92	100.00%	0	0.00%	-1.32%	-3.88%	1.25%												
Pneumonia	Week 2	0	0	0	0.00%	0	0.00%	0	0.00%	0	0.00%	n/a														
	Week 4	0	0	0	0.00%	0	0.00%	0	0.00%	0	0.00%	n/a														
	Week 6	0	0	0	0.00%	0	0.00%	0	0.00%	0	0.00%	n/a														
Muscle pain	Week 2	79	83	77	97.47%	2	2.53%	81	97.59%	2	2.41%	-0.12%	-4.91%	4.66%	0.95	0.33	2.76	0.00	2	>0.999	0.01	2	0.925	>0.999	>0.999	>0.999
	Week 4	79	83	75	94.94%	4	5.06%	79	95.18%	4	4.82%	-0.24%	-6.92%	6.43%												
	Week 6	79	83	78	98.73%	1	1.27%	82	98.80%	1	1.20%	-0.06%	-3.46%	3.34%												
Joint pain	Week 2	57	55	53	92.98%	4	7.02%	51	92.73%	4	7.27%	0.26%	-9.29%	9.80%	0.45	0.15	1.34	2.05	2	0.358	1.46	2	0.228	>0.999	0.618	0.119
	Week 4	57	55	54	94.74%	3	5.26%	54	98.18%	1	1.82%	-3.44%	-10.23%	3.34%												
	Week 6	57	55	53	92.98%	4	7.02%	55	100.00%	0	0.00%	-7.02%	-13.65%	-0.39%												
Limb pain	Week 2	71	87	66	92.96%	5	7.04%	84	96.55%	3	3.45%	-3.59%	-10.67%	3.49%	0.81	0.23	2.86	1.39	2	0.499	0.00	2	0.997	0.469	>0.999	>0.999
	Week 4	71	87	71	100.00%	0	0.00%	86	98.85%	1	1.15%	1.15%	-1.09%	3.39%												
	Week 6	71	87	71	100.00%	0	0.00%	86	98.85%	1	1.15%	1.15%	-1.09%	3.39%												
Chest pain	Week 2	57	48	52	91.23%	5	8.77%	45	93.75%	3	6.25%	-2.52%	-12.56%	7.52%	0.58	0.17	1.98	0.72	2	0.697	0.34	2	0.558	0.724	0.499	>0.999
	Week 4	57	48	55	96.49%	2	3.51%	48	100.00%	0	0.00%	-3.51%	-8.29%	1.27%												
	Week 6	57	48	56	98.25%	1	1.75%	47	97.92%	1	2.08%	0.33%	-4.96%	5.62%												
Headache	Week 2	113	115	90	79.65%	23	20.35%	85	73.91%	30	26.09%	5.73%	-5.20%	16.67%	1.44	0.91	2.30	0.08	2	0.963	2.05	2	0.152	0.348	0.380	0.768
	Week 4	113	115	104	92.04%	9	7.96%	101	87.83%	14	12.17%	4.21%	-3.58%	12.00%												
	Week 6	113	115	108	95.58%	5	4.42%	108	93.91%	7	6.09%	1.66%	-4.12%	7.45%												

Symptoms	Time	Nicotinamide	Placebo	Nicotinamide				Placebo				Difference			Odds Ratio			Woolfe test			Cochran-Mantel-Haenszel test			Two-sided Fisher exact test*		
		n total	n total	Resolution vs. baseline		Persisting symptom		Resolution vs. baseline		Persisting symptom		Diff.	95% CI		OR	95% CI		X^2	df	P	χ2MH	df	P	Week 2	Week 4	Week 6
				n	%	n	%	n	%	n	%		LCL	UCL		LCL	UCL							P	P	P
Abdominal pain	Week 2	19	21	18	94.74%	1	5.26%	19	90.48%	2	9.52%	4.26%	-11.82%	20.34%	1.38	0.22	8.76	0.14	2	0.932	0.12	2	0.732	>0.999	>0.999	>0.999
	Week 4	19	21	19	100.00%	0	0.00%	21	100.00%	0	0.00%	0.00%	0.00%	0.00%												
	Week 6	19	21	18	94.74%	1	5.26%	20	95.24%	1	4.76%	-0.50%	-14.06%	13.06%												
Diarrhea	Week 2	28	51	26	92.86%	2	7.14%	45	88.24%	6	11.76%	4.62%	-8.39%	17.63%	1.40	0.42	4.65	0.14	2	0.934	0.07	2	0.790	0.705	>0.999	>0.999
	Week 4	28	51	27	96.43%	1	3.57%	49	96.08%	2	3.92%	0.35%	-8.35%	9.05%												
	Week 6	28	51	27	96.43%	1	3.57%	49	96.08%	2	3.92%	0.35%	-8.35%	9.05%												
Nausea	Week 2	24	34	21	87.50%	3	12.50%	33	97.06%	1	2.94%	-9.56%	-23.96%	4.84%	0.34	0.06	1.90	1.31	2	0.519	0.737	2	0.391	0.30	>0.999	0.414
	Week 4	24	34	24	100.00%	0	0.00%	33	97.06%	1	2.94%	2.94%	-2.74%	8.62%												
	Week 6	24	34	23	95.83%	1	4.17%	34	100.00%	0	0.00%	-4.17%	-12.16%	3.83%												
Vomiting	Week 2	1	3	1	100.00%	0	0.00%	3	100.00%	0	0.00%	0.00%	0.00%	0.00%	n/a											
	Week 4	1	3	1	100.00%	0	0.00%	3	100.00%	0	0.00%	0.00%	0.00%	0.00%												
	Week 6	1	3	1	100.00%	0	0.00%	3	100.00%	0	0.00%	0.00%	0.00%	0.00%												
Loss of appetite / lower food intake	Week 2	125	126	118	94.40%	7	5.60%	116	92.06%	10	7.94%	2.34%	-3.87%	8.54%	1.48	0.72	3.05	0.00	2	0.999	0.78	2	0.377	0.617	0.749	>0.999
	Week 4	125	126	121	96.80%	4	3.20%	120	95.24%	6	4.76%	1.56%	-3.27%	6.39%												
	Week 6	125	126	123	98.40%	2	1.60%	123	97.62%	3	2.38%	0.78%	-2.67%	4.23%												
Other gastrointes-tinal symptoms	Week 2	11	7	9	81.82%	2	18.18%	6	85.71%	1	14.29%	-3.90%	-38.41%	30.62%	2.58	0.38	17.50	1.17	2	0.558	0.28	2	0.597	>0.999	0.389	0.389
	Week 4	11	7	11	100.00%	0	0.00%	6	85.71%	1	14.29%	14.29%	-11.64%	40.21%												
	Week 6	11	7	11	100.00%	0	0.00%	6	85.71%	1	14.29%	14.29%	-11.64%	40.21%												
Impaired sense of smell	Week 2	114	137	61	53.51%	53	46.49%	67	48.91%	70	51.09%	4.60%	-7.80%	17.01%	1.19	0.88	1.63	1.37	2	0.505	1.08	2	0.299	0.526	0.892	0.179
	Week 4	114	137	78	68.42%	36	31.58%	95	69.34%	42	30.66%	-0.92%	-12.43%	10.59%												
	Week 6	114	137	92	80.70%	22	19.30%	100	72.99%	37	27.01%	7.71%	-2.67%	18.09%												
Impaired sense of taste	Week 2	103	137	56	54.37%	47	45.63%	76	55.47%	61	44.53%	-1.11%	-13.83%	11.61%	1.11	0.81	1.54	0.95	2	0.623	0.33	2	0.567	0.896	0.776	0.281
	Week 4	103	137	74	71.84%	29	28.16%	96	70.07%	41	29.93%	1.77%	-9.81%	13.36%												
	Week 6	103	137	83	80.58%	20	19.42%	102	74.45%	35	25.55%	6.13%	-4.44%	16.70%												
Confusion	Week 2	15	16	14	93.33%	1	6.67%	15	93.75%	1	6.25%	-0.42%	-17.74%	16.90%	1.93	0.17	22.63	0.35	2	0.840	0.28	2	0.598	>0.999	>0.999	>0.999
	Week 4	15	16	15	100.00%	0	0.00%	15	93.75%	1	6.25%	6.25%	-5.61%	18.11%												
	Week 6	15	16	15	100.00%	0	0.00%	16	100.00%	0	0.00%	0.00%	0.00%	0.00%												

Symptoms	Time	Nicotinamide	Placebo	Nicotinamide				Placebo				Difference			Odds Ratio			Woolfe test			Cochran-Mantel-Haenszel test			Two-sided Fisher exact test*		
		n total	n total	Resolution vs. baseline		Persisting symptom		Resolution vs. baseline		Persisting symptom		Diff.	95% CI		OR	95% CI		X^2	df	P	χ2MH	df	P	Week 2	Week 4	Week 6
				n	%	n	%	n	%	n	%		LCL	UCL		LCL	UCL							P	P	P
Dizziness	Week 2	50	49	47	94.00%	3	6.00%	40	81.63%	9	18.37%	12.37%	-0.32%	25.05%	1.52	0.63	3.67	3.21	2	0.201	0.51	2	0.474	0.071	1.000	0.678
	Week 4	50	49	48	96.00%	2	4.00%	47	95.92%	2	4.08%	0.08%	-7.68%	7.84%												
	Week 6	50	49	46	92.00%	4	8.00%	47	95.92%	2	4.08%	-3.92%	-13.26%	5.42%												
Conjunctivitis	Week 2	3	9	2	66.67%	1	33.33%	9	100.00%	0	0.00%	-33.33%	-86.68%	20.01%	0.33	0.02	5.33	1.12	2	0.571	0.00	2	>0.999	0.250	>0.999	>0.999
	Week 4	3	9	3	100.00%	0	0.00%	8	88.89%	1	11.11%	11.11%	-9.42%	31.64%												
	Week 6	3	9	3	100.00%	0	0.00%	9	100.00%	0	0.00%	0.00%	0.00%	0.00%												
Skin rash	Week 2	17	11	15	88.24%	2	11.76%	11	100.00%	0	0.00%	-11.76%	-27.08%	3.55%	n/a											
	Week 4	17	11	16	94.12%	1	5.88%	11	100.00%	0	0.00%	-5.88%	-17.07%	5.30%												
	Week 6	17	11	16	94.12%	1	5.88%	11	100.00%	0	0.00%	-5.88%	-17.07%	5.30%												
Hair loss	Week 2	7	3	6	85.71%	1	14.29%	3	100.00%	0	0.00%	-14.29%	-40.21%	11.64%	n/a											
	Week 4	7	3	6	85.71%	1	14.29%	3	100.00%	0	0.00%	-14.29%	-40.21%	11.64%												
	Week 6	7	3	7	100.00%	0	0.00%	3	100.00%	0	0.00%	0.00%	0.00%	0.00%												
Other symptoms	Week 2	78	88	57	73.08%	21	26.92%	69	78.41%	19	21.59%	-5.33%	-18.40%	7.74%	0.68	0.43	1.07	0.33	2	0.849	2.46	2	0.116	0.470	0.207	0.535
	Week 4	78	88	62	79.49%	16	20.51%	77	87.50%	11	12.50%	-8.01%	-19.33%	3.30%												
	Week 6	78	88	63	80.77%	15	19.23%	75	85.23%	13	14.77%	-4.46%	-15.92%	7.01%												

CI, confidence interval; LCL, lower confidence limit; n, number of patients with non-missing values used for the respective analysis; n/a, not applicable; OR, odds ratio; UCL, upper confidence limit.

* Adjusted P values (see section 6).

Supplementary Table 9: Effects of nicotinamide on COVID-19 symptoms (change to baseline) in males of the RFITT population (binary symptoms).

Symptoms	Time	Nicotinamide	Placebo	Nicotinamide				Placebo				Difference			Odds Ratio			Cochran-Mantel-Haenszel test			Two-sided Fisher exact test*		
		n total	n total	Resolution vs. baseline		Persisting symptom		Resolution vs. baseline		Persisting symptom		Diff.	95% CI		OR	95% CI		χ2MH	df	P	Week 2	Week 4	Week 6
				n	%	n	%	n	%	n	%		LCL	UCL		LCL	UCL				P	P	P
Performance drop	Week 2	73	77	48	65.75%	25	34.25%	34	44.16%	43	55.84%	21.60%	6.06%	37.14%	2.43	1.25	4.70	4.30	2	0.038	0.009	0.861	0.232
	Week 4	73	77	50	68.49%	23	31.51%	54	70.13%	23	29.87%	-1.64%	-16.40%	13.13%	0.93	0.46	1.85						
	Week 6	73	77	61	83.56%	12	16.44%	58	75.32%	19	24.68%	8.24%	-4.61%	21.08%	1.67	0.74	3.73						
Fatigue	Week 2	82	78	48	58.54%	34	41.46%	45	57.69%	33	42.31%	0.84%	-14.45%	16.14%	1.04	0.55	1.94	0.00	2	0.989	1.000	1.000	1.000
	Week 4	82	78	65	79.27%	17	20.73%	62	79.49%	16	20.51%	-0.22%	-12.76%	12.32%	0.99	0.46	2.12						
	Week 6	82	78	69	84.15%	13	15.85%	66	84.62%	12	15.38%	-0.47%	-11.72%	10.78%	0.97	0.41	2.27						
Fever	Week 2	15	16	15	100.00%	0	0.00%	16	100.00%	0	0.00%	n/a											
	Week 4	15	16	15	100.00%	0	0.00%	16	100.00%	0	0.00%	n/a											
	Week 6	15	16	15	100.00%	0	0.00%	16	100.00%	0	0.00%	n/a											
Chills	Week 2	12	7	12	100.00%	0	0.00%	7	100.00%	0	0.00%	n/a											
	Week 4	12	7	12	100.00%	0	0.00%	7	100.00%	0	0.00%	n/a											
	Week 6	12	7	12	100.00%	0	0.00%	7	100.00%	0	0.00%	n/a											
Shortness of breath	Week 2	36	28	24	66.67%	12	33.33%	14	50.00%	14	50.00%	16.67%	-7.42%	40.75%	2.00	0.73	5.52	0.16	2	0.693	0.207	0.788	1.000
	Week 4	36	28	24	66.67%	12	33.33%	20	71.43%	8	28.57%	-4.76%	-27.50%	17.98%	0.80	0.27	2.34						
	Week 6	36	28	28	77.78%	8	22.22%	22	78.57%	6	21.43%	-0.79%	-21.18%	19.59%	0.95	0.29	3.16						
Whistling / Wheezing breathing	Week 2	16	14	15	93.75%	1	6.25%	13	92.86%	1	7.14%	0.89%	-17.07%	18.86%	1.15	0.07	20.34	0.41	2	0.522	1.000	0.467	0.467
	Week 4	16	14	16	100.00%	0	0.00%	13	92.86%	1	7.14%	7.14%	-6.35%	20.63%	2.46	0.08	79.33						
	Week 6	16	14	16	100.00%	0	0.00%	13	92.86%	1	7.14%	7.14%	-6.35%	20.63%	2.46	0.08	79.33						
Cough	Week 2	40	47	29	72.50%	11	27.50%	35	74.47%	12	25.53%	-1.97%	-20.59%	16.66%	0.90	0.35	2.35	0.01	2	0.928	1.000	0.562	0.721
	Week 4	40	47	33	82.50%	7	17.50%	41	87.23%	6	12.77%	-4.73%	-19.89%	10.42%	0.69	0.21	2.25						
	Week 6	40	47	37	92.50%	3	7.50%	42	89.36%	5	10.64%	3.14%	-8.88%	15.15%	1.47	0.33	6.57						
Cough with sputum production	Week 2	40	44	29	72.50%	11	27.50%	37	84.09%	7	15.91%	-11.59%	-29.15%	5.97%	0.50	0.17	1.45	2.47	2	0.11	0.287	0.418	0.476
	Week 4	40	44	36	90.00%	4	10.00%	42	95.45%	2	4.55%	-5.45%	-16.60%	5.70%	0.43	0.07	2.48						
	Week 6	40	44	39	97.50%	1	2.50%	44	100.00%	0	0.00%	-2.50%	-7.34%	2.34%	0.44	0.01	13.58						

Symptoms	Time	Nicotinamide	Placebo	Nicotinamide				Placebo				Difference			Odds Ratio			Cochran-Mantel-Haenszel test			Two-sided Fisher exact test*		
		n total	n total	Resolution vs. baseline		Persisting symptom		Resolution vs. baseline		Persisting symptom		Diff.	95% CI		OR	95% CI		χ2MH	df	P	Week 2	Week 4	Week 6
				n	%	n	%	n	%	n	%		LCL	UCL		LCL	UCL				P	P	P
Rhinitis/rhinorrhea	Week 2	73	78	57	78.08%	16	21.92%	58	74.36%	20	25.64%	3.72%	-9.84%	17.29%	1.23	0.58	2.61	2.18	2	0.139	0.703	0.189	0.368
	Week 4	73	78	68	93.15%	5	6.85%	67	85.90%	11	14.10%	7.25%	-2.40%	16.91%	2.23	0.74	6.77						
	Week 6	73	78	72	98.63%	1	1.37%	74	94.87%	4	5.13%	3.76%	-1.82%	9.33%	3.89	0.42	35.66						
Sore throat	Week 2	35	39	32	91.43%	3	8.57%	36	92.31%	3	7.69%	-0.88%	-13.37%	11.61%	0.89	0.17	4.72	0.04	2	0.847	1.000	0.662	1.000
	Week 4	35	39	32	91.43%	3	8.57%	37	94.87%	2	5.13%	-3.44%	-15.02%	8.13%	0.58	0.09	3.67						
	Week 6	35	39	35	100.00%	0	0.00%	38	97.44%	1	2.56%	2.56%	-2.40%	7.52%	1.84	0.06	56.64						
Hoarseness	Week 2	31	31	29	93.55%	2	6.45%	31	100.00%	0	0.00%	-6.45%	-15.10%	2.20%	0.23	0.01	5.40	0.25	2	0.616	0.492	1.000	1.000
	Week 4	31	31	31	100.00%	0	0.00%	30	96.77%	1	3.23%	3.23%	-2.99%	9.45%	2.07	0.07	63.92						
	Week 6	31	31	30	96.77%	1	3.23%	31	100.00%	0	0.00%	-3.23%	-9.45%	2.99%	0.48	0.02	14.97						
Pneumonia	Week 2	0	0	0	0.00%	0	0.00%	0	0.00%	0	0.00%	n/a											
	Week 4	0	0	0	0.00%	0	0.00%	0	0.00%	0	0.00%	n/a											
	Week 6	0	0	0	0.00%	0	0.00%	0	0.00%	0	0.00%	n/a											
Muscle pain	Week 2	37	30	37	100.00%	0	0.00%	30	100.00%	0	0.00%	n/a											
	Week 4	37	30	37	100.00%	0	0.00%	29	96.67%	1	3.33%	3.33%	-3.09%	9.76%	2.55	0.08	78.74						
	Week 6	37	30	37	100.00%	0	0.00%	30	100.00%	0	0.00%	n/a											
Joint pain	Week 2	24	28	23	95.83%	1	4.17%	27	96.43%	1	3.57%	-0.60%	-11.14%	9.95%	0.85	0.05	14.39	0.02	2	0.893	1.000	1.000	0.462
	Week 4	24	28	24	100.00%	0	0.00%	28	100.00%	0	0.00%	0.00%	0.00%	0.00%	n/a								
	Week 6	24	28	23	95.83%	1	4.17%	28	100.00%	0	0.00%	-4.17%	-12.16%	3.83%	0.41	0.01	12.80						
Limb pain	Week 2	32	35	29	90.63%	3	9.38%	35	100.00%	0	0.00%	-9.38%	-19.47%	0.72%	0.14	0.01	2.87	1.57	2	0.210	0.104	1.000	1.000
	Week 4	32	35	32	100.00%	0	0.00%	35	100.00%	0	0.00%	n/a											
	Week 6	32	35	32	100.00%	0	0.00%	35	100.00%	0	0.00%	n/a											
Chest pain	Week 2	28	16	25	89.29%	3	10.71%	16	100.00%	0	0.00%	-10.71%	-22.17%	0.74%	0.26	0.01	5.55	0.70	2	0.404	0.290	0.526	1.000
	Week 4	28	16	26	92.86%	2	7.14%	16	100.00%	0	0.00%	-7.14%	-16.68%	2.40%	0.41	0.02	9.59						
	Week 6	28	16	27	96.43%	1	3.57%	15	93.75%	1	6.25%	2.68%	-11.03%	16.39%	1.80	0.10	30.90						
Headache	Week 2	47	43	36	76.60%	11	23.40%	35	81.40%	8	18.60%	-4.80%	-21.59%	11.99%	0.75	0.27	2.08	0.00	2	0.947	0.615	0.705	0.345
	Week 4	47	43	44	93.62%	3	6.38%	39	90.70%	4	9.30%	2.92%	-8.23%	14.06%	1.50	0.32	7.14						
	Week 6	47	43	46	97.87%	1	2.13%	40	93.02%	3	6.98%	4.85%	-3.81%	13.51%	3.45	0.35	34.50						

Symptoms	Time	Nicotinamide	Placebo	Nicotinamide				Placebo				Difference			Odds Ratio			Cochran-Mantel-Haenszel test			Two-sided Fisher exact test*		
		n total	n total	Resolution vs. baseline		Persisting symptom		Resolution vs. baseline		Persisting symptom		Diff.	95% CI		OR	95% CI		χ2MH	df	P	Week 2	Week 4	Week 6
				n	%	n	%	n	%	n	%		LCL	UCL		LCL	UCL				P	P	P
Abdominal pain	Week 2	9	6	9	100.00%	0	0.00%	6	100.00%	0	0.00%	0.00%	0.00%	0.00%	n/a								
	Week 4	9	6	9	100.00%	0	0.00%	6	100.00%	0	0.00%	0.00%	0.00%	0.00%	n/a								
	Week 6	9	6	9	100.00%	0	0.00%	6	100.00%	0	0.00%	0.00%	0.00%	0.00%	n/a								
Diarrhea	Week 2	10	20	10	100.00%	0	0.00%	18	90.00%	2	10.00%	10.00%	-3.15%	23.15%	2.22	0.09	54.19	0.06	2	0.799	0.540	1.000	1.000
	Week 4	10	20	10	100.00%	0	0.00%	20	100.00%	0	0.00%	n/a											
	Week 6	10	20	10	100.00%	0	0.00%	20	100.00%	0	0.00%	n/a											
Nausea	Week 2	8	8	8	100.00%	0	0.00%	8	100.00%	0	0.00%	n/a											
	Week 4	8	8	8	100.00%	0	0.00%	8	100.00%	0	0.00%	n/a											
	Week 6	8	8	8	100.00%	0	0.00%	8	100.00%	0	0.00%	n/a											
Vomiting	Week 2	0	1	0	0.00%	0	0.00%	1	100.00%	0	0.00%	n/a											
	Week 4	0	1	0	0.00%	0	0.00%	1	100.00%	0	0.00%	n/a											
	Week 6	0	1	0	0.00%	0	0.00%	1	100.00%	0	0.00%	n/a											
Loss of appetite / lower food intake	Week 2	47	45	45	95.74%	2	4.26%	43	95.56%	2	4.44%	0.19%	-8.15%	8.53%	1.05	0.14	7.76	0.07	2	0.786	1.000	1.000	1.000
	Week 4	47	45	45	95.74%	2	4.26%	44	97.78%	1	2.22%	-2.03%	-9.23%	5.17%	0.51	0.04	5.84						
	Week 6	47	45	46	97.87%	1	2.13%	44	97.78%	1	2.22%	0.09%	-5.87%	6.06%	1.05	0.06	17.24						
Other gastrointes-tinal symptoms	Week 2	5	2	4	80.00%	1	20.00%	2	100.00%	0	0.00%	-20.00%	-55.06%	15.06%	1.00	0.02	44.50	0.40	2	0.527	1.000	1.000	1.000
	Week 4	5	2	5	100.00%	0	0.00%	2	100.00%	0	0.00%	n/a											
	Week 6	5	2	5	100.00%	0	0.00%	2	100.00%	0	0.00%	n/a											
Impaired sense of smell	Week 2	45	57	21	46.67%	24	53.33%	27	47.37%	30	52.63%	-0.70%	-20.21%	18.80%	0.97	0.44	2.13	0.16	2	0.687	1.000	0.394	1.000
	Week 4	45	57	28	62.22%	17	37.78%	41	71.93%	16	28.07%	-9.71%	-28.06%	8.64%	0.64	0.28	1.48						
	Week 6	45	57	34	75.56%	11	24.44%	42	73.68%	15	26.32%	1.87%	-15.11%	18.85%	1.10	0.45	2.71						
Impaired sense of taste	Week 2	44	56	23	52.27%	21	47.73%	33	58.93%	23	41.07%	-6.66%	-26.25%	12.94%	0.76	0.34	1.69	0.27	2	0.604	0.547	0.505	0.803
	Week 4	44	56	30	68.18%	14	31.82%	42	75.00%	14	25.00%	-6.82%	-24.65%	11.02%	0.71	0.30	1.72						
	Week 6	44	56	36	81.82%	8	18.18%	44	78.57%	12	21.43%	3.25%	-12.42%	18.91%	1.23	0.45	3.33						
Confusion	Week 2	7	8	6	85.71%	1	14.29%	8	100.00%	0	0.00%	-14.29%	-40.21%	11.64%	0.38	0.01	13.13	0.01	2	0.925	0.467	1.000	1.000
	Week 4	7	8	7	100.00%	0	0.00%	7	87.50%	1	12.50%	12.50%	-10.42%	35.42%	2.00	0.06	69.82						
	Week 6	7	8	7	100.00%	0	0.00%	8	100.00%	0	0.00%	n/a											

Symptoms	Time	Nicotinamide	Placebo	Nicotinamide				Placebo				Difference			Odds Ratio			Cochran-Mantel-Haenszel test			Two-sided Fisher exact test*		
		n total	n total	Resolution vs. baseline		Persisting symptom		Resolution vs. baseline		Persisting symptom		Diff.	95% CI		OR	95% CI		χ2MH	df	P	Week 2	Week 4	Week 6
				n	%	n	%	n	%	n	%		LCL	UCL		LCL	UCL				P	P	P
Dizziness	Week 2	17	9	17	100.00%	0	0.00%	7	77.78%	2	22.22%	22.22%	-4.94%	49.38%	9.71	0.39	243.52	0.78	2	0.378	0.111	1.000	1.000
	Week 4	17	9	16	94.12%	1	5.88%	8	88.89%	1	11.11%	5.23%	-18.15%	28.61%	2.00	0.11	36.31						
	Week 6	17	9	15	88.24%	2	11.76%	8	88.89%	1	11.11%	-0.65%	-26.27%	24.96%	0.94	0.07	12.00						
Conjunctivitis	Week 2	1	4	1	100.00%	0	0.00%	4	100.00%	0	0.00%	n/a											
	Week 4	1	4	1	100.00%	0	0.00%	4	100.00%	0	0.00%	n/a											
	Week 6	1	4	1	100.00%	0	0.00%	4	100.00%	0	0.00%	n/a											
Skin rash	Week 2	7	3	6	85.71%	1	14.29%	3	100.00%	0	0.00%	-14.29%	-40.21%	11.64%	1.00	0.03	39.13	0.02	2	0.8774	1.0000	1.0000	1.0000
	Week 4	7	3	6	85.71%	1	14.29%	3	100.00%	0	0.00%	-14.29%	-40.21%	11.64%	1.00	0.03	39.13						
	Week 6	7	3	7	100.00%	0	0.00%	3	100.00%	0	0.00%	n/a											
Hair loss	Week 2	1	0	1	100.00%	0	0.00%	0	0.00%	0	0.00%	n/a											
	Week 4	1	0	1	100.00%	0	0.00%	0	0.00%	0	0.00%	n/a											
	Week 6	1	0	1	100.00%	0	0.00%	0	0.00%	0	0.00%	n/a											
Other symptoms	Week 2	34	36	29	85.29%	5	14.71%	29	80.56%	7	19.44%	4.74%	-12.84%	22.31%	1.40	0.40	4.92	0.09	2	0.766	0.754	0.669	0.674
	Week 4	34	36	31	91.18%	3	8.82%	34	94.44%	2	5.56%	-3.27%	-15.39%	8.85%	0.61	0.10	3.88						
	Week 6	34	36	32	94.12%	2	5.88%	32	88.89%	4	11.11%	5.23%	-7.73%	18.19%	2.00	0.34	11.70						

CI, confidence interval; LCL, lower confidence limit; n, number of patients with non-missing values used for the respective analysis; n/a, not applicable; OR, odds ratio; UCL, upper confidence limit.

* Adjusted P values (see section 6).

Supplementary Table 10: Effects of nicotinamide on COVID-19 symptoms (change to baseline) in females of the RFITT population (binary symptoms).

Symptoms	Time	Nicotinamide	Placebo	Nicotinamide				Placebo				Difference			Odds Ratio			Cochran-Mantel-Haenszel test			Two-sided Fisher exact test*		
		n total	n total	Resolution vs. baseline		Persisting symptom		Resolution vs. baseline		Persisting symptom		Diff.	95% CI		OR	95% CI		χ2MH	df	P	Week2	Week4	Week6
				n	%	n	%	n	%	n	%		LCL	UCL		LCL	UCL				P	P	P
Performance drop	Week 2	118	111	62	52.54%	56	47.46%	46	41.44%	65	58.56%	11.10%	-1.75%	23.95%	1.56	0.93	2.64	1.28	2	0.257	0.112	0.784	1.000
	Week 4	118	111	76	64.41%	42	35.59%	69	62.16%	42	37.84%	2.24%	-10.25%	14.74%	1.10	0.64	1.89						
	Week 6	118	111	87	73.73%	31	26.27%	82	73.87%	29	26.13%	-0.15%	-11.54%	11.25%	0.99	0.55	1.79						
Fatigue	Week 2	117	120	57	48.72%	60	51.28%	51	42.50%	69	57.50%	6.22%	-6.44%	18.88%	1.29	0.77	2.15	0.09	2	0.764	0.363	0.679	0.442
	Week 4	117	120	81	69.23%	36	30.77%	80	66.67%	40	33.33%	2.56%	-9.31%	14.44%	1.13	0.65	1.94						
	Week 6	117	120	87	74.36%	30	25.64%	95	79.17%	25	20.83%	-4.81%	-15.55%	5.93%	0.76	0.42	1.40						
Fever	Week 2	10	21	10	100.00%	0	0.00%	21	100.00%	0	0.00%	n/a											
	Week 4	10	21	10	100.00%	0	0.00%	21	100.00%	0	0.00%	n/a											
	Week 6	10	21	10	100.00%	0	0.00%	21	100.00%	0	0.00%	n/a											
Chills	Week 2	12	22	12	100.00%	0	0.00%	22	100.00%	0	0.00%	n/a											
	Week 4	12	22	12	100.00%	0	0.00%	22	100.00%	0	0.00%	n/a											
	Week 6	12	22	12	100.00%	0	0.00%	22	100.00%	0	0.00%	n/a											
Shortness of breath	Week 2	56	62	32	57.14%	24	42.86%	23	37.10%	39	62.90%	20.05%	2.37%	37.73%	2.26	1.08	4.73	0.27	2	0.602	0.042	0.851	0.420
	Week 4	56	62	34	60.71%	22	39.29%	39	62.90%	23	37.10%	-2.19%	-19.74%	15.37%	0.91	0.43	1.92						
	Week 6	56	62	37	66.07%	19	33.93%	46	74.19%	16	25.81%	-8.12%	-24.63%	8.38%	0.68	0.31	1.50						
Whistling / Wheezing breathing	Week 2	12	20	10	83.33%	2	16.67%	19	95.00%	1	5.00%	-11.67%	-34.82%	11.48%	0.26	0.02	3.27	0.21	2	0.644	0.540	1.000	1.000
	Week 4	12	20	12	100.00%	0	0.00%	20	100.00%	0	0.00%	n/a											
	Week 6	12	20	12	100.00%	0	0.00%	20	100.00%	0	0.00%	n/a											
Cough	Week 2	76	72	55	72.37%	21	27.63%	45	62.50%	27	37.50%	9.87%	-5.17%	24.91%	1.57	0.79	3.14	0.02	2	0.881	0.222	0.270	1.000
	Week 4	76	72	61	80.26%	15	19.74%	63	87.50%	9	12.50%	-7.24%	-19.00%	4.53%	0.58	0.24	1.43						
	Week 6	76	72	72	94.74%	4	5.26%	68	94.44%	4	5.56%	0.29%	-7.00%	7.59%	1.06	0.25	4.40						
Cough with sputum production	Week 2	53	51	43	81.13%	10	18.87%	37	72.55%	14	27.45%	8.58%	-7.57%	24.74%	1.63	0.65	4.09	0.00	2	0.980	0.356	0.150	1.000
	Week 4	53	51	43	81.13%	10	18.87%	47	92.16%	4	7.84%	-11.02%	-23.89%	1.84%	0.37	0.11	1.25						
	Week 6	53	51	50	94.34%	3	5.66%	48	94.12%	3	5.88%	0.22%	-8.75%	9.19%	1.04	0.20	5.42						

**Symptoms**	**Time**	**Nicotinamide**	**Placebo**	**Nicotinamide**				**Placebo**				**Difference**			**Odds Ratio**			**Cochran-Mantel-Haenszel test**			**Two-sided Fisher exact test***		
		**n total**	**n total**	**Resolution vs. baseline**		**Persisting symptom**		**Resolution vs. baseline**		**Persisting symptom**		**Diff.**	**95% CI**		**OR**	**95% CI**		**χ2MH**	**df**	**P**	**Week2**	**Week4**	**Week6**
				**n**	**%**	**n**	**%**	**n**	**%**	**n**	**%**		**LCL**	**UCL**		**LCL**	**UCL**				**P**	**P**	**P**
Rhinitis/rhinorrhea	Week 2	111	100	86	77.48%	25	22.52%	68	68.00%	32	32.00%	9.48%	-2.52%	21.48%	1.62	0.88	2.99	2.96	2	0.085	0.162	0.266	1.000
	Week 4	111	100	96	86.49%	15	13.51%	80	80.00%	20	20.00%	6.49%	-3.61%	16.58%	1.60	0.77	3.33						
	Week 6	111	100	107	96.40%	4	3.60%	97	97.00%	3	3.00%	-0.60%	-5.42%	4.21%	0.83	0.18	3.79						
Sore throat	Week 2	43	54	40	93.02%	3	6.98%	45	83.33%	9	16.67%	9.69%	-2.83%	22.21%	2.67	0.67	10.54	0.56	2	0.453	0.217	1.000	0.443
	Week 4	43	54	39	90.70%	4	9.30%	48	88.89%	6	11.11%	1.81%	-10.26%	13.88%	1.22	0.32	4.63						
	Week 6	43	54	42	97.67%	1	2.33%	54	100.00%	0	0.00%	-2.33%	-6.83%	2.18%	0.39	0.01	11.87						
Hoarseness	Week 2	45	61	38	84.44%	7	15.56%	55	90.16%	0	0.00%	-5.72%	-18.68%	7.24%	0.59	0.18	1.90	0.00	2	0.974	0.388	0.238	1.000
	Week 4	45	61	44	97.78%	1	2.22%	56	91.80%	0	0.00%	5.97%	-2.15%	14.09%	3.93	0.44	34.86						
	Week 6	45	61	45	100.00%	0	0.00%	61	100.00%	0	0.00%	n/a											
Pneumonia	Week 2	0	0	0	0.00%	0	0.00%	0	0.00%	0	0.00%	n/a											
	Week 4	0	0	0	0.00%	0	0.00%	0	0.00%	0	0.00%	n/a											
	Week 6	0	0	0	0.00%	0	0.00%	0	0.00%	0	0.00%	n/a											
Muscle pain	Week 2	42	53	40	95.24%	2	4.76%	51	96.23%	2	3.77%	-0.99%	-9.22%	7.25%	0.78	0.11	5.81	0.19	2	0.667	1.000	0.696	1.000
	Week 4	42	53	38	90.48%	4	9.52%	50	94.34%	3	5.66%	-3.86%	-14.70%	6.98%	0.57	0.12	2.70						
	Week 6	42	53	41	97.62%	1	2.38%	52	98.11%	1	1.89%	-0.49%	-6.38%	5.39%	0.79	0.05	12.99						
Joint pain	Week 2	33	27	30	90.91%	3	9.09%	24	88.89%	3	11.11%	2.02%	-13.37%	17.41%	1.25	0.23	6.76	0.60	2	0.437	1.000	0.620	0.245
	Week 4	33	27	30	90.91%	3	9.09%	26	96.30%	1	3.70%	-5.39%	-17.51%	6.74%	0.38	0.04	3.93						
	Week 6	33	27	30	90.91%	3	9.09%	27	100.00%	0	0.00%	-9.09%	-18.90%	0.72%	0.19	0.01	3.87						
Limb pain	Week 2	39	52	37	94.87%	2	5.13%	49	94.23%	3	5.77%	0.64%	-8.74%	10.03%	1.13	0.18	7.13	0.15	2	0.698	1.000	1.000	1.000
	Week 4	39	52	39	100.00%	0	0.00%	51	98.08%	1	1.92%	1.92%	-1.81%	5.66%	1.53	0.05	46.77						
	Week 6	39	52	39	100.00%	0	0.00%	51	98.08%	1	1.92%	1.92%	-1.81%	5.66%	1.53	0.05	46.77						
Chest pain	Week 2	29	32	27	93.10%	2	6.90%	29	90.63%	3	9.38%	2.48%	-11.20%	16.16%	1.40	0.22	9.01	0.12	2	0.727	1.000	1.000	1.000
	Week 4	29	32	29	100.00%	0	0.00%	32	100.00%	0	0.00%	n/a											
	Week 6	29	32	29	100.00%	0	0.00%	32	100.00%	0	0.00%	n/a											
Headache	Week 2	66	72	54	81.82%	12	18.18%	50	69.44%	22	30.56%	12.37%	-1.76%	26.51%	1.98	0.89	4.41	2.31	2	0.129	0.115	0.434	1.000
	Week 4	66	72	60	90.91%	6	9.09%	62	86.11%	10	13.89%	4.80%	-5.78%	15.38%	1.61	0.55	4.71						
	Week 6	66	72	62	93.94%	4	6.06%	68	94.44%	4	5.56%	-0.51%	-8.32%	7.31%	0.91	0.22	3.80						

**Symptoms**	**Time**	**Nicotinamide**	**Placebo**	**Nicotinamide**				**Placebo**				**Difference**			**Odds Ratio**			**Cochran-Mantel-Haenszel test**			**Two-sided Fisher exact test***		
		**n total**	**n total**	**Resolution vs. baseline**		**Persisting symptom**		**Resolution vs. baseline**		**Persisting symptom**		**Diff.**	**95% CI**		**OR**	**95% CI**		**χ2MH**	**df**	**P**	**Week2**	**Week4**	**Week6**
				**n**	**%**	**n**	**%**	**n**	**%**	**n**	**%**		**LCL**	**UCL**		**LCL**	**UCL**				**P**	**P**	**P**
Abdominal pain	Week 2	10	15	9	90.00%	1	10.00%	13	86.67%	2	13.33%	3.33%	-22.00%	28.66%	1.38	0.11	17.67	0.00	2	1.000	1.000	1.000	1.000
	Week 4	10	15	10	100.00%	0	0.00%	15	100.00%	0	0.00%	n/a											
	Week 6	10	15	9	90.00%	1	10.00%	14	93.33%	1	6.67%	-3.33%	-25.81%	19.14%	0.64	0.04	11.63						
Diarrhea	Week 2	18	31	16	88.89%	2	11.11%	27	87.10%	4	12.90%	1.79%	-16.92%	20.50%	1.19	0.19	7.22	0.06	2	0.800	1.000	1.000	1.000
	Week 4	18	31	17	94.44%	1	5.56%	29	93.55%	2	6.45%	0.90%	-12.77%	14.56%	1.17	0.10	13.92						
	Week 6	18	31	17	94.44%	1	5.56%	29	93.55%	2	6.45%	0.90%	-12.77%	14.56%	1.17	0.10	13.92						
Nausea	Week 2	16	26	13	81.25%	3	18.75%	25	96.15%	1	3.85%	-14.90%	-35.41%	5.60%	0.17	0.02	1.84	1.10	2	0.295	0.146	1.000	0.381
	Week 4	16	26	16	100.00%	0	0.00%	25	96.15%	1	3.85%	3.85%	-3.55%	11.24%	1.28	0.04	40.41						
	Week 6	16	26	15	93.75%	1	6.25%	26	100.00%	0	0.00%	-6.25%	-18.11%	5.61%	0.29	0.01	9.12						
Vomiting	Week 2	1	2	1	100.00%	0	0.00%	2	100.00%	0	0.00%	n/a											
	Week 4	1	2	1	100.00%	0	0.00%	2	100.00%	0	0.00%	n/a											
	Week 6	1	2	1	100.00%	0	0.00%	2	100.00%	0	0.00%	n/a											
Loss of appetite / lower food intake	Week 2	78	81	73	93.59%	5	6.41%	73	90.12%	8	9.88%	3.47%	-5.01%	11.94%	1.60	0.50	5.12	1.43	2	0.232	0.565	0.443	1.000
	Week 4	78	81	76	97.44%	2	2.56%	76	93.83%	5	6.17%	3.61%	-2.70%	9.92%	2.50	0.47	13.29						
	Week 6	78	81	77	98.72%	1	1.28%	79	97.53%	2	2.47%	1.19%	-3.01%	5.39%	1.95	0.17	21.94						
Other gastrointes-tinal symptoms	Week 2	6	5	5	83.33%	1	16.67%	4	80.00%	1	20.00%	3.33%	-42.69%	49.36%	1.25	0.06	26.87	0.49	2	0.482	1.000	0.455	0.455
	Week 4	6	5	6	100.00%	0	0.00%	4	80.00%	1	20.00%	20.00%	-15.06%	55.06%	3.00	0.08	112.34						
	Week 6	6	5	6	100.00%	0	0.00%	4	80.00%	1	20.00%	20.00%	-15.06%	55.06%	3.00	0.08	112.34						
Impaired sense of smell	Week 2	69	80	40	57.97%	29	42.03%	40	50.00%	40	50.00%	7.97%	-8.02%	23.96%	1.38	0.72	2.64	3.12	2	0.077	0.410	0.592	0.114
	Week 4	69	80	50	72.46%	19	27.54%	54	67.50%	26	32.50%	4.96%	-9.75%	19.68%	1.27	0.63	2.57						
	Week 6	69	80	58	84.06%	11	15.94%	58	72.50%	22	27.50%	11.56%	-1.49%	24.61%	2.00	0.89	4.50						
Impaired sense of taste	Week 2	59	81	33	55.93%	26	44.07%	43	53.09%	38	46.91%	2.85%	-13.85%	19.54%	1.12	0.57	2.20	1.58	2	0.209	0.864	0.354	0.326
	Week 4	59	81	44	74.58%	15	25.42%	54	66.67%	27	33.33%	7.91%	-7.22%	23.04%	1.47	0.70	3.09						
	Week 6	59	81	47	79.66%	12	20.34%	58	71.60%	23	28.40%	8.06%	-6.15%	22.27%	1.55	0.70	3.45						
Confusion	Week 2	8	8	8	100.00%	0	0.00%	7	87.50%	1	12.50%	12.50%	-10.42%	35.42%	2.29	0.07	79.03	0.00	2	1.000	1.000	1.000	1.000
	Week 4	8	8	8	100.00%	0	0.00%	8	100.00%	0	0.00%	n/a											
	Week 6	8	8	8	100.00%	0	0.00%	8	100.00%	0	0.00%	n/a											

Symptoms	Time	Nicotinamide	Placebo	Nicotinamide				Placebo				Difference			Odds Ratio			Cochran-Mantel-Haenszel test			Two-sided Fisher exact test*		
		n total	n total	Resolution vs. baseline		Persisting symptom		Resolution vs. baseline		Persisting symptom		Diff.	95% CI		OR	95% CI		χ2MH	df	P	Week2	Week4	Week6
				n	%	n	%	n	%	n	%		LCL	UCL		LCL	UCL				P	P	P
Dizziness	Week 2	33	40	30	90.91%	3	9.09%	33	82.50%	7	17.50%	8.41%	-6.92%	23.73%	2.12	0.50	8.95	0.02	2	0.879	0.496	1.000	0.586
	Week 4	33	40	32	96.97%	1	3.03%	39	97.50%	1	2.50%	-0.53%	-8.12%	7.06%	0.82	0.05	13.64						
	Week 6	33	40	31	93.94%	2	6.06%	39	97.50%	1	2.50%	-3.56%	-13.03%	5.91%	0.40	0.03	4.59						
Conjunctivitis	Week 2	2	5	1	50.00%	1	50.00%	5	100.00%	0	0.00%	-50.00%	-119.30%	19.30%	0.10	0.00	5.55	0.45	2	0.502	0.286	1.000	1.000
	Week 4	2	5	2	100.00%	0	0.00%	4	80.00%	1	20.00%	20.00%	-15.06%	55.06%	1.00	0.02	44.50						
	Week 6	2	5	2	100.00%	0	0.00%	5	100.00%	0	0.00%	n/a											
Skin rash	Week 2	10	8	9	90.00%	1	10.00%	8	100.00%	0	0.00%	-10.00%	-28.59%	8.59%	0.56	0.02	19.12	0.31	2	0.580	1.000	1.000	1.000
	Week 4	10	8	10	100.00%	0	0.00%	8	100.00%	0	0.00%	n/a											
	Week 6	10	8	9	90.00%	1	10.00%	8	100.00%	0	0.00%	-10.00%	-28.59%	8.59%	0.56	0.02	19.12						
Hair loss	Week 2	6	3	5	83.33%	1	16.67%	3	100.00%	0	0.00%	-16.67%	-46.49%	13.15%	0.83	0.02	33.18	0.06	2	0.803	1.000	1.000	1.000
	Week 4	6	3	5	83.33%	1	16.67%	3	100.00%	0	0.00%	-16.67%	-46.49%	13.15%	0.83	0.02	33.18						
	Week 6	6	3	6	100.00%	0	0.00%	3	100.00%	0	0.00%	n/a											
Other symptoms	Week 2	44	52	28	63.64%	16	36.36%	40	76.92%	12	23.08%	-13.29%	-31.54%	4.97%	0.53	0.22	1.28	5.36	2	0.021	0.181	0.223	0.223
	Week 4	44	52	31	70.45%	13	29.55%	43	82.69%	9	17.31%	-12.24%	-29.19%	4.72%	0.50	0.19	1.31						
	Week 6	44	52	31	70.45%	13	29.55%	43	82.69%	9	17.31%	-12.24%	-29.19%	4.72%	0.50	0.19	1.31						

CI, confidence interval; LCL, lower confidence limit; n, number of patients with non-missing values used for the respective analysis; n/a, not applicable; OR, odds ratio; UCL, upper confidence limit.

* Adjusted P values (see section 6).

Supplementary Table 11: Effects of nicotinamide on COVID-19 symptoms (change to baseline) in the entire RFITT population (complaint scale).

Characteristics / symptoms	Time	Nicotinamide			Placebo			Contrast	95% CI		T	df	P
		n	Mean	SE	n	Mean	SE		LCL	UCL			
Ability to perform normal activities	Week 2	103	-3.07	0.12	95	-2.62	0.13	-0.45	-0.80	-0.11	-2.62	519.79	0.009
	Week 4	103	-3.49	0.12	95	-3.34	0.13	-0.15	-0.49	0.20	-0.84	519.79	0.400
	Week 6	103	-3.87	0.12	95	-3.92	0.13	0.05	-0.29	0.40	0.31	519.79	0.758
Cough	Week 2	44	-3.22	0.16	33	-2.76	0.18	-0.46	-0.94	0.02	-1.91	249.68	0.057
	Week 4	44	-3.43	0.16	33	-3.43	0.18	0.00	-0.48	0.48	0.00	249.68	0.997
	Week 6	44	-4.07	0.16	33	-4.01	0.18	-0.06	-0.54	0.42	-0.25	249.68	0.805
Mucus production	Week 2	40	-3.43	0.16	25	-3.15	0.20	-0.28	-0.78	0.22	-1.09	224.75	0.276
	Week 4	40	-3.61	0.16	25	-3.55	0.20	-0.05	-0.56	0.45	-0.21	224.75	0.834
	Week 6	40	-4.08	0.16	25	-4.03	0.20	-0.05	-0.55	0.45	-0.19	224.75	0.850
Shortness of breath	Week 2	20	-2.23	0.35	27	-2.20	0.30	-0.02	-0.94	0.89	-0.05	123.64	0.959
	Week 4	20	-2.78	0.35	27	-2.61	0.30	-0.17	-1.08	0.74	-0.36	123.64	0.718
	Week 6	20	-3.33	0.35	27	-2.98	0.30	-0.35	-1.26	0.57	-0.75	123.64	0.453
Sleep	Week 2	66	-3.22	0.17	61	-3.01	0.17	-0.20	-0.68	0.27	-0.85	415.15	0.399
	Week 4	66	-3.11	0.17	61	-3.67	0.17	0.56	0.09	1.03	2.33	415.15	0.020
	Week 6	66	-3.50	0.17	61	-3.93	0.17	0.43	-0.04	0.90	1.78	415.15	0.076
General feeling of sickness	Week 2	78	-2.92	0.12	76	-2.66	0.12	-0.27	-0.60	0.07	-1.56	461.39	0.120
	Week 4	78	-3.35	0.12	76	-3.42	0.12	0.07	-0.26	0.41	0.43	461.39	0.665
	Week 6	78	-3.69	0.12	76	-3.97	0.12	0.28	-0.06	0.62	1.64	461.39	0.101

According to the SAP, only patients with severe complaints (baseline score of >3) were included in the analyses.

CI, confidence interval; LCL, lower confidence limit; n, number of patients with non-missing values used for the respective analysis; SE, standard error; UCL, upper confidence limit.

Two-sided t-test of contrasts within a mixed model for repeated measures; adjusted P values (see section 6).

Supplementary Table 12: Effects of nicotinamide on COVID-19 symptoms (change to baseline) in males of the RFITT population (complaint scale).

Characteristics / symptoms	Time	Nicotinamide			Placebo			Contrast	95% CI		T	df	P
		n	Mean	SE	n	Mean	SE		LCL	UCL			
Ability to perform normal activities	Week 2	41	-3.34	0.17	34	-3.00	0.19	-0.34	-0.86	0.17	-1.33	378.49	0.185
	Week 4	41	-3.66	0.17	34	-3.53	0.19	-0.13	-0.64	0.38	-0.51	378.49	0.611
	Week 6	41	-3.90	0.17	34	-4.15	0.19	0.24	-0.27	0.75	0.93	378.49	0.352
Cough	Week 2	17	-3.31	0.21	8	-3.09	0.31	-0.22	-0.97	0.53	-0.58	141.86	0.567
	Week 4	17	-3.54	0.21	8	-3.72	0.31	0.17	-0.58	0.92	0.46	141.86	0.649
	Week 6	17	-4.07	0.21	8	-4.09	0.31	0.02	-0.73	0.77	0.05	141.86	0.962
Mucus production	Week 2	21	-3.11	0.19	6	-4.02	0.33	0.92	0.19	1.64	2.51	151.71	0.014
	Week 4	21	-3.92	0.19	6	-3.69	0.33	-0.23	-0.95	0.49	-0.63	151.71	0.533
	Week 6	21	-4.25	0.19	6	-4.19	0.33	-0.06	-0.78	0.66	-0.17	151.71	0.867
Shortness of breath	Week 2	8	-2.38	0.39	6	-3.33	0.45	0.96	-0.24	2.16	1.61	86.55	0.115
	Week 4	8	-2.88	0.39	6	-3.00	0.45	0.12	-1.08	1.33	0.21	86.55	0.835
	Week 6	8	-3.88	0.39	6	-3.83	0.45	-0.04	-1.24	1.16	-0.07	86.55	0.945
Sleep	Week 2	32	-3.34	0.21	25	-3.49	0.23	0.15	-0.46	0.77	0.49	358.54	0.622
	Week 4	32	-3.43	0.21	25	-4.13	0.23	0.70	0.09	1.32	2.24	358.54	0.026
	Week 6	32	-3.96	0.21	25	-4.17	0.23	0.21	-0.41	0.82	0.67	358.54	0.503
General feeling of sickness	Week 2	32	-3.37	0.18	27	-2.83	0.19	-0.54	-1.06	-0.02	-2.05	349.00	0.042
	Week 4	32	-3.68	0.18	27	-3.57	0.19	-0.11	-0.63	0.41	-0.42	349.00	0.674
	Week 6	32	-3.99	0.18	27	-4.20	0.19	0.21	-0.31	0.73	0.78	349.00	0.434

According to the SAP, only patients with severe complaints (baseline score of >3) were included in the analyses.

CI, confidence interval; LCL, lower confidence limit; n, number of patients with non-missing values used for the respective analysis; SE, standard error; UCL, upper confidence limit.

Two-sided, post-hoc, unadjusted t-test of contrasts within a mixed model for repeated measures.

Supplementary Table 13: Effects of nicotinamide on COVID-19 symptoms (change to baseline) in females of the RFITT population (complaint scale).

Characteristics / symptoms	Time	Nicotinamide			Placebo			Contrast	95% CI		T	df	P
		n	Mean	SE	n	Mean	SE		LCL	UCL			
Ability to perform normal activities	Week 2	62	-2.89	0.16	61	-2.41	0.16	-0.49	-0.94	-0.03	-2.11	620.72	0.035
	Week 4	62	-3.38	0.16	61	-3.24	0.16	-0.13	-0.59	0.32	-0.58	620.72	0.561
	Week 6	62	-3.84	0.16	61	-3.80	0.16	-0.04	-0.50	0.41	-0.19	620.72	0.847
Cough	Week 2	27	-3.17	0.22	25	-2.66	0.23	-0.51	-1.13	0.11	-1.61	295.08	0.109
	Week 4	27	-3.35	0.22	25	-3.34	0.23	-0.01	-0.63	0.61	-0.04	295.08	0.968
	Week 6	27	-4.06	0.22	25	-3.98	0.23	-0.08	-0.70	0.55	-0.24	295.08	0.808
Mucus production	Week 2	19	-3.97	0.25	19	-2.98	0.25	-0.99	-1.70	-0.29	-2.79	213.51	0.006
	Week 4	19	-3.44	0.25	19	-3.61	0.25	0.16	-0.54	0.87	0.46	213.51	0.648
	Week 6	19	-4.08	0.25	19	-4.08	0.25	0.01	-0.70	0.71	0.01	213.51	0.988
Shortness of breath	Week 2	12	-2.12	0.49	21	-1.88	0.37	-0.24	-1.47	0.99	-0.38	204.00	0.702
	Week 4	12	-2.70	0.49	21	-2.50	0.37	-0.20	-1.43	1.03	-0.33	204.00	0.745
	Week 6	12	-2.95	0.49	21	-2.74	0.37	-0.21	-1.45	1.02	-0.35	204.00	0.731
Sleep	Week 2	34	-3.10	0.25	36	-2.69	0.24	-0.41	-1.09	0.28	-1.17	440.31	0.244
	Week 4	34	-2.80	0.25	36	-3.35	0.24	0.55	-0.13	1.24	1.59	440.31	0.113
	Week 6	34	-3.07	0.25	36	-3.77	0.24	0.71	0.02	1.39	2.03	440.31	0.044
General feeling of sickness	Week 2	46	-2.62	0.16	49	-2.56	0.15	-0.06	-0.49	0.37	-0.28	561.95	0.781
	Week 4	46	-3.12	0.16	49	-3.34	0.15	0.21	-0.22	0.65	0.97	561.95	0.331
	Week 6	46	-3.49	0.16	49	-3.85	0.15	0.35	-0.08	0.79	1.61	561.95	0.108

According to the SAP, only patients with severe complaints (baseline score of >3) were included in the analyses.

CI, confidence interval; LCL, lower confidence limit; n, number of patients with non-missing values used for the respective analysis; SE, standard error; UCL, upper confidence limit.

Two-sided, post-hoc, unadjusted t-test of contrasts within a mixed model for repeated measures.

Supplementary Table 14: Effects of nicotinamide on SF-36 scales (change to baseline) in the RFITT population.

Subscale	Time	Nicotinamide			Placebo			Contrast	95% CI		T	df	P
		n	Mean	SE	n	Mean	SE		LCL	UCL			
Physical functioning	Week 2	113	31.62	2.02	111	32.89	2.04	-1.28	-6.93	4.38	-0.44	385.08	0.657
	Week 4	111	46.66	2.04	112	49.49	2.04	-2.83	-8.50	2.84	-0.98	386.27	0.327
	Week 6	110	55.08	2.04	111	57.98	2.04	-2.91	-8.59	2.77	-1.01	388.81	0.315
Role functioning/physical	Week 2	123	13.91	3.44	119	4.67	3.49	9.25	-0.39	18.89	1.89	4.91	0.060
	Week 4	121	32.41	3.46	120	27.87	3.48	4.53	-5.12	14.19	0.92	4.91	0.357
	Week 6	121	49.49	3.46	119	49.81	3.49	-0.32	-9.99	9.35	-0.07	4.92	0.948
Role functioning/emotional	Week 2	219	-12.53	2.57	221	-13.40	2.55	0.87	-6.23	7.98	0.24	896.46	0.809
	Week 4	216	-1.87	2.58	223	-4.45	2.55	2.58	-4.53	9.69	0.71	897.43	0.477
	Week 6	214	7.78	2.59	224	5.30	2.54	2.48	-4.64	9.60	0.69	899.25	0.494
Energy/fatigue	Week 2	122	2.70	1.76	113	1.30	1.83	1.41	-3.59	6.41	0.55	369.11	0.580
	Week 4	119	11.58	1.78	114	12.26	1.83	-0.68	-5.69	4.33	-0.27	371.22	0.789
	Week 6	118	20.70	1.78	114	21.68	1.83	-0.98	-6.00	4.04	-0.38	372.31	0.701
Emotional well-being	Week 2	132	1.05	1.34	121	0.23	1.40	0.82	-3.00	4.64	0.42	399.92	0.672
	Week 4	130	6.07	1.35	122	6.30	1.40	-0.23	-4.05	3.60	-0.12	400.89	0.906
	Week 6	127	12.56	1.36	122	12.22	1.40	0.34	-3.50	4.17	0.17	404.16	0.864
Social functioning	Week 2	116	3.50	2.17	115	3.98	2.19	-0.48	-6.54	5.58	-0.16	470.98	0.876
	Week 4	116	18.31	2.18	116	22.82	2.18	-4.51	-10.56	1.55	-1.46	469.21	0.144
	Week 6	115	34.84	2.18	116	34.78	2.18	0.06	-6.00	6.13	0.02	470.74	0.984
Pain	Week 2	125	9.48	1.97	120	5.16	2.02	4.32	-1.23	9.87	1.53	479.79	0.126
	Week 4	125	25.35	1.97	121	24.56	2.01	0.79	-4.75	6.34	0.28	478.04	0.778
	Week 6	125	38.85	1.97	121	38.34	2.01	0.51	-5.03	6.06	0.18	478.04	0.856
General health	Week 2	121	7.52	1.46	115	7.23	1.49	0.29	-3.82	4.39	0.14	339.03	0.891
	Week 4	118	12.77	1.47	116	12.02	1.49	0.75	-3.36	4.86	0.36	341.04	0.719
	Week 6	117	14.88	1.47	116	16.08	1.49	-1.20	-5.32	2.91	-0.58	341.92	0.565
Health change	Week 2	169	16.61	1.55	171	16.25	1.55	0.37	-3.94	4.67	0.17	593.36	0.867
	Week 4	166	24.35	1.56	170	24.80	1.55	-0.45	-4.77	3.87	-0.21	598.57	0.837
	Week 6	166	30.82	1.56	170	31.42	1.55	-0.60	-4.92	3.72	-0.27	598.64	0.787

According to the SAP, only patients with severe complaints (baseline values ≤ median) were included in the analyses.

CI, confidence interval; LCL, lower confidence limit; n, number of patients with non-missing values used for the respective analysis; SE, standard error; UCL, upper confidence limit.

Two-sided t-test of contrasts within a mixed model for repeated measures, adjusted for multiple timepoints.

Supplementary Table 15: Effects of nicotinamide on FACIT-F scales (change to baseline) in the RFITT population.

Subscale / score	Time	Nicotinamide			Placebo			Contrast	95% CI		T	df	P
		n	Mean	SE	n	Mean	SE		LCL	UCL			
Physical well-being	Week 2	99	9.40	0.49	106	8.98	0.48	0.42	-0.93	1.77	0.61	366.00	0.541
	Week 4	99	12.96	0.49	106	12.95	0.48	0.00	-1.35	1.35	0.00	365.15	0.998
	Week 6	100	14.38	0.49	107	14.88	0.48	-0.50	-1.85	0.85	-0.73	362.81	0.464
Social/family well-being	Week 2	111	0.83	0.33	122	0.75	0.31	0.08	-0.80	0.97	0.18	370.77	0.854
	Week 4	109	1.28	0.33	122	1.35	0.31	-0.07	-0.95	0.83	-0.14	373.38	0.887
	Week 6	109	1.79	0.33	122	1.78	0.31	0.01	-0.88	0.90	0.03	373.38	0.980
Emotional well-being	Week 2	127	3.25	0.30	124	2.72	0.30	0.53	-0.31	1.37	1.23	426.89	0.218
	Week 4	123	4.50	0.30	125	4.19	0.30	0.31	-0.54	1.15	0.72	430.73	0.475
	Week 6	120	5.16	0.31	123	4.96	0.31	0.20	-0.65	1.05	0.47	437.30	0.638
Functional well-being	Week 2	117	5.77	0.54	123	5.61	0.52	0.16	-1.31	1.63	0.22	400.96	0.828
	Week 4	115	9.34	0.54	123	8.86	0.52	0.48	-1.00	1.95	0.64	403.53	0.524
	Week 6	114	11.30	0.54	124	11.34	0.52	-0.04	-1.52	1.43	-0.06	403.71	0.955
Fatigue	Week 2	112	16.09	1.05	112	15.97	1.06	0.12	-2.82	3.05	0.08	382.10	0.937
	Week 4	113	23.56	1.05	111	24.35	1.06	-0.79	-3.72	2.15	-0.53	382.12	0.599
	Week 6	112	27.65	1.05	112	27.86	1.06	-0.21	-3.14	2.73	-0.14	381.95	0.891
FACIT-F Trial Outcome Index	Week 2	97	27.91	2.03	99	29.51	2.01	-1.60	-7.22	4.02	-0.56	334.14	0.576
	Week 4	98	42.37	2.02	98	45.45	2.02	-3.08	-8.70	2.53	-1.08	334.02	0.281
	Week 6	97	50.88	2.03	100	52.99	2.01	-2.11	-7.72	3.50	-0.74	332.71	0.460
FACT-G Score	Week 2	91	15.87	1.50	101	15.25	1.43	0.61	-3.47	4.70	0.30	304.45	0.768
	Week 4	90	24.00	1.51	100	25.30	1.43	-1.30	-5.40	2.80	-0.62	306.74	0.533
	Week 6	87	29.30	1.52	102	29.68	1.43	-0.38	-4.49	3.72	-0.18	308.88	0.854
FACIT-F General Score	Week 2	94	31.23	2.47	94	32.34	2.48	-1.11	-8.01	5.79	-0.32	309.36	0.752
	Week 4	94	47.24	2.47	91	50.16	2.50	-2.91	-9.84	4.02	-0.83	313.16	0.409
	Week 6	92	56.47	2.48	94	57.71	2.48	-1.23	-8.16	5.69	-0.35	311.88	0.726

According to the SAP, only patients with severe complaints (baseline values ≤ median) were included in the analyses.

CI, confidence interval; LCL, lower confidence limit; n, number of patients with non-missing values used for the respective analysis; SE, standard error; UCL, upper confidence limit.

Two-sided t-test of contrasts within a mixed model for repeated measures, adjusted for multiple timepoints.

2.3. Supplementary clinical data (text)

Levels of antibodies directed against the nucleocapsid (N) or spike (S) proteins of SARS-CoV-2 after at least 6 months (secondary endpoint)

For the RFITT population, no differences between the trial arms were observed with respect to the anti-N antibodies (D = 2.7, p = 0.493) and anti-S antibodies (D = 3.0, p = 0.983). Within the subgroup of vaccinated patients, no significant difference (nicotinamide vs. placebo) was seen for anti-N antibodies (D = 0.10; p = 0.985) and anti-S antibodies (D = -6.2, p = 0.974). For the subgroups of unvaccinated patients, differences between the nicotinamide and placebo groups with respect to anti-N antibodies (D = 7.8, p = 0.248) and anti-S antibodies (D = 46.0, p = 0.137) were also not significant. With respect to anti-S antibodies, RFITT responders to the nicotinamide intervention showed a non-significant difference (nicotinamide vs. placebo) of D = 154.0, p = 0.363, in the subgroup of vaccinated responders (D = 138.0, p = 0.530) and in the subgroup of unvaccinated responders (D = 55.0, p = 0.135).

Symptom resolution over time (secondary endpoints)

In the interviews at weeks 2, 4 and 6, patients were asked whether they were free of individual or all symptoms. Using this variable, 60 patients (24.19%) of the for RFITT population receiving nicotinamide and 48 patients (19.05%) receiving placebo reported to be symptom-free at week 2 (p = 0.192). At week 4, 103 patients (41.53%) receiving nicotinamide and 101 patients (40.08%) receiving placebo reported to be symptom-free (p = 0.785). At week 6, 129 patients (52.02%) receiving nicotinamide and 129 patients (51.19%) receiving placebo reported to be symptom-free (p = 0.858). For primary and key secondary endpoints as well as for being completely symptom-free, the time to resolution was analyzed using a Kaplan-Meier analysis. Only symptomatic patients who had the respectively symptom at baseline (binary queries) or showed symptom scores greater than 3 (ordinal queries) (see section 3.2 of this Supplement) were included. 'Symptom-free' was defined as a rating of zero on a scale of complaints. For RFITT patients, no significant differences between the trial arms were observed for time to resolution of performance drop (HR 0.83; 95% CI [0.66; 1.03]; p = 0.087), the ability to perform normal activities (HR 0.72; 95% CI [0.52; 1.00]; p = 0.051), fatigue (HR 0.92; 95% CI [0.75; 1.14]; p = 0.453) cough (HR 0.81; 95% CI [0.49; 1.34]; p = 0.414) or being completely symptom-free (HR 0.91; 95% CI [0.73; 1.13]; p = 0.79). The time from study inclusion (screening interview) to resolution of individual symptoms (in days) as reported within the patients' interviews was analyzed using Cox regression. For RFITT, no difference between the interventions was observed (HR 0.89; 95% CI [0.70; 1.13]; p = 0.327).

WHO COVID-19 ordinal scale for clinical improvement and occurrence of severe COVID-19 (exploratory endpoints)

The results are summarized in the safety evaluation in section 2.5 of this Supplement (Supplementary Tables 25–29.

Molecular exploratory endpoints

For the exploratory endpoints blood biomarkers, blood metabolome, SARS-CoV-2 strains and pharmacokinetics., quarantine rules did not allow us to acquire biosamples from more than 12 local patients. Therefore, a formal analysis would lack statistical power, but data can be obtained from the corresponding author.

2.4. Supplementary data from gut microbiome analyses

To assess longitudinal shifts in fecal microbial communities associated with nicotinamide, we performed 16S rDNA phylogenomic profiling and metagenomic sequencing analyses. DNA of stool samples of 88 independent patients (70 patients for 16S and 18 patients for metagenomics), which were collected at week 0 (baseline), weeks 2 and 4 (exposure to nicotinamide or placebo) and week 6 (follow-up), was extracted and subjected to 16S rRNA (n=280) and shotgun metagenomics (n=72) sequencing. A schematic study design is depicted in Fig. 2a.

After quality control, different α-diversity (within sample diversity) analyses of 16S rDNA data did not show significant longitudinal or cross-sectional differences between intervention groups (Fig. 2b and Supplementary Fig. 3).

β-diversity (between sample diversity) analyses (Aitchison distance) did not reveal a significant difference between intervention groups at baseline (Supplementary Table 19). Longitudinal, intervention-dependent β-diversity differences were computed using global scaling and constraint analyses of principal coordinates (CAP) on Aitchison distances from baseline. Differences between nicotinamide and placebo during the intervention period (week 2 and week 4) were tested with PERMANOVA with 10,000 permutations (Fig. 2c; Supplementary Table 18). Here, the gut microbiome composition of patients receiving nicotinamide significantly differed from those receiving placebo (R^2 = 0.015, false discovery rate [FDR] = 0.002). Interestingly, when identifying contributive factors associated with dissimilarity, we demonstrate that key COVID-19-related symptoms (fever, cough, impaired sense of smell or impaired sense of taste at baseline) (R^2 = 0.0067, FDR = 0.013) and placebo had a similar effect in the post-baseline samples, which was opposite to the nicotinamide group (Fig. 2c, Supplementary Table 18). Likewise, we ran a principal coordinate analysis of the 16S rRNA amplicon sequences (Supplementary Fig.s 4a and 4b) and metagenomics (Supplementary Fig.s 4c and 4d), suggesting similar microbial compositions among both cohorts.

A formal variance partition analysis was conducted at baseline to evaluate the contribution of patient covariates to the genus-level microbial composition. Among the factors, fever before baseline showed the highest contribution to the variance (3%), followed by body mass index (BMI) (2.7%), age (2.7%), and other COVID-19-specific symptoms (1.7%) (Supplementary Fig. 5, Supplementary Table 24). The highest variance contribution by fever confirms that the microbial composition of patients with COVID-19 seems to be driven by disease severity, which has been suggested previously.[1] When stratifying α-diversity indices for the presence of fever at baseline in patients with key COVID-19-related symptoms using mixed-effect regressions, we found that also within sample diversity was decreased in affected patients at early timepoints, with varying effect sizes for different indices (Supplementary Fig. 6). Significantly sustained reduction of α-diversity in patients with fever at baseline in the placebo arm was observed in Shannon and Simpson indices. No significant α-diversity reduction (Shannon and Simpson) was observed in patients receiving nicotinamide at timepoints week 4 and 6.

Further variance partition analyses were done to identify the top 20 most variable genera associated to nicotinamide intervention in the 16S rDNA amplicon sequencing cohort (Fig. 2d, Supplementary Table 20) and in the metagenomics cohort (Supplementary Fig. 7a, Supplementary Table 21) at week 2 and week 4 of intervention. We found an overlap in 11 genera from the top 50 most variable genera between both cohorts (Supplementary Fig. 7b).

To address the putative metabolic potential of fecal microbial communities according to the respective intervention groups, we analyzed metagenomic data from an independent trial patient set. Using assignment of taxonomic features and quantification of microbial communities by MetaPhlAn 3.0 and inference of functional potential profiling by HUMAnN 3.0,[ref. 2] we observed high abundance of amino acid, cofactor and vitamin biosynthesis pathways. Strikingly, there was a significant increase of tryptophan biosynthesis in patients receiving placebo compared to patients receiving nicotinamide at week 2, indicating a higher demand for *de novo* synthesis of this amino acid as expected from earlier findings. This shift was reduced in samples from the nicotinamide group. Note that at the functional level the strongest difference between placebo and active arm was observed at week 2, which reflects the timepoint with the strongest clinical effect of nicotinamide (Fig. 3). A complete list of abundance shifts of inferred pathways according to intervention and timepoint is presented in Supplementary Table 22. Overall, the findings indicate an influence of symptomatic COVID-19 on gut microbial communities, which was influenced by nicotinamide as a metabolic intervention.

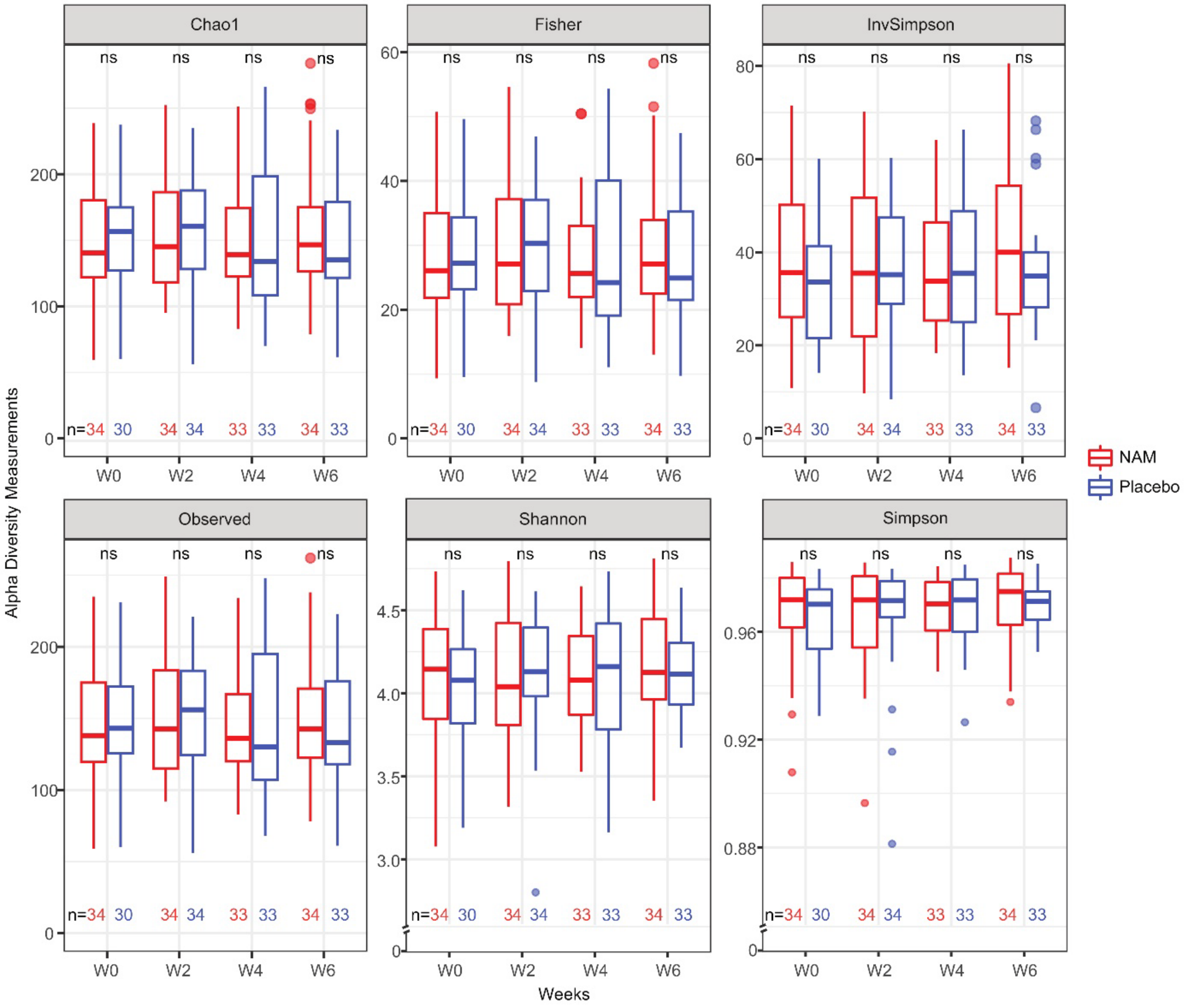

Supplementary Fig. 3: α-diversity metrics in relation to the intervention.
All the indexes were computed at the Amplicon Sequence Variant (ASV) level. Cross-sectional comparisons between nicotinamide (NAM) and placebo were performed using the two-sided Wilcoxon rank-sum test (n per group is depicted below each box plot; ns, not significant). Only samples with a minimum of 5,000 reads were included. Linear mixed-effect models with random intercepts for subjects evaluated the effect of intervention time. A likelihood ratio test comparing the full and null models showed no significant effect of time. Box plots show the median (center line), interquartile range (IQR, box), 1.5x IQR (whiskers) and outliers (points).

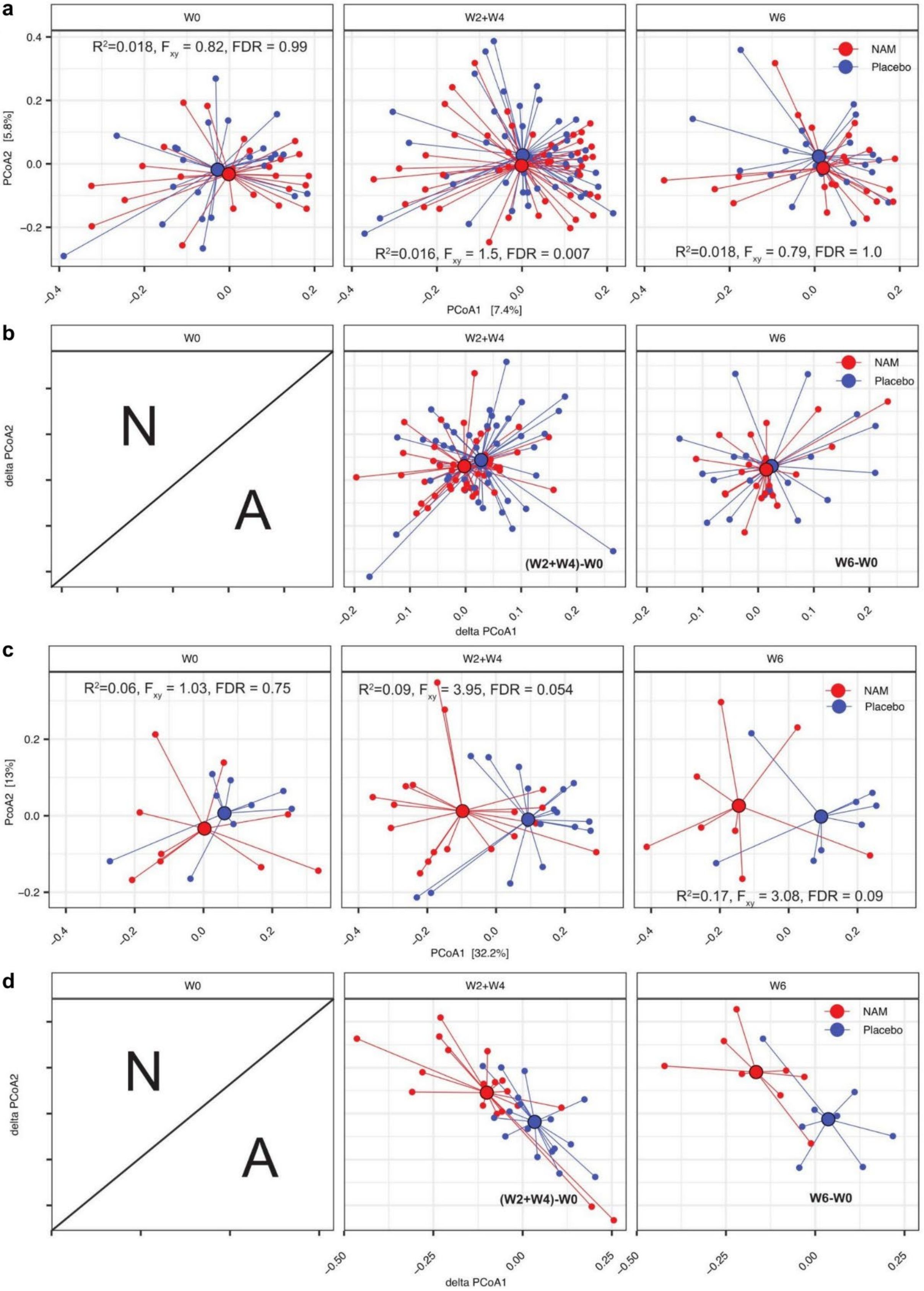

Supplementary Fig. 4 | Principal Coordinate Analysis (PCoA) based on Bray-Curtis distances showing microbial community composition changes in patients with key COVID-19-related symptoms.
a, Cohort 1, 16S rRNA gene sequencing data. PCoA plots show microbial community composition at baseline (week 0 [W0]; 24 patients receiving nicotinamide [NAM], 23 placebo), weeks 2–4 (W2+W4; n=45 per intervention), and week 6 (W6; n=23 NAM, n=21 placebo). Significant differences between the NAM and placebo

were observed at W2+W4 (R^2=0.016, F=1.50, false discovery rate [FDR]=0.007). Individual samples are connected to the group centroids for each timepoint. **b**, Cohort 1, 16S rRNA gene sequencing data (W0: n=24 NAM, n=23 placebo; W2+W4: n=45 per intervention; W6: n=23 NAM, n=21 placebo). Delta PCoA plots representing changes in principal coordinates relative to baseline (W0). For each sample, the baseline principal coordinates were subtracted from the corresponding intervention timepoints ((W2+W4) – W0) or follow-up timepoints (W6 – W0). This delta visualization emphasizes shifts in community composition during the intervention (W2+W4) and follow-up (W6) phases and was calculated as described by Maifeld et al. (2021).[3] N/A, not applicable. **c**, Cohort 2, shotgun metagenomics data (n=9 per intervention and timepoint). PCoA plots at the same time points as in panel **a** show a trend toward significance at W2+W4 (R^2=0.09, F=3.95, FDR=0.054) and W6 (R^2=0.17, F=3.08, FDR=0.09). **d,** Cohort 2, shotgun metagenomics data (n=9 per intervention and timepoint). Delta PCoA plots analogous to panel **b**, showing changes in principal coordinates relative to baseline (W0). The delta approach captures temporal shifts in microbial community composition for NAM and placebo groups during intervention and follow-up phases. PERMANOVA test with 10.000 permutations for intervention effects were done on the original distance matrices using the adonis function in the vegan (2.5-5) R package, stratified for patient ID (see section 3.5 of this Supplement). Key COVID-19-related symptoms were fever, cough, impaired sense of smell or impaired sense of taste at baseline. Only samples with a minimum of 5,000 reads were included. FDR represents Benjamini-Hochberg-corrected P values. N/A, not applicable.

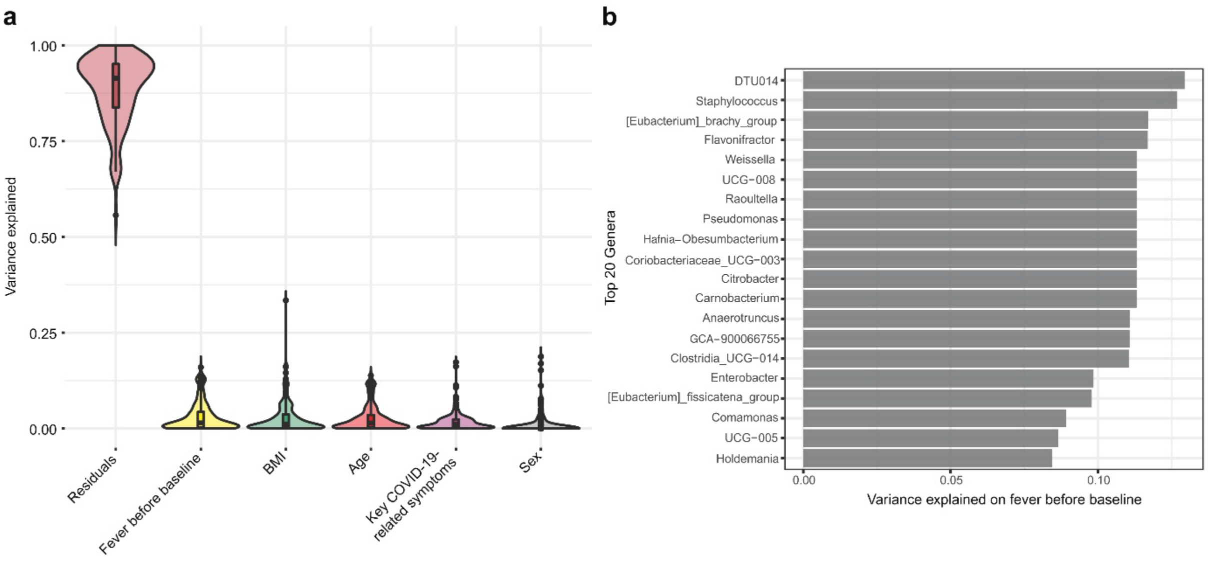

Supplementary Fig. 5: Variance contribution of baseline characteristics.
a, The effects of important patient covariates on the genus-level gut microbiome composition at baseline were evaluated using a variance partition analysis (34 patients receiving nicotinamide, 30 placebo; see also Supplementary Table 24). Only samples with a minimum of 5,000 reads were included. Fever before baseline showed the highest contribution to the variance (3%), followed by body mass index (BMI) (2.7%), age (2.7%), and other key COVID-19-related symptoms (1.7%). **b**, The 20 most important genera contributing to the variance associated with fever before baseline.

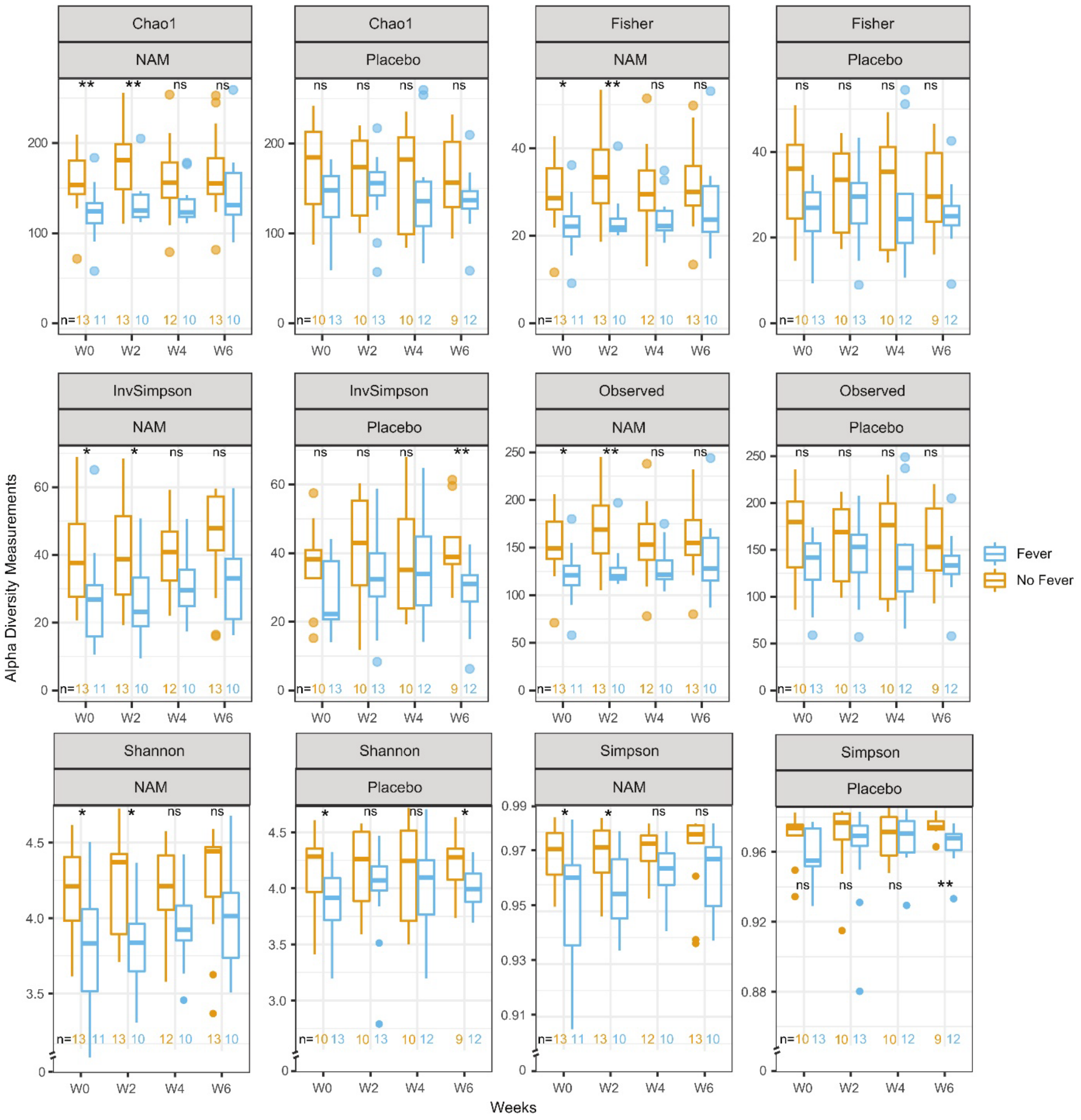

Supplementary Fig. 6: Longitudinal analysis of α-diversity in relation to fever in patients with key COVID-19-related symptoms.

Shifts in microbial diversity within samples were analyzed by α-diversity measurements. Among the genera mostly contributing to those differences we found *Staphylococcus*, *Pseudomonas*, and *Citrobacter*. All indices were computed at the Amplicon Sequence Variant (ASV) level. Cross-sectional comparisons between 'Fever' and 'No Fever' at baseline were performed using the two-sided Wilcoxon rank-sum test (n per group is depicted below each box plot). Asterisks represent significant P values (* P<0.05, ** P<0.01), corrected for multiple comparisons. Box plots show the median (center line), interquartile range (IQR, box), 1.5x IQR (whiskers) and outliers (points). Key COVID-19-related symptoms were fever, cough, impaired sense of smell or impaired sense of taste at baseline. Only samples with a minimum of 5,000 reads were included. NAM, nicotinamide.

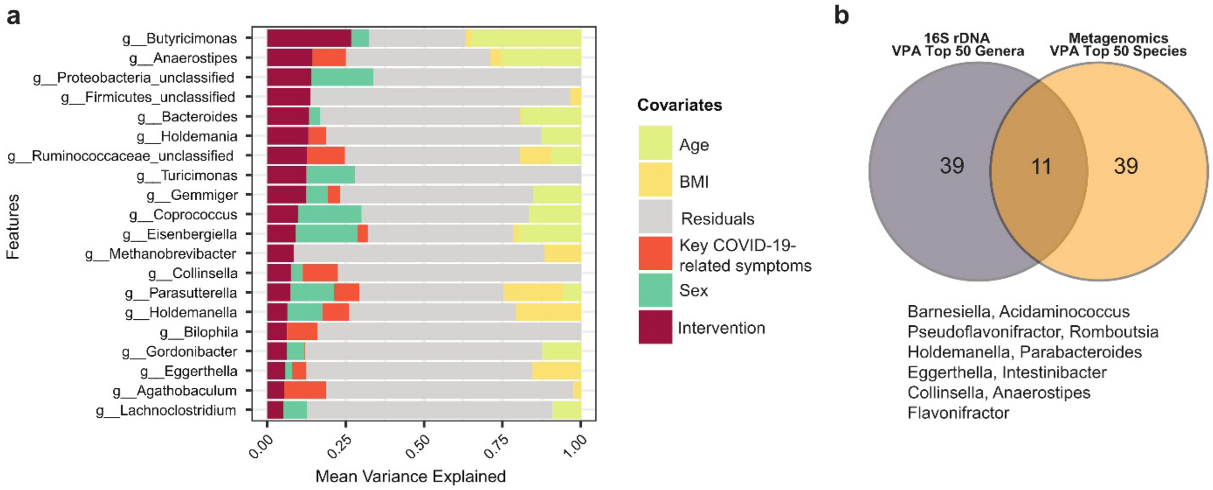

Supplementary Fig. 7 | Variance Partition Analysis (VPA) of microbial genera and comparison of 50 top genera identified in 16S rRNA gene sequencing and shotgun metagenomics.
a, VPA of the top 20 microbial genera that present most variation from the nicotinamide intervention in the shotgun metagenomics data (n=9 per intervention). The bar plot shows the mean variance explained for the top 20 microbial genera, with variance attributed to covariates including age (light green), body mass index (BMI; yellow), residuals (gray), COVID-19 symptoms (red), sex (dark green), and intervention (purple). Features are displayed at the genus level. **b**, Comparison of top 50 genera between 16S rRNA gene sequencing and shotgun metagenomics**.** The Venn diagram illustrates the overlap of the top 50 genera identified by VPA in 16S sequencing (dark gray circle) and shotgun metagenomics (orange circle). 11 genera were shared between the two technologies, including Barnesiella, Acidaminococcus, Pseudoflavonifractor, Romboutsia, Holdemanella, Parabacteroides, Eggerthella, Intestinibacter, Collinsella, Anaerostipes and Flavonifractor.

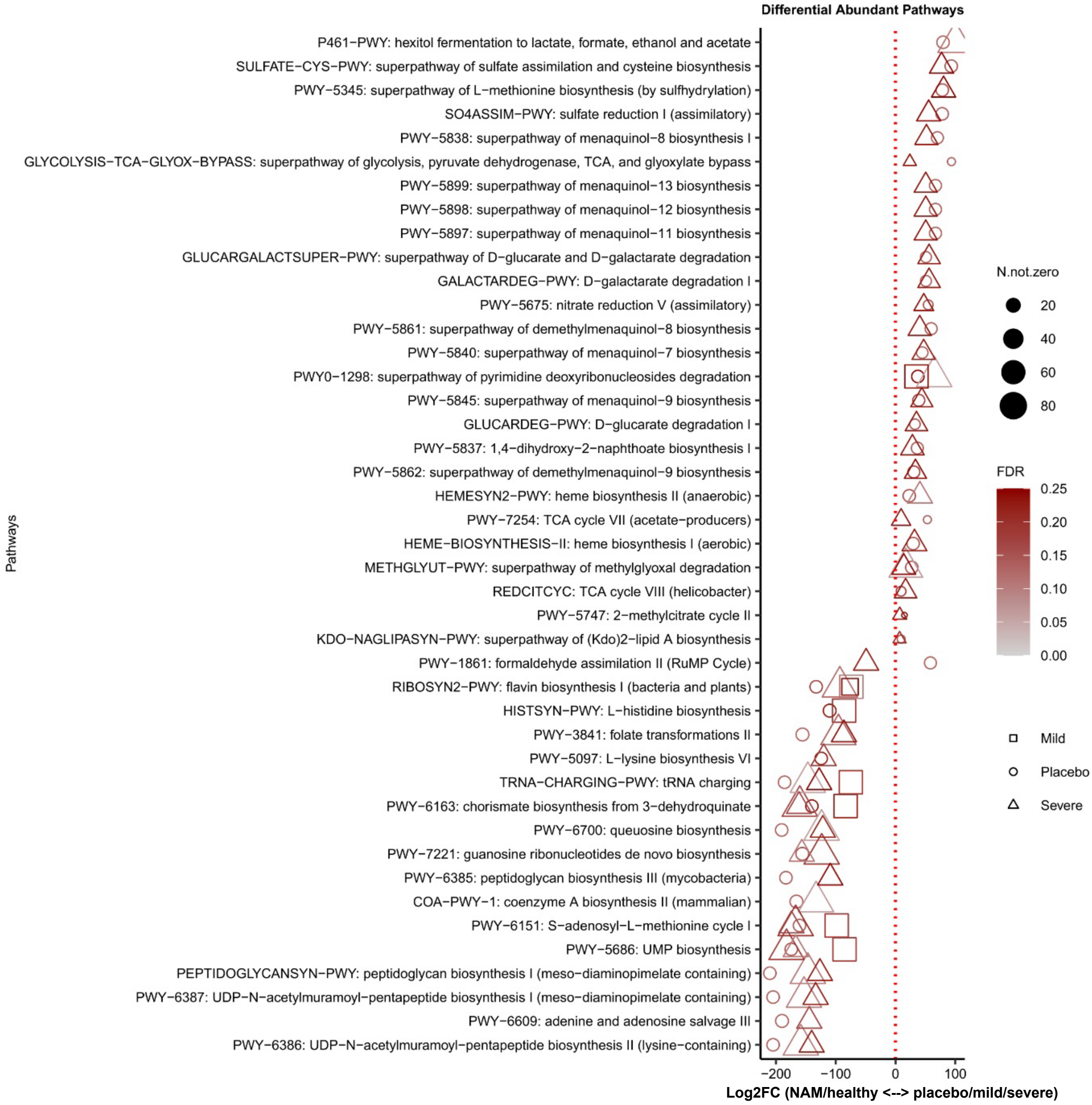

Supplementary Fig. 8 | 43 functional pathways overlap in the gut microbiota of healthy control individuals vs. patients with mild or severe COVID-19 in the public dataset from Essex et al. and nicotinamide (NAM)-receiving vs. placebo-receiving participants in COVit-2.

The dot plot represents the effect size of the differential abundant pathways between the NAM/healthy and placebo/mild/severe groups. Healthy (n=15), mild (n=15), and severe (n=8) groups were from Essex et al.[13] and NAM (n=9) and placebo (n=9) groups were from the COVit-2 trial. The x-axis represents log2 fold change (Log2FC), with negative values indicating enrichment in NAM/healthy groups and positive values indicating enrichment in placebo/mild/severe groups. Symbol size reflects the number of samples in which the pathway was detected (N.not.zero), and the false discovery rate (FDR) color scale represents the Benjamini-Hochberg-corrected P values gradient.

Supplementary Table 16: Demographic and clinical characteristics at baseline of the 16S phylogenomic subcohort (n=70).

	Levels	Nicotinamide	Placebo	Total	P
Total N (%)		35 (50.0)	35 (50.0)	70 (100.0)	
Age	Mean (SD)	41.2 (11.4)	42.4 (10.7)	41.8 (11.0)	0.66
Sex	Female	25 (71.4)	24 (68.6)	49 (70.0)	1
	Male	10 (28.6)	11 (31.4)	21 (30.0)	
Race	White	35 (100.0)	33 (94.3)	68 (97.1)	0.493
	Other	0 (0.0)	2 (5.7)	2 (2.9)	
Body mass index (BMI)	Mean (SD)	23.9 (5.9)	25.4 (5.3)	24.6 (5.6)	0.26
Age > 60 yr	Yes	2 (5.7)	1 (2.9)	3 (4.3)	1
	No	33 (94.3)	34 (97.1)	67 (95.7)	
BMI >30 and/or type 2 diabetes	Yes	5 (14.3)	7 (20.0)	12 (17.1)	0.752
	No	30 (85.7)	28 (80.0)	58 (82.9)	
Cardiovascular diseases, high blood pressure or stroke	Yes	3 (8.6)	3 (8.6)	6 (8.6)	1
	No	32 (91.4)	32 (91.4)	64 (91.4)	
Asthma, chronic obstructive pulmonary disease, or other chronic lung diseases	Yes	0 (0.0)	4 (11.4)	4 (5.7)	0.114
	No	35 (100.0)	31 (88.6)	66 (94.3)	

Fisher's exact test was used to compare variables between nicotinamide and placebo groups. No significant differences were found.

Supplementary Table 17: Demographic and clinical characteristics of the metagenomics subcohort (n=18).

	Levels	Nicotinamide	Placebo	Total	P
Total N (%)		9 (50.0)	9 (50.0)	18	
Age	Mean (SD)	37.7 (12.8)	38.6 (10.1)	38.1 (11.2)	0.872
Sex	Female	4 (44.4)	5 (55.6)	9 (50.0)	1
	Male	5 (55.6)	4 (44.4)	9 (50.0)	
Race	White	9 (100.0)	9 (100.0)	18 (100.0)	1
	Other	0 (0.0)	0 (0.0)	0 (0.0)	
Body mass index (BMI)	Mean (SD)	28.0 (8.2)	27.1 (7.0)	27.6 (7.4)	0.808
Age > 60 yr	Yes	0 (0.0)	0 (0.0)	0 (0.0)	1
	No	9 (100.0)	9 (100.0)	18 (100.0)	
BMI >30 and/or type 2 diabetes	Yes	2 (22.2)	3 (33.3)	5 (27.8)	1
	No	7 (77.8)	6 (66.7)	13 (72.2)	
Cardiovascular diseases, high blood pressure or stroke	Yes	4 (44.4)	1 (11.1)	5 (27.8)	0.294
	No	5 (55.6)	8 (88.9)	13 (72.2)	
Asthma, chronic obstructive pulmonary disease, or other chronic lung diseases	Yes	2 (22.2)	2 (22.2)	4 (22.2)	1
	No	7 (77.8)	7 (77.8)	14 (77.8)	

Fisher's exact test was used to compare variables between nicotinamide and placebo groups. No significant differences were found.

Supplementary Table 18: PERMANOVA results of the influence of age, key COVID-19-related symptoms and intervention on the gut microbial composition of the 16S phylogenomic subcohort (n=70) at week 2 and week 4.

Covariate	Df	SumOfSqs	R^2	F	P	parOmegaSq	FDR
Age	1	5730.84	0.019	3.43	0.0001	0.0133	0.0005
Key COVID-19-related symptoms	1	2065.91	0.007	1.24	0.0095	0.0013	0.0158
Intervention	2	4784.09	0.016	1.43	0.0012	0.0048	0.002
Residual	176	293762.09	0.959	NA	NA	NA	NA
Total	180	306342.93	1.000	NA	NA	NA	NA

PERMANOVA was tested with 10000 permutations.

Df, degrees of freedom; F, pseudo-F statistics value; FDR, false discovery rate; P, P value; parOmegaSq, Partial Omega Squared value; R^2, variation explained; SumOfSqs, sum of squares.

Supplementary Table 19: PERMANOVA results of the influence of age and intervention on the gut microbial composition of the 16S phylogenomic subcohort (n=70) at baseline.

Covariate	Df	SumOfSqs	R^2	F	P	parOmegaSq	FDR
Age	1	1760.5	0.024	1.08	0.1836	0.0018	0.7343
Intervention	1	1421.88	0.019	0.88	0.9187	0.0027	1
Residual	44	71484.3	0.957	NA	NA	NA	NA
Total	46	74666.67	1	NA	NA	NA	NA

PERMANOVA was tested with 10000 permutations.

Df, degrees of freedom; F, pseudo-F statistics value; FDR, false discovery rate; P, P value; R^2, variation explained; SumOfSqs, sum of squares.

Supplementary Table 20: Proportion of variation explained by each feature and confounder in the 16S phylogenomic subcohort (n=70) at weeks 2 and 4, based on 16S data.

Genus	Residuals	Age	Fever before baseline	Sex	BMI ≥ 30	Key COVID-19-related symptoms	Intervention
Genus63 | Anaerotruncus	0.769987225	0	0.109467138	0	1.75E-10	0	0.120545637
Genus188 | Frisingicoccus	0.674086754	0.127329328	0.085529187	2.36E-11	0	0	0.11305473
Genus71 | unknown	0.78692853	0.058559064	0.024222356	0.031140058	0	0	0.099149992
Genus7 | Barnesiella	0.749122481	0.11717493	0.018333896	0	1.38E-13	0.024585022	0.090783671
Genus124 | Adlercreutzia	0.653746004	0.054534943	0.012670459	0.046641635	0.146767381	0	0.085639578
Genus113 | Bifidobacterium	0.779860892	0.11790051	0.020807541	0	1.01E-12	0.000731139	0.080699918
Genus209 | Hungatella	0.653099295	0.060249946	0.164876085	0.010492298	0.030591396	1.09E-12	0.08069098
Genus162 | Acidaminococcus	0.772568702	0.041298399	4.76E-11	0.052117539	0.015927837	0.038130396	0.079957127
Genus197 | [Eubacterium]_ruminantium_group	0.84463795	3.14E-13	0.035954949	0	0.029526954	0.022827772	0.067052376
Genus105 | unknown	0.89626879	0.016918082	0.026002396	2.23E-11	6.20E-12	3.69E-10	0.060810733
Genus60 | unknown	0.791738611	0.115059016	9.03E-12	0.005272826	0	0.028570504	0.059359043
Genus196 | [Eubacterium]_xylanophilum_group	0.560067609	0.156603881	0	0.04604547	0.152227508	0.028264884	0.056790648
Genus72 | Pseudoflavonifractor	0.911050832	0.003171152	3.53E-12	0	0.031964057	0	0.05381396
Genus176 | [Eubacterium]_nodatum_group	0.764132646	0.097360039	0.090964878	4.88E-12	0	0	0.047542437
Genus202 | Howardella	0.826282588	0.032518382	4.02E-13	0.074133139	0.019561971	0	0.047503919
Genus192 | Lactococcus	0.920355606	0.010793078	3.75E-11	0	0.011930903	0.010811888	0.046108525
Genus48 | Incertae_Sedis	0.783072447	0	0.053499216	0.103229407	0	0.015828092	0.044370839
Genus190 | GCA-900066575	0.914233017	0	0	0.013561535	0	0.028947507	0.043257941
Genus98 | Oxalobacter	0.906578667	0.027607127	0.025161463	0	0	0	0.040652743
Genus131 | RF39	0.884823999	0.033860289	0.011509944	0	0.029907778	1.26E-11	0.03989799
Genus177 | Romboutsia	0.761557654	0.172920928	0.02937148	0	0	0	0.036149938
Genus215 | Lachnospiraceae_UCG-004	0.874363202	0.088576773	4.22E-10	0	0.000941472	0	0.036118553
Genus29 | [Ruminococcus]_torques_group	0.683759724	0.049102049	0.054919256	0.178545507	0	0.00011627	0.033557194
Genus150 | Holdemanella	0.95698056	0	1.77E-05	0.013086467	0	0	0.029915248
Genus54 | Faecalibacterium	0.896775553	0.058872273	2.84E-11	0	0	0.015614413	0.028737761

Genus	Residuals	Age	Fever before baseline	Sex	BMI ≥ 30	Key COVID-19-related symptoms	Intervention
Genus55 | Subdoligranulum	0.925586059	0.019203555	0.025258035	0	0	0.001254372	0.02869798
Genus111 | Victivallaceae	0.938100151	0.034084722	3.56E-11	1.04E-11	0	0	0.027815127
Genus44 | Clostridia_UCG-014	0.552718893	0.133444753	0.116428434	0.068319897	0.101385275	0	0.027702749
Genus24 | Parabacteroides	0.921353499	0.045388949	0	0.005662525	0	0	0.027595028
Genus53 | Fournierella	0.783563092	0.106201622	1.18E-11	0	0.014332756	0.075515051	0.020387479
Genus89 | Proteus	0.980840324	5.23E-18	0	0	0	0	0.019159676
Genus5 | unknown	0.879701222	0.0263693	0.042708164	0	0	0.032069345	0.01915197
Genus1 | Alistipes	0.752955646	0	0.02749562	0.185572908	0	0.01645824	0.017517586
Genus67 | Butyricicoccus	0.832089195	0.046539235	3.05E-13	0	0.043205353	0.062438813	0.015727404
Genus74 | Colidextribacter	0.951283845	0.033258188	1.04E-11	0	0	0	0.015457967
Genus122 | Eggerthella	0.694730878	0.181827377	0.108272744	0	1.87E-13	0	0.015169001
Genus179 | Intestinibacter	0.960621759	0	0.024875623	2.32E-13	0	0	0.014502618
Genus129 | Collinsella	0.792722507	0.150221576	0.039676419	0	0	0.003920782	0.013458716
Genus112 | Actinomyces	0.88829435	0	0.071061856	0.024181708	0	0.003298184	0.013163903
Genus49 | DTU089	0.929371192	0.051892611	1.06E-10	0.005749631	2.50E-12	0	0.012986566
Genus47 | Caproiciproducens	0.887844082	0	0.035442475	0.064019945	0	0	0.012693498
Genus203 | [Bacteroides]_pectinophilus_group	0.98784414	1.54E-13	0	1.82E-14	6.11E-14	0	0.01215586
Genus45 | UCG-010	0.755415525	0.142803871	0.034535048	0	0.055731791	3.05E-12	0.011513765
Genus88 | Klebsiella	0.988744896	0	4.04E-12	9.09E-12	1.84E-11	0	0.011255104
Genus14 | unknown	0.988996716	1.05E-17	0	0	0	4.09E-19	0.011003284
Genus117 | Coriobacteriaceae_UCG-003	0.852593452	0.023803801	1.24E-11	0.114512602	0	0	0.009090145
Genus199 | Anaerostipes	0.978830905	4.00E-13	0.013242307	0	0	0	0.007926788
Genus80 | Monoglobus	0.989848513	1.48E-11	0.002770011	0	1.06E-12	9.17E-14	0.007381476
Genus73 | Flavonifractor	0.826699064	0.031706062	0.107433527	0	0.026669337	0.000550757	0.006941253
Genus13 | Dysgonomonas	0.994126849	0	7.26E-11	0	0	0	0.005873151
Genus43 | Tyzzerella	0.916652513	0	0	0.05769706	0.002477042	0.017314203	0.005859183
Genus115 | unknown	0.956457044	0.034517915	0	0.003273464	0	0	0.005751577

Genus	Residuals	Age	Fever before baseline	Sex	BMI ≥ 30	Key COVID-19-related symptoms	Intervention
Genus195 | Megamonas	0.844684432	0.059843799	1.42E-13	0.081504485	0	0.008377123	0.005590161
Genus180 | Peptoclostridium	0.922253507	0.034104539	0.038100493	0	0	0	0.00554146
Genus58 | Paludicola	0.99448523	8.70E-13	0	0	0	0	0.00551477
Genus34 | Shuttleworthia	0.84782384	0.087296619	0.021882807	0.037756009	0	0	0.005240724
Genus140 | Lactobacillus	0.866030874	0.027826131	3.45E-11	0.050453071	0	0.051492275	0.004197649
Genus130 | Libanicoccus	0.816484362	0.025964468	1.60E-11	0.154586745	0	5.54E-13	0.002964425
Genus61 | Hydrogenoanaerobacterium	0.91150694	0.060725083	1.77E-13	0.002035564	0	0.022974322	0.002758091
Genus110 | Victivallis	0.998099984	0	6.03E-13	0	0	0	0.001900016
Genus145 | Asteroleplasma	0.952961848	0.045457767	1.06E-11	1.10E-10	0	0	0.001580385
Genus65 | Ruminococcus	0.881392296	0.007964947	5.57E-15	0.050794152	0.058999868	0	0.000848737
Genus178 | Terrisporobacter	0.899111136	0.080492988	0.002062106	0	0.017799709	0	0.000534061
Genus172 | Family_XIII_UCG-001	0.903608878	0	1.12E-09	0.09609108	0	2.49E-10	0.000300041
Genus23 | Bacteroides	0.851075375	0.050992231	0.061520871	2.11E-11	0	0.036411504	1.89E-08
Genus198 | [Eubacterium]_hallii_group	0.883741672	0	0.005734889	4.80E-10	0.110523429	6.57E-09	3.29E-09
Genus81 | Clostridia_vadinBB60_group	0.931930382	0.018088692	0	0.049980921	2.23E-09	0	2.71E-09
Genus97 | Comamonas	0.645286112	0.225507082	0.102481393	0	0.026725413	0	2.45E-10
Genus51 | Anaerofilum	0.963744716	0	1.26E-09	0	0	0.036255282	7.75E-11
Genus171 | Mitsuokella	0.969848737	0	0.007427583	0.02272368	6.18E-11	4.22E-12	5.45E-11
Genus109 | vadinBE97	0.944640321	0.053875615	0	0.001484063	0	0	1.47E-11
Genus185 | Defluviitaleaceae_UCG-011	0.829563994	0.147618405	0.005057433	0	0	0.017760168	1.32E-11
Genus50 | [Eubacterium]_coprostanoligenes_group	0.816883341	0.033276737	0.038726107	0.027541694	0.083572121	0	1.02E-11
Genus132 | Peptococcus	0.948578208	0	0.051421792	0	0	0	9.66E-12
Genus205 | [Eubacterium]_eligens_group	0.991605742	0.008394258	7.26E-12	0	0	0	6.01E-12
Genus159 | Catenibacterium	0.947026275	0.044529157	4.27E-11	0.008444568	0	0	1.93E-12
Genus118 | Gordonibacter	0.949226681	0.012238721	0.013940488	0.020715122	0	0.003878988	1.72E-12
Genus214 | Lachnoclostridium	0.764808821	0	0.058693627	0	0.138682108	0.037815444	1.65E-12
Genus64 | [Eubacterium]_siraeum_group	0.896499119	0.028005438	0.009740578	0.002894313	0.062860553	0	1.39E-12

Genus	Residuals	Age	Fever before baseline	Sex	BMI ≥ 30	Key COVID-19-related symptoms	Intervention
Genus103 | TM7x	0.851981154	0.04613915	0.075925005	0.02595469	0	0	8.35E-13
Genus158 | Coprobacillus	0.931250049	6.41E-12	0.02858916	0.040160791	0	0	8.27E-13
Genus154 | unknown	0.977763326	0	0.015286683	0.005913299	0	0.001036692	2.07E-13
Genus147 | Holdemania	0.844502363	0.028606969	0.107509508	5.93E-12	0	0.01938116	5.92E-14
Genus16 | Prevotella	0.990329995	0	0.009670005	0	0	0	3.21E-14
Genus201 | Eisenbergiella	0.949458137	0.023993382	0.026548481	0	0	0	2.41E-14
Genus174 | Family_XIII_AD3011_group	0.781327082	0.154854338	0.021592383	0.021799844	0	0.020426352	1.20E-14
Genus128 | Enorma	1	0	0	0	0	1.58E-18	3.92E-17
Genus2 | Rikenella	0.697951556	0.086924797	0.099043109	0.063402343	0.030498183	0.022180013	0
Genus3 | unknown	0.912397986	0.037024831	0.050577183	0	0	0	0
Genus4 | Rikenellaceae_RC9_gut_group	0.975662412	0.006209009	0.018128579	0	0	0	0
Genus6 | Coprobacter	0.999522385	0	0.000477615	0	0	1.86E-12	0
Genus8 | CAG-873	0.697951556	0.086924797	0.099043109	0.063402343	0.030498183	0.022180013	0
Genus9 | Muribaculaceae	0.973813527	0.026186473	2.14E-11	0	0	0	0
Genus10 | Sanguibacteroides	0.753907492	0.051327402	0.096373121	0.084282301	0	0.014109685	0
Genus11 | Butyricimonas	0.997402576	0	0.002597424	0	2.93E-12	1.78E-13	0
Genus12 | Odoribacter	0.970077738	3.34E-14	0.029922262	0	0	0	0
Genus15 | unknown	1	0	0	0	0	0	0
Genus17 | Prevotellaceae_NK3B31_group	0.984624852	0.015375148	0	4.10E-11	2.05E-12	8.54E-12	0
Genus18 | Prevotellaceae_UCG-001	0.697951556	0.086924797	0.099043109	0.063402343	0.030498183	0.022180013	0
Genus19 | Alloprevotella	0.968337744	0.031662256	1.89E-12	0	0	7.65E-12	0
Genus20 | Prevotellaceae_UCG-003	0.697951556	0.086924797	0.099043109	0.063402343	0.030498183	0.022180013	0
Genus21 | Paraprevotella	0.94099274	0	7.12E-12	0.011844872	0.047162388	0	0
Genus22 | Prevotellaceae_Ga6A1_group	0.697951556	0.086924797	0.099043109	0.063402343	0.030498183	0.022180013	0
Genus25 | [Ruminococcus]_gauvreauii_group	0.936543066	0.063456934	2.06E-12	0	0	0	0
Genus26 | CAG-56	1	0	0	0	0	0	0
Genus27 | Marvinbryantia	0.946527748	0.021770476	0.024489727	0.007212049	0	4.04E-15	0

Genus	Residuals	Age	Fever before baseline	Sex	BMI ≥ 30	Key COVID-19-related symptoms	Intervention
Genus28 | Fusicatenibacter	1	0	0	0	0	0	0
Genus30 | Sellimonas	0.892519815	0.076534771	0.030945413	1.17E-12	0	0	0
Genus31 | [Ruminococcus]_gnavus_group	0.934174767	0.007677177	0.036835973	0.021312083	1.42E-12	0	0
Genus32 | Agathobacter	0.995604204	0.004395796	0	4.45E-12	3.85E-12	0	0
Genus33 | Roseburia	1	0	0	0	0	0	0
Genus35 | Coprococcus	0.994482355	0.005517645	9.16E-14	0	0	0	0
Genus36 | Moryella	0.999158515	0.000841485	3.01E-11	6.12E-12	0	0	0
Genus37 | Blautia	0.766124029	0.093311856	0.129240779	0	1.17E-11	0.011323336	0
Genus38 | Dorea	0.878387392	0.119876211	0	0.001736397	0	0	0
Genus39 | unknown	1	0	0	0	0	0	0
Genus40 | [Eubacterium]_fissicatena_group	0.925921048	0.035640358	0.038438593	1.49E-10	0	0	0
Genus41 | Lachnospiraceae_UCG-003	0.962089282	0	0.034674287	0	0	0.003236431	0
Genus42 | Robinsoniella	0.697951556	0.086924797	0.099043109	0.063402343	0.030498183	0.022180013	0
Genus46 | CAG-352	0.779016982	0.050957329	0	0.012980097	0.070246311	0.086799281	0
Genus52 | UBA1819	0.894602587	0.044531321	0.060866093	0	0	0	0
Genus56 | Candidatus_Soleaferrea	0.87166525	3.09E-13	0	0.12833475	0	0	0
Genus57 | [Clostridium]_methylpentosum_group	0.935620783	0.049107532	3.80E-12	0	0.006766164	0.00850552	0
Genus59 | Negativibacillus	0.87434932	1.55E-10	0.013967311	0.017705161	0.093978207	0	0
Genus62 | Phocea	0.905076486	0.008138441	0.084266951	1.81E-13	0	0.002518121	0
Genus66 | unknown	0.989173039	0.00203169	8.99E-11	0	0	0.008795271	0
Genus68 | UCG-009	1	0	0	0	0	0	0
Genus69 | UCG-005	0.785458794	0.028822255	0.04083225	0.01930477	0.098078863	0.027503069	0
Genus70 | Intestinimonas	0.877087898	0.039789419	7.39E-14	0.017214331	0.065908352	0	0
Genus75 | Oscillibacter	0.626475449	0.04117133	0.162148517	0.170204705	0	0	0
Genus76 | Oscillospira	0.869186816	0.05478315	0	0.076030035	0	0	0
Genus77 | UCG-003	1	0	0	0	0	0	0
Genus78 | UCG-002	0.910470252	0.00273687	0.037357469	0	0.043423741	0.006011668	0

Genus	Residuals	Age	Fever before baseline	Sex	BMI ≥ 30	Key COVID-19-related symptoms	Intervention
Genus79 | NK4A214_group	0.839533662	0.043208384	0.074594353	0	0	0.0426636	0
Genus82 | Christensenellaceae_R-7_group	0.726336473	0.020391436	0.068487916	0.035549072	0.126493723	0.02274138	0
Genus83 | unknown	0.999225825	0.000774175	8.03E-13	0	0	0	0
Genus84 | Pseudomonas	0.697951556	0.086924797	0.099043109	0.063402343	0.030498183	0.022180013	0
Genus85 | Escherichia-Shigella	0.883355723	0.045227632	0.009457631	2.26E-10	0.061959014	0	0
Genus86 | Citrobacter	0.697951556	0.086924797	0.099043109	0.063402343	0.030498183	0.022180013	0
Genus87 | Enterobacter	0.657669789	0.001325401	0.010919347	3.57E-12	0.330085462	0	0
Genus90 | Morganella	0.791807982	0.054947284	0.093363166	0	6.81E-12	0.059881567	0
Genus91 | Raoultella	0.697951556	0.086924797	0.099043109	0.063402343	0.030498183	0.022180013	0
Genus92 | Hafnia-Obesumbacterium	0.766787168	0.018661819	1.72E-09	0.084143317	0.130407694	0	0
Genus93 | Succinivibrio	0.984313169	0.015686831	9.12E-12	0	4.45E-12	0	0
Genus94 | Haemophilus	0.904157171	0.084053555	3.29E-10	2.64E-09	0	0.011789271	0
Genus95 | Sutterella	0.969215381	0.02143404	4.36E-13	0	1.51E-13	0.009350579	0
Genus96 | Delftia	0.697951556	0.086924797	0.099043109	0.063402343	0.030498183	0.022180013	0
Genus99 | Parasutterella	0.93660067	0.036061114	1.31E-11	0	0	0.027338217	0
Genus100 | Acinetobacter	0.697951556	0.086924797	0.099043109	0.063402343	0.030498183	0.022180013	0
Genus101 | unknown	0.78740708	0.071155437	0.048015656	0.01473654	0.066115645	0.012569641	0
Genus102 | Elusimicrobium	0.697951556	0.086924797	0.099043109	0.063402343	0.030498183	0.022180013	0
Genus104 | Akkermansia	0.864994496	0.04016491	0	0.026118029	0.005848563	0.062874002	0
Genus106 | Bilophila	0.985420068	0.014579932	0	0	0	0	0
Genus107 | Desulfovibrio	0.705633184	0.1608615	0.102229348	0.031275968	1.27E-10	0	0
Genus108 | unknown	0.870115305	0.01801013	0.006779346	0.050165457	0.047898159	0.007031603	0
Genus114 | Gastranaerophilales	0.963257854	0	2.63E-12	0.003606099	0	0.033136047	0
Genus116 | Olsenella	1	0	0	0	0	0	0
Genus119 | unknown	1	0	0	0	0	0	0
Genus120 | unknown	0.9010027	9.56E-13	0.003120111	0.067186794	0.023872507	0.004817888	0
Genus121 | Enteroscipio	1	0	0	0	0	0	0

Genus	Residuals	Age	Fever before baseline	Sex	BMI ≥ 30	Key COVID-19-related symptoms	Intervention
Genus123 | Enterorhabdus	1	0	0	0	0	0	0
Genus125 | Senegalimassilia	0.905956775	0.084975569	2.71E-09	0	0	0.009067653	0
Genus126 | CHKCI002	0.697951556	0.086924797	0.099043109	0.063402343	0.030498183	0.022180013	0
Genus127 | Slackia	1	0	0	0	0	0	0
Genus133 | unknown	0.989965014	0	2.14E-12	0	5.19E-12	0.010034986	0
Genus134 | Anaerofustis	1	0	0	0	0	0	0
Genus135 | Cloacibacillus	0.934934224	2.72E-06	0.037919592	0	0	0.027143459	0
Genus136 | DTU014	0.939608357	0.047527467	0.012864176	0	0	1.31E-13	0
Genus137 | Enterococcus	1	0	0	0	0	0	0
Genus138 | Granulicatella	0.873935736	0.057146459	0.068917806	1.16E-11	0	0	0
Genus139 | Pediococcus	0.861543637	0.019736036	3.52E-10	0.043487427	0	0.075232899	0
Genus141 | Lysinibacillus	0.697951556	0.086924797	0.099043109	0.063402343	0.030498183	0.022180013	0
Genus142 | Turicibacter	0.857148743	0.054385571	0.079978786	0	0.0084869	0	0
Genus143 | Anaeroplasma	0.929899173	0.070100827	0	1.91E-12	0	0	0
Genus144 | Izemoplasmatales	0.955285122	0.044714878	7.85E-13	0	0	7.99E-13	0
Genus146 | Solobacterium	1	0	0	0	0	0	0
Genus148 | Dielma	0.697951556	0.086924797	0.099043109	0.063402343	0.030498183	0.022180013	0
Genus149 | Merdibacter	1	0	0	0	0	0	0
Genus151 | Faecalicoccus	0.804281511	0.137425879	0.032426273	0.017520358	0.008345979	0	0
Genus152 | Catenisphaera	0.697951556	0.086924797	0.099043109	0.063402343	0.030498183	0.022180013	0
Genus153 | Faecalitalea	0.911824422	0.040885051	3.40E-14	0	0.03942781	0.007862717	0
Genus155 | [Clostridium]_innocuum_group	0.91702618	0	0.039232004	1.21E-10	0.042977064	0.000764752	0
Genus156 | Erysipelatoclostridium	1	5.29E-18	0	0	0	8.57E-18	0
Genus157 | Erysipelotrichaceae_UCG-003	0.990621544	0	0.009378456	0	0	0	0
Genus160 | Fusobacterium	0.697951556	0.086924797	0.099043109	0.063402343	0.030498183	0.022180013	0
Genus161 | Phascolarctobacterium	0.98255711	0	0.017366573	0	6.44E-11	7.63E-05	0
Genus163 | Succiniclasticum	0.697951556	0.086924797	0.099043109	0.063402343	0.030498183	0.022180013	0

Genus	Residuals	Age	Fever before baseline	Sex	BMI ≥ 30	Key COVID-19-related symptoms	Intervention
Genus164 | Veillonella	0.998191272	0	4.35E-12	0	5.92E-11	0.001808728	0
Genus165 | Dialister	1	0	0	0	0	0	0
Genus166 | unknown	0.697951556	0.086924797	0.099043109	0.063402343	0.030498183	0.022180013	0
Genus167 | Allisonella	0.95028092	0.030566895	0.003646351	0.015505833	4.11E-13	0	0
Genus168 | Megasphaera	0.697951556	0.086924797	0.099043109	0.063402343	0.030498183	0.022180013	0
Genus169 | unknown	1	0	0	0	0	0	0
Genus170 | Anaerovibrio	0.697951556	0.086924797	0.099043109	0.063402343	0.030498183	0.022180013	0
Genus173 | [Eubacterium]_brachy_group	0.941741237	0.031337198	0.019107982	0.007813582	0	1.75E-13	0
Genus175 | Mogibacterium	0.915039964	0	0	0.084960036	4.97E-13	0	0
Genus181 | Sarcina	0.697951556	0.086924797	0.099043109	0.063402343	0.030498183	0.022180013	0
Genus182 | Clostridium_sensu_stricto_1	0.779220596	0.021905281	0.111572721	0	0.062237166	0.025064235	0
Genus183 | Epulopiscium	0.811671252	0.040104032	0.090743667	0	1.43E-13	0.057481049	0
Genus184 | Lachnospiraceae_UCG-010	0.985415086	0	3.96E-11	1.93E-11	0	0.014584914	0
Genus186 | GCA-900066755	0.92820019	0	0.07179981	0	0	6.72E-10	0
Genus187 | [Eubacterium]_ventriosum_group	0.887121541	0	0.00762595	0.105252509	0	0	0
Genus189 | Catenibacillus	0.840321188	3.37E-11	0.089435254	0	0	0.070243558	0
Genus191 | Lachnospiraceae_NK4B4_group	0.997434132	0	0.002565868	0	0	0	0
Genus193 | Streptococcus	0.892341945	0.099801758	0.001519494	3.87E-12	0	0.006336803	0
Genus194 | Lachnospiraceae_UCG-001	1	0	0	0	0	0	0
Genus200 | Lachnospiraceae_UCG-008	1	0	0	0	0	0	0
Genus204 | Lachnospiraceae_ND3007_group	1	0	0	0	0	0	0
Genus206 | Lachnospira	0.937156527	0.062843473	1.98E-12	0	0	0	0
Genus207 | CHKCI001	1	0	0	0	0	0	0
Genus208 | Butyrivibrio	0.95046196	0.022430625	7.93E-13	0	0	0.027107415	0
Genus210 | Lachnospiraceae_NK4A136_group	0.728088582	0.038405192	0	0.233506226	0	0	0
Genus211 | Lachnospiraceae_NK3A20_group	0.697951556	0.086924797	0.099043109	0.063402343	0.030498183	0.022180013	0
Genus212 | Lachnospiraceae_FCS020_group	0.834513773	0.110358344	0.004861749	0.00431221	0.014634087	0.031319837	0

Genus	Residuals	Age	Fever before baseline	Sex	BMI ≥ 30	Key COVID-19-related symptoms	Intervention
Genus213 | UC5-1-2E3	1	0	0	0	0	0	0

Supplementary Table 21: Proportion of variation explained by each feature and confounder in the metagenomics subcohort (n=18) at weeks 2 and 4, based on metagenomics data.

Genus	Residuals	Age	Sex	BMI ≥ 30	Key COVID-19-related symptoms	Intervention
g__Butyricimonas	0.30738084	0.350249027	0.057785187	0.016927827	0	0.267657118
g__Anaerostipes	0.461192624	0.253978868	0	0.033979107	0.105951007	0.144898394
g__Proteobacteria_unclassified	0.661552511	4.86E-12	0.198444193	1.02E-13	0	0.140003295
g__Firmicutes_unclassified	0.828126695	1.81E-12	0	0.034149021	0	0.137724284
g__Bacteroides	0.636868332	0.188339574	0.035813804	0.005641197	0	0.133337093
g__Holdemania	0.685279454	0.12655382	2.56E-12	9.71E-11	0.056705767	0.131460959
g__Ruminococcaceae_unclassified	0.557275475	0.09585483	0	0.0989546	0.12076597	0.127149125
g__Turicimonas	0.720913101	2.26E-10	0.154185551	3.05E-10	4.30E-10	0.124901346
g__Gemmiger	0.615485211	0.151703724	0.068848806	0.000858058	0.038824503	0.124279699
g__Coprococcus	0.533675768	0.165693907	0.20245534	1.58E-11	0	0.098174985
g__Eisenbergiella	0.462721768	0.196320524	0.197897411	0.020062346	0.032645408	0.090352543
g__Methanobrevibacter	0.800061096	1.03E-10	0	0.115777507	0	0.084161398
g__Collinsella	0.774684716	8.00E-09	0.038056503	2.01E-11	0.111823074	0.075435699
g__Parasutterella	0.461026522	0.056545139	0.139584445	0.188732143	0.080157913	0.073953836
g__Holdemanella	0.530420655	3.46E-11	0.111059459	0.208100639	0.086079585	0.064339662
g__Bilophila	0.839166444	0	0	1.89E-11	0.096910002	0.063923553
g__Gordonibacter	0.755109769	0.124525152	0.053922419	0	0.002869302	0.063573358
g__Eggerthella	0.72188593	0.003973232	0.021185177	0.150091332	0.044939751	0.057924578
g__Agathobaculum	0.789447602	3.29E-10	1.39E-11	0.022705837	0.13319415	0.05465241
g__Lachnoclostridium	0.782767928	0.089957188	0.076391683	0	0	0.050883201
g__Coprobacter	0.550099128	0.251573095	0.135793306	0.014887248	3.87E-11	0.047647223
g__Intestinimonas	0.772713485	0	0.04670935	0.020896301	0.112342894	0.047337969

Genus	Residuals	Age	Sex	BMI ≥ 30	Key COVID-19-related symptoms	Intervention
g__Barnesiella	0.503977944	0.025395476	0.332009513	0.092146078	7.21E-12	0.046470989
g__Butyrivibrio	0.541299007	0.013009399	0.084127195	0.227230348	0.088137404	0.046196647
g__Faecalicoccus	0.535091668	0.226828188	0.082111493	0.110893477	3.24E-11	0.045075174
g__Prevotella	0.816401323	0.129053716	0.01211533	3.86E-12	0	0.042429632
g__Clostridium	0.240015051	0.608872513	0.054168754	0.054541685	1.36E-10	0.042401996
g__Phascolarctobacterium	0.793611949	0.109517083	0	0.056815706	0	0.040055261
g__Flavonifractor	0.72308955	0.161511145	0.080703008	0	0	0.034696297
g__Romboutsia	0.896363965	0	0	0.069606769	1.28E-14	0.034029265
g__Olsenella	0.82116104	0.097263677	0.011872612	2.60E-12	0.039812006	0.029890665
g__Dielma	0.880525456	1.52E-12	0.070851047	0.020980309	0	0.027643188
g__Erysipelatoclostridium	0.639786609	0.113986394	0	0.219443293	0	0.026783704
g__Intestinibacter	0.592904737	0	0.055301584	0.324218086	0.006186171	0.021389421
g__Desulfovibrionaceae_unclassified	0.671764618	0.007824942	0.077334048	0.139627782	0.082416883	0.021031727
g__Cloacibacillus	0.499992519	0.356517059	0.09164589	0.030907435	2.05E-13	0.020937096
g__Enorma	0.756725516	0.079509977	0.087212184	0.005951716	0.05044432	0.020156287
g__Acidaminococcus	0.911133868	0.051385486	0	1.39E-11	0.023167049	0.014313597
g__Ruminococcus	0.735187459	0.143007345	0	0	0.110937233	0.010867964
g__Pseudoflavonifractor	0.859734708	0.052671168	0.052140661	0	0.025763972	0.009689491
g__Blastocystis	0.920043858	0.056504221	0	5.33E-11	0.016239134	0.007212787
g__Methanosphaera	0.807162572	0.179714208	0.010823911	7.30E-11	3.10E-10	0.002299308
g__Escherichia	0.774425769	0.075569372	0	0	0.148168293	0.001836565
g__Enterococcus	0.999824609	0	0	0	0	0.000175391
g__Mitsuokella	0.988293818	2.06E-11	2.58E-10	0.011706179	9.80E-10	1.65E-09
g__Sanguibacteroides	0.336257482	0.537288361	0.055910909	0.070543247	0	1.30E-09
g__Corynebacterium	0.881641152	0.118358841	5.54E-09	0	0	1.17E-09
g__Slackia	0.787018875	0.076359235	0.110101635	0	0.026520254	5.96E-11
g__Parabacteroides	0.768922729	0.222868033	0	0	0.008209239	5.85E-11

Genus	Residuals	Age	Sex	BMI ≥ 30	Key COVID-19-related symptoms	Intervention
g__Megasphaera	0.998300816	0	0	0	0.001699184	1.67E-11
g__Bifidobacterium	0.291787281	0.62260101	0	0.067589897	0.018021812	8.05E-12
g__Faecalitalea	0.552804573	0.447195427	0	0	0	7.37E-12
g__Hafnia	0.992705804	0	0	0	0.007294196	5.25E-12
g__Ruthenibacterium	0.940839807	0.021008059	0	0.038152135	0	4.42E-12
g__Lachnospira	0.88275249	0.015614688	0	0.04064253	0.060990292	3.38E-12
g__Fusicatenibacter	0.940654368	0.059345632	7.82E-12	0	0	1.60E-12
g__Actinomyces	1	0	0	0	0	0
g__Propionibacterium	0.967894073	0.014701845	1.66E-11	0.017404082	0	0
g__Adlercreutzia	1	0	0	0	0	0
g__Asaccharobacter	0.984679785	0.015320215	0	0	0	0
g__Odoribacter	1	0	0	0	0	0
g__Paraprevotella	0.91054254	0	0.035226457	2.90E-14	0.054231003	0
g__Alistipes	1	0	0	0	0	0
g__Lactobacillus	0.756391563	0.243608437	0	0	1.74E-11	0
g__Pediococcus	0.996958984	2.75E-08	0	0.003040776	2.13E-07	0
g__Leuconostoc	1	0	0	0	0	0
g__Lactococcus	0.938336168	0.02076716	0.040896671	2.51E-14	0	0
g__Streptococcus	1	0	0	0	0	0
g__Hungatella	0.824682047	0.086537591	0.030565062	0	0.058215301	0
g__Lawsonibacter	0.617854676	0.107533114	0.167083796	0.107528414	5.18E-12	0
g__Monoglobus	1	0	0	0	0	0
g__Eubacterium	0.973844521	5.27E-09	0.026155461	1.26E-08	0	0
g__Anaerotignum	1	0	0	0	0	0
g__Blautia	0.696389297	0.292617364	0	0.010993339	9.81E-11	0
g__Dorea	1	0	0	0	0	0
g__Lachnospiraceae_unclassified	0.303345671	0.316832412	0.082985263	0.037651839	0.259184815	0

**Genus**	**Residuals**	**Age**	**Sex**	**BMI ≥ 30**	**Key COVID-19-related symptoms**	**Intervention**
g__Roseburia	1	0	0	0	0	0
g__Sellimonas	1	0	0	0	0	0
g__Tyzzerella	0.994021108	4.54E-15	0	0.005978892	0	0
g__Oscillibacter	1	0	0	0	0	0
g__Clostridioides	0.967890786	0.014700076	1.66E-11	0.017409138	0	0
g__Anaeromassilibacillus	0.821508686	0.073820625	0.089101345	0	0.015569344	0
g__Anaerotruncus	0.96198999	0.03801001	0	3.52E-13	0	0
g__Faecalibacterium	0.765835044	0.170354396	0.06381056	1.02E-10	2.63E-12	0
g__Absiella	0.952576041	0.047423956	0	0	2.33E-09	0
g__Catenibacterium	0.761753166	0.027869121	0	3.28E-15	0.210377713	0
g__Coprobacillus	0.947873876	5.35E-11	0	1.90E-10	0.052126124	0
g__Massiliomicrobiota	1	0	0	0	0	0
g__Turicibacter	0.924214768	1.40E-11	0.075785232	0	5.49E-11	0
g__Allisonella	0.979419093	0.020580906	0	7.05E-10	0	0
g__Dialister	0.581353008	0.382082309	0.036564683	1.47E-12	0	0
g__Veillonella	0.580394263	0.232591945	0.003874142	0.11993764	0.06320201	0
g__Fusobacterium	1	0	0	0	0	0
g__Victivallis	1	0	0	0	0	0
g__Sutterella	0.882013904	0.008773242	0.077856624	0.004816936	0.026539294	0
g__Citrobacter	1	0	0	0	0	0
g__Enterobacter	1	0	0	0	0	0
g__Klebsiella	1	0	0	0	0	0
g__Haemophilus	0.878511108	0.116351299	0	0	0.005137593	0
g__Pseudomonas	1	0	0	0	0	0
g__Akkermansia	1	0	0	0	0	0
g__Candida	0.999223525	0.000776475	0	0	0	0
g__Saccharomyces	1	0	0	0	0	0

Supplementary Table 22: Maaslin2 output of the significantly differentially abundant pathways found in the nicotinamide vs. placebo comparisons in the metagenomics subcohort (n=18). Only significant features were found at week 2 (FDR<0.25).

Metacyc ID	Pathway name	Effect size / Beta coefficient				False Discovery Rate			
		Placebo vs. nicotinamide				Placebo vs. nicotinamide			
		Week 0	Week 2	Week 4	Week 6	Week 0	Week 2	Week 4	Week 6
AST-PWY	L-arginine degradation II (AST pathway)	-2.7	-20.4	5	-18.9	0.995	0.145	0.999	0.78
BIOTIN-BIOSYNTHESIS-PWY	biotin biosynthesis I	-3.1	-77.4	-4.3	-67.2	0.995	0.242	0.999	0.78
COA-PWY-1	coenzyme A biosynthesis II (mammalian)	20.2	165.8	21.4	111.1	0.995	0.132	0.999	0.782
COA-PWY	coenzyme A biosynthesis I	12.6	116.2	8.3	76.7	0.995	0.22	0.999	0.787
DAPLYSINESYN-PWY	L-lysine biosynthesis I	-44.3	-70	1.9	-27.1	0.995	0.175	0.999	0.852
ECASYN-PWY	enterobacterial common antigen biosynthesis	-3.1	-8.3	1.3	-8.4	0.995	0.132	0.999	0.78
ENTBACSYN-PWY	enterobactin biosynthesis	-5.2	-58.4	4.2	-34	0.995	0.132	0.999	0.78
FAO-PWY	fatty acid β-oxidation I	4.7	-96.7	10.4	-69.2	0.995	0.132	0.999	0.78
FASYN-ELONG-PWY	fatty acid elongation -- saturated	-9	-102	2.6	-88.3	0.995	0.156	0.999	0.78
FASYN-INITIAL-PWY	superpathway of fatty acid biosynthesis initiation (E. coli)	-3.1	-108.9	4.5	-93.4	0.995	0.141	0.999	0.78
FERMENTATION-PWY	mixed acid fermentation	-8.8	-57	10.9	-25	0.995	0.155	0.999	0.787
FUC-RHAMCAT-PWY	superpathway of fucose and rhamnose degradation	-4.4	-41.4	-9.9	-36	0.995	0.177	0.999	0.78
FUCCAT-PWY	fucose degradation	-6.5	-39.9	-5.9	-27.5	0.995	0.147	0.999	0.78
GALACTARDEG-PWY	D-galactarate degradation I	-2.5	-50.9	7.9	-31.3	0.995	0.132	0.999	0.78
GLUCARDEG-PWY	D-glucarate degradation I	-0.5	-32.5	3.3	-24.2	0.995	0.141	0.999	0.78
GLUCARGALACTSUPER-PWY	superpathway of D-glucarate and D-galactarate degradation	-2.5	-50.9	7.9	-31.3	0.995	0.132	0.999	0.78
GLUCOSE1PMETAB-PWY	glucose and glucose-1-phosphate degradation	-3.7	-53.6	-3.6	-26.6	0.995	0.14	0.999	0.78
GLUDEG-I-PWY	GABA shunt	-10	-47.5	-7	-33.7	0.995	0.132	0.999	0.78
GLYCOCAT-PWY	glycogen degradation I (bacterial)	-11.8	-59.4	-3.5	-29.6	0.995	0.132	0.999	0.787
GLYCOL-GLYOXDEG-PWY	superpathway of glycol metabolism and degradation	-0.2	-5.3	6.1	-2	0.995	0.132	0.999	0.782
GLYCOLYSIS-TCA-GLYOX-BYPASS	superpathway of glycolysis. pyruvate dehydrogenase. TCA. and glyoxylate bypass	3.5	-93.6	-1.3	-52.8	0.995	0.132	0.999	0.78

Metacyc ID	Pathway name	Effect size / Beta coefficient				False Discovery Rate			
		Placebo vs. nicotinamide				Placebo vs. nicotinamide			
		Week 0	Week 2	Week 4	Week 6	Week 0	Week 2	Week 4	Week 6
GLYOXYLATE-BYPASS	glyoxylate cycle	0.8	-49.1	-1.5	-23.6	0.995	0.132	0.999	0.78
HEME-BIOSYNTHESIS-II	heme biosynthesis I (aerobic)	3.5	-29.4	3.1	-19.2	0.995	0.141	0.999	0.78
HEMESYN2-PWY	heme biosynthesis II (anaerobic)	0	-23	1.6	-9.3	0.999	0.153	0.999	0.782
HEXITOLDEGSUPER-PWY	superpathway of hexitol degradation (bacteria)	-15.5	-73.1	-10.7	-37.2	0.995	0.175	0.999	0.782
HISTSYN-PWY	L-histidine biosynthesis	16.6	110.2	-10	56.1	0.995	0.228	0.999	0.808
KDO-NAGLIPASYN-PWY	superpathway of (Kdo)2-lipid A biosynthesis	-0.8	-9.4	3.4	-8.7	0.995	0.147	0.999	0.78
KETOGLUCONMET-PWY	ketogluconate metabolism	7	-43.2	7.8	-24.6	0.995	0.228	0.999	0.787
LPSSYN-PWY	superpathway of lipopolysaccharide biosynthesis	-2.5	-12.5	-2.3	-12.3	0.995	0.242	0.999	0.78
METH-ACETATE-PWY	methanogenesis from acetate	-6.1	14.4	-13.8	11.8	0.995	0.211	0.999	0.846
METHGLYUT-PWY	superpathway of methylglyoxal degradation	-6.3	-27.2	0	-9.8	0.995	0.14	0.999	0.787
ORNDEG-PWY	superpathway of ornithine degradation	-16.7	-48.9	4.6	-28.7	0.995	0.132	0.999	0.78
P105-PWY	TCA cycle IV (2-oxoglutarate decarboxylase)	4.4	-55.8	2.3	-35.9	0.995	0.132	0.999	0.78
P122-PWY	heterolactic fermentation	-1.8	-13.7	3.9	-12.5	0.995	0.132	0.999	0.782
P23-PWY	reductive TCA cycle I	-2.7	-9.5	-6.5	-2.2	0.995	0.248	0.999	0.904
P461-PWY	hexitol fermentation to lactate, formate, ethanol and acetate	-17.8	-79.5	-11.6	-38.8	0.995	0.132	0.999	0.782
PEPTIDOGLYCANSYN-PWY	peptidoglycan biosynthesis I (meso-diaminopimelate containing)	46	210.3	49	145.8	0.995	0.132	0.999	0.78
POLYAMSYN-PWY	superpathway of polyamine biosynthesis I	-6.3	-49.3	8.7	-27.2	0.995	0.14	0.999	0.782
PWY-1861	formaldehyde assimilation II (RuMP Cycle)	-13.4	-58.4	-14.6	-22.1	0.995	0.132	0.999	0.782
PWY-241	C4 photosynthetic carbon assimilation cycle. NADP-ME type	-33.1	-50	0	-24.4	0.995	0.2	0.999	0.808
PWY-2723	trehalose degradation V	-7.1	-36.2	-3.1	-16.9	0.995	0.132	0.999	0.808
PWY-3841	folate transformations II	28.5	155.6	24.2	82.2	0.995	0.132	0.999	0.787
PWY-4041	gamma-glutamyl cycle	-29.5	-68.8	-10.3	-39.4	0.995	0.14	0.999	0.78

Metacyc ID	Pathway name	Effect size / Beta coefficient				False Discovery Rate			
		Placebo vs. nicotinamide				Placebo vs. nicotinamide			
		**Week 0**	**Week 2**	**Week 4**	**Week 6**	**Week 0**	**Week 2**	**Week 4**	**Week 6**
PWY-4242	pantothenate and coenzyme A biosynthesis III	9	125.6	5.7	76.1	0.995	0.23	0.999	0.787
PWY-4702	phytate degradation I	-0.4	-57.7	-1.1	-22.9	0.995	0.132	0.999	0.782
PWY-5005	biotin biosynthesis II	1.8	12.2	3.9	-0.4	0.995	0.175	0.999	0.99
PWY-5022	4-aminobutanoate degradation V	-10.6	-45	-7.1	-33.6	0.995	0.132	0.999	0.78
PWY-5083	NAD/NADH phosphorylation and dephosphorylation	-6.9	-69.4	-6.6	-62.7	0.995	0.14	0.999	0.78
PWY-5097	L-lysine biosynthesis VI	-1.2	124.3	-4.8	71.7	0.995	0.22	0.999	0.787
PWY-5136	fatty acid beta-oxidation II (peroxisome)	5.2	-82.9	12.4	-66.1	0.995	0.132	0.999	0.78
PWY-5138	unsaturated, even numbered fatty acid beta-oxidation	-3.1	-18.6	11.4	-22.5	0.995	0.145	0.999	0.78
PWY-5173	superpathway of acetyl-CoA biosynthesis	-3	-30.5	4	-23.4	0.995	0.132	0.999	0.78
PWY-5189	tetrapyrrole biosynthesis II (from glycine)	-0.1	-25.7	5.1	-15.4	0.995	0.14	0.999	0.784
PWY-5345	superpathway of L-methionine biosynthesis (by sulfhydrylation)	-7.4	-78.2	12.5	-62.5	0.995	0.147	0.999	0.78
PWY-5384	sucrose degradation IV (sucrose phosphorylase)	-24.4	-46	-14.5	-11.5	0.995	0.171	0.999	0.846
PWY-561	superpathway of glyoxylate cycle and fatty acid degradation	3.4	-54.3	3.9	-40.6	0.995	0.132	0.999	0.78
PWY-5656	mannosylglycerate biosynthesis I	-0.9	-32.8	1	-17.7	0.995	0.132	0.999	0.78
PWY-5667	CDP-diacylglycerol biosynthesis I	11.8	112.7	-19.1	34.5	0.995	0.23	0.999	0.904
PWY-5675	nitrate reduction V (assimilatory)	-3.8	-54.7	-0.3	-30.2	0.995	0.147	0.999	0.782
PWY-5676	acetyl-CoA fermentation to butanoate II	-20.8	-51.7	-1	-24.3	0.995	0.132	0.999	0.78
PWY-5686	UMP biosynthesis	20.8	174.5	8.3	117.7	0.995	0.147	0.999	0.782
PWY-5705	allantoin degradation to glyoxylate III	-2	-8.7	2.8	-4	0.995	0.132	0.999	0.78
PWY-5723	Rubisco shunt	-0.5	-63.1	3.1	-46.2	0.995	0.132	0.999	0.78
PWY-5747	2-methylcitrate cycle II	-0.6	-14.9	1.9	-11.7	0.995	0.228	0.999	0.782
PWY-5791	1.4-dihydroxy-2-naphthoate biosynthesis II (plants)	0.5	-36.5	-1.2	-27.6	0.995	0.141	0.999	0.78

Metacyc ID	Pathway name	Effect size / Beta coefficient				False Discovery Rate			
		Placebo vs. nicotinamide				Placebo vs. nicotinamide			
		Week 0	Week 2	Week 4	Week 6	Week 0	Week 2	Week 4	Week 6
PWY-5837	1.4-dihydroxy-2-naphthoate biosynthesis I	0.5	-36.5	-1.2	-27.6	0.995	0.141	0.999	0.78
PWY-5838	superpathway of menaquinol-8 biosynthesis I	0.2	-70	-9	-57.8	0.995	0.132	0.999	0.78
PWY-5840	superpathway of menaquinol-7 biosynthesis	13.5	-44.7	11.2	-28.1	0.995	0.132	0.999	0.78
PWY-5845	superpathway of menaquinol-9 biosynthesis	12.3	-38.9	8	-34.7	0.995	0.171	0.999	0.78
PWY-5850	superpathway of menaquinol-6 biosynthesis I	7.4	-52.9	7.2	-33.3	0.995	0.132	0.999	0.78
PWY-5860	superpathway of demethylmenaquinol-6 biosynthesis I	5.9	-40.4	7.4	-25	0.995	0.132	0.999	0.78
PWY-5861	superpathway of demethylmenaquinol-8 biosynthesis	-0.3	-59.6	-6.4	-46.9	0.995	0.138	0.999	0.78
PWY-5862	superpathway of demethylmenaquinol-9 biosynthesis	9.6	-30.7	8	-26.4	0.995	0.176	0.999	0.78
PWY-5863	superpathway of phylloquinol biosynthesis	-0.9	-32.3	-2.3	-25.8	0.995	0.132	0.999	0.78
PWY-5896	superpathway of menaquinol-10 biosynthesis	7.4	-52.9	7.2	-33.3	0.995	0.132	0.999	0.78
PWY-5897	superpathway of menaquinol-11 biosynthesis	0.6	-66.9	-7.7	-55	0.995	0.132	0.999	0.78
PWY-5898	superpathway of menaquinol-12 biosynthesis	0.6	-66.9	-7.7	-55	0.995	0.132	0.999	0.78
PWY-5899	superpathway of menaquinol-13 biosynthesis	0.6	-66.9	-7.7	-55	0.995	0.132	0.999	0.78
PWY-5913	TCA cycle VI (obligate autotrophs)	-43.2	-65.9	-3.9	-29.4	0.995	0.153	0.999	0.795
PWY-5918	superpathway of heme biosynthesis from glutamate	4.1	-36.4	3.1	-23.1	0.995	0.132	0.999	0.78
PWY-5920	superpathway of heme biosynthesis from glycine	2.6	-23.3	-2.8	-16.7	0.995	0.141	0.999	0.78
PWY-5971	palmitate biosynthesis II (bacteria and plants)	5.5	-105	8.1	-78.3	0.995	0.141	0.999	0.78
PWY-5989	stearate biosynthesis II (bacteria and plants)	-10.6	-103.4	3.7	-87.5	0.995	0.141	0.999	0.78
PWY-6113	superpathway of mycolate biosynthesis	-2.3	-67	15.1	-45	0.995	0.162	0.999	0.782
PWY-6151	S-adenosyl-L-methionine cycle I	16.7	160.7	-3.2	142.8	0.995	0.14	0.999	0.78

Metacyc ID	Pathway name	Effect size / Beta coefficient				False Discovery Rate			
		Placebo vs. nicotinamide				Placebo vs. nicotinamide			
		Week 0	Week 2	Week 4	Week 6	Week 0	Week 2	Week 4	Week 6
PWY-6163	chorismate biosynthesis from 3-dehydroquinate	12.7	140	9	92.3	0.995	0.228	0.999	0.791
PWY-6282	palmitoleate biosynthesis I (from (5Z)-dodec-5-enoate)	-7	-102.8	5	-86.3	0.995	0.141	0.999	0.78
PWY-6284	superpathway of unsaturated fatty acids biosynthesis (E. coli)	-2.1	-25.9	11.6	-19.1	0.995	0.228	0.999	0.78
PWY-6285	superpathway of fatty acids biosynthesis (E. coli)	-4.7	-53	11.7	-36.9	0.995	0.14	0.999	0.78
PWY-6385	peptidoglycan biosynthesis III (mycobacteria)	37	183.4	38.8	127.4	0.995	0.132	0.999	0.782
PWY-6386	UDP-N-acetylmuramoyl-pentapeptide biosynthesis II (lysine-containing)	44.8	204.8	46.2	147.7	0.995	0.132	0.999	0.78
PWY-6387	UDP-N-acetylmuramoyl-pentapeptide biosynthesis I (meso-diaminopimelate containing)	44.7	204.8	45.7	144.2	0.995	0.132	0.999	0.78
PWY-6519	8-amino-7-oxononanoate biosynthesis I	-3.7	-87.3	0.1	-74	0.995	0.175	0.999	0.78
PWY-6531	mannitol cycle	-9.6	-43.5	-11.4	-32.1	0.995	0.14	0.999	0.78
PWY-6588	pyruvate fermentation to acetone	-1.2	-29.3	4.5	-8.9	0.995	0.132	0.999	0.78
PWY-6609	adenine and adenosine salvage III	37.3	189.9	21.8	96.4	0.995	0.132	0.999	0.787
PWY-6628	superpathway of L-phenylalanine biosynthesis	-2.4	-70.3	-6.6	-51.6	0.995	0.228	0.999	0.78
PWY-6629	superpathway of L-tryptophan biosynthesis	10.9	-103.4	-2.9	-49.2	0.995	0.132	0.999	0.78
PWY-6630	superpathway of L-tyrosine biosynthesis	-0.9	-35.4	-8.8	-14.1	0.995	0.155	0.999	0.795
PWY-6700	queuosine biosynthesis	50.3	190.4	17.6	112.9	0.995	0.132	0.999	0.78
PWY-6731	starch degradation III	-7.9	-34.1	-3.6	-17.2	0.995	0.132	0.999	0.805
PWY-6803	phosphatidylcholine acyl editing	0.8	-40	0.5	-18	0.995	0.132	0.999	0.787
PWY-6823	molybdenum cofactor biosynthesis	-0.4	-8.1	6.6	-4.3	0.995	0.179	0.999	0.782
PWY-6837	fatty acid beta-oxidation V (unsaturated. odd number. di-isomerase-dependent)	-0.8	-5.7	2.8	-9.2	0.995	0.242	0.999	0.78
PWY-6969	TCA cycle V (2-oxoglutarate-ferredoxin oxidoreductase)	7.6	-36.7	0.7	-24.1	0.995	0.246	0.999	0.782

Metacyc ID	Pathway name	Effect size / Beta coefficient				False Discovery Rate			
		Placebo vs. nicotinamide				Placebo vs. nicotinamide			
		Week 0	Week 2	Week 4	Week 6	Week 0	Week 2	Week 4	Week 6
PWY-7013	L-1.2-propanediol degradation	-13	-26.9	8.3	-12.3	0.995	0.221	0.999	0.78
PWY-7046	4-coumarate degradation (anaerobic)	-4.2	-17.8	-2.6	-8.1	0.995	0.144	0.999	0.78
PWY-7094	fatty acid salvage	1.9	-15.9	2.6	-14.2	0.995	0.132	0.999	0.78
PWY-7117	C4 photosynthetic carbon assimilation cycle. PEPCK type	-49.2	-64.8	-9.9	-31.1	0.995	0.177	0.999	0.793
PWY-7199	pyrimidine deoxyribonucleosides salvage	13.5	111.1	-1.2	51.6	0.995	0.132	0.999	0.846
PWY-7204	pyridoxal 5'-phosphate salvage II (plants)	-2.8	-24.2	2.8	-16.7	0.995	0.14	0.999	0.78
PWY-7219	adenosine ribonucleotides de novo biosynthesis	18.9	183.2	28.2	109.6	0.995	0.132	0.999	0.782
PWY-7221	guanosine ribonucleotides de novo biosynthesis	5.6	156	9.2	101.8	0.995	0.147	0.999	0.787
PWY-7254	TCA cycle VII (acetate-producers)	8	-53.3	5.5	-28.4	0.995	0.132	0.999	0.78
PWY-7328	superpathway of UDP-glucose-derived O-antigen building blocks biosynthesis	-5.6	-37.6	-3.2	-18.5	0.995	0.132	0.999	0.787
PWY-7388	octanoyl-[acyl-carrier protein] biosynthesis (mitochondria. yeast)	-5.8	-108.4	4.9	-93.4	0.995	0.144	0.999	0.78
PWY-7664	oleate biosynthesis IV (anaerobic)	-8.9	-101.9	3.4	-86.3	0.995	0.147	0.999	0.78
PWY-821	superpathway of sulfur amino acid biosynthesis (Saccharomyces cerevisiae)	-11.2	-43.8	2.2	-27.4	0.995	0.132	0.999	0.78
PWY0-1298	superpathway of pyrimidine deoxyribonucleosides degradation	-2.5	-37.4	8.7	-31	0.995	0.248	0.999	0.78
PWY0-1319	CDP-diacylglycerol biosynthesis II	11.8	112.7	-19.1	34.5	0.995	0.23	0.999	0.904
PWY0-1415	superpathway of heme biosynthesis from uroporphyrinogen-III	3.4	-27	0.4	-14.4	0.995	0.132	0.999	0.78
PWY0-1533	methylphosphonate degradation I	-1.7	-2.6	0.3	-4.7	0.995	0.242	0.999	0.782
PWY0-42	2-methylcitrate cycle I	-0.6	-15.6	2.3	-11.5	0.995	0.21	0.999	0.78
PWY0-862	(5Z)-dodec-5-enoate biosynthesis	-8.8	-101.1	4.6	-85.6	0.995	0.141	0.999	0.78

Metacyc ID	Pathway name	Effect size / Beta coefficient				False Discovery Rate			
		Placebo vs. nicotinamide				Placebo vs. nicotinamide			
		Week 0	Week 2	Week 4	Week 6	Week 0	Week 2	Week 4	Week 6
PWY3O-355	stearate biosynthesis III (fungi)	-2.7	-13.2	0.7	-9.4	0.995	0.14	0.999	0.78
PWY66-391	fatty acid beta-oxidation VI (peroxisome)	2	-13	-0.1	-16.6	0.995	0.248	0.999	0.782
PWYG-321	mycolate biosynthesis	-10.1	-106.4	2.7	-97.7	0.995	0.147	0.999	0.78
PYRIDNUCSAL-PWY	NAD salvage pathway I	-25.5	-73.1	-1.4	-33.9	0.995	0.141	0.999	0.787
REDCITCYC	TCA cycle VIII (helicobacter)	2	-9.3	-4.6	-7	0.995	0.175	0.999	0.78
RIBOSYN2-PWY	flavin biosynthesis I (bacteria and plants)	33.7	132.8	-2.8	75.4	0.995	0.167	0.999	0.787
SO4ASSIM-PWY	sulfate reduction I (assimilatory)	-8.9	-78.1	17.9	-57.1	0.995	0.14	0.999	0.78
SULFATE-CYS-PWY	superpathway of sulfate assimilation and cysteine biosynthesis	-7.1	-93.3	15.1	-73.7	0.995	0.14	0.999	0.78
TCA-GLYOX-BYPASS	superpathway of glyoxylate bypass and TCA	-1.3	-68.4	0.2	-39.2	0.995	0.132	0.999	0.78
TRNA-CHARGING-PWY	tRNA charging	44.3	185.6	57.2	130.9	0.995	0.132	0.999	0.78
ARG+POLYAMINE-SYN	superpathway of arginine and polyamine biosynthesis	-5.3	-62.8	12.7	-36.2	0.995	0.147	0.999	0.782
1CMET2-PWY	*N*-formyl-tetrahydrofolate biosynthesis	30.8	111.5	-1.8	51.2	0.995	0.195	0.999	0.846

Supplementary Table 23: Maaslin2 output of overlapping pathways between the Essex et al. cohort (n=38) and the COVit-2 metagenomics subcohort (n=18).

Metacyc ID	Pathway Name	Metadata	value	coef	stderr	pval	name	qval	N	N.not.zero	model
PWY0-1298	PWY0-1298: superpathway of pyrimidine deoxyribonucleosides degradation	Intervention	Placebo	37.4099348	16.5001362	0.04186536	InterventionPLB	0.24755878	18	17	NAMvsPLB_W2
METHGLYUT-PWY	METHGLYUT-PWY: superpathway of methylglyoxal degradation	OSCI_Class	Severe	13.8658885	5.28596317	0.01349321	OSCI_ClassSevere	0.24478599	38	34	CtrlvsOSCI_Class_Visit1
PWY-6385	PWY-6385: peptidoglycan biosynthesis III (mycobacteria)	OSCI_Class	Severe	-109.19865	41.6363584	0.01335622	OSCI_ClassSevere	0.24477325	38	38	CtrlvsOSCI_Class_Visit1
PWY-5345	PWY-5345: superpathway of L-methionine biosynthesis (by sulfhydrylation)	OSCI_Class	Severe	80.4284709	30.4813101	0.01276326	OSCI_ClassSevere	0.24403195	38	35	CtrlvsOSCI_Class_Visit1
SO4ASSIM-PWY	SO4ASSIM-PWY: sulfate reduction I (assimilatory)	OSCI_Class	Severe	55.4883109	20.9663651	0.01251874	OSCI_ClassSevere	0.24403195	38	35	CtrlvsOSCI_Class_Visit1
SULFATE-CYS-PWY	SULFATE-CYS-PWY: superpathway of sulfate assimilation and cysteine biosynthesis	OSCI_Class	Severe	77.0732927	29.2732912	0.01300097	OSCI_ClassSevere	0.24403195	38	35	CtrlvsOSCI_Class_Visit1
TRNA-CHARGING-PWY	TRNA-CHARGING-PWY: tRNA charging	OSCI_Class	Severe	-127.7557	48.6714578	0.0131799	OSCI_ClassSevere	0.24403195	38	38	CtrlvsOSCI_Class_Visit1
PWY-5862	PWY-5862: superpathway of demethylmenaquinol-9 biosynthesis	OSCI_Class	Severe	33.3786446	12.5352202	0.01202663	OSCI_ClassSevere	0.2431695	38	29	CtrlvsOSCI_Class_Visit1
PWY-3841	PWY-3841: folate transformations II	OSCI_Class	Severe	-86.313336	31.9223219	0.01097873	OSCI_ClassSevere	0.23474927	38	38	CtrlvsOSCI_Class_Visit1
PWY-5675	PWY-5675: nitrate reduction V (assimilatory)	OSCI_Class	Severe	47.3074943	17.4865551	0.01091362	OSCI_ClassSevere	0.23474927	38	23	CtrlvsOSCI_Class_Visit1
RIBOSYN2-PWY	RIBOSYN2-PWY: flavin biosynthesis I (bacteria and plants)	OSCI_Class	Mild	-76.075471	28.0410439	0.01064365	OSCI_ClassMild	0.23474927	38	38	CtrlvsOSCI_Class_Visit1
PWY0-1298	PWY0-1298: superpathway of pyrimidine deoxyribonucleosides degradation	OSCI_Class	Mild	34.966509	13.0070595	0.00977496	OSCI_ClassMild	0.2343015	81	81	Longitudinal
GALACTARDEG-PWY	GALACTARDEG-PWY: D-galactarate degradation I	OSCI_Class	Severe	56.1704457	19.1040268	0.00605283	OSCI_ClassSevere	0.23059394	38	30	CtrlvsOSCI_Class_Visit1
GLUCARDEG-PWY	GLUCARDEG-PWY: D-glucarate degradation I	OSCI_Class	Severe	35.049727	12.180839	0.00715019	OSCI_ClassSevere	0.23059394	38	30	CtrlvsOSCI_Class_Visit1
GLUCARGALACTSUPER-PWY	GLUCARGALACTSUPER-PWY: superpathway of D-glucarate and D-galactarate degradation	OSCI_Class	Severe	56.1704457	19.1040268	0.00605283	OSCI_ClassSevere	0.23059394	38	30	CtrlvsOSCI_Class_Visit1
GLYCOLYSIS-TCA-GLYOX-BYPASS	GLYCOLYSIS-TCA-GLYOX-BYPASS: superpathway of glycolysis. pyruvate dehydrogenase. TCA. and glyoxylate bypass	OSCI_Class	Severe	23.9617966	8.67747713	0.00953505	OSCI_ClassSevere	0.23059394	38	12	CtrlvsOSCI_Class_Visit1
PWY-5747	PWY-5747: 2-methylcitrate cycle II	OSCI_Class	Severe	7.03222378	2.4531806	0.00728244	OSCI_ClassSevere	0.23059394	38	13	CtrlvsOSCI_Class_Visit1
PWY-5837	PWY-5837: 1.4-dihydroxy-2-naphthoate biosynthesis I	OSCI_Class	Severe	28.3332032	10.3734966	0.01017749	OSCI_ClassSevere	0.23059394	38	33	CtrlvsOSCI_Class_Visit1
PWY-5838	PWY-5838: superpathway of menaquinol-8 biosynthesis I	OSCI_Class	Severe	51.7483401	18.2192064	0.00777294	OSCI_ClassSevere	0.23059394	38	32	CtrlvsOSCI_Class_Visit1
PWY-5845	PWY-5845: superpathway of menaquinol-9 biosynthesis	OSCI_Class	Severe	44.0044157	15.9779145	0.00962409	OSCI_ClassSevere	0.23059394	38	29	CtrlvsOSCI_Class_Visit1

Metacyc ID	Pathway Name	Metadata	value	coef	stderr	pval	name	qval	N	N.not.zero	model
PWY-5861	PWY-5861: superpathway of demethylmenaquinol-8 biosynthesis	OSCI_Class	Severe	40.497192	14.6560182	0.00941087	OSCI_ClassSevere	0.23059394	38	32	CtrlvsOSCI_Class_Visit1
PWY-5897	PWY-5897: superpathway of menaquinol-11 biosynthesis	OSCI_Class	Severe	50.5862896	17.8387885	0.00786203	OSCI_ClassSevere	0.23059394	38	32	CtrlvsOSCI_Class_Visit1
PWY-5898	PWY-5898: superpathway of menaquinol-12 biosynthesis	OSCI_Class	Severe	50.5862896	17.8387885	0.00786203	OSCI_ClassSevere	0.23059394	38	32	CtrlvsOSCI_Class_Visit1
PWY-5899	PWY-5899: superpathway of menaquinol-13 biosynthesis	OSCI_Class	Severe	50.5862896	17.8387885	0.00786203	OSCI_ClassSevere	0.23059394	38	32	CtrlvsOSCI_Class_Visit1
PWY-7254	PWY-7254: TCA cycle VII (acetate-producers)	OSCI_Class	Severe	9.33241814	3.38281607	0.01015915	OSCI_ClassSevere	0.23059394	38	22	CtrlvsOSCI_Class_Visit1
REDCITCYC	REDCITCYC: TCA cycle VIII (helicobacter)	OSCI_Class	Severe	16.8746978	5.72216538	0.0063237	OSCI_ClassSevere	0.23059394	38	30	CtrlvsOSCI_Class_Visit1
HISTSYN-PWY	HISTSYN-PWY: L-histidine biosynthesis	Intervention	Placebo	-110.18472	46.643097	0.03567061	InterventionPLB	0.22829187	18	18	NAMvsPLB_W2
PWY-5747	PWY-5747: 2-methylcitrate cycle II	Intervention	Placebo	14.8989671	6.28389986	0.03451748	InterventionPLB	0.2281287	18	9	NAMvsPLB_W2
PWY-6163	PWY-6163: chorismate biosynthesis from 3-dehydroquinate	Intervention	Placebo	-139.99202	59.4614654	0.03492687	InterventionPLB	0.2281287	18	17	NAMvsPLB_W2
HEME-BIOSYNTHESIS-II	HEME-BIOSYNTHESIS-II: heme biosynthesis I (aerobic)	OSCI_Class	Severe	31.7228249	10.065858	0.00351443	OSCI_ClassSevere	0.22392838	38	37	CtrlvsOSCI_Class_Visit1
PEPTIDOGLYCANSYN-PWY	PEPTIDOGLYCANSYN-PWY: peptidoglycan biosynthesis I (meso-diaminopimelate containing)	OSCI_Class	Severe	-126.51023	42.3095803	0.00538255	OSCI_ClassSevere	0.22392838	38	38	CtrlvsOSCI_Class_Visit1
PWY-1861	PWY-1861: formaldehyde assimilation II (RuMP Cycle)	OSCI_Class	Severe	-49.210176	16.1113202	0.00451898	OSCI_ClassSevere	0.22392838	38	38	CtrlvsOSCI_Class_Visit1
PWY-5840	PWY-5840: superpathway of menaquinol-7 biosynthesis	OSCI_Class	Severe	47.118169	15.7129105	0.00521761	OSCI_ClassSevere	0.22392838	38	32	CtrlvsOSCI_Class_Visit1
PWY-6386	PWY-6386: UDP-N-acetylmuramoyl-pentapeptide biosynthesis II (lysine-containing)	OSCI_Class	Severe	-140.30826	46.3484817	0.004876	OSCI_ClassSevere	0.22392838	38	38	CtrlvsOSCI_Class_Visit1
PWY-6387	PWY-6387: UDP-N-acetylmuramoyl-pentapeptide biosynthesis I (meso-diaminopimelate containing)	OSCI_Class	Severe	-133.67513	44.7162142	0.00537206	OSCI_ClassSevere	0.22392838	38	38	CtrlvsOSCI_Class_Visit1
PWY-5097	PWY-5097: L-lysine biosynthesis VI	Intervention	Placebo	-124.33038	52.77046	0.032493	InterventionPLB	0.22029149	18	17	NAMvsPLB_W2
PWY-6700	PWY-6700: queuosine biosynthesis	OSCI_Class	Severe	-121.62666	37.9507924	0.00317492	OSCI_ClassSevere	0.21119095	38	38	CtrlvsOSCI_Class_Visit1
PWY-6151	PWY-6151: S-adenosyl-L-methionine cycle I	OSCI_Class	Severe	-167.09189	48.9554323	0.00723467	OSCI_ClassSevere	0.20748352	81	81	Longitudinal
HISTSYN-PWY	HISTSYN-PWY: L-histidine biosynthesis	OSCI_Class	Mild	-85.997104	30.52032	0.00709674	OSCI_ClassMild	0.20691198	81	81	Longitudinal
PWY-6163	PWY-6163: chorismate biosynthesis from 3-dehydroquinate	OSCI_Class	Mild	-83.459458	29.3668035	0.00700081	OSCI_ClassMild	0.20633377	81	81	Longitudinal
TRNA-CHARGING-PWY	TRNA-CHARGING-PWY: tRNA charging	OSCI_Class	Mild	-74.659177	26.7237321	0.00691649	OSCI_ClassMild	0.20608873	81	81	Longitudinal

Metacyc ID	Pathway Name	Metadata	value	coef	stderr	pval	name	qval	N	N.not.zero	model
KDO-NAGLIPASYN-PWY	KDO-NAGLIPASYN-PWY: superpathway of (Kdo)2-lipid A biosynthesis	OSCI_Class	Severe	6.85139742	2.12431062	0.00289818	OSCI_ClassSevere	0.20019726	38	12	CtrlvsOSCI_Class_Visit1
PWY-6151	PWY-6151: S-adenosyl-L-methionine cycle I	OSCI_Class	Mild	-98.415087	34.4756118	0.00617243	OSCI_ClassMild	0.18923586	81	81	Longitudinal
PWY-5097	PWY-5097: L-lysine biosynthesis VI	OSCI_Class	Severe	-120.20356	36.8193294	0.00261235	OSCI_ClassSevere	0.18767122	38	38	CtrlvsOSCI_Class_Visit1
PWY-7221	PWY-7221: guanosine ribonucleotides de novo biosynthesis	OSCI_Class	Severe	-123.52293	40.7050094	0.00587824	OSCI_ClassSevere	0.18641915	81	81	Longitudinal
PWY-5686	PWY-5686: UMP biosynthesis	OSCI_Class	Severe	-182.36579	40.1441212	0.00560823	OSCI_ClassSevere	0.17996102	81	81	Longitudinal
PWY-5862	PWY-5862: superpathway of demethylmenaquinol-9 biosynthesis	Intervention	Placebo	30.6504653	11.9056594	0.02391452	InterventionPLB	0.17551941	18	16	NAMvsPLB_W2
PWY-5686	PWY-5686: UMP biosynthesis	OSCI_Class	Mild	-85.296629	29.0286723	0.00513939	OSCI_ClassMild	0.17491429	81	81	Longitudinal
REDCITCYC	REDCITCYC: TCA cycle VIII (helicobacter)	Intervention	Placebo	9.32562113	3.69097672	0.02325092	InterventionPLB	0.17464776	18	12	NAMvsPLB_W2
PWY-5845	PWY-5845: superpathway of menaquinol-9 biosynthesis	Intervention	Placebo	38.8863992	14.8597031	0.02206677	InterventionPLB	0.17139239	18	16	NAMvsPLB_W2
PWY-6163	PWY-6163: chorismate biosynthesis from 3-dehydroquinate	OSCI_Class	Severe	-160.13387	44.1169067	0.00453931	OSCI_ClassSevere	0.16746044	81	81	Longitudinal
RIBOSYN2-PWY	RIBOSYN2-PWY: flavin biosynthesis I (bacteria and plants)	Intervention	Placebo	-132.7633	51.5491279	0.02110374	InterventionPLB	0.16715832	18	17	NAMvsPLB_W2
PWY-6151	PWY-6151: S-adenosyl-L-methionine cycle I	OSCI_Class	Severe	-174.09261	51.0092188	0.00176305	OSCI_ClassSevere	0.16588355	38	38	CtrlvsOSCI_Class_Visit1
PWY-6163	PWY-6163: chorismate biosynthesis from 3-dehydroquinate	OSCI_Class	Severe	-161.39829	47.0920648	0.00174696	OSCI_ClassSevere	0.16588355	38	38	CtrlvsOSCI_Class_Visit1
PWY-6609	PWY-6609: adenine and adenosine salvage III	OSCI_Class	Severe	-144.29214	42.8666343	0.00205883	OSCI_ClassSevere	0.16588355	38	38	CtrlvsOSCI_Class_Visit1
HEMESYN2-PWY	HEMESYN2-PWY: heme biosynthesis II (anaerobic)	Intervention	Placebo	23.0241131	8.4873752	0.0183393	InterventionPLB	0.15282749	18	17	NAMvsPLB_W2
KDO-NAGLIPASYN-PWY	KDO-NAGLIPASYN-PWY: superpathway of (Kdo)2-lipid A biosynthesis	Intervention	Placebo	9.40481141	3.40724048	0.01690677	InterventionPLB	0.1470868	18	10	NAMvsPLB_W2
PWY-5345	PWY-5345: superpathway of L-methionine biosynthesis (by sulfhydrylation)	Intervention	Placebo	78.2412898	28.2849817	0.0166289	InterventionPLB	0.1470868	18	17	NAMvsPLB_W2
PWY-5675	PWY-5675: nitrate reduction V (assimilatory)	Intervention	Placebo	54.7066298	19.7253425	0.01640906	InterventionPLB	0.1470868	18	12	NAMvsPLB_W2
PWY-5686	PWY-5686: UMP biosynthesis	Intervention	Placebo	-174.47422	64.4880043	0.01627726	InterventionPLB	0.1470868	18	17	NAMvsPLB_W2
PWY-7221	PWY-7221: guanosine ribonucleotides de novo biosynthesis	Intervention	Placebo	-155.97834	58.094458	0.01696418	InterventionPLB	0.1470868	18	18	NAMvsPLB_W2
GLUCARDEG-PWY	GLUCARDEG-PWY: D-glucarate degradation I	Intervention	Placebo	32.5348383	11.3651748	0.0139434	InterventionPLB	0.1411257	18	13	NAMvsPLB_W2
HEME-BIOSYNTHESIS-II	HEME-BIOSYNTHESIS-II: heme biosynthesis I (aerobic)	Intervention	Placebo	29.4235404	10.2367076	0.01361252	InterventionPLB	0.1411257	18	17	NAMvsPLB_W2

Metacyc ID	Pathway Name	Metadata	value	coef	stderr	pval	name	qval	N	N.not.zero	model
PWY-5837	PWY-5837: 1.4-dihydroxy-2-naphthoate biosynthesis I	Intervention	Placebo	36.4887406	12.8063722	0.01428898	InterventionPLB	0.1411257	18	16	NAMvsPLB_W2
PYRIDNUCSAL-PWY	PYRIDNUCSAL-PWY: NAD salvage pathway I	Intervention	Placebo	73.144394	26.219424	0.01374221	InterventionPLB	0.1411257	18	17	NAMvsPLB_W2
METHGLYUT-PWY	METHGLYUT-PWY: superpathway of methylglyoxal degradation	Intervention	Placebo	27.246507	9.27733463	0.01207246	InterventionPLB	0.13962377	18	17	NAMvsPLB_W2
PWY-6151	PWY-6151: S-adenosyl-L-methionine cycle I	Intervention	Placebo	-160.66872	55.2736857	0.01084442	InterventionPLB	0.13962377	18	17	NAMvsPLB_W2
SO4ASSIM-PWY	SO4ASSIM-PWY: sulfate reduction I (assimilatory)	Intervention	Placebo	78.0781031	26.0761247	0.01075156	InterventionPLB	0.13962377	18	17	NAMvsPLB_W2
SULFATE-CYS-PWY	SULFATE-CYS-PWY: superpathway of sulfate assimilation and cysteine biosynthesis	Intervention	Placebo	93.2602457	31.2324564	0.01091073	InterventionPLB	0.13962377	18	17	NAMvsPLB_W2
PWY-3841	PWY-3841: folate transformations II	OSCI_Class	Severe	-95.534361	25.3503453	0.00335088	OSCI_ClassSevere	0.13766514	81	81	Longitudinal
PWY-5861	PWY-5861: superpathway of demethylmenaquinol-8 biosynthesis	Intervention	Placebo	59.6472691	19.5702179	0.00980529	InterventionPLB	0.13761805	18	16	NAMvsPLB_W2
COA-PWY-1	COA-PWY-1: coenzyme A biosynthesis II (mammalian)	Intervention	Placebo	-165.78645	53.9111621	0.00769766	InterventionPLB	0.1321481	18	18	NAMvsPLB_W2
GALACTARDEG-PWY	GALACTARDEG-PWY: D-galactarate degradation I	Intervention	Placebo	50.8573341	15.3857294	0.00610934	InterventionPLB	0.1321481	18	14	NAMvsPLB_W2
GLUCARGALACTSUPER-PWY	GLUCARGALACTSUPER-PWY: superpathway of D-glucarate and D-galactarate degradation	Intervention	Placebo	50.8573341	15.3857294	0.00610934	InterventionPLB	0.1321481	18	14	NAMvsPLB_W2
GLYCOLYSIS-TCA-GLYOX-BYPASS	GLYCOLYSIS-TCA-GLYOX-BYPASS: superpathway of glycolysis. pyruvate dehydrogenase. TCA. and glyoxylate bypass	Intervention	Placebo	93.58483	27.4113497	0.00485441	InterventionPLB	0.1321481	18	10	NAMvsPLB_W2
P461-PWY	P461-PWY: hexitol fermentation to lactate, formate, ethanol and acetate	Intervention	Placebo	79.5284894	23.5169072	0.00516443	InterventionPLB	0.1321481	18	17	NAMvsPLB_W2
PEPTIDOGLYCANSYN-PWY	PEPTIDOGLYCANSYN-PWY: peptidoglycan biosynthesis I (meso-diaminopimelate containing)	Intervention	Placebo	-210.26714	53.1362643	0.00126501	InterventionPLB	0.1321481	18	17	NAMvsPLB_W2
PWY-1861	PWY-1861: formaldehyde assimilation II (RuMP Cycle)	Intervention	Placebo	58.4274854	17.6403696	0.00558204	InterventionPLB	0.1321481	18	16	NAMvsPLB_W2
PWY-3841	PWY-3841: folate transformations II	Intervention	Placebo	-155.64632	45.3162395	0.003686	InterventionPLB	0.1321481	18	18	NAMvsPLB_W2
PWY-5838	PWY-5838: superpathway of menaquinol-8 biosynthesis I	Intervention	Placebo	70.0437239	22.679501	0.00904325	InterventionPLB	0.1321481	18	16	NAMvsPLB_W2
PWY-5840	PWY-5840: superpathway of menaquinol-7 biosynthesis	Intervention	Placebo	44.6758256	14.5329233	0.00925037	InterventionPLB	0.1321481	18	15	NAMvsPLB_W2
PWY-5897	PWY-5897: superpathway of menaquinol-11 biosynthesis	Intervention	Placebo	66.8765235	21.6941825	0.00914145	InterventionPLB	0.1321481	18	16	NAMvsPLB_W2
PWY-5898	PWY-5898: superpathway of menaquinol-12 biosynthesis	Intervention	Placebo	66.8765235	21.6941825	0.00914145	InterventionPLB	0.1321481	18	16	NAMvsPLB_W2
PWY-5899	PWY-5899: superpathway of menaquinol-13 biosynthesis	Intervention	Placebo	66.8765235	21.6941825	0.00914145	InterventionPLB	0.1321481	18	16	NAMvsPLB_W2

Metacyc ID	Pathway Name	Metadata	value	coef	stderr	pval	name	qval	N	N.not.zero	model
PWY-6385	PWY-6385: peptidoglycan biosynthesis III (mycobacteria)	Intervention	Placebo	-183.38051	55.1606787	0.00462102	InterventionPLB	0.1321481	18	17	NAMvsPLB_W2
PWY-6386	PWY-6386: UDP-N-acetylmuramoyl-pentapeptide biosynthesis II (lysine-containing)	Intervention	Placebo	-204.84427	54.7119214	0.0019546	InterventionPLB	0.1321481	18	17	NAMvsPLB_W2
PWY-6387	PWY-6387: UDP-N-acetylmuramoyl-pentapeptide biosynthesis I (meso-diaminopimelate containing)	Intervention	Placebo	-204.81418	53.8723519	0.00173668	InterventionPLB	0.1321481	18	17	NAMvsPLB_W2
PWY-6609	PWY-6609: adenine and adenosine salvage III	Intervention	Placebo	-189.90436	48.1064036	0.00128983	InterventionPLB	0.1321481	18	18	NAMvsPLB_W2
PWY-6700	PWY-6700: queuosine biosynthesis	Intervention	Placebo	-190.44389	52.8328035	0.00260073	InterventionPLB	0.1321481	18	17	NAMvsPLB_W2
PWY-7254	PWY-7254: TCA cycle VII (acetate-producers)	Intervention	Placebo	53.3077718	15.0818748	0.0039111	InterventionPLB	0.1321481	18	10	NAMvsPLB_W2
TRNA-CHARGING-PWY	TRNA-CHARGING-PWY: tRNA charging	Intervention	Placebo	-185.62854	53.3461342	0.00336065	InterventionPLB	0.1321481	18	17	NAMvsPLB_W2
RIBOSYN2-PWY	RIBOSYN2-PWY: flavin biosynthesis I (bacteria and plants)	OSCI_Class	Severe	-93.198681	28.8209267	0.00270549	OSCI_ClassSevere	0.11764008	81	81	Longitudinal
P461-PWY	P461-PWY: hexitol fermentation to lactate, formate, ethanol and acetate	OSCI_Class	Severe	100.361386	30.8078532	0.00231188	OSCI_ClassSevere	0.10808037	81	81	Longitudinal
HEMESYN2-PWY	HEMESYN2-PWY: heme biosynthesis II (anaerobic)	OSCI_Class	Severe	40.7776722	10.4873294	0.00049377	OSCI_ClassSevere	0.10619678	38	38	CtrlvsOSCI_Class_Visit1
PWY-5686	PWY-5686: UMP biosynthesis	OSCI_Class	Severe	-166.88799	44.8950782	0.00077193	OSCI_ClassSevere	0.10619678	38	38	CtrlvsOSCI_Class_Visit1
PWY-7221	PWY-7221: guanosine ribonucleotides de novo biosynthesis	OSCI_Class	Severe	-156.79178	37.9834431	0.0002486	OSCI_ClassSevere	0.10619678	38	38	CtrlvsOSCI_Class_Visit1
PEPTIDOGLYCANSYN-PWY	PEPTIDOGLYCANSYN-PWY: peptidoglycan biosynthesis I (meso-diaminopimelate containing)	OSCI_Class	Severe	-146.69463	37.3505936	0.00225046	OSCI_ClassSevere	0.10612364	81	81	Longitudinal
PWY-6387	PWY-6387: UDP-N-acetylmuramoyl-pentapeptide biosynthesis I (meso-diaminopimelate containing)	OSCI_Class	Severe	-153.12316	41.3172769	0.00206664	OSCI_ClassSevere	0.0991806	81	81	Longitudinal
PWY-6386	PWY-6386: UDP-N-acetylmuramoyl-pentapeptide biosynthesis II (lysine-containing)	OSCI_Class	Severe	-158.38076	43.2082438	0.00193128	OSCI_ClassSevere	0.09880497	81	81	Longitudinal
RIBOSYN2-PWY	RIBOSYN2-PWY: flavin biosynthesis I (bacteria and plants)	OSCI_Class	Mild	-73.948307	22.1599036	0.00160839	OSCI_ClassMild	0.08846376	81	81	Longitudinal
COA-PWY-1	COA-PWY-1: coenzyme A biosynthesis II (mammalian)	OSCI_Class	Severe	-132.95548	36.204371	0.00084129	OSCI_ClassSevere	0.06525202	81	81	Longitudinal
METHGLYUT-PWY	METHGLYUT-PWY: superpathway of methylglyoxal degradation	OSCI_Class	Severe	17.2698114	4.79670869	0.00085023	OSCI_ClassSevere	0.06525202	81	73	Longitudinal
PWY-6700	PWY-6700: queuosine biosynthesis	OSCI_Class	Severe	-123.66623	33.0083041	0.00070764	OSCI_ClassSevere	0.06091353	81	81	Longitudinal
PWY0-1298	PWY0-1298: superpathway of pyrimidine deoxyribonucleosides degradation	OSCI_Class	Severe	64.7373965	15.5071111	0.00057161	OSCI_ClassSevere	0.05879199	81	81	Longitudinal
TRNA-CHARGING-PWY	TRNA-CHARGING-PWY: tRNA charging	OSCI_Class	Severe	-146.73103	38.1097349	0.00059158	OSCI_ClassSevere	0.05879199	81	81	Longitudinal

Supplementary Table 24: Mean variance contribution of patients' covariates at baseline.

Covariate	Variance mean
Residuals	0.888
Fever before baseline	0.031
BMI	0.028
Age	0.027
Other key COVID-19-related symptoms	0.017
Sex	0.009

Data from 34 patients receiving nicotinamide and 30 receiving placebo.

2.5. Safety data

Supplementary Table 25: Adverse events sorted by MedDRA System Organ Class and Preferred Term.

System Organ Class	Preferred Term	Complaint / adverse event	Nicotinamide			Placebo			Total			Fisher exact test
			Patients	%	Events	Patients	%	Events	Patients	%	Events	P value**
Any System Organ Class	All*		317	70.76%	1798	297	65.71%	1732	614	68.22%	3530	0.115
Blood and lymphatic system disorders	All		0	0.00%	0	1	0.22%	1	1	0.11%	1	>0.999
	Lymphadenopathy	Swollen lymph nodes	0	0.00%	0	1	0.22%	1	1	0.11%	1	>0.999
Cardiac disorders	All		12	2.68%	12	9	1.99%	9	21	2.33%	21	0.517
	Angina pectoris	Angina pectoris	0	0.00%	0	1	0.22%	1	1	0.11%	1	>0.999
	Arrhythmia	Cardiac arrhythmia	2	0.45%	2	0	0.00%	0	2	0.22%	2	0.248
	Cardiovascular disorder	Circulatory problems	1	0.22%	1	1	0.22%	1	2	0.22%	2	>0.999
	Chest pain	Heart pain	0	0.00%	0	3	0.66%	3	3	0.33%	3	0.249
	Myocarditis	Myocarditis	1	0.22%	1	0	0.00%	0	1	0.11%	1	0.498
	Peripheral swelling	Swollen leg	1	0.22%	1	0	0.00%	0	1	0.11%	1	0.498
		Swollen leg: possible post-thrombotic syndrome	0	0.00%	0	1	0.22%	1	1	0.11%	1	>0.999
	Tachycardia	Tachycardia	7	1.56%	7	3	0.66%	3	10	1.11%	10	0.222
Ear and labyrinth disorders	All		11	2.46%	11	12	2.65%	12	23	2.56%	23	>0.999
	Ear pain	Ear pain	3	0.67%	3	1	0.22%	1	4	0.44%	4	0.372
	Eustachian tube dysfunction	Plugged ears	0	0.00%	0	6	1.33%	6	6	0.67%	6	0.031
	External ear inflammation	Ear canal inflammation	1	0.22%	1	0	0.00%	0	1	0.11%	1	0.498
	Hyperacusis	Noise sensitivity	0	0.00%	0	1	0.22%	1	1	0.11%	1	>0.999
	Hypoacusis	Impaired hearing	2	0.45%	2	0	0.00%	0	2	0.22%	2	0.248
	Middle ear effusion	Middle ear effusion	0	0.00%	0	1	0.22%	1	1	0.11%	1	>0.999
	Otitis media	Otitis media	1	0.22%	1	0	0.00%	0	1	0.11%	1	0.498
	Tinnitus	Tinnitus	3	0.67%	3	3	0.66%	3	6	0.67%	6	>0.999
	Vertigo	Transient vertigo	1	0.22%	1	0	0.00%	0	1	0.11%	1	0.498
Endocrine disorders	All		1	0.22%	1	0	0.00%	0	1	0.11%	1	0.498
	Thyroiditis subacute	Viral thyroiditis	1	0.22%	1	0	0.00%	0	1	0.11%	1	0.498
Eye disorders	All		11	2.46%	11	3	0.66%	3	14	1.56%	14	0.033
	Blepharospasm	Eye twitching	1	0.22%	1	0	0.00%	0	1	0.11%	1	0.498

System Organ Class	Preferred Term	Complaint / adverse event	Nicotinamide			Placebo			Total			Fisher exact test
			Patients	%	Events	Patients	%	Events	Patients	%	Events	P value**
Eye disorders (*cont.*)	Conjunctivitis	Conjunctivitis	6	1.34%	6	0	0.00%	0	6	0.67%	6	0.015
	Dry eye	Dry eyes	1	0.22%	1	1	0.22%	1	2	0.22%	2	>0.999
	Visual impairment	Impaired vision	3	0.67%	3	2	0.44%	2	5	0.56%	5	0.685
Gastrointestinal disorders	All		113	25.22%	113	80	17.70%	82	193	21.44%	195	0.007
	Abdominal distension	Bloating	2	0.45%	2	3	0.66%	3	5	0.56%	5	>0.999
	Abdominal pain	Abdominal pain	10	2.23%	10	6	1.33%	6	16	1.78%	16	0.326
	Breath odor	Bad breath	1	0.22%	1	0	0.00%	0	1	0.11%	1	0.498
	Constipation	Constipation	4	0.89%	4	2	0.44%	2	6	0.67%	6	0.450
	Diarrhea	Diarrhea	10	2.23%	10	10	2.21%	11	20	2.22%	21	>0.999
		Increased diarrhea	1	0.22%	1	0	0.00%	0	1	0.11%	1	0.498
	Dyspepsia	Heartburn	3	0.67%	3	2	0.44%	2	5	0.56%	5	0.685
	Dysphagia	Difficulty swallowing the trial tablets	2	0.45%	2	0	0.00%	0	2	0.22%	2	0.248
		Dysphagia	1	0.22%	1	0	0.00%	0	1	0.11%	1	0.498
	Eructation	Belching	1	0.22%	1	0	0.00%	0	1	0.11%	1	0.498
	Feces discolored	Stool color changes	0	0.00%	0	1	0.22%	1	1	0.11%	1	>0.999
	Feces hard	Hard stool	3	0.67%	3	0	0.00%	0	3	0.33%	3	0.123
	Feces soft	Soft stool	6	1.34%	6	1	0.22%	1	7	0.78%	7	0.068
	Flatulence	Flatulence	2	0.45%	2	3	0.66%	3	5	0.56%	5	>0.999
	Gastritis	Gastritis	0	0.00%	0	2	0.44%	2	2	0.22%	2	0.499
	Gastrointestinal disorder	Digestive problems	1	0.22%	1	1	0.22%	1	2	0.22%	2	>0.999
	Gastrointestinal sounds abnormal	Bowel sounds	1	0.22%	1	0	0.00%	0	1	0.11%	1	0.498
		Stomach noises	2	0.45%	2	0	0.00%	0	2	0.22%	2	0.248
	Gastroesophageal reflux disease	Increased reflux	1	0.22%	1	0	0.00%	0	1	0.11%	1	0.498
	Gingival bleeding	Bleeding gums	1	0.22%	1	0	0.00%	0	1	0.11%	1	0.498
	Nausea	Nausea	8	1.79%	8	7	1.55%	7	15	1.67%	15	0.801
	Noninfective gingivitis	Inflamed gums	0	0.00%	0	1	0.22%	1	1	0.11%	1	>0.999
	Pulpitis dental	Inflamed tooth	1	0.22%	1	0	0.00%	0	1	0.11%	1	0.498
	Taste disorder	Impaired sense of taste	48	10.71%	48	40	8.85%	41	88	9.78%	89	0.370
	Tongue coated	Coated tongue	2	0.45%	2	0	0.00%	0	2	0.22%	2	0.248
	Tongue discomfort	Burning sensation on the tongue	2	0.45%	2	0	0.00%	0	2	0.22%	2	0.248

System Organ Class	Preferred Term	Complaint / adverse event	Nicotinamide			Placebo			Total			Fisher exact test
			**Patients**	**%**	**Events**	**Patients**	**%**	**Events**	**Patients**	**%**	**Events**	**P value****
Gastrointestinal disorders (*cont.*)	Vomiting	Vomiting	0	0.00%	0	1	0.22%	1	1	0.11%	1	>0.999
General disorders and administration site conditions	All		111	24.78%	122	109	24.12%	118	220	24.44%	240	0.877
	Adverse reaction	Unspecified side effects	2	0.45%	2	2	0.44%	2	4	0.44%	4	>0.999
	Chest pain	Chest pain	13	2.90%	13	16	3.54%	17	29	3.22%	30	0.707
	Chills	Chills	2	0.45%	2	1	0.22%	1	3	0.33%	3	0.623
	Decreased activity	Performance drop (reduced physical performance)	51	11.38%	58	33	7.30%	34	84	9.33%	92	0.039
	Facial pain	Pain in the face and jaws	0	0.00%	0	1	0.22%	1	1	0.11%	1	>0.999
	Fatigue	Fatigue	18	4.02%	19	23	5.09%	23	41	4.56%	42	0.523
	General physical health deterioration	Reduced ability to perform normal activities	15	3.35%	17	17	3.76%	22	32	3.56%	39	0.858
	Hot flush	Hot flashes	3	0.67%	3	1	0.22%	1	4	0.44%	4	0.372
	Malaise	General feeling of sickness / malaise	2	0.45%	3	6	1.33%	8	8	0.89%	11	0.287
	Mucosal disorder	Dry mucous membranes (respiratory tract)	1	0.22%	1	1	0.22%	1	2	0.22%	2	>0.999
	Night sweats	Night sweats	2	0.45%	2	1	0.22%	1	3	0.33%	3	0.623
	Peripheral coldness	Cold fingers	0	0.00%	0	1	0.22%	1	1	0.11%	1	>0.999
		Cold hands and feet	0	0.00%	0	1	0.22%	1	1	0.11%	1	>0.999
	Pyrexia	Fever	1	0.22%	1	3	0.66%	3	4	0.44%	4	0.624
	Sensation of foreign body	Lump in throat	1	0.22%	1	2	0.44%	2	3	0.33%	3	>0.999
Infections and infestations	All		8	1.79%	8	14	3.10%	14	22	2.44%	22	0.280
	Appendicitis	Suspected appendicitis	1	0.22%	1	0	0.00%	0	1	0.11%	1	0.498
	COVID-19	Death by COVID-19	0	0.00%	0	0	0.00%	0	0	0.00%	0	>0.999
	Herpes zoster	Shingles	0	0.00%	0	1	0.22%	1	1	0.11%	1	>0.999
	Pharyngitis	Pharyngitis	2	0.45%	2	0	0.00%	0	2	0.22%	2	0.248
	Pneumonia	Pneumonia	4	0.89%	4	7	1.55%	7	11	1.22%	11	0.546
	Sinusitis	Sinusitis	1	0.22%	1	4	0.88%	4	5	0.56%	5	0.374
	Tonsillitis	Tonsillitis	0	0.00%	0	2	0.44%	2	2	0.22%	2	0.499
Investigations	All		16	3.57%	16	15	3.32%	15	31	3.44%	31	0.857
	Blood pressure decreased	Decreased blood pressure	1	0.22%	1	1	0.22%	1	2	0.22%	2	>0.999
	Blood pressure increased	Increased blood pressure	1	0.22%	1	2	0.44%	2	3	0.33%	3	>0.999

System Organ Class	Preferred Term	Complaint / adverse event	Nicotinamide			Placebo			Total			Fisher exact test
			**Patients**	**%**	**Events**	**Patients**	**%**	**Events**	**Patients**	**%**	**Events**	**P value****
Investigations (*cont.*)	Blood test abnormal	Deteriorated blood parameters	1	0.22%	1	0	0.00%	0	1	0.11%	1	0.498
	Emergency care examination	Examination in an emergency department without hospitalization	8	1.79%	8	7	1.55%	7	15	1.67%	15	0.801
	Heart rate increased	Elevated heart rate (resting)	3	0.67%	3	2	0.44%	2	5	0.56%	5	0.685
		Elevated heart rate (under stress)	0	0.00%	0	1	0.22%	1	1	0.11%	1	>0.999
	Hepatic enzyme increased	Increased liver enzymes	0	0.00%	0	1	0.22%	1	1	0.11%	1	>0.999
	Respiratory rate increased	Increased respiratory rate	0	0.00%	0	1	0.22%	1	1	0.11%	1	>0.999
	Tidal volume decreased	Reduced lung volume	2	0.45%	2	0	0.00%	0	2	0.22%	2	0.248
Metabolism and nutrition disorders	All		8	1.79%	9	3	0.66%	3	11	1.22%	12	0.142
	Alcohol intolerance	Reduced alcohol tolerance	1	0.22%	1	0	0.00%	0	1	0.11%	1	0.498
	Decreased appetite	Loss of appetite / lower food intake	6	1.34%	7	3	0.66%	3	9	1.00%	10	0.339
	Increased appetite	Increased appetite	1	0.22%	1	0	0.00%	0	1	0.11%	1	0.498
Musculoskeletal and connective tissue disorders	All		35	7.81%	35	28	6.19%	28	63	7.00%	63	0.363
	Arthralgia	Joint pain	9	2.01%	9	8	1.77%	8	17	1.89%	17	0.812
	Back pain	Back pain	3	0.67%	3	3	0.66%	3	6	0.67%	6	>0.999
	Myalgia	Muscle pain	13	2.90%	13	14	3.10%	14	27	3.00%	27	>0.999
	Neck pain	Neck pain	1	0.22%	1	1	0.22%	1	2	0.22%	2	>0.999
	Pain in extremity	Finger pain	1	0.22%	1	0	0.00%	0	1	0.11%	1	0.498
		Foot pain	1	0.22%	1	0	0.00%	0	1	0.11%	1	0.498
		Limb pain	7	1.56%	7	2	0.44%	2	9	1.00%	9	0.106
Nervous system disorders	All		73	16.29%	74	75	16.59%	75	148	16.44%	149	0.928
	Aphasia	Word finding difficulties	4	0.89%	4	7	1.55%	7	11	1.22%	11	0.546
	Balance disorder	Balance disorders	0	0.00%	0	1	0.22%	1	1	0.11%	1	>0.999
	Dizziness	Dizziness	22	4.91%	23	18	3.98%	18	40	4.44%	41	0.522
	Headache	Headache	31	6.92%	31	32	7.08%	32	63	7.00%	63	>0.999
	Memory impairment	Memory problems	12	2.68%	12	15	3.32%	15	27	3.00%	27	0.697
	Migraine	Migraine	1	0.22%	1	0	0.00%	0	1	0.11%	1	0.498
	Neuralgia	Nerve pain (arm, hand)	0	0.00%	0	1	0.22%	1	1	0.11%	1	>0.999

System Organ Class	Preferred Term	Complaint / adverse event	Nicotinamide			Placebo			Total			Fisher exact test
			Patients	%	Events	Patients	%	Events	Patients	%	Events	P value**
Nervous system disorders (*cont.*)	Neuralgia (*cont.*)	Nerve pain (arm)	1	0.22%	1	0	0.00%	0	1	0.11%	1	0.498
		Nerve pain (leg)	0	0.00%	0	1	0.22%	1	1	0.11%	1	>0.999
	Paraesthesia	Tingling in the abdomen	1	0.22%	1	0	0.00%	0	1	0.11%	1	0.498
	Restless legs syndrome	Restless legs	1	0.22%	1	0	0.00%	0	1	0.11%	1	0.498
Psychiatric disorders	All		99	22.10%	122	112	24.78%	147	211	23.44%	269	0.346
	Apathy	Motivation problems	1	0.22%	1	0	0.00%	0	1	0.11%	1	0.498
	Confusional state	Confusion	9	2.01%	9	11	2.43%	11	20	2.22%	20	0.822
	Depression	Depression	0	0.00%	0	1	0.22%	1	1	0.11%	1	>0.999
	Disturbance in attention	Concentration problems	25	5.58%	25	24	5.31%	24	49	5.44%	49	0.884
	Irritability	Irritability	1	0.22%	1	1	0.22%	1	2	0.22%	2	>0.999
	Nervousness	Nervousness	0	0.00%	0	2	0.44%	2	2	0.22%	2	0.499
	Panic attack	Panic attacks	1	0.22%	1	1	0.22%	1	2	0.22%	2	>0.999
	Restlessness	Restlessness	0	0.00%	0	2	0.44%	2	2	0.22%	2	0.499
	Sleep disorder	Disturbed sleep	62	13.84%	85	70	15.49%	105	132	14.67%	190	0.510
Reproductive system and breast disorders	All		1	0.22%	1	2	0.44%	2	3	0.33%	3	>0.999
	Intermenstrual bleeding	Intermenstrual bleeding	0	0.00%	0	1	0.22%	1	1	0.11%	1	>0.999
	Menstrual disorder	Prolonged menstrual bleeding	1	0.22%	1	0	0.00%	0	1	0.11%	1	0.498
	Menstruation irregular	Irregular menstrual cycle	0	0.00%	0	1	0.22%	1	1	0.11%	1	>0.999
Respiratory, thoracic and mediastinal disorders	All		368	82.14%	404	368	81.42%	405	736	81.78%	809	0.796
	Bronchial disorder	Mucus in the bronchi	1	0.22%	1	0	0.00%	0	1	0.11%	1	0.498
	Bronchitis	Bronchitis	1	0.22%	1	2	0.44%	2	3	0.33%	3	>0.999
	Chest discomfort	Chest tightness	7	1.56%	7	5	1.11%	5	12	1.33%	12	0.577
	Cough	Cough	59	13.17%	63	65	14.38%	67	124	13.78%	130	0.629
		Severe cough	0	0.00%	0	1	0.22%	1	1	0.11%	1	>0.999
	Dysphonia	Hoarseness	7	1.56%	7	10	2.21%	11	17	1.89%	18	0.626
	Dyspnea	Shortness of breath	135	30.13%	162	121	26.77%	139	256	28.44%	301	0.269
	Hyposmia	Impaired sense of smell	58	12.95%	59	52	11.50%	54	110	12.22%	113	0.542
	Nasal discomfort	Cold nose	0	0.00%	0	1	0.22%	1	1	0.11%	1	>0.999
	Nasal dryness	Dry nose	0	0.00%	0	1	0.22%	1	1	0.11%	1	>0.999
	Oropharyngeal pain	Sore throat	21	4.69%	21	17	3.76%	18	38	4.22%	39	0.512

System Organ Class	Preferred Term	Complaint / adverse event	Nicotinamide			Placebo			Total			Fisher exact test
			Patients	%	Events	Patients	%	Events	Patients	%	Events	P value**
Respiratory, thoracic and mediastinal disorders (*cont.*)	Productive cough	Cough with sputum production	28	6.25%	29	20	4.42%	21	48	5.33%	50	0.238
	Respiratory tract congestion	Congested sensation in the respiratory tract	1	0.22%	1	0	0.00%	0	1	0.11%	1	0.498
	Respiratory tract disorders	Mucus production in the respiratory tract	19	4.24%	22	36	7.96%	47	57	6.33%	69	0.025
	Respiratory tract irritation	Lung irritation	0	0.00%	0	1	0.22%	1	1	0.11%	1	>0.999
	Rhinorrhea	Rhinitis/rhinorrhea	21	4.69%	21	28	6.19%	29	49	5.44%	50	0.378
	Sinus congestion	Sinus congestion	1	0.22%	1	3	0.66%	3	4	0.44%	4	0.624
	Throat clearing	Frequent throat clearing	2	0.45%	2	1	0.22%	1	3	0.33%	3	0.623
	Wheezing	Whistling/wheezing breathing	7	1.56%	7	4	0.88%	4	11	1.22%	11	0.383
Skin and subcutaneous tissue disorders	All		26	5.80%	26	29	6.42%	29	55	6.11%	55	0.781
	Alopecia	Hair loss	10	2.23%	10	10	2.21%	10	20	2.22%	20	>0.999
	Dermatitis atopic	Atopic dermatitis flare	1	0.22%	1	0	0.00%	0	1	0.11%	1	0.498
	Dry skin	Dry skin	2	0.45%	2	1	0.22%	1	3	0.33%	3	0.623
	Hyperhidrosis	Sweats	1	0.22%	1	3	0.66%	3	4	0.44%	4	0.624
	Pruritus	Pruritus	1	0.22%	1	1	0.22%	1	2	0.22%	2	>0.999
	Rash	Skin rash	10	2.23%	10	12	2.65%	12	22	2.44%	22	0.830
	Skin discoloration	Discolored toes	1	0.22%	1	0	0.00%	0	1	0.11%	1	0.498
	Skin discomfort	Skin discomfort (face)	0	0.00%	0	1	0.22%	1	1	0.11%	1	>0.999
		Skin discomfort (legs)	0	0.00%	0	1	0.22%	1	1	0.11%	1	>0.999
Social circumstances	All		0	0.00%	0	1	0.22%	1	1	0.11%	1	>0.999
	Overwork	Overwhelmed at work	0	0.00%	0	1	0.22%	1	1	0.11%	1	>0.999
Surgical and medical procedures	All		4	0.89%	4	4	0.88%	4	8	0.89%	8	>0.999
	Hospitalization	Hospitalization	4	0.89%	4	3	0.66%	3	7	0.78%	7	0.724
	Hospitalization, oxygen therapy	Hospitalization with a continuous oxygen requirement of more than 24 hours	0	0.00%	0	1	0.22%	1	1	0.11%	1	>0.999
	Intensive care	Intensive care requirement	0	0.00%	0	0	0.00%	0	0	0.00%	0	>0.999
	Mechanical ventilation	Ventilation requirement	0	0.00%	0	0	0.00%	0	0	0.00%	0	>0.999
Vascular disorders	All		2	0.45%	2	0	0.00%	0	2	0.22%	2	0.248
	Hematoma	Hematomas	1	0.22%	1	0	0.00%	0	1	0.11%	1	0.498

System Organ Class	Preferred Term	Complaint / adverse event	Nicotinamide			Placebo			Total			Fisher exact test
			**Patients**	**%**	**Events**	**Patients**	**%**	**Events**	**Patients**	**%**	**Events**	**P value****
Vascular disorders (*cont.*)	Varicose vein ruptured	Recurrence of anal varicose vein rupture	1	0.22%	1	0	0.00%	0	1	0.11%	1	0.498
Vision disorders	All		0	0.00%	0	1	0.22%	1	1	0.11%	1	>0.999
	Photopsia	Flashes of light in the eyes	0	0.00%	0	1	0.22%	1	1	0.11%	1	>0.999

The safety population (n=900) consisted of 448 patients assigned to receive nicotinamide and 452 patients assigned to receive placebo.

* As some patients reported AEs from multiple System Organ Classes, the total number of patients in the summary line “System Organ Class: Any System Organ Class; Preferred Term: All” is not identical to the total number of patients from all individual System Organ Classes / Preferred Terms.

** Unadjusted P values, two-sided, from exploratory analyses. None of the tests remains statistically significant after applying corrections for multiple testing.

Supplementary Table 26: Severe COVID-19, Serious Adverse Events and emergency treatment in the safety population at week 0 (baseline).

Events / status at week 0	Nicotinamide n=448		Placebo n=452		Total n=900
	n	%	n	%	n
Missing	0	0.00	0	0.00	0
**WHO Scale (current status at week 0 interview)**					
0 – No clinical or virological evidence of infection	0	0.00	0	0.00	0
1 – No limitation of activities	65	14.51	70	15.49	135
2 – Limitation of activities	383	85.49	382	84.51	765
3 – Hospitalized, no oxygen therapy	0	0.00	0	0.00	0
4 – Oxygen by mask or nasal prongs	0	0.00	0	0.00	0
5 – Non-invasive ventilation or high-flow oxygen	0	0.00	0	0.00	0
6 – Intubation and mechanical ventilation	0	0.00	0	0.00	0
7 – Ventilation + additional organ support – pressors, RRT, ECMO	0	0.00	0	0.00	0
8 – Death	0	0.00	0	0.00	0
**Severe COVID-19 (before week 0 [Baseline])**					
Examination in an emergency department without hospitalization	2	0.45	0	0.00	2
Hospitalization	0	0.00	0	0.00	0
Hospitalization with a continuous oxygen requirement of ≥24 hours	0	0.00	0	0.00	0
Intensive care requirement	0	0.00	0	0.00	0
Ventilation requirement	0	0.00	0	0.00	0
Death by COVID-19	0	0.00	0	0.00	0
**Duration of hospitalization [days]**					
0	448	100.00	452	100.00	900

Supplementary Table 27: Severe COVID-19, Serious Adverse Events and emergency treatment in the safety population at week 2.

Events / status at week 2	Nicotinamide n=448		Placebo n=452		Total n=900
	n	%	n	%	n
Missing	31	6.92	27	5.97	58
**WHO Scale (current status at week 2 interview)**					
0 – No clinical or virological evidence of infection	0	0.00	1	0.24	1
1 – No limitation of activities	233	55.88	222	52.24	455
2 – Limitation of activities	184	44.12	201	47.29	385
3 – Hospitalized, no oxygen therapy	0	0.00	0	0.00	0
4 – Oxygen by mask or nasal prongs	0	0.00	1	0.24	1
5 – Non-invasive ventilation or high-flow oxygen	0	0.00	0	0.00	0
6 – Intubation and mechanical ventilation	0	0.00	0	0.00	0
7 – Ventilation + additional organ support – pressors, RRT, ECMO	0	0.00	0	0.00	0
8 – Death	0	0.00	0	0.00	0
**Severe COVID-19 (last two weeks)**					
Examination in an emergency department without hospitalization	3	0.72	5	1.18	10
Hospitalization	4	0.96	3	0.71	7
Hospitalization with a continuous oxygen requirement of ≥24 hours	0	0.00	1	0.24	0
Intensive care requirement	0	0.00	0	0.00	0
Ventilation requirement	0	0.00	0	0.00	0
Death by COVID-19	0	0.00	0	0.00	0
**Duration of hospitalization [days]**					
0	413	99.04	422	99.28	841
2	1	0.24	0	0.00	1
4	0	0.00	1	0.24	1
5	1	0.24	1	0.24	2
7	1	0.24	0	0.00	1
8	1	0.24	1	0.24	2

Supplementary Table 28: Severe COVID-19, Serious Adverse Events and emergency treatment in the safety population at week 4.

Events / status at week 4	Nicotinamide n=448		Placebo n=452		Total n=900
	n	%	n	%	n
Missing	36	8.04	34	7.52	70
**WHO Scale (current status at week 4 interview)**					
0 – No clinical or virological evidence of infection	0	0.00	0	0.00	0
1 – No limitation of activities	276	66.99	281	67.22	557
2 – Limitation of activities	136	33.01	137	32.78	273
3 – Hospitalized, no oxygen therapy	0	0.00	0	0.00	0
4 – Oxygen by mask or nasal prongs	0	0.00	0	0.00	0
5 – Non-invasive ventilation or high-flow oxygen	0	0.00	0	0.00	0
6 – Intubation and mechanical ventilation	0	0.00	0	0.00	0
7 – Ventilation + additional organ support – pressors, RRT, ECMO	0	0.00	0	0.00	0
8 – Death	0	0.00	0	0.00	0
**Severe COVID-19 (last two weeks)**					
Examination in an emergency department without hospitalization	1	0.24	2	0.48	3
Hospitalization	0	0.00	1	0.24	1
Hospitalization with a continuous oxygen requirement of ≥24 hours	0	0.00	0	0.00	0
Intensive care requirement	0	0.00	0	0.00	0
Ventilation requirement	0	0.00	0	0.00	0
Death by COVID-19	0	0.00	0	0.00	0
**Duration of hospitalization [days]**					
0	412	100.00	417	99.76	829
2 (plus 8 before Week 2 interview)	0	0.00	1	0.24	1

Supplementary Table 29: Severe COVID-19, Serious Adverse Events and emergency treatment in the safety population at week 6.

Events / status at week 6	Nicotinamide n=448		Placebo n=452		Total n=900
	n	%	n	%	n
missing	46	10.27	34	7.52	80
**WHO Scale (current status at week 6 interview)**					
0 – No clinical or virological evidence of infection	0	0.00	0	0.00	0
1 – No limitation of activities	309	76.87	333	79.67	642
2 – Limitation of activities	93	23.13	85	20.33	178
3 – Hospitalized, no oxygen therapy	0	0.00	0	0.00	0
4 – Oxygen by mask or nasal prongs	0	0.00	0	0.00	0
5 – Non-invasive ventilation or high-flow oxygen	0	0.00	0	0.00	0
6 – Intubation and mechanical ventilation	0	0.00	0	0.00	0
7 – Ventilation + additional organ support – pressors, RRT, ECMO	0	0.00	0	0.00	0
8 – Death	0	0.00	0	0.00	0
**Severe COVID-19 (last two weeks)**					
Examination in an emergency department without hospitalization	2	0.50	0	0.00	2
Hospitalization	0	0.00	0	0.00	0
Hospitalization with a continuous oxygen requirement of ≥24 hours	0	0.00	0	0.00	0
Intensive care requirement	0	0.00	0	0.00	0
Ventilation requirement	0	0.00	0	0.00	0
Death by COVID-19	0	0.00	0	0.00	0
**Duration of hospitalization [days]**					
0	402	100.00	418	100.00	820

3. Supplementary information on methods for COVit-2

3.1. Trial procedures and design of COVit-2

The COVit-2 trial was performed remotely. Patients had to be able to give electronic written informed consent, which was explained and confirmed by a direct telephone call with the patient.

Patients were recruited through 24 independent diagnostic laboratory service providers with a total of 71 sites all over Germany. The following alphabetical list also contains the key collaborators:

1. alphaomega Labor GbR (PD Dr. Grit Ackermann, Sandra Wolf), Leipzig.
2. AniCon Labor GmbH (Dr. Klaus-Peter Behr), Höltinghausen.
3. Bioscientia Healthcare GmbH (Dr. Oliver Harzer), Ingelheim.
4. Centogene GmbH (Prof. Dr. Peter Bauer), Rostock.
5. Labopart – Labor Chemnitz (Dr. Michael Gerber), Chemnitz.
6. Laborärztliche Gemeinschaftspraxis Lübeck (Dr. Andreas Bobrowski), Lübeck.
7. Labor Becker & Kollegen MVZ GbR (Prof. Dr. Dr. Jürgen Durner), Munich.
8. Labor Blumenstraße (Dr. Frank Wietschel), Erfurt.
9. Labor Dr. Heidrich & Kollegen MVZ GmbH (Prof. Dr. Matthias Maaß), Hamburg.
10. Labor Dr. Krause & Kollegen MVZ GmbH (Dr. Thomas Lorentz, Dr. Olaf Grobe), Kiel.
11. Labor Dr. von Froreich GmbH (Prof. Dr. Tammo von Schrenck), Hamburg.
12. Labor Mohr (Harald Mohr), Kiel.
13. LADR Laboratory Group Dr. Kramer & Colleagues (Prof. Dr. Jan Kramer), Geesthacht.
14. Medizinisches Labor Ostsachsen MVZ GbR (Thomas Kirchner), Dresden.
15. Medizinisches Labor Westsachsen MVZ GbR (PD Dr. Dr. Peter Reichardt), Zwickau.
16. MVZ Dr. Eberhard & Partner Dortmund ÜBAG (Albert Pranada), Dortmund.
17. MVZ Dr. Stein + Kollegen (Dr. Dietmar Dreßen, Dr. Jelena Cucuz), Mönchengladbach.
18. MVZ Humangenetik Ulm GbR (PD Dr. Dietmar Plonné), Ulm.
19. MVZ Labor 28 GmbH (Dr. Michael Müller), Berlin.
20. MVZ Labor Dortmund (Dr. Matthias Aymanns), Dortmund.
21. MVZ Labor PD Dr. Volkmann und Kollegen GbR (PD Dr. Martin Volkmann), Karlsruhe.
22. MVZ Labor Ludwigsburg GbR (Prof. Dr. Rüdiger Braun), Ludwigsburg.
23. MVZ Medizinische Labore Dessau Kassel GmbH (Dr. Juliane Böttcher-Lorenz, Dr. Christina Kiel), Dessau-Roßlau.
24. Praxis für Labormedizin und Mikrobiologie ÜBAG GbR (Dr. Silke Biermann-Göcke), Bochum.

All patients who tested positive in the recruitment period at the laboratory sites were contacted together with the transmission of their laboratory results. Patients volunteered their identity to the recruitment center and after verification of key data including the referring laboratories and key inclusion criteria such as the test date, patients received the personalized patient information and a participation code, and could give informed consent on a secure trial website. Subsequently, they were contacted by telephone to check all inclusion and exclusion criteria and explain the informed consent. Immediately after confirming eligibility, patients were randomized and received the intervention and the optional paper questionnaires (SF-36 questionnaire: RAND 36-Item Health Survey 1.0; FACIT-F: 40-item Functional Assessment of Chronic Illness Therapy – Fatigue) by next-day courier shipment. Eligible patients were randomly assigned in a 1:1 ratio, by means of a randomization code in the trial database, to self-administer daily with breakfast either 1 g/d nicotinamide (500 mg immediate-release nicotinamide and 500 mg controlled-ileocolonic-release nicotinamide [CICR-NAM]) or matched placebo, for 4 weeks.

Nicotinamide is Generally Recognized As Safe (GRAS) by the FDA and has been assigned a daily Tolerable Upper Intake Level of 900 mg/d as a dietary supplement (vitamin B3) by the European authorities (EFSA).[4] Immediate-release nicotinamide was purchased as 500-mg dietary supplement tablets (NicoPel®; Derma Enzinger; Ainring, Germany). Corresponding placebo tablets were purchased from FAGRON (Barsbüttel, Germany). The 500-mg CICR-NAM tablets and corresponding placebo tablets were manufactured by NextPharma (Göttingen, Germany). Tablets were released by the pharmacy of the University Hospital Schleswig-Holstein (Kiel, Germany). Together with the trial intervention, patients received administration instructions which were additionally explained to them in detail at the baseline visit.

Patients underwent structured telephone interviews conducted by blinded personnel at weeks 2, 4 and 6 as well as 6 months after baseline. Due to the patient-focused nature of the interview questions, patients usually did not have to retrieve data from their attending physician or other sources. Concomitant medication and supplements were recorded in detail at each interview. Any nicotinamide intake from dietary supplements was far below the intervention dose in the trial (1,000 mg/d). The interviewers queried symptoms one after another in a structured manner, according to SOPs and carefully instructed about not introducing bias or asking any leading questions. The interviewers made sure that the patients understood each question correctly. For example, it was made clear

that the reference point for performance drop or a reduced ability to perform normal activities (the two main and closely related endpoints pertaining to physical performance) was the health status of the patient directly prior to SARS-CoV-2 infection. Compliance was surveyed by tablet count during each interview and through specific questions. After week 6, patients returned the remaining trial supplements for accountability and destruction as well as the optional paper questionnaires completed on the same day of the interviews. Optional stool samples (at the time of each interview) and optional capillary blood samples for measuring levels of antibodies directed against the nucleocapsid (N) or spike (S) proteins of SARS-CoV-2 (after at least 6 months; AProof® Duo Test; Adversis Pharma/AP Diagnostics GmbH, Leipzig, Germany) were collected from patients all over Germany via mail. All trial procedures were documented and quality monitored, and all personnel received formal training.

3.2. Lists of COVID-19 symptoms queried by telephone interviews in COVit-2

Binary queries: is the symptom present or not?

A patient is usually fully aware of his/her normal physical performance and can clearly report any even minor discrepancies. This defines the binary primary endpoint "performance drop" as a both robust and sensitive means to detect any patient-reported physical impairment (see Discussion).

- Performance drop (**primary endpoint**)
- Fatigue (**3rd key secondary endpoint**)

- Fever
- Chills
- Shortness of breath
- Whistling/wheezing breathing
- Cough
- Cough with sputum production
- Rhinitis/rhinorrhea
- Sore throat
- Hoarseness
- Pneumonia

- Muscle pain
- Joint pain
- Limb pain
- Chest pain
- Headache

- Abdominal pain
- Diarrhea
- Nausea
- Vomiting
- Loss of appetite/lower food intake
- Other gastrointestinal symptoms (reported individually)

- Impaired sense of smell
- Impaired sense of taste

- Confusion
- Dizziness
- Conjunctivitis
- Skin rash
- Hair loss
- Other symptoms (reported individually)

Ordinal queries from a complaint scale for lower respiratory tract infections:

Gradations: 0 = normal, 1 = very minor problem, 2 = minor problem, 3 = moderately poor, 4 = poor, 5 = very poor, 6 = maximally poor.

- Ability to perform normal activities (**1st key secondary endpoint**)
- Cough (**2nd key secondary endpoint**)
- Mucus production
- Shortness of breath
- Sleep
- General feeling of sickness

3.3. Symptoms and risk factors at baseline in COVit-2

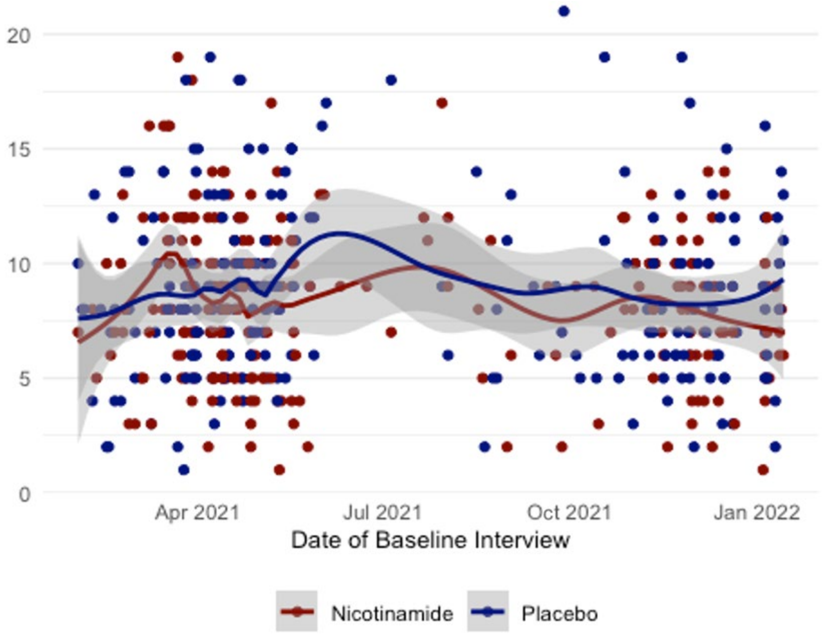

Supplementary Fig. 9: Number of symptoms reported at baseline in the RFITT population.
Each dot represents one patient (n=500). Lines were produced with locally weighted scatterplot (LOESS) smoothing in R. Grey shades are 95% CI bounds of LOESS smoothing.

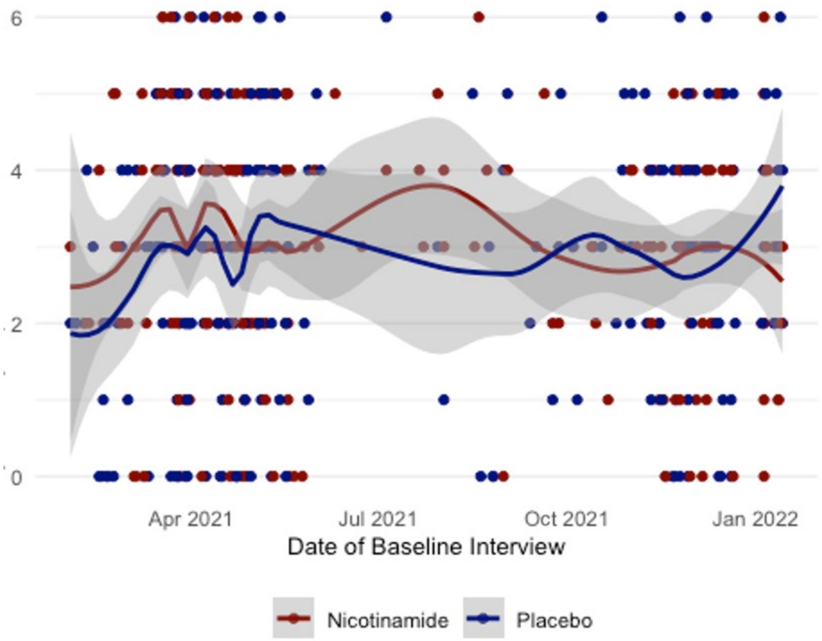

Supplementary Fig. 10: Intensity of reduced ability to perform normal activities reported at baseline in the RFITT population.
Each dot represents one patient (n=500). Lines were produced with locally weighted scatterplot (LOESS) smoothing in R. Grey shades are 95% CI bounds of LOESS smoothing.

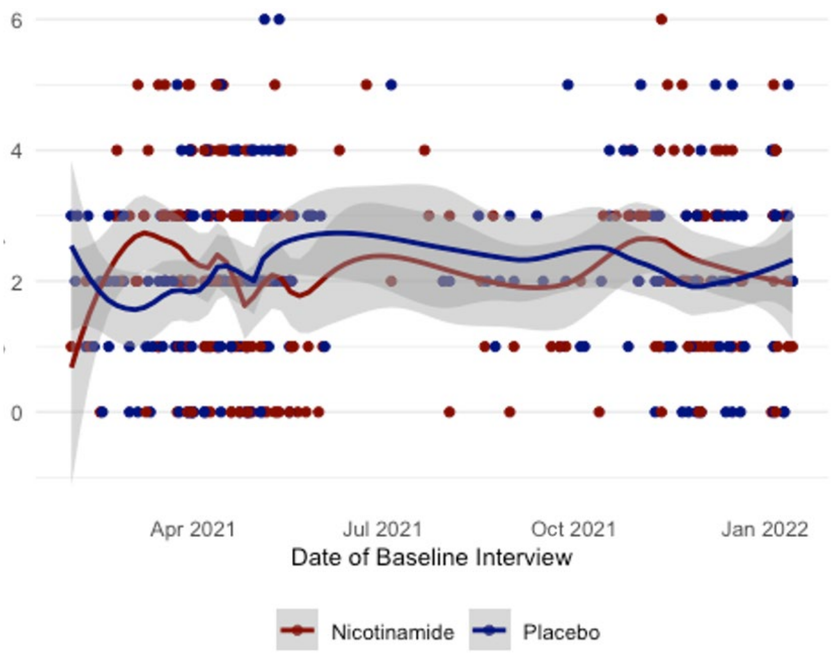

Supplementary Fig. 11: Intensity of cough reported at baseline in the RFITT population.
Each dot represents one patient (n=500). Lines were produced with locally weighted scatterplot (LOESS) smoothing in R. Grey shades are 95% CI bounds of LOESS smoothing.

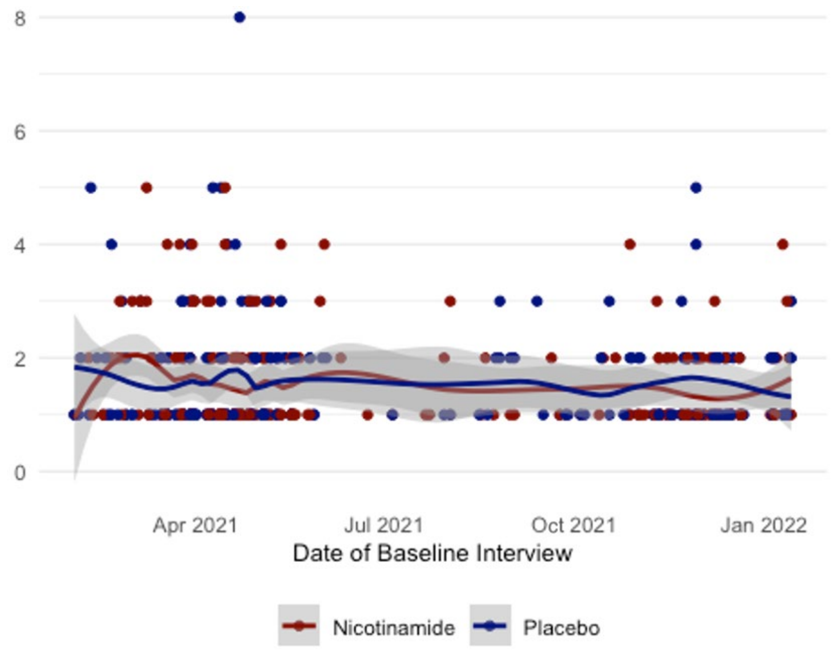

Supplementary Fig. 12: Number of risk factors for severe COVID-19 reported at baseline in the RFITT population.
Each dot represents one patient (n=500). Lines were produced with locally weighted scatterplot (LOESS) smoothing in R. Grey shades are 95% CI bounds of LOESS smoothing.

3.4. Analysis populations and risk factor subgroups in COVit-2

The safety population included all randomized subjects.

The intention-to-treat (ITT) population included all randomized subjects who received at least one dose of the intervention.

Populations and risk groups were defined in the statistical analysis plans (SAPs) for the analyses of acute COVID-19 and for the 6-month follow-up (see sections 6 and 7 of this Supplement, respectively. The RFITT (risk factor intention-to-treat) population was defined as the primary analysis population for efficacy for acute COVID-19. The RFITT population comprised all subjects of the ITT population with a least one characteristic or underlying medical condition associated with an increased risk of developing severe illness from COVID-19 (cf. https://www.cdc.gov/covid/hcp/clinical-care/underlying-conditions.html, last accessed on 11-Mar-2025), selected from the group of traits consisting of

- an age of ≥60 years;
- a body mass index of ≥30.0;
- type 1 diabetes;
- type 2 diabetes;
- cardiovascular diseases;
- high blood pressure;
- stroke;
- asthma;
- chronic obstructive pulmonary disease;
- other chronic lung diseases;
- current or former smokers (the latter being defined as patients who smoked more than 100 cigarettes or other smoking products in total so far, but have not smoked for at least 4 weeks);
- chronic liver diseases;
- chronic kidney diseases;
- cancer;
- organ transplants;
- current immunosuppressive therapy;
- chronic neurological diseases (multiple sclerosis, Parkinson's disease);

The per-protocol populations (PP and RFPP, respectively) included patients of ITT and RFITT, respectively, who did not

- drop out or
- comply regarding investigational product intake for at least 80%, i.e. intake of investigational product for at least 11 of 14 days between each study interval: week 0 – week 2 and week 2 – week 4.

The per-protocol populations were only approximately 5% smaller than the ITT populations (n=831 in PP vs. n=867 in ITT [95.8% per protocol] and n=472 in RFPP vs. n=500 in RFITT [94.4% per protocol], respectively).

The following pre-defined risk subgroups were investigated to differentiate their contribution to the primary and key secondary endpoint results for RFITT in acute COVID-19:

1. Age of ≥60 years.
2. Body mass index of ≥30.0 or type 2 diabetes.
3. Cardiovascular diseases, high blood pressure or stroke.
4. Asthma, chronic obstructive pulmonary disease or other chronic lung diseases.
5. Current or former smokers (the latter being defined as patients who smoked more than 100 cigarettes or other smoking products in total so far, but have not smoked for at least 4 weeks).

For the analysis of post-COVID syndrome (PCS) at the 6-month follow-up, the primary analysis population was the ITT population.

In order to identify patients at risk for developing PCS, baseline characteristics and symptoms of all available patients of the placebo population (n=425) were used to delineate predictors for a PCS score[5] of ≥ 5 at month 6 with a significance threshold of $P<0.05$. The following predictors were found:

- medical history: age >60 years, chronic obstructive pulmonary disease, chronic inflammatory disease, depression, other chronic diseases, permanent intake of medication;
- symptoms from the onset of COVID-19 until the trial baseline: shortness of breath, whistling/wheezing breathing, absence of rhinitis/rhinorrhea, limb pain, nausea, dizziness;

- at baseline: shortness of breath, whistling/wheezing breathing, absence of rhinitis/rhinorrhea, conjunctivitis, chills, muscle pain, limb pain, dizziness, limitation of activity (a score of 2 on the WHO scale of COVID-19 severity developed in 2020).[6]

In order to be included into a subgroup of patients at risk for PCS, patients had to have more than 5 of these predictors at baseline.

A further subgroup was established based on improvement in the primary endpoint or one of the three key secondary endpoints (see section 3.2 of this Supplement) in the acute phase of the disease.

3.5. Methods for gut microbiome analyses in COVit-2

Stool sample collection and selection of the 16S phylogenomic and metagenomics subcohorts

Native samples were collected and shipped by the participants using collection tubes in special return envelopes. Immediately upon arrival at the central laboratory at the Institute of Clinical Molecular Biology (Kiel University and University Hospital Schleswig-Holstein, Kiel, Germany), samples were frozen and stored at – 80°C until DNA extraction. Only samples from complete sample series including week 0, week 2, week 4 and week 6 were used for the analyses.

Stool sampling was optional and conducted during periods of strict quarantine regulations and/or lockdowns. Only a subset of participants provided samples that met the quality criteria for analysis. The main quality control attributes for the selection of stool samples for analysis were the precise sequence of collection dates, the duration of shipping intervals and sufficient sample volumes.

A subcohort of 70 individuals was selected for an initial exploration of the microbiome using 16S rRNA amplicon sequencing. Additionally, a subcohort of 18 individuals was selected based on a targeted matching process. Patients were matched by age, sex, BMI, and key COVID-19-related symptoms (fever, cough, impaired sense of smell or impaired sense of taste at baseline), resulting in the selection of 9 patients from the nicotinamide group and 9 from the placebo group, independent of the initial cohort. Statistical tests confirmed no significant baseline differences in demographics between the nicotinamide and placebo subgroups selected for 16S phylogenome and metagenome analyses (Supplementary Tables 16 and 17).

16S rRNA sequencing and preprocessing

Fecal DNA from 280 stool samples was extracted using the DNeasy PowerSoil Pro Kit (Qiagen) following the manufacturer's protocol. Extracted DNA was eluted from the spin filter silica membrane with 100 µl of elution buffer and stored at –80°C. 16S profiling and MiSeq sequencing was performed as described earlier[7] with the following modifications: the V3-V4 region of the 16S gene was amplified using the dual barcoded primers 341F (GTGCCAGCMGCCGCGGTAA) and 806R (GGACTACHVGGGTWTCTAAT). Each primer contained additional sequences for a 12 base Golay barcode, Illumina adaptor and a linker sequence. PCR was performed using the Phusion Hot Start Flex 2X Master Mix (New England Biolabs, Frankfurt am Main, Germany) in a GeneAmp PCR system 9700 (Applied Biosystems/Thermo Fisher Scientific, Darmstadt, Germany) and the following program: 98°C for 3 min, 25x (98°C for 20 s, 55°C for 30 s, 72°C for 45 s), 72°C for 10 min, hold at 4°C. Performance of the PCR reactions was checked using agarose gel electrophoresis. Normalization was performed using the SequalPrep Normalization Plate Kit (Thermo Fisher Scientific, Darmstadt, Germany) following the manufacturer's instructions. Equal volumes of SequalPrep-normalized amplicons were pooled and the pool then handed to the Competence Centre for Genomic Analysis (Kiel, Germany) for sequencing on an Illumina MiSeq v3.0 (2 x 300 nt).

To characterize the microbiome composition, we first used the QIIME2 environment[8] (release 2021.4) for processing the reads, which are deposited and available at ENA under the accession code PRJEB61276 (https://www.ebi.ac.uk/ena/browser/view/PRJEB61276; last accessed on 11-Mar-2025). The paired-end sequences were filtered, denoised, merged and chimera-removed using the DADA2 plugin[9] (version 2021.2) implemented in QIIME2. After this step, the 16S rRNA gene amplicon sequencing reads retained on average 10,566 reads per sample. Where applicable, 16S rRNA gene sequence data were rarefied to a uniform sequencing depth of 5,000 reads, ensuring consistent coverage across samples for downstream analyses. In these downstream analyses, only samples with at least 5,000 reads were retained. Samples that did not meet this minimum threshold criterion were excluded.

To assess taxonomic assignments, we employed the QIIME2 scikit-learn classifier trained on the SILVA[10] (release 138) reference database (https://zenodo.org/records/6395539; last accessed on 11-Mar-2025).

Metagenome data processing

Shotgun metagenomics was performed at the Competence Centre for Genomic Analysis (Kiel, Germany). Metagenomes were processed using TOFU-MAaPO v1.2.2 (https://github.com/ikmb/TOFU-MAaPO; last accessed on 11-Mar-2025) that relies on the bioBakery 3 environment[2] (MetaPhlAn 3.0 and HUMAnN 3.0). Raw reads were quality-trimmed and mapped to the human genome (hg19) to discern between sequenced reads from the host (human) and the gut microbiome bacteria. Only samples containing >1 Gbp after trimming and host decontamination were kept in the analysis.

Taxonomic and functional potential analysis

The metagenomics analysis was performed using the general guidelines.[11] Where applicable, metagenomic sequence data were rarefied to a uniform sequencing depth of 1,000,000 reads, ensuring consistent coverage across

samples for downstream analyses. Taxonomic features and quantification of microbial communities' relative abundances on 72 samples were done by using MetaPhlAn 3.0[ref. 2] with default parameters. MetaPhlAn 3.0 relies on a database of clade-specific marker genes identified from ~13,500 bacterial/archaeal and ~3,500 viral genomes. Likewise, functional potential profiling (stratified pathways, gene families and enzyme categories) in the same samples was performed using HUMAnN 3.0,[ref 2] with default parameters. Functional potential analysis was based on MetaCyc v26.1 (https://metacyc.org/; last accessed on 11-Mar-2025).[12] All abundances were transformed to CPMs before statistical analysis.

Public cohort data

To further investigate if nicotinamide mitigates COVID-19-associated gut microbiota changes, we decided to explore the functional potential of stool samples of a longitudinal public cohort of COVID-19 individuals who were hospitalized in the Charité Universitätsmedizin Berlin.[13] We analyzed the longitudinal gut microbiota data of 15 healthy individuals, 19 patients with mild COVID-19 and 7 patients with severe COVID-19, from a total of 81 stool samples. All the fastq files were processed exactly as described above for our metagenomics samples.

Statistics

Most of the univariate and multivariate analyses were done in the R statistical software[14] (v.4.2.1) under phyloseq[15] (v.1.40.0), vegan[16] (v.2.6-2) and MAasLin2[ref. 17] (v.1.10.0).

Within-sample diversity (α-diversity) was explored by computing different diversity indexes (Chao1, Shannon, Inverse Simpson, Fisher, Observed Amplicon Sequence Variants [ASVs]) on ASV abundance data and finding differences among certain groups (Wilcoxon signed rank test or Kruskal-Wallis tests were performed).

For cross-sectional comparisons of microbial diversity between intervention groups, we used the two-sided Wilcoxon rank-sum test.

To assess the effect of time of microbial diversity, we fitted linear mixed-effects models using the lme4 package in R (v1.1-34). The response variable was modeled as a function of time, with subjects as random intercepts to account for repeated measures. Furthermore, a likelihood ratio test was used to compare the full model (Diversity index~ time + (1|Subject)) with a null model (Diversity index~ 1 + (1|Subject)). Significance was determined based on the chi-square statistic (χ^2) and associated P value.

Between-sample diversity (β-diversity) and the differences between intervention groups were explored and visualized in a distance-based redundancy analysis (db-RDA) plot. Quantification was performed by analyzing Aitchinson distances on centered log ratios from ASVs abundance data. Associations of microbiome composition to external covariates were tested with the implementation of PERMANOVA models (using adonis2 function from the vegan package). The P and R^2 values were determined by 10,000 permutations using Intervention Group, Age, and key COVID-19-related symptoms as covariables in the model.

To detect differences in changes of microbial features, taxonomic (16S rRNA) or pathways (shotgun sequencing) between the intervention groups over time, we built linear mixed models in the MaAslin2 package[17] where we adjusted for BMI and sex as fixed effect variables, and individual and age group as random effect variables. The final model used for this analysis is as follows: Feature Abundances ~ Intervention + Time + BMI + Sex + (1|Individual) + (1|Age Group). P values were corrected for multiple hypothesis testing using the Benjamini-Hochberg procedure, and a false discovery rate (FDR) <0.25 was defined as the significant threshold.

To detect differences in changes of pathways in the public dataset from Essex et al.,[13] we built a similar linear mixed model in which we included BMI, sex, WHO ordinal scale of clinical improvement (OSCI) class, visits and antibiotic usage at sampling day as fixed effect variables and individual and age group as random effect variables. The final model used for this analysis is as follows: Feature Abundances ~ OSCI Class + Visit + BMI + Sex + AbxSamp + (1|Individual) + (1|Age Group). We utilized this model to identify differentially abundant pathways between OSCI classes (Healthy vs. Mild, Healthy vs. Severe) both at the onset of and during the hospitalization period in COVID-19 patients. The pathways identified as differentially abundant between OSCI classes in the Essex et al.[13] cohort were subsequently compared to those observed between nicotinamide and placebo groups in our cohort.

To understand the major sources of variation in our microbiome data at the genus level, we applied variancePartition[18] v.1.26.0, which uses linear mixed models to compute the attributable percentage of variation of a feature based on selected covariates (individual, age, body mass index, fever prior to baseline, nicotinamide intervention, COVID-19-specific symptoms).

3.6. Sample size and futility analysis in COVit-2

Data available from the literature available at the beginning of the COVit-2 trial and results of the COVit-1 pilot trial (see section 1 of this Supplement) suggested that 40% of symptomatic patients with COVID-19 become symptom-free during the spontaneous course of the disease within two weeks. An increase of the event rate of becoming symptom-free to 50% (relative risk 1.25) through the use of nicotinamide at a 5% level of significance and 80% power resulted in a required sample size of at least 385 patients per group (for details, see section 6 of this Supplement). With an assumed drop-out rate of 10%, 420 patients per group (assigned in a 1:1 ratio) were to be included. Based on the results of the futility analysis after 400 patients, the Data Management Board (DMB) of the COVit-2 trial recommended (1) to keep the previously planned sample size of approximately 840 patients and (2) to base the efficacy analysis on patients with at least one risk factor for developing severe COVID-19 (RFITT, see section 3.4 of this Supplement). To ensure that the subpopulation recruited after the futility analysis also included an adequate proportion of such patients, the frequencies of risk factors were monitored in a blinded manner and 900 patients were recruited as a safeguard against possibly increased dropout rates. The Principal Investigator and the blinded trial team members remained blinded until the end of the trial and received only the recommendation of the DMB.

4. Supplementary references

1. Yeoh, Y.K. et al. Gut microbiota composition reflects disease severity and dysfunctional immune responses in patients with COVID-19. *Gut* **70**, 698-706 (2021).
2. Beghini, F. et al. Integrating taxonomic, functional, and strain-level profiling of diverse microbial communities with bioBakery 3. *Elife* **10**, e65088 (2021).
3. Maifeld, A. et al. Fasting alters the gut microbiome reducing blood pressure and body weight in metabolic syndrome patients. *Nat. Commun.* **12**, 1970 (2021).
4. EFSA. Scientific Opinion on Dietary Reference Values for niacin (EFSA Panel on Dietetic Products, Nutrition and Allergies). *EFSA J.* **12**, 3759 (2014).
5. Bahmer, T. et al. Severity, predictors and clinical correlates of Post-COVID syndrome (PCS) in Germany: A prospective, multi-centre, population-based cohort study. *EClinicalMedicine* **51**, 101549 (2022).
6. WHO. R&D Blueprint: novel Coronavirus - COVID-19 Therapeutic Trial Synopsis. https://www.who.int/publications/i/item/covid-19-therapeutic-trial-synopsis (last accessed on 11-Mar-2025) (2020).
7. Moltzau Anderson, J. et al. NOD2 Influences Trajectories of Intestinal Microbiota Recovery After Antibiotic Perturbation. *Cell. Mol. Gastroenterol. Hepatol.* **10**, 365-389 (2020).
8. Bolyen, E. et al. Reproducible, interactive, scalable and extensible microbiome data science using QIIME 2. *Nat. Biotechnol.* **37**, 852-857 (2019).
9. Callahan, B.J. et al. DADA2: High-resolution sample inference from Illumina amplicon data. *Nat. Methods* **13**, 581-583 (2016).
10. Quast, C. et al. The SILVA ribosomal RNA gene database project: improved data processing and web-based tools. *Nucleic Acids Res.* **41**, D590-596 (2013).
11. Quince, C., Walker, A.W., Simpson, J.T., Loman, N.J. & Segata, N. Shotgun metagenomics, from sampling to analysis. *Nat. Biotechnol.* **35**, 833-844 (2017).
12. Caspi, R. et al. The MetaCyc database of metabolic pathways and enzymes - a 2019 update. *Nucleic Acids Res.* **48**, D445-D453 (2019).
13. Essex, M. et al. Gut microbiota dysbiosis is associated with altered tryptophan metabolism and dysregulated inflammatory response in COVID-19. *NPJ Biofilms Microbiomes* **10**, 66 (2024).
14. Ihaka, R. & Gentleman, R. R: a language for data analysis and graphics. *J. Comput. Graph. Stat.* **5**, 299-314 (1996).
15. McMurdie, P.J. & Holmes, S. phyloseq: an R package for reproducible interactive analysis and graphics of microbiome census data. *PLoS One* **8**, e61217 (2013).
16. Dixon, P. VEGAN, a package of R functions for community ecology. *J. Veg. Sci.* **14**, 927-930 (2003).
17. Mallick, H. et al. Multivariable association discovery in population-scale meta-omics studies. *PLoS Comput. Biol.* **17**, e1009442 (2021).
18. Hoffman, G.E. & Schadt, E.E. variancePartition: interpreting drivers of variation in complex gene expression studies. *BMC Bioinformatics* **17**, 483 (2016).

5. Trial protocols

This section contains

- the English translation of the protocol of the COVit-2 trial (18 pp.) and
- the original German trial protocol of the COVit-2 trial (19 pp.)

in the final version 2.3 of 13 December 2021

and

- the English translation of the protocol of the COVit-1 trial (12 pp.) resulting in the pilot study and
- the original German trial protocol of the COVit-1 trial (13 pp.)

in the final version 1.1 of 26 March 2020.

CLINICAL TRIAL PROTOCOL

Improvement of the Nutritional Status Regarding Nicotinamide (Vitamin B3) and the Disease Course of COVID-19

Trial Code: COVit-2

Version: 2.3

Date: 13 December 2021

PI: Prof. Dr. Stefan Schreiber

Department of Internal Medicine I

University Medical Center Schleswig-Holstein, Campus Kiel

Arnold-Heller-Strasse 3, Building K1

24105 Kiel

Germany

Table of Contents

1. Summary of the Project
2. Responsibilities
3. Scientific Background
4. Project Objectives
    4.1. Primary Objective and Hypothesis
    4.2. Secondary Objectives
    4.3. Exploratory Objectives und Questions
5. Endpoints
6. Trial Design
7. Trial Population
8. Trial Procedures
    8.1. Informed Consent
    8.2. Measures (Intervention/Control) and Target Sizes
    8.3. Trial Duration
9. Risk-Benefit Assessment
10. Sample Size Calculation and Statistics
11. Futility Analysis and Sample Size Adaptation
12. Data Management, Data Protection and Handling of Biomaterials
13. Data Verification
14. Protocol Changes after Trial Start
15. Ethical Aspects
16. Subject Insurance
17. Publication Rules
18. Signature of the Principal Investigator
19. Appendix
    19.1. Patient Information
    19.2. Setup of the Online Informed Consent
    19.3. Case Report Form (CRF)
    19.4. Questionnaire Fatigue (FACIT-F)
    19.5. Questionnaire Quality of Life (SF-36)
    19.6. Additional Questionnaires for the Follow-Up after 6 Months
    19.7. Additional Informed Consent for the Smell Test, Additional Questionnaires and Cognitive Test in the Follow-Up after 6 Months
    19.8. Additional Informed Consent for Blood Sampling for Pharmacokinetics
    19.9. Additional Informed Consent for the Antibody Test

1 Summary of the Project

Based on the literature, it seems likely that a nutritional intervention with nicotinamide (a form of vitamin B3) can support the therapy of SARS-CoV-2 infection (COVID-19) by, *e.g.*, improving the availability of tryptophan and its metabolism-supporting metabolites (e.g. NAD). A pilot phase of the COVit trial (COVit-1) surprisingly showed a significant effect of nicotinamide on the time to complete resolution of COVID-19 symptoms. In addition, diarrhea is a common symptom of COVID-19. Therefore, in a second part of the trial, 420 symptomatic patients each with confirmed SARS-CoV-2 infection shall self-administer 1,000 mg nicotinamide (500 mg conventional nicotinamide and 500 mg nicotinamide released in a controlled manner in the intestine) or corresponding placebos per day in a blinded fashion for 4 weeks. The primary endpoint of the trial is the occurrence of individual COVID-19 symptoms at week 0, week 2 (primary analysis time point), week 4 and week 6, and after 6 months. Secondary endpoints are the severity of COVID-19 symptoms, the occurrence and severity of symptoms at the 6-month follow-up (post-COVID-19 syndrome, PCS), the levels of antibodies against N-protein and S-protein of SARS-CoV-2 after at least 6 months, the complete resolution of symptoms at 2, 4 and 6 weeks, and the time from diagnosis to resolution of individual or all symptoms. Exploratory endpoints will include the WHO clinical scale for COVID-19 and any development of severe COVID-19 (emergency department examination, hospitalization with at least 24 hours of oxygen requirement, intensive care requirement, ventilator requirement, or death), changes in fatigue and quality of life, and various biomarkers. Patients are recruited after positive testing, receive the information for informed consent and can declare their participation via a website. After randomized distribution of the trial medication, the patients will be asked about their disease course in telephone interviews at baseline (week 0) and after 2, 4 and 6 weeks. Stool samples will be collected from up to 400 patients at week 0, week 2, week 4, week 6 and after 6 months. In addition, in up to 20 selected patients, various inflammatory markers and the metabolome, in particular tryptophan metabolism, are examined in addition to the blood count and standard blood profile. In these patients, the viral strain is also determined by sequencing from nasopharyngeal swabs. In selected patients, in addition to the blood parameters mentioned above, the pharmacokinetics of nicotinamide, nicotinic acid and nicotinuric acid as well as metabolites of nicotinamide and tryptophan metabolism will be examined in the first 48 hours after a single administration of the trial interventions. In the stool, the changes in microbiome (in 100–300 patients) as well as metagenome and metabolome (in a subgroup) will be analyzed. The trial aims to generate rapid results on whether nicotinamide supplementation can alleviate the disease course of COVID-19. In addition, a follow-up interview, a smell test, a cognition test validated for telephone interviews (T3MS) and a test for SARS-CoV-2 antibodies after at least 6 months will be used to investigate whether such supplementation has an impact on the PCS as well as the immune response and, if applicable, vaccination against SARS-CoV-2.

2 Responsibilities

Principal Investigator of the trial:

Prof. Dr. med. Stefan Schreiber
Department of Internal Medicine I
University Medical Center Schleswig-Holstein (UKSH), Campus Kiel
Arnold-Heller-Strasse 3, Building K1, 24105 Kiel, Germany
Tel.: +49-431-500-15101, e-mail: s.schreiber@mucosa.de

Responsible centers and contacts:

Trial centers:

Department of Internal Medicine I, UKSH, Campus Kiel.

For recruitment, a large number of medical practices, general hospitals or testing sites/laboratories in Germany that treat COVID-19 patients will be used.

Participating trial laboratories:

Institute of Clinical Chemistry, UKSH, Campus Kiel: blood analyses.
Fraunhofer ITEM (Hannover, Germany), MVZ Dr. Eberhard & Partner (Dortmund, Germany) and the Medical Center of the University of Munich (Germany): blood analyses.
Institute of Clinical Molecular Biology, Kiel University and UKSH, Campus Kiel: microbiome, metagenome and metabolome analyses in stool; blood analyses, SARS-CoV-2 sequencing.
Adversis Pharma GmbH (Leipzig, Germany): antibody analyses in dried blood spot samples.

Telephone interviews:

Competence Network Intestinal Diseases e.V., Kiel (Germany).

Data management and statistics:

Competence Network Intestinal Diseases e.V., Kiel (Germany): data management and statistics.
Novustat AG, Wollerau (Switzerland): external statistics (particularly futility analysis).

Biobanking and sample management:

Institute of Clinical Molecular Biology, Kiel University and UKSH, Campus Kiel.

Funding:

The trial is funded by the Department of Internal Medicine I (UKSH, Campus Kiel), by start-up funding from the state of Schleswig-Holstein (project DOI30) and by the Cluster of Excellence PMI. The trial protocol was registered in the German Register of Clinical Trials (DRKS) prior to the start of recruitment and will be updated accordingly following a positive assessment. In parallel, the trial is registered at ClinicalTrials.gov.

3 Scientific Background

The availability of tryptophan and its metabolites, especially nicotinamide (a form of vitamin B3) as a building block of NAD and NADP, are important factors in chronic systemic inflammation. Here, an evolutionarily ancient defense mechanism can be misdirected by the body restricting energy use in cells analogous to iron reduction during inflammation (Boergeling & Ludwig 2017, FEBS J. 284:218). While this makes sense in simple organisms, such mechanisms lead to dysfunctions (e.g. inflammation-induced anemia) in complex organisms. Reduced levels of nicotinamide and thus NAD(P) compromise the immune defense against coronaviruses (Heer et al. 2020, J. Biol. Chem. 295:17986) and cell metabolism, especially of macrophages and epithelial cells. The latter in particular are subject to a critically high rate of renewal in the gut and lungs. In addition, kynurenine, as the main degradation product of tryptophan, can also have a strong immune-activating effect. Another link between SARS-CoV-2 beyond inflammation and the immune system could also be the SARS-CoV-2 entry receptor ACE2, as the presence of ACE2 on the cell surface allows uptake of tryptophan via the receptor B0AT1. In severe courses of influenza, as in chronic inflammation, increased tryptophan degradation and thus increased kynurenine levels are observed, and inhibition of tryptophan degradation has beneficial effects in animal models (Pizzini et al. 2019, Influenza Other Respir. Viruses 13:603; Boergeling & Ludwig 2017, FEBS J. 284:218). In turn, there is evidence in a wide variety of virus types that nicotinamide can reduce viral replication and support the body's defense mechanisms, e.g. in vaccinia (Child et al. 1988, Virus Res. 9:119), HIV (Murray 2003, Clin. Infect. Dis. 36:453), enteroviruses (Moell et al. 2009, J. Med. Virol. 81:1082) or hepatitis B (Li et al. 2016, Arch. Virol. 161:621). Sufficient supply of B vitamins and particularly nicotinamide to strengthen the immune system is also recommended to combat SARS-CoV-2 infection (Zhang & Liu 2020, J. Med. Virol. 92:479; Gharote 2020, Ind. J. Med. Sci. 72:25; Shakoor et al. 2021, Maturitas 144:108). Here, the improvement of the defense against secondary bacterial infections in disease models is particularly emphasized (Zhang & Liu 2020, J. Med. Virol. 92:479). The reduced oxygen saturation observed in one study at a dose of 400 mg/kg (this would correspond to an arguably toxic dose of 28 g in a 70-kg person) has no significance for a dietary intervention with 1,000 mg <u>total</u> dose and given the acceptable daily intake of 900 mg/day (EFSA Panel on Dietetic Products, Nutrition and Allergies 2014, EFSA J. 12:3759). In the COVit trial, 1,000 mg are administered, which is far below potentially harmful doses of several grams per day and very close to the ADI [OECD-SIDS: 3-pyridinecarboxamide (nicotinamide), SIDS Initial Assessment Report for SIAM 15, Boston, Massachusetts, 22-25 October 2002]. Meanwhile, several publications suggest the use of nicotinamide in COVID-19, but there are no trial data available (Shi et al. 2020, Cell Death Differ. 27:1451; Gharote 2020, Ind. J. Med. Sci. 72:25; Mehmel et al. 2020, Nutrients 12:1616; Shakoor et al. 2021, Maturitas 144:108; Heer et al. 2020, J. Biol. Chem. 295:17986).

In the pilot phase of the COVit trial (COVit-1), at first n=8 patients each received 1,000 mg of conventional nicotinamide or 245 mg of silica as placebo. During a quality control of the data collection, it was surprisingly observed that 4 of 8 patients (50%) in the nicotinamide group were already completely symptom-free after two weeks of administration, whereas this was the case in only one of the 8 patients in the control group. The evaluation of the COVit pilot trial with n=28 per group showed a significant reduction of the time to complete resolution of symptoms, which was driven by effects in female patients. From the literature, depending on the patient population, very long convalescences were also to be expected, e.g.

- 43% of patients were symptom-free at 14-21 days after positive SARS-CoV-2 test (Tenforde et al. 2020, MMWR 69:99),

- <30% without respiratory symptoms after 1 month (Marshall 2020, Nature 585:339),
- 32% symptom-free at 30 days and still only 34% at 60 days after SARS-CoV-2 diagnosis (Carvalho-Schneider et al. 2021, Clin. Microbiol. Infect. 27:258), or
- only 12.6% symptom-free after a mean of 60 days (Carfi et al. 2020, JAMA 324:604).

Moreover, since the start of the originally planned COVit trial, COVID-19 has been shown to be a systemic disease with an unexpectedly high frequency of gastrointestinal symptoms (Mitsuyama et al. 2020, J. Clin. Med. 9:3630). Recently, it has been published that the gut microbiome is also significantly and, in some cases, negatively affected by COVID-19 (Yeoh et al. 2021, Gut 70:698). Therefore, in the main phase of the trial (COVit-2), two different tablets, each containing 500 mg nicotinamide, will now be used as investigational drugs: the conventional immediate-release nicotinamide tablets from the pilot phase and the UKSH proprietary development CICR-NAM (controlled-ileocolonic-release nicotinamide), whose tablets release nicotinamide in a delayed and continuous manner starting in the lower small intestine. This is expected to increase the gastrointestinal beneficial effects of nicotinamide, specifically also on the microbiome and its interaction with the gut, as conventional nicotinamide is very rapidly absorbed into the circulation (Fangmann et al. 2018, Diabetes Care 41:398). Previous experiments with a CICR-NAM prototype have shown that systemic exposure from the new dosage form will be lower than in the pilot phase of the study (Fangmann et al. 2018, Diabetes Care 41:398), in which exposure was already close to the ADI anyway (see above). This will further reduce the already minimal risk of side effects.

The scientific background has evolved during the course of the study and has led to an adjustment of endpoints and analyses as part of the planned futility analysis and sample size review. This is explained in detail in Section 11.

4 Project Objectives

4.1 Primary Objective and Hypothesis

The primary objective of the trial is to investigate the hypothesis that COVID-19 patients lose individual COVID-19 symptoms more rapidly under real-world conditions if they supplement 1,000 mg of nicotinamide (500 mg of conventional nicotinamide and 500 mg of CICR-NAM).

4.2 Secondary Objectives

In addition to the primary objective mentioned above, the secondary objectives of the trial are to investigate the following parameters in more detail: severity of individual symptoms, occurrence and severity of symptoms at the 6-month follow-up (PCS), levels of antibodies against the N-protein and S-protein of SARS-CoV-2 after at least 6 months, complete resolution of symptoms at 2, 4, and 6 weeks, and time from diagnosis to freedom from individual or all symptoms.

4.3 Exploratory Objectives and Questions

The WHO clinical scale for COVID-19 and a development of severe COVID-19 (examination in an emergency department, hospitalization with at least 24 hours of oxygen requirement, intensive care requirement, ventilator requirement, or death) will be investigated in an exploratory fashion. Changes in fatigue and quality of life will be assessed using validated questionnaires (FACIT-F and SF-36). Further exploratory analyses in up to 20 selected patients will provide information on blood count, standard blood profile, various inflammatory

markers and the metabolome in the blood, in particular tryptophan metabolism. In these patients, the viral strain will also be determined by sequencing from nasopharyngeal swabs. In selected patients, detailed pharmacokinetics may follow. In the stool, changes in the microbiome (in 100–300 patients) as well as in the metagenome and metabolome (in a subgroup) will be analyzed.

5 Endpoints

The disease course of the nicotinamide-supplemented group and the placebo group will be compared using the following endpoints:

Primary endpoint:

Frequencies of individual COVID-19 symptoms at week 0, week 2 (primary time of analysis), week 4 and week 6 as well as after 6 months.

Secondary endpoints:

1. Severity of individual COVID-19 symptoms at week 0, week 2 (primary time of analysis), week 4 and week 6 as well as after 6 months.
2. As part of the 6-month follow-up: frequency and severity of symptoms characteristic of post-COVID-19 syndrome.

   For this purpose, a validated smell test (Smell Identification Test™; Sensonics / MediSense) is performed by the patient. Furthermore, in addition to the FACIT-F and SF-36 questionnaires (as during the actual study period from week 0 to week 6), additional questionnaires (see Appendix 19.6.) will be completed on olfactory and gustatory abilities (incl. Questionnaire of Olfactory Disorders, QOD), on respiration (Multidimensional Dyspnea Profile, MDP), on mental state (Patient Health Questionnaire Depression, PHQ-8; Generalized Anxiety Disorder 7, GAD-7; Perceived Stress Scale, PSS; Brief Resilience Scale, BRS), on sleep quality (Pittsburgh Sleep Quality Index, PSQI), and on fatigue (Multidimensional Fatigue Inventory, MFI). In addition, a cognition test validated for telephone interviews (T3MS) will be performed.
3. Levels of antibodies directed against the N protein or S protein of SARS-CoV-2 after at least 6 months (anti-S protein stratified for presence and type of booster vaccination).
4. Complete symptom resolution after 2 weeks.
5. Complete symptom resolution after 4 weeks.
6. Complete symptom resolution after 6 weeks.
7. Time from diagnosis to resolution of individual symptoms (in days, up to 6 weeks).
8. Time from diagnosis to complete symptom resolution (in days, up to 6 weeks).

The following symptoms are queried in telephone interviews and explored individually in their course: Fatigue / tiredness / exhaustion / lack of strength, drop in performance / limited physical performance, fever (up to 39 °C or above), chills, shortness of breath, whistling or wheezing breathing, pneumonia, cough (with or without sputum production), rhinitis / runny nose / nasal mucus production / rhinorrhea, sore throat / pharyngitis or scratchy throat, hoarseness, muscle pain (myalgia), joint pain (arthralgia), limb pain, chest pain, headache,

abdominal pain, diarrhea, nausea, vomiting, loss of appetite / decreased food intake, other gastrointestinal symptoms (to be named individually by the patient), impaired sense of smell, impaired sense of taste, impaired consciousness / confusion, dizziness, conjunctivitis, skin rash, hair loss, and other symptoms (to be named individually by the patient).

In a subgroup of patients, daily changes in the severity of individual symptoms during the first 4 weeks are investigated in an exploratory fashion; gradations of symptoms: 0 = not at all, 1 = mild, 2 = moderate, 3 = severe, 4 = intolerable.

In addition, a complaint scale for lower respiratory tract infections is queried with the aspects of cough, mucus production, shortness of breath, sleep, ability to perform normal activities, and general feeling of illness with the following gradations: 0 = normal, 1 = very minor problem, 2 = minor problem, 3 = moderately poor, 4 = poor, 5 = very poor, 6 = maximally poor.

Exploratory endpoints:

1. World Health Organization (WHO) COVID-19 Ordinal Scale for Clinical Improvement (see https://www.who.int/publications/i/item/covid-19-therapeutic-trial-synopsis) at week 0 (baseline), week 2, week 4 and week 6 as well as after 6 months.
2. Occurrence of severe COVID-19, defined as achieving one of the following characteristics:
    a. examination in an emergency department;
    b. hospitalization with continuous oxygen requirement of at least 24 hours;
    c. intensive care requirement;
    d. ventilation requirement;
    e. death by COVID-19.
3. Comparison between findings from the pilot phase of the trial and the second part of the trial.
4. Changes in fatigue (FACIT-F questionnaire).
5. Changes in quality of life (SF-36 questionnaire).
6. Changes in blood levels of tryptophan (in selected patients).
7. Changes in blood levels of tryptophan metabolites (in selected patients).
8. Changes in blood levels of inflammatory markers (C-reactive protein, interleukin-6, ferritin, neopterin, D-dimers) (in selected patients).
9. Changes in blood count and standard blood profile (in selected patients).
10. Changes in blood metabolome composition (in selected patients).
11. Strain of SARS-CoV-2 virus (in selected patients).
12. Changes in stool microbiome composition (in selected patients).
13. Changes in stool metagenome composition (in selected patients).
14. Changes in stool metabolome composition (in selected patients).
15. Pharmacokinetics (in selected patients).

6 Trial Design

This is a monocentric, randomized trial in which symptomatic COVID-19 patients receive dietary supplementation with 1,000 mg nicotinamide per day in tablet form (1 x 500 mg NicoPel, IFC Germany/Derma Enzinger, and 1 x 500 mg CICR-NAM, NextPharma/UKSH) or matching placebo tablets (Fagron and NextPharma/UKSH, respectively). In the pilot phase, the nicotinamide group received 2 x 500 mg NicoPel and the control group received 245 mg silica in a capsule (1 x 245 mg silica capsule, Twardy/Saluspharma). The less than perfect pairing of verum and placebo-like control was justified by the market availability of the investigational products and the tremendous urgency of the trial. It is assumed that the comparator group “remedy silica” had an effect size comparable to the placebo treatment, since silica consists mainly of inert silicon dioxide. However, as positive statements on the nutritional benefit have been established for silica as advertizable evaluations, the placebo was optimized for the second part of the trial. Double blinding was and is ensured as far as possible. For the patients, it is not clear from the packaging and intake instructions of the dietary supplement sent to them which investigational product they received. Data collection personnel on the telephones are instructed by SOP not to discuss the type of investigational product and are blinded as to the group allocation of the trial participants they are calling. All patients receive the usual clinical standard of care, and the nutritional supplement is purely additive to the standard of care.

7 Trial Population

The trial population to be investigated, totaling up to approximately 840 trial participants, involves patients with confirmed SARS-CoV-2 infection and symptoms, e.g. in the respiratory and/or gastrointestinal tract.

Inclusion criteria are:

- SARS-CoV-2 infection confirmed by laboratory findings; the positive test must not date back more than 7 days.
- Relevant infection symptoms, e.g. in the respiratory or gastrointestinal tract.
- The patient has been able to give written consent via a website before any trial procedure is performed and can comply with the trial-dependent prerequisites and requirements.
- The patient is of age (at least 18 years).

Exclusion criteria are:

- Current participation in another trial.
- Pregnancy or breastfeeding.
- Vaccination against SARS-CoV-2.

Recruitment will take place in Kiel, Germany, and in other physician offices, hospital centers, and testing sites/laboratories for SARS-CoV-2 in Germany.

8 Trial Procedures

8.1 Informed Consent

Treatment decisions for COVID-19 are outside the trial protocol, i.e. the indication is independent and established without influence by the research program. Only after the indication has been established, a decision is made regarding possible recruitment.

All patients in the respective centers (outpatient and inpatient) who meet all inclusion and exclusion criteria will be asked to participate in the trial. There will be no trial-specific procedure outside the clinical standard in screening. Patients will receive a written patient information with informed consent. If necessary, a hotline is available for queries and a medical consultation for informed consent. After sufficient time for consideration, the patient can then consent online and document his or her consent by printing out the relevant page for him or herself (subject consent). This procedure also allows patients in quarantine at home to participate. Only after online consent, the first telephone contact is made for data collection for week 0 (baseline), wherein at the beginning the patient's ability to consent is checked again by study staff on the telephone according to an SOP. Again, the patient can ask further questions and the medical hotline can be consulted.

Supplemental written informed consent will be obtained from patients for the olfactory test, the additional questionnaires, the cognition test, and the test for SARS-CoV-2 antibodies at the 6-month follow-up, as well as for the pharmacokinetic measurements.

8.2 Measures (Intervention/Control) and Target Sizes

After consent and fulfillment of all requirements, patients are randomly assigned to the nicotinamide arm or the placebo arm. The patient will be informed about this.

After randomization in a 1:1 ratio, control/interview calls are planned at week 0 (baseline) as well as week 2, week 4 and week 6. After approximately 6 months, another follow-up call is planned to clarify long-term symptoms. Together with the tablets, patients will receive printed questionnaires to characterize fatigue (FACIT-F) and quality of life (SF-36), which they should fill out themselves and return by prepaid envelope. In this context, wearables analogous to vote A 101/20 may also be used in perspective; this would then be specified and presented in more detail. Up to 400 patients will receive sample containers for stool samples, which will be sent to the IKMB via return envelope. The aim is to obtain 100–300 sample series for stool microbiome analyses, of which a representative portion will be additionally analyzed for changes in the stool metagenome and stool metabolome. In an expected number of up to 20 patients from Kiel and the surrounding area (up to 10 patients each with verum or placebo), additional blood samples will be taken at the interview time points and at week 1 to perform the exploratory blood analyses (see endpoints). In these patients, the viral strain will also be determined by sequencing from nasopharyngeal swabs at baseline. In up to 5 selected patients from this group, the pharmacokinetics of nicotinamide, nicotinic acid and nicotinuric acid, as well as of metabolites of nicotinamide and tryptophan metabolism, will also be assessed at a later time point during the first 48 hours after a single administration of the trial products.

Furthermore, a follow-up interview, additional questionnaires, a smell test, a cognition test and a test for SARS-CoV-2 antibodies after 6 months will be used to investigate whether supplementation with nicotinamide has an influence on the PCS as well as the immune response and possibly vaccination against SARS-CoV-2. The information obtained here is consistent (except for the additional antibody test) with the data collected in the COVIDOM trial and will allow for subsequent cross-linking of the two trials to be reviewed separately.

The telephone interviews are conducted by the Competence Network Intestinal Diseases e.V. For medical questions, a physician from the Department of Internal Medicine I calls back. The following parameters are queried or entered during the calls:

Week 0:

- Personal and demographic data
- COVID-19-relevant and other symptoms
- Inclusion criteria
- Comorbidities
- Smoking status
- Chronic medication and supplementation
- Hospitalization
- WHO scale for COVID-19
- Type of supplementation
- Epidemiological questions about living conditions

Weeks 2, 4 and 6:

- Intake of supplementation and other dietary supplements, if applicable.
- COVID-19-related and other symptoms
- Death and cause of death
- smoking status
- Examination in an emergency department
- Hospitalization
- WHO scale for COVID-19
- Ventilation requirement
- Intensive care treatment

The trial flow is summarized in the following flow chart:

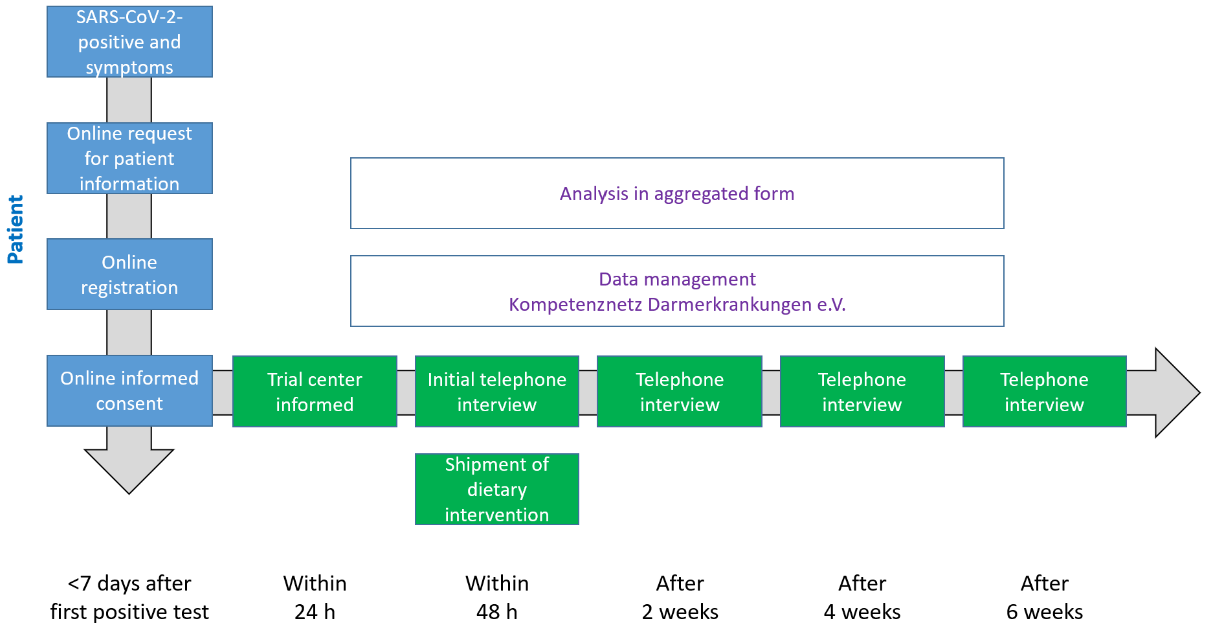

8.3 Trial Duration

The trial duration for the individual patient is regularly 4 weeks until the end of the trial, 6 weeks if symptoms persist. A final interview takes place after approximately 6 months. This is particularly aimed at quality of life and possible late effects and also includes the EQ-5D-5L questionnaire (in the eCRF) as well as further paper questionnaires, an olfactory test and a cognition test (see section 5) analogous to the COVIDOM trial, in which the patients of the COVit trial can also be included. In addition, patients may have a test performed for SARS-CoV-2 antibodies. The total duration of the trial will depend on the recruitment success and the further course of the COVID-19 epidemic. Enrollment has started on 01 February 2021. We anticipate enrolment of the last patient in late 2021 / early 2022.

9 Risk-Benefit Assessment

If the optimization of the nicotinamide status helps at least some of the COVID-19 patients to overcome the disease more quickly and/or more easily, then – in the repeatedly dramatically worsening situation – every patient who is not hospitalized or requires ventilation and every patient who recovers completely more quickly is a gain personally (intrinsic benefit) and also in relation to the totality of all COVID-19 patients (group benefit) and other patients in the hospital, as well as in relation to the socioeconomic consequences (sick days, late effects). The other extraneous benefit is a gain in knowledge for clinical research (improvement of patient care by a simple, immediately available and safe nutritional supplement). Risks are not recognizable.

Patient supplementation is discontinued when a critical condition is reached and the patient is admitted to a monitoring area.

10 Sample Size Calculation and Statistics

The available data at the start of the COVit-2 trial suggested that at most 40% of the already symptomatic COVID-19 patients would be completely symptom-free two weeks after the start of the intervention (primary endpoint defined at that time). This would likely have been the case in the placebo-treated patient collective. The hypothesis was that this 40% event rate could be increased to 50% with the use of nicotinamide.

Statistical analysis is performed using SAS software version 9.3 or later (SAS Institute, Inc., Cary, North Carolina) or R. The statistical methodology is laid down in detail in a statistical analysis plan (SAP) in final form before the database lock. Any changes to the analysis plan in the protocol are documented in the SAP and presented in the final trial report. Continuous variables are summarized by descriptive statistics (number of observations (n), mean/median, standard deviation, minimum and maximum). Categorical variables are reported in frequency tables summarizing the number and percentage of patients in each category.

The intention-to-treat (ITT) population is defined as “all included patients”. The “all-randomized” (AR) population includes all patients who were observed for at least two weeks (intake of the dietary supplement and telephone interviews). The AR population is used to assess the primary and secondary endpoints. The biomarker (BM) population includes all patients from whom biomaterials could be obtained.

For sample size calculation, the standard variables were assumed to be: α-error = 0.05; power = 0.8; resulting in a K = 7.85 (factor calculated from α-error and power). The following simplified formula was used:

$$n = \frac{K[(R+1) - p_2(R^2+1)]}{p_2(1-R)^2}$$

n = required case number

p1 = Event rate in the treatment group (50%)

p2 = Event rate in the control group (40%)

R = relative risk (p1/p2) = here: 50% / 40% = 1.25

As a result, 385 patients per group were required. Based on previous experience, just under 10% were estimated as a safety margin for dropouts, therefore 420 patients per group should be included. If necessary, the case number calculation should be adjusted on the basis of the blinded event rate in the study.

The primary endpoint is analyzed using a generalized linear model with log-link and binomial error distribution. Results are presented as relative risk with 95% confidence interval. Binary secondary endpoints are analyzed using logistic regression models. Ordinal secondary endpoints (e.g. symptom severity) will be analyzed with proportional odds models with treatment group as the independent variable. In addition, stratifications will be made according to important prognostic factors such as symptom severity or specific symptoms at week 0 (baseline). The details are laid down in the SAP.

Based on the results of the futility analysis, the Data Management Board (DMB) recommended to the study management to maintain the previously planned enrollment size of approximately 840 patients and to select the subgroup of patients with at least one risk factor for severe COVID-19 progression as the primary clinical analysis population (see Section 11). To ensure that an adequate proportion of such patients is also included in the subpopulation recruited after futility analysis, the frequencies of risk factors will be monitored in a blinded manner and up to 10% more patients will be recruited as a safeguard against possible clustering of study dropouts.

11 Futility Analysis and Sample Size Adaptation

After 400 completed patients (with data collection including week 6), the frequency of the primary endpoint was assessed in the blinded data. When designing the COVit-2 study, it was expected that approximately 45% of patients should be symptom-free after 2 weeks of supplementation. If necessary, an interim evaluation and adjustment of the sample size with expansion or reduction of the number of patients should be performed.

For this planned futility analysis by the DMB of the COVit trial after collection of all data up to week 6 for 400 patients, a cleaned data set with n = 402 patients (201 each supplemented with nicotinamide or placebo, respectively) was available. In this data set, statistically significant positive effects of nicotinamide on certain subgroups were found by the external and independent statisticians of Novustat AG, which occurred most frequently and most strongly after 2 weeks.

For the patients recruited in the meantime, blinded and unadjusted frequencies of leading symptoms and affiliations to major risk groups were requested from the data management of the Competence Network Intestinal Diseases e.V. These indicated that the patient population from patient no. 403 onwards did not differ significantly from the one before. However,

because of the exclusion criterion of vaccination against SARS-CoV-2, increasing social selection is occurring.

The previous primary endpoint "complete resolution of symptoms after 2 weeks" and the corresponding secondary endpoints were based on the understanding of the disease course at the beginning of the COVit-2 trial and the, from today's perspective, unusual distribution of risk factors in the patient cohort of the pilot trial COVit-1. It is now clear that the populations of COVit-1 and COVit-2 differ and that the complete symptom resolution achieved comparatively often in COVit-1 occurs much less frequently in patients in COVit-2. This is true both for the overall population and for subgroups with COVID-19 leading symptoms such as cough at week 0. Moreover, the frequency of these leading symptoms is lower in patients in COVit-2 than in COVit-1, which reduces the signal amplitude. On the other hand, the longer duration of illness observed in COVit-2 is consistent with current literature data, also relating to PCS. Based on the advice of the DMB, the PCS should be considered more strongly than before in the evaluation of the COVit-2 trial, especially since a meaningful battery of tests and questionnaires was added in the course of the trial.

In addition, the designs and initial results of clinical trials with orally administered drug candidates such as molnupiravir and PAXLOVID™ should be considered. Similar to nicotinamide, these compounds are intended to be used immediately after diagnosis of SARS-CoV-2 infection to mitigate disease progression. Unfortunately, such candidates did not exist at the time COVit-2 was planned. However, the studies of molnupiravir and PAXLOVID™ have included only patients with at least one risk factor for severe disease progression because these appear to benefit particularly from early intervention. The COVit-2 population as a whole, on the other hand, shows a disease that is too mild and too prolonged to perform meaningful evaluations with the previous strategy.

Recent findings on NAD metabolism in mature B cells (Bernardes et al. 2020, Immunity 53:1296) also suggest that the immunological response to SARS-CoV-2 should be investigated in addition to symptom-based analysis in the acute and post-acute disease course. It is quite conceivable that supplementation with nicotinamide could support antibody formation during infection by increasing the availability of NAD. Appropriate measurements of antibody levels at approximately 6 months are already part of the 6-month follow-up and should be upgraded.

The DMB therefore drew the following conclusions in its deliberations:

1. The continuation of the trial was and is warranted because patients have no discernible disadvantages from the supplementation and certain groups benefit significantly from nicotinamide supplementation.

2. The previously planned recruitment size of approx. 840 patients should be maintained. Since significantly higher effect sizes than previously assumed could be observed in individual subgroups with regard to COVID-19 symptoms or risk factors, highly significant signals could thus probably be expected in the total population of $n \geq 800$ (with a corresponding reserve of approx. 5% for study dropouts, hence 840). Even if divided into a hypothesis-generating population of the first n = 402 and a validation population of approximately equal size, some significant results would be expected according to the findings in the first n = 402.

3. As this is not a trial according to AMG, as the prerequisites for the trial have changed considerably in view of the recruited population, and as the current state of knowledge on suitable populations for COVID-19 studies in non-hospitalized patients should be

considered, the subgroup of patients with at least one risk factor for a severe course of COVID-19 is recommended as the primary clinical analysis population. The total population of n ≥ 800 should serve only as a secondary analysis population.

4. It is recommended to convert the last two secondary endpoints (frequency and severity of symptoms) into the primary endpoint (frequency) and the first secondary endpoint (severity) and to maintain the endpoints regarding the time until complete resolution of symptoms as secondary endpoints. Furthermore, a focus on the frequency and severity after 2 weeks is recommended.
5. To adequately address the PCS, the DMB recommends upgrading the previously exploratory endpoint regarding the PCS into a secondary endpoint.
6. Based on current knowledge of B cell metabolism, the recommended immunological analysis population is the total population (n ≥ 800), and the secondary immunological analysis population should be the subgroup of patients with at least one risk factor for a severe course of COVID-19. For possible effects of booster vaccination, stratification must be performed in both populations.
7. The levels of antibodies against SARS-CoV-2 should be investigated as a secondary endpoint.

12 Data Management, Data Protection and Handling of Biomaterials

Medical data incl. routine clinical findings will not be pseudonymized in this trial. However, a pseudonymization of the patient for the trial with a trial patient ID takes place as soon as the data are released for evaluation. In this process, the association of the patient data with the trial patient ID does not leave the recruitment center. The recruitment center is operated by the Competence Network Intestinal Diseases e.V., which also supports the trial in data management and by providing trained trial personnel for telephone data acquisition. Outside the recruitment center, only trial patient IDs will be handled.

A web-based Case Report Form (CRF) database set up for this trial will collect the information listed in Section 19.3 of the Appendix. In this process, data entry is blinded.

The data of the blood and stool analyses as well as the virus sequencing are stored pseudonymized in a digital database at the Institute for Clinical Molecular Biology (Kiel). For the blood tests at UKSH, study cases are created in ORBIS. The data storage will of course take place behind the firewall of CAU/UKSH and with all appropriate security precautions. Since the project is under medical management, all employees are subject to medical confidentiality. Any disclosure of data to unauthorized third parties (especially employers, insurance companies) is excluded. The transfer of samples and information to scientific cooperation partners takes place exclusively in pseudonymized form, i.e. without personal details. At the end of the research activities (after 20 years at the earliest), the samples and the associated data are destroyed. At the patient's request, they will receive a copy with a detailed description of the data management at the Institute of Clinical Molecular Biology. This complies with the analytical standards developed by the IHEC (International Human Epigenome Consortium; *http://ihec-epigenomes.org*). Before analysis of biosamples, these are recoded and linked to a pseudonymized data set.

Participation in this clinical trial is absolutely voluntary. Consent can be revoked by the patient at any time. This does not result in any disadvantages for the patients. Any samples already taken will then be destroyed immediately, and the data (except for data already

published/analyzed) will be deleted immediately, as far as technically possible (deletion after evaluation is no longer possible, the patient will also be informed of this). Patients may address any revocation of consent in writing to the project management of the Department of Internal Medicine I, University Medical Center Schleswig-Holstein, Campus Kiel, Rosalind-Franklin-Str. 12, Building K1, D-24105 Kiel.

It is possible that patents may arise in the course of this research project. In this case, there is no individual patent claim of the patients based on their individual data or individual biological materials.

13 Data Verification

Since the results of the telephone contacts are entered directly into the database, no systematic monitoring takes place. Exploratory data are only collected centrally and are subject to the data processing standards at the Institute for Clinical Molecular Biology (Kiel) described in section 12. If necessary, additional data will be requested from the attending physicians.

14 Protocol Changes after Trial Start

Protocol Amendments

Any changes to this protocol will be documented in a Protocol Amendment and require the approval of the Principal Investigator. These Protocol Amendments will be communicated to the relevant ethics committees and, in the case of substantial changes, will be submitted for prior approval.

Protocol Deviations

Deviations from this protocol should be avoided. If a protocol deviation occurs, the Principal Investigator will order a data reconciliation and the consequences of the deviation will be reviewed and discussed. Any deviation will be documented in the study file.

Early Termination of the Trial

The Principal Investigator has the right to terminate the trial at any time. In this case, the Principal Investigator must ensure that further decision-making is in the best interests of the patients. Termination of the trial will be reported to the appropriate ethics committee. The trial may be terminated by the responsible clinicians if

- the continuation of the study is not reasonable from an ethical or medical point of view,
- the resources to continue the study are not sufficiently available, or
- a meaningful outcome does not appear likely.

Individual trial participants whose health and safety are at risk may be excluded from the trial by participating clinicians at their discretion.

15 Ethical Aspects

Independent Ethics Committee

The Independent Ethics Committee of the Medical Faculty of CAU Kiel will evaluate this protocol as well as the subject information and subject consent, their updates (if any) and any other material given to the patients in writing.

End of Trial and End-of-Trial Notification

The end of trial is defined as the date on which the last trial participant is contacted by telephone at their last trial visit. At the end of the trial, the responsible ethics committee is notified.

Ethical Conduct of the Trial

This clinical trial will be conducted according to ethical principles following the Declaration of Helsinki and according to this approved protocol.

16 Subject Insurance

No subject or commuting accident insurance will be taken out for the trial. It is explicitly stated in the patient information that there is no subject commuting accident insurance. According to information provided by the UKSH insurance broker (Marsh Medical Consulting) and the responsible insurer (Berkshire Hathaway Specialty Insurance) on 03 December 2020, the UKSH public liability insurance covers the (extremely small) risks arising from the use of the non-marketed CICR-NAM within the scope of the trial.

17 Publication Rules

After completion of the trial, one or more manuscripts will be prepared for joint publications. Authorship will be assigned according to ICMJE criteria (for an official version, see http://www.ICMJE.org). The author list will follow the respective guidelines of the relevant journals or congresses. Contract laboratories do not have publication rights with respect to this trial.

18 Signature of the Principal Investigator

Kiel, ______________________

____

Prof. Dr. med. Stefan Schreiber

19 Appendix

19.1 Patient Information

19.2 Setup of the Online Informed Consent

19.3 Case Report Form (CRF)

19.4 Questionnaire Fatigue (FACIT-F)

19.5 Questionnaire Quality of Life (SF-36)

19.6 Additional Questionnaires for the Follow-Up after 6 Months

19.7 Additional Informed Consent for the Smell Test, Additional Questionnaires and Cognitive Test in the Follow-Up after 6 Months

19.8 Additional Informed Consent for Blood Sampling for Pharmacokinetics

19.9 Additional Informed Consent for the Antibody Test

<u>KLINISCHES STUDIENPROTOKOLL</u>

Verbesserung des Ernährungsstatus bezüglich Nicotinamid (Vitamin B3) und Verlauf der COVID-19-Erkrankung

Studienkürzel:
COVit-2

Version: 2.3

Datum: 13. Dezember 2021

Projektleiter: Prof. Dr. Stefan Schreiber

Klinik für Innere Medizin I

Universitätsklinikum Schleswig-Holstein, Campus Kiel

Arnold-Heller-Straße 3, Haus K1

24105 Kiel

Inhaltsverzeichnis

1. Zusammenfassung des Projektes
2. Verantwortlichkeiten
3. Wissenschaftlicher Hintergrund
4. Projektziele
   4.1. Primärziel und Hypothese
   4.2. Sekundärziele
   4.3. Explorative Ziele und Fragestellungen
5. Endpunkte
6. Studiendesign
7. Studienpopulation
8. Studienablauf
   8.1. Aufklärung und Einwilligung
   8.2. Maßnahmen (Intervention/Kontrolle) und Zielgrößen
   8.3. Studiendauer
9. Nutzen-Risiko-Abwägung
10. Fallzahlberechnung und Statistik
11. Futility-Analyse und Anpassung der Stichprobengröße
12. Datenmanagement, Datenschutz und Umgang mit Biomaterialien
13. Datenverifizierung
14. Protokolländerungen nach Studienbeginn
15. Ethische Aspekte
16. Probandenversicherung
17. Publikationsregeln
18. Unterschrift des Studienleiters
19. Anhang
   19.1. Patienteninformation
   19.2. Aufbau der Online-Einwilligung
   19.3. Case Report Form (CRF)
   19.4. Fragebogen Fatigue (FACIT-F)
   19.5. Fragebogen Lebensqualität (SF-36)
   19.6. Zusatzfragebögen zur Nachbefragung nach 6 Monaten
   19.7. Ergänzende Einwilligung für den Riechtest, die Zusatzfragebögen und den Kognitionstest zur Nachbefragung nach 6 Monaten
   19.8. Ergänzende Einwilligung für Blutabnahmen zur Pharmakokinetik
   19.9. Ergänzende Einwilligung zum Antikörpertest

1 Zusammenfassung des Projekts

Aufgrund der Literatur erscheint es wahrscheinlich, dass eine Ernährungsintervention mit Nicotinamid (einer Form von Vitamin B3) die Therapie der SARS-CoV-2-Infektion (COVID-19) u.a. durch eine Verbesserung der Verfügbarkeit von Tryptophan und seiner stoffwechselunterstützenden Metaboliten (z.B. NAD) unterstützen kann. Eine Pilotphase der COVit-Studie (COVit-1) zeigte überraschend einen deutlichen Effekt von Nicotinamid auf die Zeit bis zum vollständigen Verschwinden der COVID-19-Symptome. Zudem ist Diarrhoe ein häufiges Symptom von COVID-19. Je 420 symptomatische Patienten mit bestätigter SARS-CoV-2-Infektion sollen daher in einem zweiten Teil der Studie für 4 Wochen verblindet pro Tag 1.000 mg Nicotinamid (500 mg herkömmliches Nicotinamid und 500 mg kontrolliert im Darm freigesetztes Nicotinamid) oder entsprechende Placebos einnehmen. Primärer Endpunkt der Studie ist das Auftreten einzelner COVID-19-Symptome zu Woche 0, Woche 2 (primärer Analysezeitpunkt), Woche 4 und Woche 6 sowie nach 6 Monaten. Sekundäre Endpunkte sind die Stärke von COVID-19-Symptomen, das Auftreten und die Stärke von Symptomen bei der 6-Monats-Nachverfolgung (Post-COVID-19-Syndrom, PCS), die Spiegel von Antikörpern gegen N-Protein und S-Protein von SARS-CoV-2 nach mindestens 6 Monaten, die vollständige Symptomfreiheit nach 2, 4 und 6 Wochen sowie die Zeit von der Diagnose bis zur Freiheit von einzelnen oder allen Symptomen. Als explorative Endpunkte werden u.a. die klinische WHO-Skala für COVID-19 und eine Entwicklung von schwerem COVID-19 (Untersuchung in einer Notaufnahme, Hospitalisierung mit mindestens 24 Stunden Sauerstoffpflicht, Intensivpflicht, Beatmungspflichtigkeit oder Tod), Änderungen bei Fatigue und Lebensqualität sowie verschiedene Biomarker untersucht. Die Patienten werden nach positiver Testung angesprochen, aufgeklärt und können über eine Webseite ihre Teilnahme erklären. Nach randomisierter Austeilung der Studienmedikation werden die Patienten zu Beginn (Woche 0) und nach 2, 4 und 6 Wochen in Telefoninterviews zu ihrem Krankheitsverlauf befragt. Bei bis zu 400 Patienten werden zu Woche 0, Woche 2, Woche 4, Woche 6 und nach 6 Monaten Stuhlproben gewonnen. Zusätzlich werden bei bis zu 20 ausgewählten Patienten neben Blutbild und Standardblutprofil verschiedene Entzündungsmarker und das Metabolom, insbesondere der Tryptophanstoffwechsel, untersucht. Bei diesen Patienten wird auch der Virenstamm durch Sequenzierung aus Nasen-Rachen-Abstrichen bestimmt. Bei ausgewählten Patienten wird neben den genannten Blutwerten auch die Pharmakokinetik von Nicotinamid, Nicotinsäure und Nicotinursäure sowie von Metaboliten des Nicotinamid- und Tryptophanstoffwechsels in den ersten 48 Stunden nach einer einmaligen Gabe der Studienpräparate untersucht. Im Stuhl werden die Veränderungen von Mikrobiom (bei 100–300 Patienten) sowie Metagenom und Metabolom (bei einer Subgruppe) analysiert. Die Studie soll schnelle Ergebnisse dahingehend produzieren, ob eine Supplementation von Nicotinamid den Krankheitsverlauf von COVID-19 lindern kann. Darüber hinaus soll durch eine Nachbefragung, einen Riechtest, einen für Telefonbefragungen validierten Kognitionstest (T3MS) und einen Test auf SARS-CoV-2-Antikörper nach mindestens 6 Monaten untersucht werden, ob eine solche Supplementation einen Einfluss auf das PCS sowie die Immunreaktion und ggf. Impfung gegen SARS-CoV-2 hat.

2 Verantwortlichkeiten

Studienleiter:

Prof. Dr. med. Stefan Schreiber
Klinik für Innere Medizin I
Universitätsklinikum Schleswig-Holstein (UKSH), Campus Kiel
Arnold-Heller-Straße 3, Haus K1, 24105 Kiel
Tel.: 0431-500-15101, Email: s.schreiber@mucosa.de

Verantwortliche Zentren und Kontakte:

Studienzentren:

Klinik für Innere Medizin I, UKSH, Campus Kiel.

Zur Rekrutierung wird eine Vielzahl von Arztpraxen, Schwerpunktkrankenhäusern oder Teststellen/-laboren in Deutschland eingesetzt, die COVID-19-Patienten behandeln.

Beteiligte Studien-Labore:

Institut für Klinische Chemie, UKSH, Campus Kiel: Blutanalysen.
Fraunhofer ITEM (Hannover), MVZ Dr. Eberhard & Partner (Dortmund) und Klinikum der Universität München: Blutanalysen.
Institut für Klinische Molekularbiologie, CAU und UKSH, Campus Kiel: Mikrobiom-, Metagenom- und Metabolomanalysen im Stuhl; Blutanalysen; SARS-CoV-2-Sequenzierung.
Adversis Pharma GmbH (Leipzig): Antikörperanalysen in Dried-Blood-Spot-Proben.

Telefonische Visiten:

Kompetenznetz Darmerkrankungen e.V., Kiel.

Datenhaltung und Statistik:

Kompetenznetz Darmerkrankungen e.V., Kiel: Datenhaltung und Statistik.
Novustat AG, Wollerau (CH): Externe Statistik (speziell Futility-Analyse).

Biobanking und Sample Management:

Institut für Klinische Molekularbiologie, CAU und UKSH, Campus Kiel.

Finanzierung:

Die Studie wird aus Eigenmitteln der Klinik für Innere Medizin I (UKSH, Campus Kiel), aus einer Anschubfinanzierung des Landes Schleswig-Holstein (Projekt DOI30) und vom Exzellenzcluster PMI finanziert. Das Studienprotokoll wurde vor Beginn der Rekrutierung im Deutschen Register Klinischer Studien (DRKS) registriert und wird nach zustimmender Bewertung entsprechend aktualisiert. Parallel dazu ist die Studie bei ClinicalTrials.gov registriert.

3 Wissenschaftlicher Hintergrund

Die Verfügbarkeit von Tryptophan und seiner Metabolite, insbesondere Nicotinamid (einer Form von Vitamin B3) als Baustein von NAD und NADP, sind wichtige Faktoren bei chronischen systemischen Entzündungen. Hier kann ein evolutionär alter Verteidigungsmechanismus fehlgeleitet werden, indem der Körper die Energienutzung in Zellen analog zur Eisenreduktion bei Entzündung drosselt (Boergeling & Ludwig 2017, FEBS J. 284:218). Während dies in einfachen Organismen Sinn ergibt, führen solche Mechanismen in komplexen Organismen zu Dysfunktionen (z.B. zu entzündungsbedingter Anämie). Reduzierte Spiegel an Nicotinamid und damit NAD(P) behindern die Immunabwehr gegen Coronaviren (Heer *et al.* 2020, J. Biol. Chem. 295:17986) und den Zellstoffwechsel, insbesondere auch der Makrophagen und Epithelzellen. Gerade letztere unterliegen einer kritisch hohen Erneuerungsrate im Darm und in der Lunge. Zudem kann Kynurenin als Hauptabbauprodukt des Tryptophans auch stark immunaktivierend wirken. Eine weitere Verbindung zwischen SARS-CoV-2 jenseits von Entzündung und Immunsystem könnte auch der SARS-CoV-2-Eintrittsrezeptor ACE2 sein, da die Anwesenheit von ACE2 auf der Zelloberfläche die Aufnahme von Tryptophan über den Rezeptor B0AT1 ermöglicht. Bei schweren Verläufen von Influenza werden ebenso wie bei chronischer Entzündung ein erhöhter Tryptophan-Abbau und damit erhöhte Kynurenin-Spiegel beobachtet, und Hemmung des Tryptophan-Abbaus hat im Tiermodell positive Effekte (Pizzini *et al.* 2019, Influenza Other Respir. Viruses 13:603; Boergeling & Ludwig 2017, FEBS J. 284:218). Im Gegenzug gibt es Hinweise bei unterschiedlichsten Virustypen, dass Nicotinamid die Virusreplikation reduzieren und die Abwehrmechanismen des Körpers unterstützen kann, z.B. bei Vaccinia (Child *et al.* 1988, Virus Res. 9:119), HIV (Murray 2003, Clin. Infect. Dis. 36:453), Enteroviren (Moell *et al.* 2009, J. Med. Virol. 81:1082) oder Hepatitis B (Li *et al.* 2016, Arch. Virol. 161:621). Auch für die Bekämpfung der Infektion mit SARS-CoV-2 wird eine hinreichende Versorgung mit B-Vitaminen und explizit Nicotinamid zur Stärkung des Immunsystems empfohlen (Zhang & Liu 2020, J. Med. Virol. 92:479; Gharote 2020, Ind. J. Med. Sci. 72:25; Shakoor et al. 2021, Maturitas 144:108). Hier wird besonders die Verbesserung der Abwehr bakterieller Sekundärinfektionen in Krankheitsmodellen hervorgehoben (Zhang & Liu 2020, J. Med. Virol. 92:479). Die in einer Studie beobachtete verminderte Sauerstoffsättigung bei einer Dosis von 400 mg/kg (dies entspräche einer wohl toxischen Dosis von 28 g bei einer 70-kg-Person) hat keine Aussagekraft für eine Nahrungsintervention mit 1.000 mg Gesamtdosis und angesichts des *acceptable daily intake* von 900 mg/Tag (EFSA Panel on Dietetic Products, Nutrition and Allergies 2014, EFSA J. 12:3759). In der COVit-Studie werden 1.000 mg verabreicht, was weit unter potentiell schädlichen Dosen von etlichen Gramm pro Tag und sehr nahe am ADI liegt [OECD-SIDS: 3-pyridinecarboxamide (nicotinamide), SIDS Initial Assessment Report for SIAM 15, Boston, Massachusetts, 22-25 October 2002]. Mittlerweile legen etliche Publikationen den Einsatz von Nicotinamid bei COVID-19 nahe, aber es gibt keine Studiendaten dazu (Shi *et al.* 2020, Cell Death Differ. 27:1451; Gharote 2020, Ind. J. Med. Sci. 72:25; Mehmel *et al.* 2020, Nutrients 12:1616; Shakoor *et al.* 2021, Maturitas 144:108; Heer *et al.* 2020, J. Biol. Chem. 295:17986).

In der Pilotphase der COVit-Studie (COVit-1) erhielten zunächst je n=8 Patienten 1.000 mg herkömmliches Nicotinamid oder 245 mg Kieselerde als Placebo. Bei einer Qualitätskontrolle der Datenerhebung wurde überraschend beobachtet, dass 4 von 8 Patienten (50 %) aus der Nicotinamidgruppe bereits nach zweiwöchiger Gabe vollständig symptomfrei waren, während dies in der Kontrollgruppe nur bei einem der 8 Patienten (12,5 %) der Fall war. Die Auswertung der COVit-Vorstudie mit n=28 pro Gruppe zeigte eine signifikante Verkürzung der Zeit bis zur vollständigen Symptomfreiheit, die von Effekten bei Patientinnen getragen wurde. Aus der

Literatur waren je nach Patientenpopulation ebenfalls sehr lange Rekonvaleszenzen zu erwarten, z.B.

- 43 % Symptomfreiheit 14–21 Tage nach positivem SARS-CoV-2-Test (Tenforde et al. 2020, MMWR 69:99),
- <30 % ohne Atembeschwerden nach einem Monat (Marshall 2020, Nature 585:339),
- 32 % Symptomfreiheit nach 30 Tagen und immer noch nur 34 % nach 60 Tagen nach SARS-CoV-2-Diagnose (Carvalho-Schneider et al. 2021, Clin. Microbiol. Infect. 27:258), oder
- nur 12,6 % Symptomfreiheit nach im Mittel 60 Tagen (Carfi et al. 2020, JAMA 324:604).

Zudem hat sich COVID-19 seit Beginn der ursprünglich geplanten COVit-Studie als Systemerkrankung mit einer unerwartet hohen gastrointestinalen Symptomhäufigkeit erwiesen (Mitsuyama et al. 2020, J. Clin. Med. 9:3630). Vor kurzem wurde publiziert, dass auch das Darmmikrobiom von COVID-19 deutlich und z.T. negativ beeinflusst wird (Yeoh *et al.* 2021, Gut 70:698). Daher werden in der Hauptphase der Studie (COVit-2) nun zwei verschiedene Tabletten mit je 500 mg Nicotinamid als Prüfpräparate eingesetzt: die herkömmlichen Nicotinamidtabletten mit unmittelbarer Freisetzung aus der Pilotphase und die UKSH-Eigenentwicklung CICR-NAM (*controlled-ileocolonic-release nicotinamide*), deren Tabletten das Nicotinamid verzögert und kontinuierlich ab dem unteren Dünndarm freisetzen. Dadurch soll die gastrointestinale positive Wirkung von Nicotinamid, speziell auch auf das Mikrobiom und dessen Interaktion mit dem Darm, erhöht werden, da das herkömmliche Nicotinamid sehr schnell in den Kreislauf aufgenommen wird (Fangmann *et al.* 2018, Diabetes Care 41:398). Vorexperimente mit einem CICR-NAM-Prototyp haben gezeigt, dass die systemische Exposition durch die neue Dosierung geringer sein wird als in der Pilotphase der Studie (Fangmann *et al.* 2018, Diabetes Care 41:398), in der die Exposition ohnehin schon nahe am ADI lag (s.o.). Damit wird das ohnehin minimale Risiko für Nebenwirkungen noch weiter reduziert.

Der wissenschaftliche Hintergrund hat sich im Laufe der Studie weiterentwickelt und im Rahmen der geplanten Futility-Analyse und Fallzahlüberprüfung zu einer Anpassung von Endpunkten und Analysen geführt. Dies wird ausführlich in Abschnitt 11 erläutert.

4 Projektziele

4.1 Primärziel und Hypothese

Das Primärziel der Studie ist die Untersuchung der Hypothese, dass COVID-19-Patienten unter realen Bedingungen einzelne COVID-19-Symptome schneller verlieren, wenn sie 1.000 mg Nicotinamid (500 mg herkömmliches Nicotinamid und 500 mg CICR-NAM) supplementieren.

4.2 Sekundärziele

Neben dem genannten Primärziel sind die Sekundärziele der Studie die genauere Untersuchung folgender Parameter: Stärke einzelner Symptome, Auftreten und Stärke von Symptomen bei der 6-Monats-Nachverfolgung (PCS), Spiegel von Antikörpern gegen N-Protein und S-Protein von SARS-CoV-2 nach mindestens 6 Monaten, vollständige Symptomfreiheit nach 2, 4 und 6 Wochen sowie die Zeit von der Diagnose bis zur Freiheit von einzelnen oder allen Symptomen.

4.3 Explorative Ziele und Fragestellungen

Explorativ sollen die klinische WHO-Skala für COVID-19 und eine Entwicklung von schwerem COVID-19 (Untersuchung in einer Notaufnahme, Hospitalisierung mit mindestens 24 Stunden Sauerstoffpflicht, Intensivpflicht, Beatmungspflichtigkeit oder Tod) untersucht werden. Änderungen bei Fatigue und Lebensqualität werden mittels validierter Fragebögen (FACIT-F und SF-36) analysiert. Weitere explorative Untersuchungen an bis zu 20 ausgewählten Patienten sollen Aufschluss über Blutbild, Standardblutprofil, verschiedene Entzündungsmarker und das Metabolom im Blut, insbesondere den Tryptophanstoffwechsel, geben. Bei diesen Patienten wird auch der Virenstamm durch Sequenzierung aus Nasen-Rachen-Abstrichen bestimmt. Bei ausgewählten Patienten kann sich eine detaillierte Pharmakokinetik anschließen. Im Stuhl werden die Veränderungen von Mikrobiom (bei 100–300 Patienten) sowie Metagenom und Metabolom (bei einer Subgruppe) analysiert.

5 Endpunkte

Der Krankheitsverlauf der Nicotinamid-supplementierten Gruppe und der Placebogruppe wird anhand folgender Endpunkte verglichen:

Primärer Endpunkt:

Auftreten einzelner COVID-19-Symptome zu Woche 0, Woche 2 (primärer Analysezeitpunkt), Woche 4 und Woche 6 sowie nach 6 Monaten.

Sekundäre Endpunkte:

1. Stärke einzelner COVID-19-Symptome zu Woche 0, Woche 2 (primärer Analysezeitpunkt), Woche 4 und Woche 6 sowie nach 6 Monaten.
2. Im Rahmen der 6-Monats-Nachverfolgung: Auftreten und Stärke von Symptomen, die charakteristisch für das PCS sind.

   Hierzu wird vom Patienten selbst ein dafür validierter Riechtest (Smell Identification Test™; Sensonics / MediSense) durchgeführt. Zudem werden neben den Fragebögen FACIT-F und SF-36 (wie während des eigentlichen Studienzeitraums von Woche 0 bis Woche 6) noch weitere Fragebögen (siehe Anhang 19.6.) zum Riech- und Schmeckvermögen (inkl. Questionnaire of Olfactory Disorders, QOD), zur Atmung (Multidimensionales Dyspnoe-Profil, MDP), zur seelischen Verfassung (Patient Health Questionnaire Depression, PHQ-8; Generalized Anxiety Disorder 7, GAD-7; Perceived Stress Scale, PSS; Brief Resilience Scale, BRS), zur Schlafqualität (Pittsburgh Sleep Quality Index, PSQI) und zur Fatigue (Multidimensional Fatigue Inventory, MFI) ausgefüllt. Zusätzlich wird ein für Telefonbefragungen validierter Kognitionstest (T3MS) durchgeführt.
3. Spiegel von Antikörpern gegen N-Protein und S-Protein von SARS-CoV-2 nach mindestens 6 Monaten (Anti-S-Protein stratifiziert nach Vorhandensein und Art einer Boosterimpfung).
4. Vollständige Symptomfreiheit nach 2 Wochen.
5. Vollständige Symptomfreiheit nach 4 Wochen.
6. Vollständige Symptomfreiheit nach 6 Wochen.

7. Zeit von der Diagnose bis zur Freiheit von einzelnen Symptomen (in Tagen, bis zu 6 Wochen).

8. Zeit von der Diagnose bis zur vollständigen Symptomfreiheit (in Tagen, bis zu 6 Wochen).

Folgende Symptome werden in Telefoninterviews abgefragt und in ihrem Verlauf einzeln explorativ untersucht: Fatigue / Abgeschlagenheit / Erschöpfung / Kraftlosigkeit, Leistungsabfall / eingeschränkte körperliche Leistungsfähigkeit, Fieber (bis 39 °C bzw. darüber), Schüttelfrost, Atemnot / Kurzatmigkeit, pfeifende oder keuchende Atmung, Lungenentzündung, Husten (mit oder ohne Auswurf / Sputumproduktion), Schnupfen / laufende Nase / Nasenschleimproduktion / Rhinorrhoea, Hals- / Rachenschmerzen oder Halskratzen, Heiserkeit, Muskelschmerzen (Myalgie), Gelenkschmerzen (Arthralgie), Gliederschmerzen, Brustschmerzen, Kopfschmerzen, Bauchschmerzen, Durchfall / Diarrhoe, Übelkeit, Erbrechen, Appetitlosigkeit / geringere Nahrungsaufnahme, weitere Magen-Darm-Symptome (vom Patienten individuell zu nennen), eingeschränkter Geruchssinn, eingeschränkter Geschmackssinn, Bewusstseinsstörungen / Verwirrtheit, Schwindel, Bindehautentzündung (Konjunktivitis), Hautausschlag, Haarausfall und andere Symptome (vom Patienten individuell zu nennen).

In einer Subgruppe von Patienten werden die täglichen Änderungen des Schweregrads einzelner Symptome während der ersten 4 Wochen explorativ untersucht; Abstufungen der Symptome: 0 = gar nicht, 1 = leicht, 2 = mäßig, 3 = schwer, 4 = unerträglich.

Zudem wird eine Beschwerdeskala für Infektionen der unteren Atemwege mit den Aspekten Husten, Schleimbildung, Kurzatmigkeit, Schlaf, Fähigkeit zur normalen Aktivität und allgemeines Krankheitsgefühl mit folgenden Abstufungen abgefragt: 0 = normal, 1 = sehr geringes Problem, 2 = geringes Problem, 3 = mäßig schlecht, 4 = schlecht, 5 = sehr schlecht, 6 = maximal schlecht.

Explorative Endpunkte:

1. World Health Organization (WHO) COVID-19 Ordinal Scale for Clinical Improvement (siehe https://www.who.int/publications/i/item/covid-19-therapeutic-trial-synopsis) zu Woche 0 (Baseline), Woche 2, Woche 4 und Woche 6 sowie nach 6 Monaten.

2. Auftreten von schwerem COVID-19, definiert als Erreichung eines der folgenden Charakteristika:

    a. Untersuchung in einer Notaufnahme;

    b. Hospitalisierung mit einer durchgehenden Sauerstoffpflicht von mindestens 24 Stunden;

    c. Intensivpflicht;

    d. Beatmungspflicht;

    e. Tod durch COVID-19.

3. Vergleich zwischen den Befunden aus der Pilotphase der Studie und dem zweiten Studienteil.

4. Änderungen bei der Fatigue (Fragebogen FACIT-F).

5. Änderungen bei der Lebensqualität (Fragebogen SF-36).

6. Änderung der Spiegel von Tryptophan im Blut (bei ausgewählten Patienten).
7. Änderung der Spiegel von Tryptophan-Metaboliten im Blut (bei ausgewählten Patienten).
8. Änderung von Entzündungsmarkern (C-reaktives Protein, Interleukin-6, Ferritin, Neopterin, D-Dimere) im Blut (bei ausgewählten Patienten).
9. Änderungen im Blutbild und Standardblutprofil (bei ausgewählten Patienten).
10. Änderungen in der Zusammensetzung des Blutmetaboloms (bei ausgewählten Patienten).
11. Stamm des SARS-CoV-2-Virus (bei ausgewählten Patienten).
12. Änderungen in der Zusammensetzung des Stuhlmikrobioms (bei ausgewählten Patienten).
13. Änderungen in der Zusammensetzung des Stuhlmetagenoms (bei ausgewählten Patienten).
14. Änderungen in der Zusammensetzung des Stuhlmetaboloms (bei ausgewählten Patienten).
15. Pharmakokinetik (bei ausgewählten Patienten).

6 Studiendesign

Es handelt sich hierbei um eine monozentrische, randomisierte Studie, bei der symptomatische COVID-19-Patienten eine Nahrungsergänzung mit 1.000 mg Nicotinamid pro Tag in Tablettenform (1 x 500 mg NicoPel, IFC Deutschland/Derma Enzinger, und 1 x 500 mg CICR-NAM, NextPharma/UKSH) oder passende Placebotabletten (Fagron bzw. NextPharma/UKSH) erhalten. In der Pilotphase erhielt die Nicotinamid-Gruppe 2 x 500 mg NicoPel und die Kontrollgruppe 245 mg Kieselerde in einer Kapsel (1 x 245 mg Kieselerde-Kapsel, Twardy/Saluspharma). Die nicht perfekte Paarung von Verum und Placebo-ähnlicher Kontrolle war durch die Marktverfügbarkeit der Prüfpräparate und die enorme Dringlichkeit der Studie zu rechtfertigen. Es wird davon ausgegangen, dass die Vergleichsgruppe „Heilmittel Kieselerde“ eine mit der Placebobehandlung vergleichbare Effektstärke hatte, da Kieselerde überwiegend aus inertem Siliziumdioxid besteht. Allerdings sind auch für Kieselerde positive Aussagen über den Ernährungsbenefit als bewerbbare Beurteilungen etabliert, daher wurde das Placebo für den zweiten Studienteil optimiert. Eine doppelte Verblindung war und ist soweit wie möglich gewährleistet. Für die Patienten geht aus der Verpackung und Einnahmeanweisung des zugesandten Nahrungsergänzungsmittels nicht hervor, welches Präparat sie bekommen haben. Das datenerhebende Personal an den Telefonen wird per SOP angewiesen, die Art des Prüfpräparats nicht zu thematisieren und ist hinsichtlich der Gruppenzugehörigkeit der angerufenen Studienteilnehmer verblindet. Alle Patienten erhalten den üblichen klinischen Behandlungsstandard, die Nahrungsergänzung ist rein additiv zum Standard of Care.

7 Studienpopulation

Die zu untersuchende Studienpopulation von insgesamt bis zu ca. 840 Studienteilnehmern betrifft Patienten mit bestätigter SARS-CoV-2-Infektion und Symptomen, z.B. im Bereich der Atemwege und/oder des Gastrointestinaltrakts.

Die Einschlusskriterien sind:

- Durch Laborbefund gesicherte SARS-CoV-2-Infektion; der positive Test darf nicht mehr als 7 Tage zurückliegen.
- Einschlägige Infektsymptome, z.B. im Bereich der Atemwege oder des Gastrointestinaltrakts.
- Der Patient/die Patientin konnte vor Durchführung jeglicher Studien-Prozedur schriftlich über eine Webseite einwilligen und kann die studienabhängigen Voraussetzungen und Anforderungen einhalten.
- Der Patient/die Patientin ist volljährig (vollendetes 18. Lebensjahr).

Die Ausschlusskriterien sind:

- Aktuelle Teilnahme an einer anderen Studie.
- Schwangerschaft oder Stillen.
- Eine Impfung gegen SARS-CoV-2.

Die Rekrutierung findet in Kiel und in weiteren Arztpraxen, Schwerpunktkrankenhäusern und Teststellen/-laboren für SARS-CoV-2 in Deutschland statt.

8 Studienablauf

8.1 Aufklärung und Einwilligung

Die Therapieentscheidungen bei der Behandlung von COVID-19 liegen außerhalb des Studienprotokolls, d. h. die Indikationsstellung ist unabhängig und wird ohne Einfluss durch das Forschungsprogramm gestellt. Erst nach Indikationsstellung wird über eine mögliche Rekrutierung entschieden.

Alle Patienten in den jeweiligen Zentren (ambulant und stationär), welche alle Ein- und Ausschlusskriterien erfüllen, werden um eine Studienteilnahme gebeten. Eine studienspezifische Prozedur außerhalb des klinischen Standards im Screening erfolgt nicht. Die Patienten erhalten eine schriftliche Patienteninformation mit Aufklärung. Für Rückfragen und ein ärztliches Aufklärungsgespräch steht bei Bedarf eine Hotline zur Verfügung. Der Patient kann im Anschluss nach ausreichender Bedenkzeit online einwilligen und seine Einwilligung durch Ausdruck der entsprechenden Seite für sich dokumentieren (Probandeneinwilligung). Dieses Vorgehen ermöglicht auch die Teilnahme von Patienten in häuslicher Quarantäne. Erst nach der Online-Einwilligung erfolgt der erste Telefonkontakt zur Datenerhebung für Woche 0 (Baseline), bei dem zu Beginn die Fähigkeit zur Einwilligung des Patienten durch das Studienpersonal am Telefon noch einmal nach einer SOP überprüft wird. Auch hier kann der Patient weitere Rückfragen stellen und die ärztliche Hotline kann hinzugezogen werden.

Für den Riechtest, die zusätzlichen Fragebögen, den Kognitionstest und den Test auf SARS-CoV-2-Antikörper bei der 6-Monats-Nachverfolgung sowie für die pharmakokinetischen Messungen werden ergänzende schriftliche Einwilligungen der Patienten eingeholt.

8.2 Maßnahmen (Intervention/Kontrolle) und Zielgrößen

Nach der Einwilligung und Erfüllung aller Voraussetzungen werden die Patienten nach dem Zufallsprinzip dem Nicotinamid-Arm oder dem Placebo-Arm zugeordnet. Darüber wird der Patient aufgeklärt.

Nach der Randomisierung im Verhältnis 1:1 sind Kontroll-/Interviewanrufe zu Woche 0 (Baseline) sowie Woche 2, Woche 4 und Woche 6 geplant. Nach ca. 6 Monaten ist ein weiterer

Kontrollanruf zur Abklärung von Langzeitsymptomen vorgesehen. Zusammen mit den Tabletten erhalten die Patienten ausgedruckte Fragebögen zur Charakterisierung von Fatigue (FACIT-F) und Lebensqualität (SF-36), die sie selbst ausfüllen und per frankiertem Rückumschlag zurückschicken sollen. In diesem Zusammenhang können perspektivisch auch Wearables analog zum Votum A 101/20 zum Einsatz kommen, dies würde dann noch genauer spezifiziert und vorgelegt. Bis zu 400 Patienten erhalten Probenbehälter für Stuhlproben, die per Rückumschlag ans IKMB gesandt werden. Das Ziel ist es, 100–300 Probenserien für Stuhlmikrobiomanalysen zu erhalten, von denen ein repräsentativer Teil zusätzlich auf Änderungen im Stuhlmetagenom und Stuhlmetabolom untersucht wird. Bei voraussichtlich bis zu 20 Patienten aus Kiel und Umgebung (je bis zu 10 Patienten mit Verum und Placebo) werden zusätzlich Blutproben zu den Interviewzeitpunkten sowie zu Woche 1 genommen, um die explorativen Blutanalysen durchzuführen (siehe Endpunkte). Bei diesen Patienten wird zu Beginn der Untersuchungen auch der Virenstamm durch Sequenzierung aus Nasen-Rachen-Abstrichen bestimmt. Bei bis zu 5 ausgewählten Patienten aus dieser Gruppe wird außerdem zu einem späteren Zeitpunkt die Pharmakokinetik von Nicotinamid, Nicotinsäure und Nicotinursäure sowie von Metaboliten des Nicotinamid- und Tryptophanstoffwechsels in den ersten 48 Stunden nach einer einmaligen Gabe der Studienpräparate untersucht.

Darüber hinaus soll durch eine Nachbefragung, zusätzliche Fragebögen, einen Riechtest, einen Kognitionstest und einen Test auf SARS-CoV-2-Antikörper nach 6 Monaten untersucht werden, ob die Supplementation mit Nicotinamid einen Einfluss auf das PCS sowie die Immunreaktion und ggf. Impfung gegen SARS-CoV-2 hat. Die hier gewonnenen Informationen entsprechen (bis auf den zusätzlichen Antikörpertest) den im Rahmen der COVIDOM-Studie erhobenen Daten und erlauben eine spätere, separat zu begutachtende Vernetzung der beiden Studien.

Die Telefoninterviews werden vom Kompetenznetz Darmerkrankungen e.V. geführt. Bei medizinischen Fragen ruft ein Arzt aus der Klinik für Innere Medizin I zurück. Bei den Anrufen werden folgende Parameter abgefragt bzw. eingetragen:

Woche 0:

- Persönliche und demographische Daten
- COVID-19-relevante und andere Symptome
- Einschlusskriterien
- Komorbiditäten
- Raucherstatus
- Chronische Medikation und Supplementierung
- Hospitalisierung
- WHO-Skala zu COVID-19
- Art der Supplementierung
- Epidemiologische Fragen zu den Lebensumständen

Woche 2, 4 und 6:

- Einnahme der Supplementierung und ggf. anderer Nahrungsergänzungsmittel
- COVID-19-relevante und andere Symptome
- Tod und Todesursache
- Raucherstatus

- Untersuchung in einer Notaufnahme
- Hospitalisierung
- WHO-Skala zu COVID-19
- Beatmungspflicht
- Intensivstationäre Behandlung

Der Ablauf der Studie wird in folgendem Flow Chart zusammengefasst:

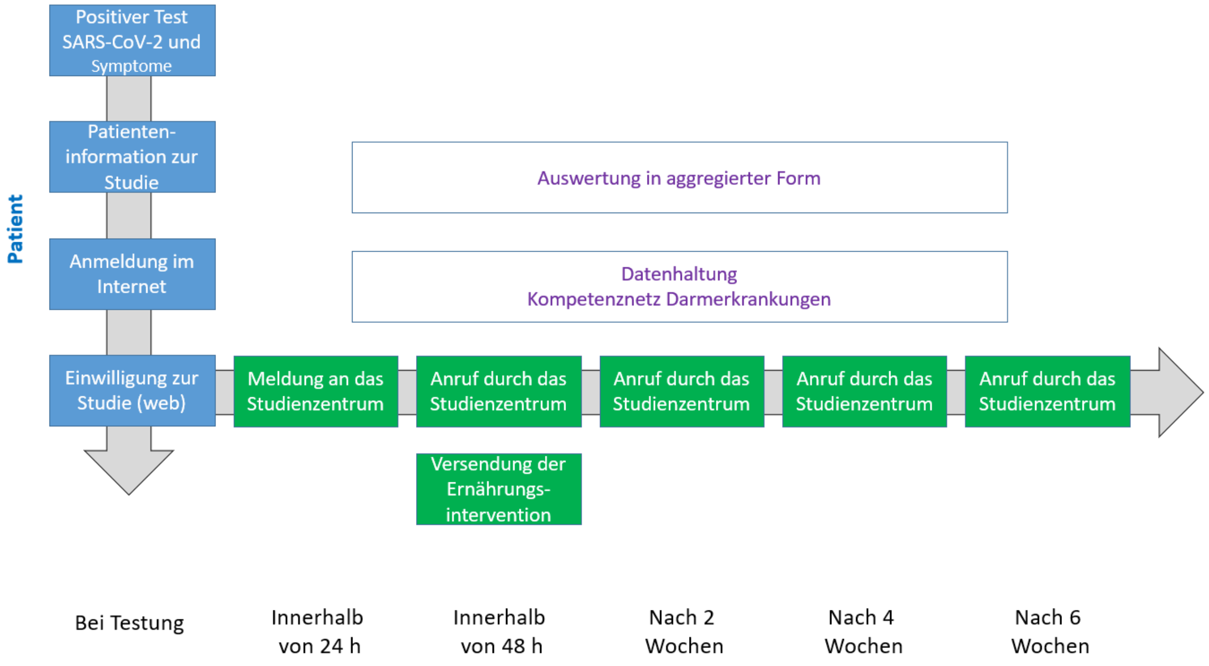

8.3 Studiendauer

Die Studiendauer für den einzelnen Patienten beträgt regulär 4 Wochen bis zum Studienende, bei persistierenden Symptomen 6 Wochen. Ein Abschlussgespräch erfolgt nach ca. 6 Monaten. Dieses zielt besonders auf die Lebensqualität und eventuelle Spätfolgen ab und umfasst auch den Fragebogen EQ-5D-5L (im eCRF) sowie weitere Papierfragebögen, einen Riechtest und einen Kognitionstest (siehe Abschnitt 5) analog zur COVIDOM-Studie, in welche die Patienten der COVit-Studie auch eingeschlossen werden können. Zudem können die Patienten einen Test auf SARS-CoV-2-Antikörper durchführen lassen. Die Gesamtdauer der Studie richtet sich nach dem Rekrutierungserfolg und dem weiteren Verlauf der COVID-19-Epidemie. Die Rekrutierung hat am 01.02.2021 begonnen. Wir rechnen mit einem Einschluss des letzten Patienten Ende 2021 / Anfang 2022.

9 Nutzen-Risiko-Abwägung

Wenn die Optimierung des Nicotinamid-Status zumindest einem Teil der COVID-19-Erkrankten dabei hilft, die Krankheit schneller und/oder leichter zu überwinden, ist bei der sich immer wieder dramatischen zuspitzenden Lage jeder nicht hospitalisierte oder beatmungspflichtige und jeder schneller vollständig gesundete Patient persönlich (Eigennutzen) und auch bezogen auf die Gesamtheit aller COVID-19-Patienten (Gruppennutzen) und anderen Patienten im Krankenhaus sowie bezogen auf die

sozioökonomischen Folgen (Krankheitstage, Spätfolgen) ein Gewinn. Der weitere Fremdnutzen besteht in einem Erkenntnisgewinn für die klinische Forschung (Verbesserung der Krankenversorgung durch ein einfaches, sofort verfügbares und sicheres Nahrungsergänzungsmittel). Risiken sind nicht erkennbar.

Die Supplementation von Patienten wird bei Erreichen eines kritischen Zustands und Aufnahme des Patienten in einen Überwachungsbereich abgebrochen.

10 Fallzahlberechnung und Statistik

Die Datenlage zu Beginn der COVit-2-Studie ließ annehmen, dass höchstens 40 % der bereits symptomatischen COVID-19-Patienten zwei Wochen nach Beginn der Intervention vollständig symptomfrei sein würden (damals festgelegter primärer Endpunkt). Dies wäre im Kollektiv der mit Placebo behandelten Patienten voraussichtlich der Fall gewesen. Die Hypothese war, dass diese Ereignisrate von 40 % durch den Einsatz von Nicotinamid auf 50 % gesteigert werden kann.

Die statistische Analyse wird mit SAS-Software Version 9.3 oder neuer (SAS Institute, Inc., Cary, North Carolina) bzw. mit R durchgeführt. Die statistische Methodologie wird im Detail in einem statistischen Analyseplan (SAP) in finaler Form vor dem Database-Lock niedergelegt. Jegliche Änderung des Analyseplans im Protokoll wird im SAP dokumentiert und im finalen Studienbericht dargestellt. Kontinuerliche Variablen werden mit einer deskriptiven Statistik zusammengefasst (Zahl der Beobachtungen (n), Mittelwert/Median, Standardabweichung, Minimum und Maximum). Kategorische Variablen werden in Frequency Tables berichtet, welche die Zahl und den Prozentsatz von Patienten in den jeweiligen Kategorien zusammenfassen.

Die „Intention-to-treat" (ITT)-Population ist definiert als „alle eingeschlossenen Patienten". Die „All-Randomized" (AR)-Population umfasst alle Patienten, die für mindestens zwei Wochen beobachtet wurden (Einnahme des Nahrungsergänzungsmittels und Telefoninterviews). Die AR-Population wird für die Beurteilung der primären und sekundären Endpunkte genutzt. Die Biomarker (BM)-Population umfasst alle Patienten, bei denen Biomaterialien asserviert werden konnten.

Für die Fallzahlplanung wurden als Standardgrößen angenommen: α-Fehler = 0,05; Power = 0,8; es ergibt sich ein K = 7,85 (Faktor, der sich aus α-Fehler und Power berechnet). Die folgende vereinfachte Formel wurde benutzt:

$$n = \frac{K\,[(R+1) - p_2\,(R^2+1)]}{p_2\,(1-R)^2}$$

n = benötigte Fallzahl

p1 = Ereignisrate in der Behandlungsgruppe (50 %)

p2 = Ereignisrate in der Kontrollgruppe (40 %)

R = relatives Risiko (p1/p2) = hier: 50 % / 40 % = 1,25

Im Ergebnis wurden 385 Patienten pro Gruppe benötigt. Als Sicherheitsreserve für Dropouts wurden aufgrund der bisherigen Erfahrungen knapp 10 % veranschlagt, daher sollten 420 Patienten pro Gruppe eingeschlossen werden. Die Fallzahlberechnung sollte ggf. anhand der verblindet ermittelten Ereignisrate in der Studie angepasst werden.

Der primäre Endpunkt wird unter Verwendung eines generalisierten linearen Modells mit Log-Link und binomialer Fehlerverteilung analysiert. Die Ergebnisse werden als relatives Risiko mit 95%-Konfidenzintervall dargestellt. Binäre sekundäre Endpunkte werden mit logistischen Regressionsmodellen analysiert. Ordinale sekundäre Endpunkte (z.B. Symptomschwere) werden mit Proportional-Odds-Modellen mit der Behandlungsgruppe als unabhängiger Variablen analysiert. Zusätzlich werden Stratifizierungen nach wichtigen prognostischen Faktoren wie Symptomschwere oder bestimmten Symptomen zu Woche 0 (Baseline) vorgenommen. Die Details sind im SAP niedergelegt.

Aufgrund der Ergebnisse der Futility-Analyse hat das Data Management Board (DMB) der Studienleitung empfohlen, den bislang geplanten Rekrutierungsumfang von ca. 840 Patienten beizubehalten und als primäre klinische Analysepopulation die Subgruppe von Patienten mit mindestens einem Risikofaktor für einen schweren COVID-19-Verlauf zu wählen (siehe Abschnitt 11). Um sicherzustellen, dass auch in der nach der Futility-Analyse rekrutierten Teilpopulation ein adäquater Anteil solcher Patienten enthalten ist, werden die Frequenzen der Risikofaktoren verblindet überwacht und zur Absicherung gegen eine eventuelle Häufung von Studienabbrüchen bis zu 10 % Patienten mehr rekrutiert.

11 Futility-Analyse und Anpassung der Stichprobengröße

Nach 400 abgeschlossenen Patienten (mit Datenerhebung bis inkl. Woche 6) wurde die Frequenz des primären Endpunkts in den verblindeten Daten beurteilt. Bei der Planung der COVit-2-Studie wurde erwartet, dass dann ca. 45 % der Patienten nach 2 Wochen Nahrungsergänzung symptomfrei sein sollten. Gegebenenfalls sollte eine Zwischenauswertung und eine Anpassung der Stichprobengröße mit Erweiterung oder Verringerung der Patientenzahl erfolgen.

Für diese geplante Futility-Analyse durch das DMB der COVit-Studie nach Erhebung aller Daten bis Woche 6 für 400 Patienten stand ein bereinigter Datensatz mit n = 402 Patienten (je 201 mit Nicotinamid bzw. Placebo supplementiert) zur Verfügung. In diesem Datensatz wurden von den externen und unabhängigen Statistikern der Firma Novustat AG statistisch signifikante positive Effekte von Nicotinamid auf bestimmte Subgruppen gefunden, die am häufigsten und stärksten nach 2 Wochen auftraten.

Für die zwischenzeitlich weiter rekrutierten Patienten wurden beim Datenmanagement des Kompetenznetzes Darmerkrankungen e.V. verblindete und unbereinigte Frequenzen von Leitsymptomen und Zugehörigkeiten zu größeren Risikogruppen angefordert. Diese wiesen darauf hin, dass sich das Patientengut ab Patient Nr. 403 nicht wesentlich von dem davor unterscheidet. Aufgrund des Ausschlusskriteriums einer Impfung gegen SARS-CoV-2 findet allerdings eine zunehmende soziale Selektion statt.

Der bisherige primäre Endpunkt „vollständige Symptomfreiheit nach 2 Wochen“ und die entsprechenden sekundären Endpunkte basierten auf dem Verständnis des Krankheitsverlaufs zu Beginn der COVit-2-Studie und der aus heutiger Sicht ungewöhnlichen Verteilung der Risikofaktoren in der Patientenkohorte der Vorstudie COVit-1. Mittlerweile ist klar, dass sich die Populationen von COVit-1 und COVit-2 unterscheiden und die in COVit-1 vergleichsweise oft erreichte vollständige Symptomfreiheit bei den Patienten in COVit-2 viel seltener auftritt. Dies gilt sowohl für die Gesamtpopulation als auch für Subgruppen mit COVID-19-Leitsymptomen wie z.B. Husten zu Woche 0. Zudem ist die Häufigkeit dieser Leitsymptome bei Patienten in COVit-2 geringer als in COVit-1, was die Signalamplitude verringert. Die in COVit-2 andererseits beobachtete längere Erkrankungsdauer deckt sich mit aktuellen Literaturdaten, auch zum PCS. Das PCS sollte auf Anraten des DMBs stärker als

bislang bei der Auswertung der COVit-2-Studie berücksichtigt werden, zumal hier im Studienverlauf noch eine aussagekräftige Batterie von Tests und Fragebögen hinzugekommen ist.

Ebenfalls neu zu berücksichtigen seien die Designs und ersten Ergebnisse klinischer Studien mit oral einnehmbaren Medikamentenkandidaten wie Molnupiravir und PAXLOVID™. Diese Substanzen sollen, ähnlich wie Nicotinamid, gleich nach der Diagnose einer SARS-CoV-2-Infektion eingesetzt werden, um Krankheitsverläufe abzumildern. Solche Kandidaten gab es zum Zeitpunkt der Planung von COVit-2 leider noch nicht. Die Studien zu Molnupiravir und PAXLOVID™ haben jedoch nur Patienten mit mindestens einem Risikofaktor für einen schweren Krankheitsverlauf einbezogen, weil diese besonders von einer frühzeitigen Intervention zu profitieren scheinen. Die Gesamtpopulation von COVit-2 ist hingegen zu leicht und zu lange erkrankt, um mit der bisherigen Strategie sinnvolle Auswertungen zu betreiben.

Neuere Erkenntnisse zum NAD-Metabolismus reifer B-Zellen (Bernardes et al. 2020, Immunity 53:1296) deuten zudem darauf hin, dass neben der symptombezogenen Analyse im akuten und postakuten Krankheitsverlauf auch die immunologische Antwort auf SARS-CoV-2 untersucht werden sollte. Es ist gut vorstellbar, dass eine Supplementation mit Nicotinamid durch die erhöhte Verfügbarkeit von NAD die Antikörperbildung während der Infektion unterstützen könnte. Entsprechende Messungen der Antikörperspiegel nach ca. 6 Monaten sind bereits Teil der 6-Monats-Nachverfolgung und sollten aufgewertet werden.

Das DMB hat bei seinen Beratungen daher folgende Schlussfolgerungen gezogen:

1. Die Weiterführung der Studie war und ist geboten, weil die Patienten keine erkennbaren Nachteile durch die Supplementation haben und bestimmte Gruppen von der Supplementation mit Nicotinamid signifikant profitieren.

2. Der bislang geplante Rekrutierungsumfang von ca. 840 Patienten sollte beibehalten werden. Da in einzelnen Subgruppen bezüglich COVID-19-Symptomen bzw. Risikofaktoren wesentlich höhere Effektstärken als bislang angenommen beobachtet werden konnten, wären damit voraussichtlich hochsignifikante Signale in der Gesamtpopulation von $n \geq 800$ (mit entsprechender Reserve von ca. 5 % für Studienabbrüche, daher 840) zu erwarten. Auch bei einer Aufteilung in eine hypothesengenerierende Population der ersten $n = 402$ und eine ca. gleich große Validierungspopulation wären gemäß den Befunden in den ersten $n = 402$ einige signifikante Ergebnisse zu erwarten.

3. Da es sich nicht um eine Studie nach AMG handelt, sich die Voraussetzungen für die Studie angesichts der rekrutierten Population stark geändert haben und da der aktuelle Wissensstand zu geeigneten Populationen für COVID-19-Studien bei nicht hospitalisierten Patienten berücksichtigt werden sollte, wird als primäre klinische Analysepopulation die Subgruppe von Patienten mit mindestens einem Risikofaktor für einen schweren COVID-19-Verlauf empfohlen. Die Gesamtpopulation von $n \geq 800$ sollte nur als sekundäre Analysepopulation dienen.

4. Es wird empfohlen, die bislang letzten beiden sekundären Endpunkte (Häufigkeit und Schwere von Symptomen) zum primären (Häufigkeit) und ersten sekundären Endpunkt (Schwere) zu machen und die Endpunkte zur Zeit bis zur vollständigen Symptomfreiheit als untergeordnete sekundäre Endpunkte weiterzuführen. Weiterhin ist eine Konzentration auf die Häufigkeit bzw. Schwere nach 2 Wochen empfehlenswert.

5. Um dem PCS angemessen Rechnung zu tragen, empfiehlt das DMB das Hochstufen des bislang explorativen Endpunkts zum PCS zu einem sekundären Endpunkt.
6. Aufgrund aktueller Erkenntnisse zum B-Zell-Metabolismus wird als immunologische Analysepopulation die Gesamtpopulation ($n \geq 800$) empfohlen, als nachgeordnete immunologische Analysepopulation sollte die die Subgruppe von Patienten mit mindestens einem Risikofaktor für einen schweren COVID-19-Verlauf dienen. Für evtl. Effekte einer Boosterimpfung muss in beiden Populationen stratifiziert werden.
7. Die Höhe der Antikörperspiegel gegen SARS-CoV-2 sollte als sekundärer Endpunkt untersucht werden.

12 Datenmanagement, Datenschutz und Umgang mit Biomaterialien

Die medizinischen Daten inkl. klinischer Routine-Befunde werden in dieser Studie nicht pseudonymisiert. Es erfolgt jedoch eine Pseudonymisierung des Patienten für die Studie mit einer Studien-Patienten-ID, sobald die Daten zur Auswertung freigegeben werden. Die Zuordnung der Patientenklardaten mit der Studien-Patienten-ID verlässt dabei das Rekrutierungszentrum nicht. Das Rekrutierungszentrum wird vom Kompetenznetz Darmerkrankungen e.V. betrieben, das die Studie zudem bei der Datenhaltung und durch die Gestellung geschulten Studienpersonals für die telefonische Datenakquise unterstützt. Außerhalb des Rekrutierungszentrums wird ausschließlich mit Studien-Patienten-IDs umgegangen.

In einer für diese Studie eingerichteten, web-basierten Case Report Form (CRF)-Datenbank werden die in Abschnitt 19.3 des Anhangs aufgeführten Informationen gesammelt. Dabei erfolgt die Eingabe der Daten verblindet.

Die Daten der Blut- und Stuhl-Analysen sowie der Virus-Sequenzierungen werden im Institut für Klinische Molekularbiologie (Kiel) in einer digitalen Datenbank pseudonymisiert gespeichert. Für die Blutuntersuchungen am UKSH werden Studienfälle im ORBIS angelegt. Die Datenspeicherung erfolgt selbstverständlich hinter der Firewall von CAU/UKSH und mit allen entsprechenden Sicherheitsvorkehrungen. Da das Projekt unter ärztlicher Leitung steht, unterliegen alle Mitarbeiter der ärztlichen Schweigepflicht. Eine Weitergabe der Daten an unberechtigte Dritte (insbesondere Arbeitgeber, Versicherungen) ist ausgeschlossen. Die Weitergabe von Proben und Informationen an wissenschaftliche Kooperationspartner erfolgt ausschließlich in pseudonymisierter Form, d.h. ohne Angaben zur Person. Bei Beendigung der Forschungsaktivitäten (frühestens nach 20 Jahren) werden die Proben und die dazu gehörenden Daten vernichtet. Auf Patientenwunsch erhalten diese eine Kopie mit ausführlicher Darstellung des Datenmanagements am Institut für Klinische Molekularbiologie. Diese entspricht den analytischen Standards, welche vom IHEC (International Human Epigenome Consortium; http://*ihec-epigenomes.org*) entwickelt wurden. Vor Analyse von Bioproben werden diese rekodiert und mit einem pseudonymisierten Datensatz verbunden.

Die Teilnahme an dieser klinischen Studie ist absolut freiwillig. Die Zustimmung kann jederzeit vom Patienten widerrufen werden. Daraus entstehen den Patienten keinerlei Nachteile. Gegebenenfalls bereits entnommene Proben werden dann unverzüglich vernichtet, und die Daten (außer bereits veröffentlichte/analysierte Daten) werden umgehend gelöscht, soweit technisch möglich (eine Löschung nach Auswertung ist nicht mehr möglich, darüber wird der Patient auch informiert). Einen etwaigen Widerruf der Zustimmung können die Patienten schriftlich an die Projektleitung der Klinik für Innere Medizin I, Universitätsklinikum Schleswig-Holstein, Campus Kiel, Rosalind-Franklin-Str. 12, Haus K1, D-24105 Kiel, richten.

Es kann sein, dass im Rahmen dieses Forschungsvorhabens Patente entstehen. In diesem Fall besteht kein individueller Patentanspruch der Patienten basierend auf deren individuellen Daten oder individuellen biologischen Materialien.

13 Datenverifizierung

Da die Eingabe der Ergebnisse der Telefonkontakte direkt in die Datenbank erfolgt, findet kein systematisches Monitoring statt. Explorative Daten werden nur zentral erhoben und unterliegen den in Abschnitt 12 beschriebenen Datenverarbeitungsstandards am Institut für Klinische Molekularbiologie (Kiel). Falls erforderlich, werden zusätzliche Daten von den behandelnden Ärzten abgefordert.

14 Protokolländerungen nach Studienbeginn

Protokoll-Amendments

Jede Veränderung dieses Protokolls wird in einem Protokoll-Amendment dokumentiert, und benötigt die Zustimmung des Studienleiters. Diese Protokoll-Amendments werden den zuständigen Ethikkommissionen mitgeteilt und im Falle wesentlicher Änderungen vorher zur Genehmigung vorgelegt.

Protokollabweichungen

Abweichungen von diesem Protokoll sollen vermieden werden. Falls eine Protokollabweichung stattfindet, beauftragt der Studienleiter einen Datenabgleich, und die Folgen der Abweichung werden überprüft und diskutiert. Jede Abweichung wird im Studienordner dokumentiert.

Frühzeitige Beendigung der Studie

Der Studienleiter hat das Recht, die Studie zu jeder Zeit zu beenden. In diesem Fall muss der Studienleiter sicherstellen, dass das weitere Entscheidungen dem bestmöglichen Wohl der Patienten entsprechen. Die Beendigung der Studie wird der zuständigen Ethikkommission mitgeteilt. Die Studie kann durch die verantwortlichen Kliniker beendet werden, falls

- die Fortführung der Studie aus ethischer oder medizinischer Sicht nicht zumutbar ist,
- die Ressourcen zur Fortführung der Studie nicht suffizient vorhanden sind, oder
- ein aussagekräftiges Resultat nicht als wahrscheinlich erscheint.

Einzelne Studienteilnehmer, dessen Gesundheit und Sicherheit gefährdet ist, können von beteiligten Klinikern nach deren Ermessen von der Studie ausgeschlossen werden.

15 Ethische Aspekte

Unabhängige Ethik-Kommission

Die unabhängige Ethik-Kommission der medizinischen Fakultät der CAU Kiel wird dieses Protokoll sowie die Probandeninformation und Probandeneinwilligung, deren Aktualisierungen (falls vorhanden) und jedes weitere den Patienten schriftlich ausgehändigte Material evaluieren.

End-of-Trial und End-of-Trial-Benachrichtigung

Das End-of-Trial ist definiert als Datum, an dem der letzte Studienteilnehmer an seiner letzten Studienvisite telefonisch kontaktiert wird. Am Ende der Studie wird die zuständige Ethik-Kommission hierüber benachrichtigt.

Ethische Durchführung der Studie

Diese klinische Studie wird gemäß ethischen Prinzipien in Anlehnung an die Deklaration von Helsinki und nach diesem genehmigten Protokoll durchgeführt.

16 Probandenversicherung

Für die Studie wird keine Probanden- oder Wegeunfallversicherung abgeschlossen. In der Patienteninformation wird ausdrücklich darauf hingewiesen, dass keine Probanden- oder Wegeunfallversicherung besteht. Laut Auskunft des UKSH-Versicherungsmaklers (Marsh Medical Consulting) und des zuständigen Versicherers (Berkshire Hathaway Specialty Insurance) vom 03.12.2020 deckt die Betriebshaftpflichtversicherung des UKSH die (extrem geringen) Risiken aus der Verwendung des nicht auf dem Markt befindlichen CICR-NAM im Rahmen der Studie ab.

17 Publikationsregeln

Nach Beendigung der Studie werden ein oder mehrere Manuskripte für gemeinsame Publikationen erstellt. Die Autorenschaft wird nach ICMJE-Kriterien vergeben (für eine offizielle Version siehe http://www.ICMJE.org). Die Autorenliste richtet sich nach den jeweiligen Richtlinien der entsprechenden Fachzeitschriften oder Kongressen. Auftragslabore haben bezüglich dieser Studie kein Publikationsrecht.

18 Unterschrift des Studienleiters

Kiel, 13.12.2021

Prof. Dr. med. Stefan Schreiber

19 Anhang

19.1 Patienteninformation

19.2 Aufbau der Online-Einwilligung

19.3 Case Report Form (CRF)

19.4 Fragebogen Fatigue (FACIT-F)

19.5 Fragebogen Lebensqualität (SF-36)

19.6 Zusatzfragebögen zur Nachbefragung nach 6 Monaten

19.7 Ergänzende Einwilligung für den Riechtest, die Zusatzfragebögen und den Kognitionstest zur Nachbefragung nach 6 Monaten

19.8 Ergänzende Einwilligung für Blutabnahmen zur Pharmakokinetik

19.9 Ergänzende Einwilligung zum Antikörpertest

CLINICAL TRIAL PROTOCOL

Improvement of the Nutritional Status Regarding Nicotinamide (Vitamin B3) and the Disease Course of COVID-19

Trial Code: COVit

Version: 1.1

Date: 26 March 2020

PI: Prof. Dr. Stefan Schreiber
Department of Internal Medicine I
University Medical Center Schleswig-Holstein, Campus Kiel
Arnold-Heller-Strasse 3, Building K1
24105 Kiel
Germany

Table of Contents

1. Summary of the Project
2. Responsibilities
3. Scientific Background
4. Project Objectives
    4.1. Primary Objective and Hypothesis
    4.2. Secondary Objectives and Exploratory Questions
5. Endpoints
6. Trial Design
7. Trial Population
8. Trial Procedures
    8.1. Informed Consent
    8.2. Measures (Intervention/Control) and Target Sizes
    8.3. Trial Duration
9. Risk-Benefit Assessment
10. Sample Size Calculation and Statistics
11. Sample Size Adaptation
12. Data Management, Data Protection and Handling of Biomaterials
13. Data Verification
14. Protocol Changes after Trial Start
15. Ethical Aspects
16. Subject Insurance
17. Publication Rules
18. Signature of the Principal Investigator
19. Appendix
    19.1. Patient Information
    19.2. Setup of the Online Informed Consent
    19.3. Case Report Form (CRF)

1 Summary of the Project

Based on the literature, it seems likely that a nutritional intervention with nicotinamide (a form of vitamin B3) can support the therapy of SARS-CoV-2 infection (COVID-19) by, *e.g.*, improving the availability of tryptophan and its metabolism-supporting metabolites (e.g. NAD). Five hundred patients each with confirmed SARS-CoV-2 infection and symptoms in the respiratory tract shall therefore self-administer 1,000 mg nicotinamide or 245 mg of the control substance silica per day in a blinded fashion for 4 weeks. The primary endpoint of the trial is the frequency of oxygen requirement, secondary endpoints are death, the frequencies of emergency department examination or hospitalisation and the time to resolution of symptoms. Patients are recruited after positive testing, receive the information for informed consent and can declare their participation via a website. After randomized distribution of the trial medication, the patients will be asked about their disease course in telephone interviews at baseline (week 0) and after 2, 4 and 6 weeks. From patients at UKSH giving global consent, serum samples to analyse levels of tryptophan, tryptophan metabolites and neopterin as well as stool samples to analyse the stool microbiome will be collected. The trial aims to generate rapid results on whether nicotinamide supplementation can alleviate the disease course of COVID-19.

2 Responsibilities

Principal Investigator of the trial:

Prof. Dr. med. Stefan Schreiber
Department of Internal Medicine I
University Medical Center Schleswig-Holstein (UKSH), Campus Kiel
Arnold-Heller-Strasse 3, Building K1, 24105 Kiel, Germany
Tel.: +49-431-500-15101, e-mail: s.schreiber@mucosa.de

Responsible centers and contacts:

Trial centers:

Department of Internal Medicine I, UKSH, Campus Kiel.

In addition, a large number of specialty hospitals or testing sites in Germany that treat COVID-19 patients will be contacted.

Participating trial laboratories:

Institute of Clinical Chemistry, UKSH, Campus Kiel (serum analyses).
Helmholtz GSF and Medical Center of the University of Munich (Germany) (serum analyses).
Institute of Clinical Molecular Biology, Kiel University and UKSH, Campus Kiel (stool microbiome analyses).

Telephone interviews:

Institute of Epidemiology (Popgen), Kiel University and UKSH, Campus Kiel (Prof. Dr. Wolfgang Lieb).
Competence Network Intestinal Diseases e.V., Kiel (Germany).

Data management and statistics:

Competence Network Intestinal Diseases e.V., Kiel (Germany).

Biobanking and sample management:

Institute of Clinical Molecular Biology, Kiel University and UKSH, Campus Kiel.

Funding:

The trial is funded by the Department of Internal Medicine I (UKSH, Campus Kiel). In parallel, attempts are being made to raise funds from the BMBF. The trial protocol will be registered in the German Register of Clinical Trials (DRKS) prior to the start of recruitment and will be registered at ClinicalTrials.gov.

3 Scientific Background

The availability of tryptophan and its metabolites, especially nicotinamide (a form of vitamin B3) as a building block of NAD and NADP, are important factors in chronic systemic inflammation. Here, an evolutionarily ancient defence mechanism can be misdirected by the body restricting energy use in cells analogous to iron reduction during inflammation (Boergeling & Ludwig 2017, FEBS J. 284:218). While this makes sense in simple organisms, such mechanisms lead to dysfunctions (e.g. inflammation-induced anaemia) in complex organisms. Reduced levels of nicotinamide and thus NAD(P) compromise cell metabolism, especially of macrophages and epithelial cells. The latter in particular are subject to a critically high rate of renewal in the gut and lungs. In addition, kynurenine, as the main degradation product of tryptophan, can also have a strong immune-activating effect. Another link between SARS-CoV-2 beyond inflammation and the immune system could also be the SARS-CoV-2 entry receptor ACE2, as the presence of ACE2 on the cell surface allows uptake of tryptophan via the receptor B0AT1. In severe courses of influenza, as in chronic inflammation, increased tryptophan degradation and thus increased kynurenine levels are observed, and inhibition of tryptophan degradation has beneficial effects in animal models (Pizzini et al. 2019, Influenza Other Respir. Viruses 13:603; Boergeling & Ludwig 2017, FEBS J. 284:218). In turn, there is evidence in a wide variety of virus types that nicotinamide can reduce viral replication and support the body's defence mechanisms, e.g. in vaccinia (Child et al. 1988, Virus Res. 9:119), HIV (Murray 2003, Clin. Infect. Dis. 36:453), enteroviruses (Moell et al. 2009, J. Med. Virol. 81:1082) or hepatitis B (Li et al. 2016, Arch. Virol. 161:621). Sufficient supply of B vitamins to strengthen the immune system is also recommended to combat SARS-CoV-2 infection (Zhang & Liu 2020, J. Med. Virol. 92:479). Here, the improvement of the defence against secondary bacterial infections in disease models is particularly emphasized (Zhang & Liu 2020, J. Med. Virol. 92:479). The reduced oxygen saturation observed in one study at a dose of 400 mg/kg (this would correspond to an arguably toxic dose of 28 g in a 70-kg person) has no significance for a dietary intervention with 1,000 mg <u>total</u> dose and given the acceptable daily intake of 900 mg/day (EFSA Panel on Dietetic Products, Nutrition and Allergies 2014, EFSA J. 12:3759). In the trial, 1,000 mg are administered, which is far below potentially harmful doses of several grams per day and very close to the ADI [OECD-SIDS: 3-pyridinecarboxamide (nicotinamide), SIDS Initial Assessment Report for SIAM 15, Boston, Massachusetts, 22-25 October 2002].

4 Project Objectives

4.1 Primary Objective and Hypothesis

The primary objective of the trial is to investigate the hypothesis that, under real clinical conditions, the frequency of severe disease courses in patients with COVID-19 decreases if they supplement 1,000 mg of nicotinamide.

4.2 Secondary Objectives and Exploratory Questions

In addition to the primary objective mentioned above, the secondary objectives of the trial are to investigate the frequencies of COVID-19-induced death, ventilator requirement, development of an ARDS and the time to complete resolution of symptoms in patients symptomatic at inclusion. Changes in serum levels of tryptophan, tryptophan metabolites and neopterin as well as in the stool microbiome between the beginning and the end of the intervention will be investigated in an exploratory fashion.

5 Endpoints

The disease course of the nicotinamide-supplemented group and the silica-supplemented group will be compared using the following endpoints:

Primary endpoint: frequency of hospitalization with a continuous oxygen requirement of at least 24 hours.

Secondary endpoints:

1. Frequency of ventilation requirement.
2. Frequency of death by COVID-19.
3. Frequency of examination in an emergency department.
4. Frequency of intensive care requirement.
5. Time from diagnosis to complete symptom resolution
6. Percentage of patients experiencing improvement of symptom severity at week 2 (endpoint 6A) or week 4 (endpoint 6B) (severe/moderate/mild).

Exploratory endpoints in UKSH patients:

1. Changes in serum levels of tryptophan.
2. Changes in serum levels of tryptophan metabolites.
3. Changes in serum levels of neopterin.
4. Changes in stool microbiome composition.

6 Trial Design

This is a monocentric, randomized trial in which symptomatic COVID-19 patients receive dietary supplementation with 1,000 mg nicotinamide per day in tablet form (2 x 500 mg NicoPel, IFC Germany/Derma Enzinger) or 245 mg silica in a capsule (1 x 245 mg silica capsule, Twardy/Saluspharma) in the control group. The less than perfect pairing of verum and placebo-like control is justified by the market availability of the investigational products and the tremendous urgency of the trial. Double blinding is ensured as far as possible. For the patients, it is not clear from the packaging and intake instructions of the dietary supplement sent to them which investigational product they received. Data collection personnel on the

telephones are instructed by SOP not to discuss the type of investigational product and are blinded as to the group allocation of the trial participants they are calling. All patients receive the usual clinical standard of care, the nutritional supplement is purely additive to the standard of care. It is assumed that the comparator group "remedy silica" has an effect size comparable to a placebo treatment, since silica consists mainly of inert silicon dioxide. However, positive statements on the nutritional benefit have also been established for silica as advertizable evaluations.

7 Trial Population

The trial population to be investigated, totalling 1,000 trial participants, involves patients with confirmed SARS-CoV-2 infection and symptoms in the respiratory tract.

Inclusion criteria are:

- SARS-CoV-2 infection confirmed by laboratory findings.
- Relevant infection symptoms in the respiratory or gastrointestinal tract (diarrhoea).
- The patient has been able to give written consent via a website before any trial procedure is performed and can comply with the trial-dependent prerequisites and requirements.
- The patient is of age (at least 18 years).

There are no exclusion criteria.

Recruitment will take place in Kiel, Germany, and in other specialty hospitals/testing sites for SARS-CoV-2 in Germany.

8 Trial Procedures

8.1 Informed Consent

Treatment decisions for COVID-19 are outside the trial protocol, i.e. the indication is independent and established without influence by the research program. Only after the indication has been established, a decision is made regarding possible recruitment.

All patients in the respective centers (outpatient and inpatient) who meet all inclusion and exclusion criteria will be asked to participate in the trial. There will be no trial-specific procedure outside the clinical standard in screening. Patients will receive a written patient information with informed consent. If necessary, a hotline is available for queries and a medical consultation for informed consent. After sufficient time for consideration, the patient can then consent online and document his or her consent by printing out the relevant page for him or herself (subject consent). This procedure also allows patients in quarantine at home to participate. Only after online consent, the first telephone contact is made for data collection for week 0 (baseline), wherein at the beginning the patient's ability to consent is checked again by study staff on the telephone according to an SOP. Again, the patient can ask further questions and the medical hotline can be consulted.

8.2 Measures (Intervention/Control) and Target Sizes

After consent and fulfilment of all requirements, patients are randomly assigned to the nicotinamide arm or the silica arm. The patient will be informed about this.

After randomization in a 1:1 ratio, control/interview calls are planned at week 0 (baseline) as well as week 2, week 4 and week 6. From patients at UKSH giving global consent, residual materials of serum and stool will be preserved for exploratory analyses of serum levels of

tryptophan, tryptophan metabolites and neopterin as well as of the composition of the stool microbiome.

The telephone interviews are conducted by the Institute of Epidemiology (Popgen). For medical questions, a physician from the Department of Internal Medicine I calls back. The following parameters are queried or entered during the calls:

Week 0:

- Personal and demographic data
- COVID-19-relevant and other symptoms
- Inclusion criteria
- Comorbidities
- Smoking status
- Chronic medication and supplementation
- Hospitalization
- Type of supplementation
- Epidemiological questions about living conditions

Weeks 2, 4 and 6:

- Intake of supplementation and other dietary supplements, if applicable.
- COVID-19-related and other symptoms
- Death and cause of death
- Smoking status
- Examination in an emergency department
- Hospitalization
- Ventilation requirement
- Intensive care treatment

The trial flow is summarized in the following flow chart:

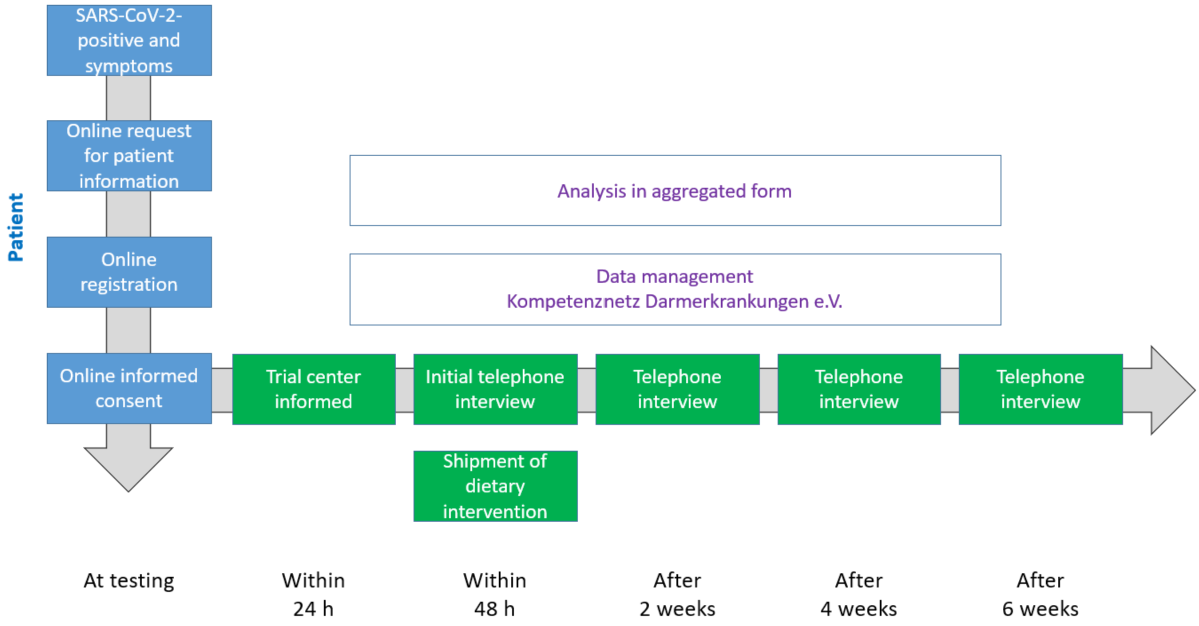

As part of the UKSH Global Consent for preserved residual biomaterials, serum and stool are preserved (only for UKSH patients).

8.3 Trial Duration

The trial duration for the individual patient is regularly 4 weeks until the end of the trial, potentially elongated to 6 weeks. A final interview takes place after approximately 6 months. The total duration of the trial will depend on the recruitment success and the further course of the COVID-19 epidemic. Enrolment shall start in April 2020. We anticipate enrolment of the last patient in summer 2020.

9 Risk-Benefit Assessment

If the optimization of the nicotinamide status helps at least some of the COVID-19 patients to overcome the disease more quickly and/or more easily, then – in the dramatically worsening situation – every patient who is not hospitalized or requires ventilation is a gain personally (intrinsic benefit) and also in relation to the totality of all COVID-19 patients (group benefit) and other patients in the hospital. The extraneous benefit is a gain in knowledge for clinical research (improvement of patient care by a simple, immediately available and safe nutritional supplement). Risks are not recognizable.

Patient supplementation is discontinued when a critical condition is reached and the patient is admitted to a monitoring area.

10 Sample Size Calculation and Statistics

The available data suggests that approximately 5% of already symptomatic COVID-19 patients could become hospitalized (due to oxygen therapy requirement) (primary endpoint). This would probably be the case in the silica-treated patient collective. The hypothesis is that this 5% event rate can be reduced to 2% with the use of nicotinamide.

Statistical analysis is performed using SAS software version 9.3 or later (SAS Institute, Inc., Cary, North Carolina). The statistical methodology is laid down in detail in a statistical analysis plan (SAP) in final form before the database lock. Any changes to the analysis plan in the protocol are documented in the SAP and presented in the final trial report. Continuous variables are summarized by descriptive statistics (number of observations (n), mean/median, standard deviation, minimum and maximum). Categorical variables are reported in frequency tables summarizing the number and percentage of patients in each category.

The intention-to-treat (ITT) population is defined as “all included patients”. The “all-randomized” (AR) population includes all patients who were observed for at least two weeks (intake of the dietary supplement and telephone interviews). The AR population is used to assess the primary and secondary endpoints. The biomarker (BM) population includes all patients from whom biomaterials could be obtained.

For sample size calculation, the standard variables were assumed to be: α-error = 0.05; power = 0.8; resulting in a K = 7.85 (factor calculated from α-error and power). The following simplified formula was used:

$$n = \frac{K\,[(R+1) - p_2\,(R^2+1)]}{p_2\,(1-R)^2}$$

n = required case number

p1 = Event rate in the treatment group (2%)

p2 = Event rate in the control group (5%)

R = relative risk (p1/p2) = here: 2% / 5% = 0.4

As a result, 607 patients per are were required. The case number calculation may be adjusted on the basis of the blinded event rate in the study.

The primary endpoint is tested with a two-sided significance level of 5% in the ITT population using Fisher's Exact Test. Secondary endpoints are tested sequentially with the same test until significance at the 5% level is no longer achieved. Thereafter, these are exploratory and are only analysed descriptively.

11 Sample Size Adaptation

After 500 completed patients, the frequency of the primary endpoint will be assessed in the blinded data. It is expected that approximately 37 patients will then have received hospital care with mandatory oxygen. If necessary, the sample size will be adjusted by increasing the number of patients.

12 Data Management, Data Protection and Handling of Biomaterials

Medical data incl. routine clinical findings will not be pseudonymized in this trial. However, a pseudonymization of the patient for the trial with a trial patient ID takes place as soon as the data are released for evaluation. In this process, the association of the patient data with the trial patient ID does not leave the recruitment center. The recruitment center is operated and supervised by the Institute of Epidemiology, which is supported by the Competence Network Intestinal Diseases e.V. in terms of data management and by providing trained trial personnel for telephone data acquisition. Outside the recruitment center, only trial patient IDs will be handled.

A web-based Case Report Form (CRF) database set up for this trial will collect the information listed in Section 19.3 of the Appendix. In this process, data entry is blinded.

The data of the blood and stool analyses are stored pseudonymized in a digital database at the Institute for Clinical Molecular Biology (Kiel). The data storage will of course take place behind the firewall of CAU/UKSH and with all appropriate security precautions. Since the project is under medical management, all employees are subject to medical confidentiality. Any disclosure of data to unauthorized third parties (especially employers, insurance companies) is excluded. The transfer of samples and information to scientific cooperation partners takes place exclusively in pseudonymized form, i.e. without personal details. At the end of the research activities (after 20 years at the earliest), the samples and the associated data are destroyed. At the patient's request, they will receive a copy with a detailed description of the data management at the Institute of Clinical Molecular Biology. This complies with the analytical standards developed by the IHEC (International Human Epigenome Consortium; *http://ihec-epigenomes.org*). Before analysis of biosamples, these are recoded and linked to a pseudonymized data set.

Participation in this clinical trial is absolutely voluntary. Consent can be revoked by the patient at any time. This does not result in any disadvantages for the patients. Any samples already taken will then be destroyed immediately, and the data (except for data already published/analysed) will be deleted immediately, as far as technically possible (deletion after evaluation is no longer possible, the patient will also be informed of this). Patients may address any revocation of consent in writing to the project management of the Department of Internal Medicine I, University Medical Center Schleswig-Holstein, Campus Kiel, Rosalind-Franklin-Str. 12, Building K1, D-24105 Kiel.

It is possible that patents may arise in the course of this research project. In this case, there is no individual patent claim of the patients based on their individual data or individual biological materials.

13 Data Verification

Since the results of the telephone contacts are entered directly into the database, no systematic monitoring takes place. Exploratory data are only collected centrally and are subject to the data processing standards at the Institute for Clinical Molecular Biology (Kiel) described in section 12. If necessary, additional data will be requested from the attending physicians.

14 Protocol Changes after Trial Start

Protocol Amendments

Any changes to this protocol will be documented in a Protocol Amendment and require the approval of the Principal Investigator. These Protocol Amendments will be communicated to the relevant ethics committees and, in the case of substantial changes, will be submitted for prior approval.

Protocol Deviations

Deviations from this protocol should be avoided. If a protocol deviation occurs, the Principal Investigator will order a data reconciliation and the consequences of the deviation will be reviewed and discussed. Any deviation will be documented in the study file.

Early Termination of the Trial

The Principal Investigator has the right to terminate the trial at any time. In this case, the Principal Investigator must ensure that further decision-making is in the best interests of the patients. Termination of the trial will be reported to the appropriate ethics committee. The trial may be terminated by the responsible clinicians if

- the continuation of the study is not reasonable from an ethical or medical point of view,
- the resources to continue the study are not sufficiently available, or
- a meaningful outcome does not appear likely.

Individual trial participants whose health and safety are at risk may be excluded from the trial by participating clinicians at their discretion.

15 Ethical Aspects

Independent Ethics Committee

The Independent Ethics Committee of the Medical Faculty of CAU Kiel will evaluate this protocol as well as the subject information and subject consent, their updates (if any) and any other material given to the patients in writing.

End of Trial and End-of-Trial Notification

The end of trial is defined as the date on which the last trial participant is contacted by telephone at their last trial visit. At the end of the trial, the responsible ethics committee is notified.

Ethical Conduct of the Trial

This clinical trial will be conducted according to ethical principles following the Declaration of Helsinki and according to this approved protocol.

16 Subject Insurance

No subject or commuting accident insurance will be taken out for the trial. It is explicitly stated in the patient information that there is no subject or commuting accident insurance.

17 Publication Rules

After completion of the trial, one or more manuscripts will be prepared for joint publications. Authorship will be assigned according to ICMJE criteria (for an official version, see http://www.ICMJE.org). The author list will follow the respective guidelines of the relevant journals or congresses. Contract laboratories do not have publication rights with respect to this trial.

18 Signature of the Principal Investigator

Kiel, ______________________

____

Prof. Dr. med. Stefan Schreiber

19 Appendix

19.1 Patient Information

19.2 Setup of the Online Informed Consent

19.3 Case Report Form (CRF)

KLINISCHES STUDIENPROTOKOLL

Verbesserung des Ernährungsstatus bezüglich Nicotinamid (Vitamin B3) und Verlauf der COVID-19-Erkrankung

Studienkürzel:
COVit

Version: 1.1

Datum: 26. März.2020

Projektleiter: Prof. Dr. Stefan Schreiber

Klinik für Innere Medizin I

Universitätsklinikum Schleswig-Holstein, Campus Kiel

Arnold-Heller-Straße 3, Haus K1/K3

24105 Kiel

Inhaltsverzeichnis

1. Zusammenfassung des Projektes
2. Verantwortlichkeiten
3. Wissenschaftlicher Hintergrund
4. Projektziele
    4.1. Primärziel und Hypothesen
    4.2. Sekundärziele und explorative Fragestellungen
5. Endpunkte
6. Studiendesign
7. Studienpopulation
8. Studienablauf
    8.1. Aufklärung und Einwilligung
    8.2. Maßnahmen (Intervention/Kontrolle) und Zielgrößen
    8.3. Studiendauer
9. Nutzen-Risiko-Abwägung
10. Fallzahlberechnung und Statistik
11. Anpassung der Stichprobengröße
12. Datenmanagement, Datenschutz und Umgang mit Biomaterialien
13. Datenverifizierung
14. Protokolländerungen nach Studienbeginn
15. Ethische Aspekte
16. Probandenversicherung
17. Publikationsregeln
18. Unterschrift des Studienleiters
19. Anhang:
    19.1. Patienteninformation
    19.2. Aufbau der Online-Einwilligung
    19.3. Case Report Form (CRF)

1 Zusammenfassung des Projekts

Aufgrund der Literatur erscheint es wahrscheinlich, dass eine Ernährungsintervention mit Nicotinamid (einer Form von Vitamin B3) die Therapie der SARS-CoV-2-Infektion (COVID-19) u.a. durch eine Verbesserung der Verfügbarkeit von Tryptophan und seinen stoffwechselunterstützenden Metaboliten (z.B. NAD) unterstützen kann. Je 500 Patienten mit bestätigter COVID-19-Infektion und Symptomen im Bereich der Atemwege sollen daher für 4 Wochen verblindet pro Tag 1.000 mg Nicotinamid oder 245 mg der Kontrollsubstanz Kieselerde einnehmen. Primärer Endpunkt der Studie ist die Häufigkeit von Beatmungspflichtigkeit, sekundäre Endpunkte sind Tod, die Häufigkeiten einer Untersuchung in einer Notaufnahme oder Hospitalisierung und die Zeit bis zum Verschwinden der Symptome. Die Patienten werden nach positiver Testung angesprochen, aufgeklärt und können über eine Webseite ihre Teilnahme erklären. Nach randomisierter Austeilung der Studienmedikation werden die Patienten zu Beginn (Woche 0) und nach 2, 4 und 6 Wochen Telefoninterviews zu ihrem Krankheitsverlauf befragt. Bei Patienten am UKSH werden im Rahmen des Global Consent Serumproben zur Analyse der Spiegel von Tryptophan, Tryptophan-Metaboliten und Neopterin sowie Stuhlproben zur Analyse des Stuhlmikrobioms gesammelt. Die Studie soll schnelle Ergebnisse dahingehend produzieren, ob eine Supplementation von Nicotinamid den Krankheitsverlauf von COVID-19 lindern kann.

2 Verantwortlichkeiten

Studienleiter:

Prof. Dr. med. Stefan Schreiber
Klinik für Innere Medizin I
Universitätsklinikum Schleswig-Holstein (UKSH), Campus Kiel
Arnold-Heller-Straße 3, Haus K1, 24105 Kiel
Tel.: 0431-500-15101, Email: s.schreiber@mucosa.de

Verantwortliche Zentren und Kontakte:

Studienzentren:

Klinik für Innere Medizin I, UKSH, Campus Kiel.

Zudem wird eine Vielzahl von Schwerpunktkrankenhäusern oder Teststellen in Deutschland angeschrieben, die COVID-19-Patienten behandeln.

Beteiligte Studien-Labore:

Institut für Klinische Chemie, UKSH, Campus Kiel (Serumanalysen).
Helmholz GSF und Klinikum der Universität München (Serumanalysen).
Institut für Klinische Molekularbiologie, CAU und UKSH, Campus Kiel (Stuhlmikrobiomanalysen).

Telefonische Visiten:

Institut für Epidemiologie (Popgen), CAU und UKSH, Campus Kiel (Prof. Dr. Wolfgang Lieb).
Kompetenznetz Darmerkrankungen e.V., Kiel.

Datenhaltung und Statistik:

Kompetenznetz Darmerkrankungen e.V., Kiel.

Biobanking und Sample Management:

Institut für Klinische Molekularbiologie, CAU und UKSH, Campus Kiel.

Finanzierung:

Die Studie wird aus Eigenmitteln der Klinik für Innere Medizin I (UKSH, Campus Kiel) finanziert. Parallel dazu wird versucht, Mittel vom BMBF einzuwerben. Das Studienprotokoll wird vor Beginn der Rekrutierung im Deutschen Register Klinischer Studien (DRKS) und unter ClinicalTrials.gov registriert.

3 Wissenschaftlicher Hintergrund

Die Verfügbarkeit von Tryptophan und seiner Metabolite, insbesondere Nicotinamid (einer Form von Vitamin B3) als Baustein von NAD und NADP, sind wichtige Faktoren bei chronischen systemischen Entzündungen. Hier kann ein evolutionär alter Verteidigungsmechanismus fehlgeleitet werden, indem der Körper die Energienutzung in Zellen analog zur Eisenreduktion bei Entzündung drosselt (Boergeling & Ludwig 2017, FEBS J. 284:218). Während dies in einfachen Organismen Sinn ergibt, führen solche Mechanismen in komplexen Organismen zu Dysfunktionen (z.B. entzündungsbedingte Anämie). Reduzierte Spiegel an Nicotinamid und damit NAD(P) behindern den Zellstoffwechsel, insbesondere auch der Makrophagen und Epithelzellen. Gerade letztere unterliegen einer kritisch hohen Erneuerungsrate im Darm und in der Lunge. Zudem kann Kynurenin als Hauptabbauprodukt des Tryptophan auch stark immunaktivierend wirken. Eine weitere Verbindung zwischen SARS-CoV-2 jenseits von Entzündung und Immunsystem könnte auch der SARS-CoV-2-Eintrittsrezeptor ACE2 sein, da die Anwesenheit von ACE2 auf der Zelloberfläche die Aufnahme von Tryptophan über den Rezeptor B0AT1 ermöglicht. Bei schweren Verläufen von Influenza werden ebenso wie bei chronischer Entzündung ein erhöhter Tryptophan-Abbau und damit erhöhte Kynurenin-Spiegel beobachtet, und Hemmung des Tryptophan-Abbaus hat im Tiermodell positive Effekte (Pizzini *et al.* 2019, Influenza Other Respir. Viruses 13:603; Boergeling & Ludwig 2017, FEBS J. 284:218). Im Gegenzug gibt es Hinweise bei unterschiedlichsten Virustypen, dass Nicotinamid die Virusreplikation reduzieren und die Abwehrmechanismen des Körpers unterstützen kann, z.B. bei Vaccinia (Child *et al.* 1988, Virus Res. 9:119), HIV (Murray 2003, Clin. Infect. Dis. 36:453), Enteroviren (Moell *et al.* 2009, J. Med. Virol. 81:1082) oder Hepatitis B (Li *et al.* 2016, Arch. Virol. 161:621). Auch für die Bekämpfung der Infektion mit SARS-CoV-2 wird ein hinreichender B-Vitamin-Status zur Stärkung des Immunsystems empfohlen (Zhang & Liu 2020, J. Med. Virol. 92:479). Hier wird besonders die Verbesserung der Abwehr bakterieller Sekundärinfektionen in Krankheitsmodellen hervorgehoben (Zhang & Liu 2020, J. Med. Virol. 92:479). Die in einer Studie beobachtete verminderte Sauerstoffsättigung bei einer Dosis von 400 mg/kg (dies entspräche einer wohl toxischen Dosis von 28 g bei einer 70-kg-Person) hat keine Aussagekraft für eine Nahrungsintervention mit 1.000 mg Gesamtdosis und angesichts des *acceptable daily intake* von 900 mg/Tag (EFSA Panel on Dietetic Products, Nutrition and Allergies 2014, EFSA J. 12:3759). In der Studie werden 1.000 mg verabreicht, was weit unter potentiell schädlichen Dosen von etlichen Gramm pro Tag und sehr nahe am ADI liegt [OECD-SIDS: 3-pyridinecarboxamide (nicotinamide), SIDS Initial Assessment Report for SIAM 15, Boston, Massachusetts, 22-25 October 2002].

4 Projektziele

4.1 Primärziel und Hypothesen

Das Primärziel der Studie ist die Untersuchung der Hypothese, dass unter realen klinischen Bedingungen die Häufigkeit der schweren Verläufe bei COVID-19-Erkrankten sinkt, wenn sie 1.000 mg Nicotinamid supplementieren.

4.2 Sekundärziele und explorative Fragestellungen

Neben dem genannten Primärziel sind die Sekundärziele der Studie die Untersuchung der Häufigkeit von COVID-19-induziertem Tod, die Häufigkeit einer Beatmung, die Häufigkeit der Entwicklung eins ARDS und die Zeit bis zur vollständigen Symptomfreiheit der bei Einschluss symptomatischen Patienten. Explorativ sollen die Entwicklung der Spiegel von Tryptophan, Tryptophanmetaboliten und Neopterin im Serum sowie des Stuhlmikrobioms zwischen Beginn und Ende der Behandlung untersucht werden.

5 Endpunkte

Der Krankheitsverlauf der Nicotinamid-supplementierten Gruppe und der Kieselerde-supplementierten Kontrollgruppe wird anhand folgender Endpunkte verglichen:

Primärer Endpunkt: Häufigkeit einer Hospitalisierung mit einer durchgehenden Sauerstoffpflicht von mindestens 24 Stunden.

Sekundäre Endpunkte:

1. Häufigkeit von Beatmungspflicht.
2. Häufigkeit von Tod durch COVID-19.
3. Häufigkeit von Untersuchung in einer Notaufnahme.
4. Häufigkeit von Intensivpflicht.
5. Zeit von der Diagnose bis zur Symptomfreiheit.
6. Prozentsatz der Patienten, die Verbesserung der Symptomschwere zur Woche 2 (Endpunkt 6A) oder Woche 4 (6B) erfahren (schwer/moderat/mild).

Explorative Endpunkte bei UKSH-Patienten:

1. Änderung der Spiegel von Tryptophan im Serum.
2. Änderung der Spiegel von Tryptophan-Metaboliten im Serum.
3. Änderung der Spiegel von Neopterin im Serum.
4. Änderung der Zusammensetzung des Stuhlmikrobioms.

6 Studiendesign

Es handelt sich hierbei um eine monozentrische, randomisierte Studie, bei der symptomatische COVID-19-Patienten eine Nahrungsergänzung mit 1.000 mg Nicotinamid pro Tag in Tablettenform (2 x 500 mg NicoPel, IFC Deutschland/Derma Enzinger) oder in der Kontrollgruppe 245 mg Kieselerde in einer Kapsel (1 x 245 mg Kieselerde-Kapsel, Twardy/Saluspharma) erhalten. Die nicht perfekte Paarung von Verum und Placebo-ähnlicher Kontrolle ist durch die Marktverfügbarkeit der Prüfpräparate und die enorme Dringlichkeit der Studie zu rechtfertigen. Eine doppelte Verblindung ist soweit wie möglich gewährleistet. Für die Patienten geht aus der Verpackung und Einnahmeanweisung des zugesandten

Nahrungsergänzungsmittels nicht hervor, welches Präparat sie bekommen haben. Das datenerhebende Personal an den Telefonen wird der SOP angewiesen, die Art des Prüfpräparats nicht zu thematisieren und ist hinsichtlich der Gruppenzugehörigkeit der angerufenen Studienteilnehmer verblindet. Alle Patienten erhalten den üblichen klinischen Behandlungsstandard, die Nahrungsergänzung ist rein additiv zum Standard of Care. Es wird davon ausgegangen, dass die Vergleichsgruppe „Heilmittel Kieselerde“ eine einer Placebobehandlung vergleichbare Effektstärke hat, da Kieselerde überwiegend aus inertem Siliziumdioxid besteht. Allerdings sind auch für Kieselerde positive Aussagen über den Ernährungsbenefit als bewerbbare Beurteilungen etabliert.

7 Studienpopulation

Die zu untersuchende Studienpopulation von insgesamt 1.000 Studienteilnehmern betrifft Patienten mit bestätigter SARS-CoV-2-Infektion und Symptomen im Bereich der Atemwege.

Die Einschlusskriterien sind hierbei:

- Durch Laborbefund gesicherte SARS-CoV-2-Infektion.
- Einschlägige Infektsymptome im Bereich der Atemwege oder des Gastrointestinaltrakts (Durchfall).
- Der Patient/die Patientin konnte vor Durchführung jeglicher Studien-Prozedur schriftlich über eine Webseite einwilligen und kann die Studien-abhängigen Voraussetzungen und Anforderungen einhalten.
- Der Patient/die Patientin ist volljährig (vollendetes 18. Lebensjahr).

Ausschlusskriterien gibt es nicht.

Die Rekrutierung findet in Kiel und in weiteren Schwerpunktkrankenhäusern/Teststellen für SARS-CoV-2 in Deutschland statt.

8 Studienablauf

8.1 Aufklärung und Einwilligung

Die Therapieentscheidungen bei der Behandlung von COVID-19 liegen außerhalb des Studienprotokolls, d. h. die Indikationsstellung ist unabhängig und wird ohne Einfluss durch das Forschungsprogramm gestellt. Erst nach Indikationsstellung wird über eine mögliche Rekrutierung entschieden.

Alle Patienten in den jeweiligen Zentren (ambulant und stationär), welche alle Ein- und Ausschlusskriterien erfüllen, werden um eine Studienteilnahme gebeten. Eine studienspezifische Prozedur außerhalb des klinischen Standards im Screening erfolgt nicht. Die Patienten erhalten eine schriftliche Patienteninformation mit Aufklärung. Für Rückfragen und ein ärztliches Aufklärungsgespräch steht bei Bedarf eine Hotline zur Verfügung. Der Patient kann im Anschluss nach ausreichender Bedenkzeit online einwilligen und seine Einwilligung durch Ausdruck der entsprechenden Seite für sich dokumentieren (Probandeneinwilligung). Dieses Vorgehen ermöglicht auch die Teilnahme von Patienten in häuslicher Quarantäne. Erst nach der Online-Einwilligung erfolgt der erste Telefonkontakt zur Datenerhebung für Woche 0, bei dem zu Beginn die Fähigkeit zur Einwilligung des Patienten durch das Studienpersonal am Telefon noch einmal nach einer SOP überprüft wird. Auch hier kann der Patient weitere Rückfragen stellen und die ärztliche Hotline kann hinzugezogen werden.

8.2 Maßnahmen (Intervention/Kontrolle) und Zielgrößen

Nach der Einwilligung und Erfüllung aller Voraussetzungen werden die Patienten nach dem Zufallsprinzip dem Nicotinamid-Arm oder dem Kieselerde-Arm zugeordnet. Darüber wird der Patient aufgeklärt.

Nach der Randomisierung im Verhältnis 1:1 sind Kontrollanrufe zu Woche 0, Woche 2, Woche 4 und Woche 6 geplant. Bei UKSH-Patienten werden im Rahmen des Global Consent Restmaterialien Serum und Stuhl für explorative Analysen der Serumspiegel von Tryptophan, Tryptophan-Metaboliten und Neopterin sowie der Zusammensetzung des Stuhlmikrobioms asserviert.

Die Telefoninterviews werden vom Institut für Epidemiologie (Popgen) geführt. Bei medizinischen Fragen ruft ein Arzt aus der Klinik für Innere Medizin I zurück. Bei den Anrufen werden folgende Parameter abgefragt bzw. eingetragen:

Woche 0:

- Persönliche und demographische Daten
- COVID-19-relevante und andere Symptome
- Einschlusskriterien
- Komorbiditäten
- Raucherstatus
- Chronische Medikation und Supplementierung
- Hospitalisierung
- Art der Supplementierung
- Epidemiologische Fragen zu den Lebensumständen

Woche 2, 4 und 6:

- Einnahme der Supplementierung und ggf. anderer Nahrungsergänzungsmittel
- COVID-19-relevante und andere Symptome
- Tod und Todesursache
- Raucherstatus
- Untersuchung in einer Notaufnahme
- Hospitalisierung
- Beatmungspflicht
- Intensivstationäre Behandlung

Der Ablauf der Studie wird in folgendem Flow Chart zusammengefasst:

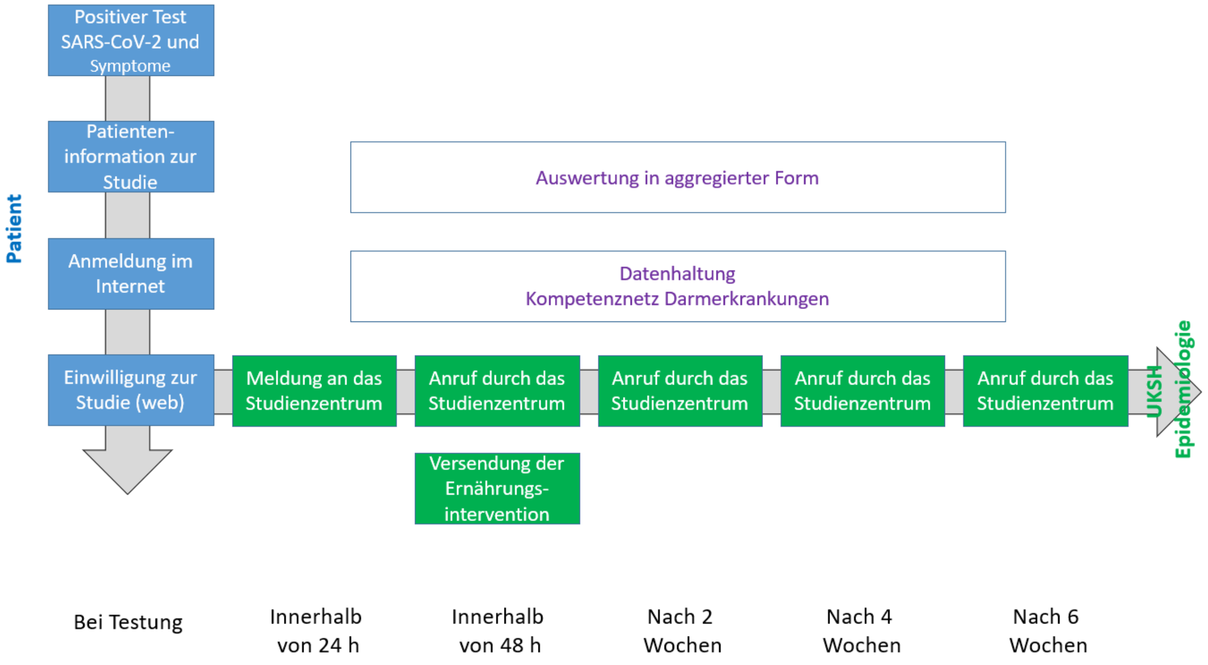

Im Rahmen des UKSH-Global-Consent zu asservierten Rest-Biomaterialien werden Serum und Stuhl asserviert (nur bei UKSH-Patienten).

8.3 Studiendauer

Die Studiendauer für den einzelnen Patienten beträgt regulär 4 Wochen bis zum Studienende, ggf. mit Verlängerung auch 6 Wochen. Ein Abschlussgespräch erfolgt nach ca. 6 Monaten. Die Gesamtdauer der Studie richtet sich nach dem Rekrutierungserfolg und dem weiteren Verlauf der COVID-19-Epidemie. Die Rekrutierung soll im April 2020 starten. Wir rechnen mit einem Einschluss des letzten Patienten im Sommer 2020.

9 Nutzen-Risiko-Abwägung

Wenn die Optimierung des Nicotinamid-Status zumindest einem Teil der COVID-19-Erkrankten dabei hilft, die Krankheit schneller und/oder leichter zu überwinden, ist bei der sich dramatischen zuspitzenden Lage jeder nicht hospitalisierte oder beatmungspflichtige Patient persönlich (Eigennutzen) und auch bezogen auf die Gesamtheit aller COVID-19-Patienten (Gruppennutzen) und anderen Patienten im Krankenhaus ein Gewinn. Der Fremdnutzen besteht in einem Erkenntnisgewinn für die klinische Forschung (Verbesserung der Krankenversorgung durch ein einfaches, sofort verfügbares und sicheres Nahrungsergänzungsmittel). Risiken sind nicht erkennbar.

Die Supplementation von Patienten wird bei Erreichen eines kritischen Zustands und Aufnahme des Patienten in einen Überwachungsbereich abgebrochen.

10 Fallzahlberechnung und Statistik

Die bisherige Datenlage lässt annehmen, dass rund 5 % der bereits symptomatischen COVID-Patienten krankenhauspflichtig (wegen Bedarfs an Sauerstofftherapie) werden könnten (primärer Endpunkt). Dies wäre im Kollektiv der mit Kieselerde behandelten Patienten voraussichtlich der Fall. Die Hypothese ist, dass diese Ereignisrate von 5 % durch den Einsatz von Nicotinamid auf 2 % gesenkt werden kann.

Die statistische Analyse wird mit SAS-Software Version 9.3 oder neuer (SAS Institute, Inc., Cary, North Carolina) durchgeführt. Die statistische Methodologie wird im Detail in einem statistischen Analyseplan (SAP) in finaler Form vor dem Database-Lock niedergelegt. Jegliche Änderung des Analyseplans im Protokoll wird im SAP dokumentiert und im finalen Studienbericht dargestellt. Kontinuerliche Variablen werden mit einer deskriptiven Statistik zusammengefasst (Zahl der Beobachtungen (n), Mittelwert/Median, Standardabweichung, Minimum und Maximum). Kategorische Variablen werden in Frequency Tables berichtet, welche die Zahl und den Prozentsatz von Patienten in den jeweiligen Kategorien zusammenfassen.

Die „Intention-to-treat" (ITT)-Population ist definiert als „alle eingeschlossenen Patienten". Die „All-Randomized" (AR)-Population umfasst alle Patienten, die für mindestens zwei Wochen beobachtet wurden (Einnahme des Nahrungsergänzungsmittels und Telefoninterviews). Die AR-Population wird für die Beurteilung der primären und sekundären Endpunkte genutzt. Die Biomarker (BM)-Population umfasst alle Patienten, bei denen Biomaterialien asserviert werden konnten.

Für die Fallzahlplanung werden als Standardgrößen angenommen: α-Fehler = 0,05; Power = 0,8; es ergibt sich ein K = 7,85 (Faktor, der sich aus α-Fehler und Power berechnet). Die folgende vereinfachte Formel wurde benutzt:

$$n = \frac{K\,[(R+1) - p_2\,(R^2+1)]}{p_2\,(1-R)^2}$$

n = benötigte Fallzahl
p1 = Ereignisrate in der Behandlungsgruppe (2 %)
p2 = Ereignisrate in der Kontrollgruppe (5 %)
R = relatives Risiko (p1/p2) = hier: 2 % / 5% = 0,4

Im Ergebnis werden 607 Patienten pro Gruppe benötigt. Die Fallzahlberechnung wird ggf. anhand der verblindet ermittelten Ereignisrate in der Studie angepasst.

Der primäre Endpunkt wird mit einem zwei-seitigen Signifikanz-Niveau von 5 % in der ITT-Population mit dem Fisher's Exact Test untersucht. Sekundäre Endpunkte werden mit demselben Test sequentiell getestet, bis eine Signifikanz auf dem 5-%-Niveau nicht mehr erreicht wird. Danach sind diese exploratorisch und werden nur deskriptiv ausgewertet.

11 Anpassung der Stichprobengröße

Nach 500 abgeschlossenen Patienten wird die Frequenz des primären Endpunkts in den verblindeten Daten beurteilt. Es wird erwartet, dass dann ca. 37 Patienten eine Krankenhauspflege mit Sauerstoffpflicht erhalten haben. Gegebenenfalls erfolgt eine Anpassung der Stichprobengröße mit Erweiterung der Patientenzahl.

12 Datenmanagement, Datenschutz und Umgang mit Biomaterialien

Die medizinischen Daten inkl. klinischer Routine-Befunde werden in dieser Studie nicht pseudonymisiert. Es erfolgt jedoch eine Pseudonymisierung des Patienten für die Studie mit einer Studien-Patienten-ID, sobald die Daten zur Auswertung freigegeben werden. Die Zuordnung der Patientenklardaten mit der Studien-Patienten-ID verlässt dabei das Rekrutierungszentrum nicht. Das Rekrutierungszentrum wird vom Institut für Epidemiologie betrieben und beaufsichtigt, das vom Kompetenznetz Darmerkrankungen e.V. bei der Datenhaltung und durch die Gestellung geschulten Studienpersonals für die telefonische

Datenakquise unterstützt wird. Außerhalb des Rekrutierungszentrums wird ausschließlich mit Studien-Patienten-IDs umgegangen.

In einer für diese Studie eingerichteten, web-basierten Case Report Form (CRF)-Datenbank werden die in Abschnitt 19.3 des Anhangs aufgeführten Informationen gesammelt. Dabei erfolgt die Eingabe der Daten verblindet.

Die Daten der Serum- und Stuhl-Analysen werden im Institut für Klinische Molekularbiologie (Kiel) in einer digitalen Datenbank pseudonymisiert gespeichert. Die Datenspeicherung erfolgt selbstverständlich hinter der Firewall von CAU/UKSH und mit allen entsprechenden Sicherheitsvorkehrungen. Da das Projekt unter ärztlicher Leitung steht, unterliegen alle Mitarbeiter der ärztlichen Schweigepflicht. Eine Weitergabe der Daten an unberechtigte Dritte (insbesondere Arbeitgeber, Versicherungen) ist ausgeschlossen. Die Weitergabe von Proben und Informationen an wissenschaftliche Kooperationspartner erfolgt ausschließlich in pseudonymisierter Form, d.h. ohne Angaben zur Person. Bei Beendigung der Forschungsaktivitäten (frühestens nach 20 Jahren) werden die Proben und die dazu gehörenden Daten vernichtet. Auf Patientenwunsch erhalten diese eine Kopie mit ausführlicher Darstellung des Datenmanagements am Institut für Klinische Molekularbiologie. Diese entspricht den analytischen Standards, welche vom IHEC (International Human Epigenome Consortium; http://*ihec-epigenomes.org*) entwickelt wurden. Vor Analyse von Bioproben werden diese rekodiert und mit einem pseudonymisierten Datensatz verbunden.

Die Teilnahme an dieser klinischen Studie ist absolut freiwillig. Die Zustimmung kann jederzeit vom Patienten widerrufen werden. Daraus entstehen den Patienten keinerlei Nachteile. Gegebenenfalls bereits entnommene Proben werden dann unverzüglich vernichtet, und die Daten (außer bereits veröffentlichte/analysierte Daten) werden umgehend gelöscht, soweit technisch möglich (eine Löschung nach Auswertung ist nicht mehr möglich, darüber wird der Patient auch informiert). Einen etwaigen Widerruf der Zustimmung können die Patienten schriftlich an die Projektleitung der Klinik für Innere Medizin I, Universitätsklinikum Schleswig-Holstein, Campus Kiel, Rosalind-Franklin-Str. 12, Haus K1, D-24105 Kiel, richten.

Es kann sein, dass im Rahmen dieses Forschungsvorhabens Patente entstehen. In diesem Fall besteht kein individueller Patentanspruch der Patienten basierend auf deren individuellen Daten oder individuellen biologischen Materialien.

13 Datenverifizierung

Da die Eingabe der Ergebnisse der Telefonkontakte direkt in die Datenbank erfolgt, findet kein systematisches Monitoring statt. Explorative Daten werden nur zentral erhoben und unterliegen den in Abschnitt 12 beschriebenen Datenverarbeitungsstandards am Institut für Klinische Molekularbiologie (Kiel). Falls erforderlich, werden zusätzliche Daten von den behandelnden Ärzten abgefordert.

14 Protokolländerungen nach Studienbeginn

Protokoll-Amendments

Jede Veränderung dieses Protokolls wird in einem Protokoll-Amendment dokumentiert, und benötigt die Zustimmung des Studienleiters. Diese Protokoll-Amendments werden den zuständigen Ethikkommissionen mitgeteilt und im Falle wesentlicher Änderungen vorher zur Genehmigung vorgelegt.

Protokollabweichungen

Abweichungen von diesem Protokoll sollen vermieden werden. Falls eine Protokollabweichung stattfindet, beauftragt der Studienleiter einen Datenabgleich, und die Folgen der Abweichung werden überprüft und diskutiert. Jede Abweichung wird im Studienordner dokumentiert.

Frühzeitige Beendigung der Studie

Der Studienleiter hat das Recht, die Studie zu jeder Zeit zu beenden. In diesem Fall muss der Studienleiter sicherstellen, dass das weitere Entscheidungen dem bestmöglichen Wohl der Patienten entsprechen. Die Beendigung der Studie wird den zuständigen Ethikkommissionen mitgeteilt. Die Studie kann von einzelnen Studienzentren durch die verantwortlichen Kliniker beendet werden, falls

- die Fortführung der Studie aus ethischer oder medizinischer Sicht nicht zumutbar ist,
- die Ressourcen zur Fortführung der Studie nicht suffizient vorhanden sind, oder
- ein aussagekräftiges Resultat nicht als wahrscheinlich erscheint.

Einzelne Studienteilnehmer, dessen Gesundheit und Sicherheit gefährdet ist, können von beteiligten Klinikern nach deren Ermessen von der Studie ausgeschlossen werden.

15 Ethische Aspekte

Unabhängige Ethik-Kommission

Die unabhängige Ethik-Kommission der medizinischen Fakultät der CAU Kiel wird dieses Protokoll sowie die Probandeninformation und Probandeneinwilligung, deren Aktualisierungen (falls vorhanden) und jedes weitere den Patienten schriftlich ausgehändigte Material evaluieren.

End-of-Trial und End-of-Trial-Benachrichtigung

Das End-of-Trial ist definiert als Datum, an dem der letzte Studienteilnehmer an seiner letzten Studienvisite telefonisch kontaktiert wird. Am Ende der Studie werden die zuständigen Ethik-Kommissionen hierüber benachrichtigt.

Ethische Durchführung der Studie

Diese klinische Studie wird gemäß ethischen Prinzipien in Anlehnung an die Deklaration von Helsinki und nach diesem genehmigten Protokoll durchgeführt.

16 Probandenversicherung

Für die Studie wird keine Probanden- oder Wegeunfallversicherung abgeschlossen. In der Patienteninformation wird ausdrücklich darauf hingewiesen, dass keine Probanden- oder Wegeunfallversicherung besteht.

17 Publikationsregeln

Nach Beendigung der Studie werden ein oder mehrere Manuskripte für gemeinsame Publikationen erstellt. Die Autorenschaft wird nach ICMJE-Kriterien vergeben (für eine offizielle Version siehe http://www.ICMJE.org). Die Autorenliste richtet sich nach den jeweiligen Richtlinien der entsprechenden Fachzeitschriften oder Kongressen. Auftragslabore haben bezüglich dieser Studie kein Publikationsrecht.

18 Unterschrift des Studienleiters

Kiel, 26.3.2020

Prof. Dr. med. Stefan Schreiber

19 Anhang

19.1 Patienteninformation

19.2 Aufbau der Online-Einwilligung

19.3 Case Report Form (CRF)

6. Statistical analysis plan for acute COVID-19

This section contains the statistical analysis plan for final analyses for acute COVID-19 in the final version 1.1 of 27 July 2022 (35 pp.).

STATISTICAL ANALYSIS PLAN

Improvement of the nutritional status regarding nicotinamide (vitamin B3) and the disease course of COVID-19

COVit-2

Final analyses for acute COVID-19

Type of study: Interventional (Clinical Trial)

Biometrician: Regina Hollweck (Novustat)

Version number: 1.1

Date of issue: 27 July 2022

Type of report: Final Analysis

Version History

SAP Version History Summary

SAP Version	Date	Change	Rationale
1	27 April 2022	Not applicable	Initial release
1.1	27 July 2022	1. Differentiation ITT and safety population<br>2. Primary analysis population RFITT<br>3. Subgroup analysis for complementary subgroups<br>4. Further specification of subgroups cardiovascular diseases<br>5. Definition of responders<br>6. Baseline description<br>7. Expanded description on missing data and collection of safety information<br>8. Duration of hospitalization as additional exploratory endpoint<br>9. Analyses of SF-36 and FACIT-F questionnaires for RFITT and RFPP with severe baseline scores and subscores (below or equal to the median)	1. Safety analysis will include also patients without intake of study medication<br>2. More robust than RFPP<br>3. Complementary subgroups facilitate interpretation of results and are the gold standard for subgroup analyses<br>4. Patients with stroke show other behaviour in disease progress in newly released scientific publication<br>5. Responder analysis as additional exploratory analysis<br>6. Shortening of variable list for baseline description<br>7. Clarification of procedures<br>8. Important health economic endpoint<br>9. Focus on patients with moderate/severe baseline symptomatology.

Signatures for Approval

27/07/22

(Date) (Regina Hollweck, Trial Statistician)

02 AUG 2022

(Date) (Prof. Dr. Stefan Schreiber, Principal Investigator)

Table of Contents

1. Introduction 9
2. Study Objectives 10
2.1. Primary Objective of the Main Study 10
2.2. Secondary Objectives 11
2.3. Exploratory Objectives 11
3. Trial Methods 11
3.1. Trial Design 11
3.2. Randomization and Blinding 11
3.3. Sample Size 12
3.4. Framework 12
3.5. Investigational Product 13
3.5.1. Nicotinamide Group 13
3.5.2. Control Group 13
3.6. Study Duration and Schedule 13
4. Statistical Principles 14
4.1. Confidence Intervals and P-Values 14
4.2. Adherence and Protocol Deviations 14
4.3. Analysis Populations 15
5. Trial population 16
5.1. Eligibility 16
5.1.1. Inclusion Criteria 16
5.1.2. Exclusion Criteria 16
5.2. Recruitment 16
5.3. Withdrawal/Follow-up 17
6. Analysis 17
6.1. Outcome Definition 17
6.1.1. Primary Outcomes 17
6.1.2. Secondary Outcomes 18
6.1.3. Exploratory Outcomes 19
6.2. Baseline Patient Characteristics 20
6.3. Prior/Concomitant Medication and Supplementation 20
7. Analysis Methods 21
7.1. Pre-Processing 21

7.1.1. Interview Data....21
7.1.2. Daily Symptom Questionnaire....21
7.1.3. FACIT-F....22
7.1.4. SF-36....22
7.2. Main Analysis....22
7.3. Subgroups....25
7.4. Final Analyses and Reporting....25
7.5. Missing Data....26
7.6. Multiple Testing Procedure....26
7.7. Statistical Software....27
8. Safety....27
9. References....29
Appendix A: Summary of Analyses....34

List of Abbreviations

ACE2 .... Angiotensin-converting enzyme 2
AR(1) .... Autoregression of lag 1
B0AT1 .... Sodium-dependent neutral amino acid transporter
CI .... Confidence interval
CICR-NAM .... Controlled-ileocolonic-release nicotinamide
DMB .... Data Management Board
FACIT-F .... Functional Assessment of Chronic Illness Therapy – Fatigue
ITT .... Intention-to-treat
LS .... Least square
M .... Mean
MAR .... Missing at random
MedDRA .... Medical Dictionary for Regulatory Activities
MMRM .... Mixed model repeated measures
N .... Number of observations
NAD .... Nicotinamide adenine dinucleotide
NADP .... Nicotinamide adenine dinucleotide phosphate
RFITT .... Risk factor intention-to-treat
RFPP .... Risk factor per protocol
SAP .... Statistical Analysis Plan
SARS-CoV-2 .... Severe acute respiratory syndrome coronavirus type 2
SD .... Standard deviation
SF-36 .... RAND 36-Item Health Survey
WHO .... World Health Organisation

Trial Summary

**Principal Investigator**	Prof. Dr. Stefan Schreiber and Prof. Dr. Matthias Laudes<br>Department of Internal Medicine I<br>University Medical Center Schleswig-Holstein (UKSH), Campus Kiel<br>Arnold-Heller-Str. 3<br>24105 Kiel<br>Telephone: +49 431 500-22201<br>Fax: +49 431 500-22204<br>E-mail: s.schreiber@mucosa.de
**Sponsor**	University Medical Center Schleswig-Holstein (UKSH), Campus Kiel<br>Arnold-Heller-Str. 3<br>24105 Kiel
**Title**	Improvement of the nutritional status regarding nicotinamide (vitamin B3) and the disease course of COVID-19
**Protocol Title**	Verbesserung des Ernährungsstatus bezüglich Nicotinamid (Vitamin B3) und Verlauf der COVID-19-Erkrankung
**Acronym**	COVit-2
**Background**	Nicotinamide, a form of vitamin B3, plays an important role in chronic systemic inflammation and reduced circulating levels impede immune defense against coronaviruses. We also assume an anti-inflammatory barrier-strengthening property. Results from a pilot study suggest a potential efficacy of nicotinamide on the resolution of COVID-19 symptoms. Confirmatory data are needed to determine whether nicotinamide is effective in reducing the symptom burden in patients with COVID-19.
**Study Design**	Double-blind, placebo-controlled, randomized clinical trial.
**Intervention Group**	Nicotinamide, 1,000 mg/day p.o. (one each of 500-mg immediate-release and delayed-release nicotinamide tablets), for 4 weeks.
**Control Group**	Placebo, p.o. (2 tablets), for 4 weeks.
**Randomization**	Patients are randomized continuously in a 1:1 ratio according to a randomization scheme in which every second patient is

	randomized and the following one then receives the opposite allocation.
**Blinding**	Quadruple (Participant, Care Provider, Investigator, Outcomes Assessor)
**Primary Analysis**	Comparison of the change to baseline of the primary outcome between patients randomized to nicotinamide versus placebo using the Cochran-Mantel-Haenszel test for change of binary variables. Post hoc Fisher Exact test will be applied to compare the effect at week 2 (primary analysis time point). For the futility population, a total of 4 primary key symptoms will be analyzed using a hierarchical testing procedure. For continuous or ordinal scaled variables, a mixed effect model repeat measurement (MMRM) with fixed effect terms for baseline value, treatment group, time (categorical variables) and treatment-by-time interaction (metric or ordinal variables) will be used.
**Primary Endpoint of the Acute Disease Phase**	Frequencies of key individual COVID-19 symptoms at week 0 (baseline), week 2 (primary time of analysis), week 4 and week 6:<br>1. Performance drop (yes/no).<br>For the futility population, additional key symptoms will be analyzed as part of the primary endpoint:<br>2. Ability to perform normal activities (quantified by the complaint scale for lower respiratory tract infections);<br>3. Cough (quantified by the complaint scale);<br>4. Fatigue (yes/no).

1. Introduction

The present document aims at detailing the statistical analyses that will be performed for the study DRKS00021214 (NCT04751604) with the data from the acute disease phase of COVID-19 (week 0 to week 6) on the basis of the current study protocol (Version 2.3, dated 13 Dec 2021). This document has been written by the lead statistician and reviewed and agreed by the Principal Investigator of the trial.

The availability of tryptophan and its metabolites, especially nicotinamide (a form of vitamin B3) as a building block of NAD and NADP, are important factors in the immune system. Dysregulation can lead to immune deficiency, but also to chronic inflammation (1-5). Particularly in the context of pneumonia, certain metabolites of tryptophan are important and can strengthen the immune functions of the lungs and counteract chronic changes caused by inflammatory reactions in the lungs (6-12). Nicotinamide supplementation has a potent anti-inflammatory effect in both tryptophan-deficient and normally fed animals (13-16). Reduced levels of nicotinamide and thus NAD(P) also impede immune defense against coronaviruses (17) and cellular metabolism, especially in macrophages and epithelial cells. The latter in particular are subject to a critically high rate of renewal in the gut and lungs. Another link to SARS-CoV-2 beyond inflammation and the immune system may also be the SARS-CoV-2 entry receptor ACE2, as the presence of ACE2 on the cell surface allows uptake of tryptophan via the transporter B0AT1 (13). In severe courses of influenza, as in chronic inflammation, increased tryptophan degradation and thus increased kynurenine levels are observed, and inhibition of tryptophan degradation has beneficial effects in animal models (18, 19). In turn, there is evidence in a wide variety of virus types that nicotinamide can reduce viral replication and support the body's defense mechanisms, *e.g.*, in the context of infections with vaccinia (20), human immunodeficiency virus (21), enteroviruses (21), or hepatitis B (22). Sufficient supply of B vitamins and particularly nicotinamide to strengthen the immune system is also recommended to combat SARS-CoV-2 infection (23-26). Here, the improvement of the defense against secondary bacterial infections in disease models is particularly emphasized (23). In the COVit trial, 1,000 mg of nicotinamide are administered, which is far below potentially harmful doses of several grams per day and very close to the acceptable daily intake of 900 mg/day (27, 28). Meanwhile, several publications suggest the use of nicotinamide in COVID-19 but there are no trial data available (17, 24-26, 29). In the pilot phase of the COVit trial, patients received 1,000 mg of conventional

nicotinamide or 245 mg of silica as an inert placebo-like dietary supplement. During a quality control of the data collection, it was surprisingly observed that significantly more patients in the nicotinamide group were already completely symptom-free after two weeks of administration than in the silica group. From the literature, depending on the patient population, very long convalescences can also be expected, e.g.

- 43% of patients were symptom-free at 14-21 days after positive SARS-CoV-2 test (30),
- <30% without respiratory symptoms after 1 month (31),
- 32% symptom-free at 30 days and still only 34% at 60 days after SARS-CoV-2 diagnosis (32), or
- only 12.6% symptom-free after a mean of 60 days (33).

Moreover, COVID-19 has been shown to be a systemic disease with an unexpectedly high frequency of gastrointestinal symptoms (34). It has been published that the gut microbiome can be significantly and, in some cases, negatively affected by COVID-19 (35). The link between the gut microbiome and the metabolism of tryptophan and nicotinamide plays an important role in intestinal inflammation (13-16). Therefore, two different tablets, each containing 500 mg nicotinamide, are now being used as investigational drugs in the main phase of the COVit trial: the conventional immediate-release nicotinamide tablets from the pilot phase and the newly developed controlled-ileocolonic-release nicotinamide (CICR-NAM) tablets, which release nicotinamide in a delayed and continuous manner starting in the lower small intestine. This should increase the gastrointestinal beneficial effects of nicotinamide, including those specifically on the microbiome and its interaction with the intestine, as conventional nicotinamide is absorbed very rapidly into the circulation (36). A study with a CICR-NAM prototype has shown that systemic exposure from this new dosage form will be lower than in the pilot phase of the study (36), in which exposure was already close to the acceptable daily intake anyway (see above). This will further reduce the already minimal risk of side effects.

2. Study Objectives

2.1. Primary Objective of the Main Study

The primary objective of the main study is to investigate the hypothesis that COVID-19 patients lose individual COVID-19 symptoms more quickly in an outpatient setting when supplemented with 1,000 mg of nicotinamide.

2.2. Secondary Objectives

The secondary objectives of the main study are to examine the following parameters: severity of individual symptoms, complete resolution of symptoms after 2, 4 or 6 weeks and the time from diagnosis to freedom from individual or all symptoms.

2.3. Exploratory Objectives

The WHO COVID-19 Ordinal Scale for Clinical Improvement (37) and the frequency of severe COVID-19 (examination in an emergency department, hospitalization with a continuous oxygen requirement of at least 24 hours, intensive care requirement, ventilation requirement or death by COVID-19) will be investigated in an exploratory fashion.

The findings from the pilot trial (COVit-1), in which only immediate-release nicotinamide was supplemented, and from the COVit-2 trial will be compared.

Changes in fatigue and quality of life will be assessed using validated questionnaires (FACIT-F and SF-36).

Further exploratory analyses on up to 20 selected patients will measure blood count, standard blood profile, various inflammatory markers and the metabolome in the blood, in particular tryptophan metabolism. In these patients, the virus strain will also be determined by sequencing from nasopharyngeal swabs. In selected patients, a detailed pharmacokinetic analysis can be performed. In the stool, changes in the microbiome (in 100-300 patients) as well as in the metagenome and metabolome (in a subgroup) will be analyzed.

3. Trial Methods

3.1. Trial Design

This is a double-blinded, randomized, placebo-controlled parallel group trial.

3.2. Randomization and Blinding

Patients are randomized in one of both treatment groups (nicotinamide or placebo) continuously as they are enrolled. The study is blinded for participants, care providers, investigators and outcome assessors as well as for the statisticians. The staff distributing the trial supplements is not blinded to ensure proper delivery. Trial supplements are provided in neutral containers. The blinding will not be violated by interim analyses as the allocation of randomization codes to treatment groups is not broken.

3.3. Sample Size

The data available at the beginning of the COVit-2 trial suggested that at most 40% of the already symptomatic COVID-19 patients would be completely symptom-free two weeks after the start of the intervention (primary endpoint defined at that time). This would likely have been the case in the placebo-treated patient population. The hypothesis was that this 40% event rate could be increased to 50% with the use of nicotinamide. Therefore, the following default values were assumed in the primary sample size calculation: α-error = 0.05; power = 0.8; the result is K = 7.85 (factor calculated from α-error and power).

The following simplified formula was used:

$$n = \frac{K\,[(R+1) - p_2\,(R^2+1)]}{p_2\,(1-R)^2}$$

n = required number of cases

p1 = event rate in the treatment group (50%)

p2 = event rate in the control group (40%)

R = relative risk (p1/p2) = here: 50% / 40% = 1.25

As a result, 385 patients were needed per group. With an assumed drop-out rate of close to 10%, 420 patients per group were to be included. Based on the results of the futility analysis after n=400 patients, the Data Management Board (DMB) of the COVit-2 trial recommended to keep the previously planned sample size of approximately 840 patients, to focus the primary endpoint on key symptoms (section 6.1.1.) and to select the subgroup of patients with at least one risk factor for severe COVID-19 as the primary clinical analysis population. To ensure that the subpopulation recruited after the futility analysis also included an adequate proportion of such patients, the frequencies of risk factors were monitored in a blinded manner and 900 patients were recruited as a safeguard against possibly increased dropout rates.

3.4. Framework

This clinical investigation evaluates the efficacy of a 4-week dietary intervention with a combination of 500 mg immediate-release nicotinamide and 500 mg controlled-ileocolonic release nicotinamide (CICR-NAM) on the recovery from COVID-19 symptoms both in a short-term (up 6 weeks) and a long-term setting (at approximately 6 months). The present SAP describes the analyses pertaining to the short-term setting.

3.5. Investigational Product

The trial uses four investigational products: conventional tablets that start to release nicotinamide immediately after reaching the stomach (immediate-release formulation) as well as novel tablets for CICR-NAM plus a similarly constructed matching placebo. The reasons for applying the innovative tablets for release in the ileum and colon are the gastrointestinal infections with SARS-CoV-2 and recent data showing dysbalances in the intestinal microbiome of COVID-19 patients. CICR-NAM was designed to beneficially influence the intestinal microbiome by topically increasing nicotinamide availability.

3.5.1. Nicotinamide Group

Participants assigned to the nicotinamide group self-administer 2 nicotinamide tablets (1 conventional 500-mg tablet and 1 500-mg tablet with CICR-NAM) for 4 weeks. The overall dose strength of the verum tablets is 1,000 mg nicotinamide, taken once daily with breakfast.

3.5.2. Control Group

Participants randomized to the control group self-administer 2 placebo tablets with an appearance corresponding to the respective nicotinamide tablets in the same manner as described above for the nicotinamide group.

3.6. Study Duration and Schedule

The study duration for the individual patient (see schedule below) comprises the 4-week active treatment period, a safety follow-up at 6 weeks and a recall at 6 months with characterization for remaining COVID-19 symptoms (post-COVID syndrome, PCS). Recruitment started on 01 February 2021 and the last patient was randomized on 17 January 2022. Week 6 observations were completed in March 2022. It is expected that the last data of the 6-month follow-up will be obtained in late July 2022. A planned futility analysis was performed in November 2021 after the 6-week data for 400 patients were available (see study protocol version 2.2 and SAP Futility Analysis, version 1.0).

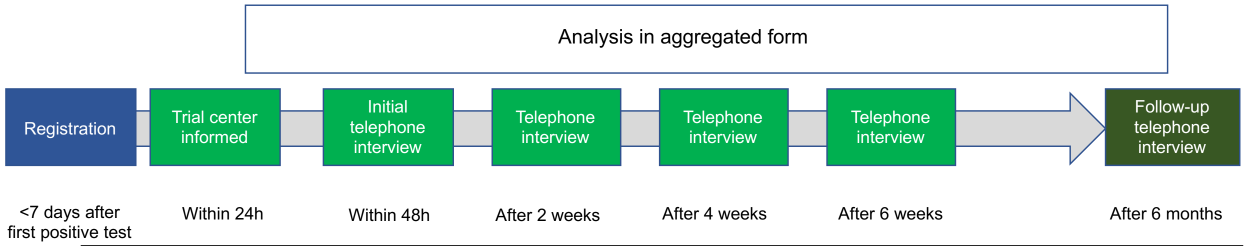

	Week 0	Week 2	Week 4	Week 6	....	Month 6
**Interviews**	x	x	x	x		x
**SF-36**	x	x	x	x		x
**FACIT-F**	x	x	x	x		x
**Daily symptom questionnaire**	x	x	x			

Figure 1: Scheme of visits during the trial. The present SAP describes the analyses until Week 6 (light blue box).

4. STATISTICAL PRINCIPLES

4.1. Confidence Intervals and P-Values

For all analyses, two-sided 95% confidence intervals (CI) will be reported. Confidence intervals provide an adequately plausible range for the true value related to the measurement of the point estimate. Statements are possible on the direction of the effects, as well as its strength and the presence of a statistically significant result. For (approximatively) normally distributed variables, parametric 95% CI will be shown. If this assumption is violated, the corresponding CI will be estimated by bootstrapping methods.

In contrast to confidence intervals, p-values give the difference from a previously specified statistical level α. P-values will be calculated in case of 1) stated hypotheses or 2) in an exploratory fashion to evaluate the difference from a statistical level α. P-values will be shown with three decimal places, whereby p-values less than .05 will be considered as statistically significant. If not otherwise stated, p-values are two-tailed.

4.2. Adherence and Protocol Deviations

The planned analysis will be performed according to the clinical investigation plan, its amendments, and this statistical analysis plan. If there are contradictions between the clinical investigation plan or its amendments and this statistical analysis plan, this analysis plan will prevail.

If a protocol deviation or concurrent illness / adverse event occurs, which, according to the clinical judgment of the Investigator, may invalidate the study by pharmacokinetic or pharmacodynamic interference with the trial products, the subject will be withdrawn by the Investigator.

4.3. Analysis Populations

The safety population will include all randomized subjects.

The intention-to-treat (**ITT**) population will include all randomized subjects who have received at least one dose of any study treatment. ITT has been carried forward for understanding the interaction between nicotinamide and immune reactions to SARS-CoV-2.

According to the recommendations of the DMB after the planned futility analysis, the primary analysis population for efficacy will be the **RFITT** population. The RFITT population will be defined as all subjects of the ITT population with a least one characteristic or underlying medical condition associated with an increased risk of developing severe illness from COVID-19, selected from the group consisting of

- an age of ≥60 years;
- a body mass index of ≥30.0;
- type 1 diabetes;
- type 2 diabetes;
- cardiovascular diseases;
- high blood pressure;
- stroke;
- asthma;
- chronic obstructive pulmonary disease;
- other chronic lung diseases;
- current or former smokers (the latter being defined as patients who smoked more than 100 cigarettes or other smoking products in total so far, but have not smoked for at least 4 weeks);
- chronic liver diseases;
- chronic kidney diseases;
- cancer;
- organ transplants;
- current immunosuppressive therapy;
- chronic neurological diseases (multiple sclerosis, Parkinson’s disease);

The **RFPP** population will be defined as all subjects of the RFITT population, excluding those patients who

- drop out or
- do not comply regarding investigational product intake for at least 80%, *i.e.* intake of investigational product for at least 11 of 14 days between each study interval: week 0 – week 2 and week 2 – week 4.

Futility analysis was performed after approximately 50% of patients had completed the Week 6 interview. All of these patients (n = 402) form a separate futility subgroup.

5. TRIAL POPULATION

5.1. Eligibility

5.1.1. Inclusion Criteria

- SARS-CoV-2 infection confirmed by laboratory findings; the positive test must not date back more than 7 days.
- Relevant symptoms of a SARS-CoV-2 infection, *e.g.* in the respiratory or gastrointestinal tract.
- The patient has been able to give written consent via a website before any trial procedure is performed and can comply with the trial-dependent prerequisites and requirements.
- The patient is 18 years or older.

5.1.2. Exclusion Criteria

Exclusion criterions are vaccination against SARS-CoV-2 before inclusion, current participation in another study and pregnancy or breast-feeding.

5.2. Recruitment

A flow diagram showing the progress through the phases of the trial (enrolment, intervention allocation, follow-up, and data analysis) will be produced for both treatment groups.

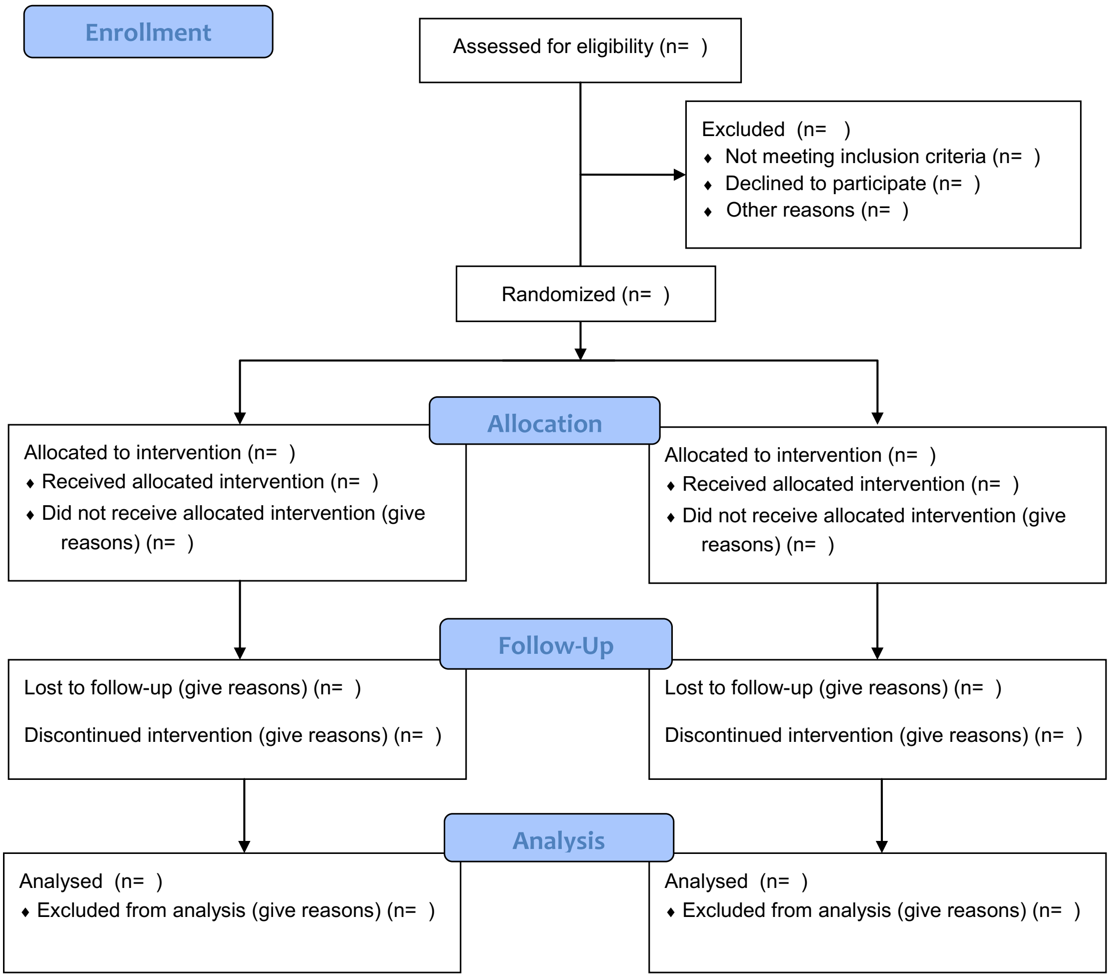

Figure 2: Flow chart according to CONSORT requirements.

5.3. Withdrawal/Follow-up

All subjects with evaluable data will be used for each analysis, irrespective of whether or not the subject subsequently dropped out.

The number of drop-outs and withdrawals will be reported as well as the time spent on the study (in days since enrolment) for those participants not completing the study protocol until the 6-weeks period.

6. ANALYSIS

6.1. Outcome Definition

6.1.1. Primary Outcomes

Frequencies of key individual COVID-19 symptoms at week 0, week 2 (primary time of analysis), week 4 and week 6:

1. Performance drop (yes/no).

For the futility population, additional key symptoms will be analyzed as part of the primary endpoint:

2. Ability to perform normal activities (quantified by the complaint scale for lower respiratory tract infections);
3. Cough (quantified by the complaint scale);
4. Fatigue (yes/no).

6.1.2. Secondary Outcomes

<u>Interviews:</u>

- Frequencies of individual COVID-19 symptoms (all symptoms recorded and not part of the primary endpoint) at week 0, week 2 (primary time of analysis), week 4 and week 6.

  In the primary analysis population RFITT, the key secondary outcomes *ability to perform normal activities (quantified by the complaint scale)* and *cough (quantified by the complaint scale)* in patients with severe complaints indicated by a complaint scale score of >3, respectively, as well as *fatigue (yes/no)* will be analyzed first and tested hierarchically. The complaint scale for lower respiratory tract infections has the following gradations: 0 = normal, 1 = very minor problem, 2 = minor problem, 3 = moderately poor, 4 = poor, 5 = very poor, 6 = maximally poor.
- Severity of all individual COVID-19 symptoms (all symptoms recorded and not part of the primary endpoint, including interviews and daily symptom questionnaire) at week 0, week 2 (primary time of analysis), week 4 and week 6 or on a daily basis, respectively.
- Frequency of being completely symptom-free after 2 weeks.
- Frequency of being completely symptom-free after 4 weeks.
- Frequency of being completely symptom-free after 6 weeks.
- Time from diagnosis to resolution of individual symptoms (in 2-week intervals, up to 6 weeks after the beginning of the dietary intervention).
- Time from diagnosis to complete resolution of symptoms (in days and in 2-week intervals, up to 6 weeks after the beginning of the dietary intervention).

<u>Daily symptom questionnaire:</u>

- Time from diagnosis to resolution of individual symptoms (in days, up to day 28 of the dietary intervention, *i.e.* up to approximately 35 days).

- Time from diagnosis to complete resolution of symptoms (in days, up to day 28 of the dietary intervention).

6.1.3. Exploratory Outcomes

Exploratory analyses will assess dose and time effects by using appropriate statistical models. Especially, analyses in the time course will adjust for the fact (by, e. g., mixed models or covariance pattern analyses) that measurements are repeatedly taken within the same subject. Various exploratory analyses are planned:

- World Health Organization (WHO) COVID-19 Ordinal Scale for Clinical Improvement (see https://www.who.int/publications/i/item/covid-19-therapeutic-trial-synopsis) at week 0 (baseline), week 2, week 4 and week 6.
- Severe COVID-19, defined as occurrence of one of the following characteristics:
  a. Examination in an emergency department;
  b. Hospitalization with a continuous oxygen requirement of at least 24 hours;
  c. Intensive care requirement;
  d. Ventilation requirement;
  e. Death by COVID-19.
- Duration of hospitalization in days.
- Comparison between the findings from the pilot trial (COVit-1), in which only immediate-release nicotinamide was supplemented, and from the COVit-2 trial.
- Change to Baseline of the 40-item Functional Assessment of Chronic Illness Therapy – Fatigue (FACIT-F) questionnaire and its subscales at week 2, week 4 and week 6.
- Change to Baseline of the SF-36 questionnaire (RAND 36-Item Health Survey 1.0) and its subscales at week 2, week 4 and week 6.
- Changes in blood levels of tryptophan (in selected patients).
- Changes in blood levels of tryptophan metabolites (in selected patients).
- Change in blood levels of inflammatory markers (C-reactive protein, interleukin-6, ferritin, neopterin, D-dimers) (in selected patients).
- Changes in blood count and standard blood profile (in selected patients).
- Changes in blood metabolome composition (in selected patients).
- Strain of SARS-CoV-2 virus (in selected patients).
- Changes in stool microbiome composition (in selected patients).

- Changes in stool metagenome composition (in selected patients).
- Changes in stool metabolome composition (in selected patients).
- Pharmacokinetics (in selected patients).

6.2. Baseline Patient Characteristics

Demographic characteristics will include

- Age
- Gender
- Height
- Weight
- Ethnicity

Demographic and Baseline characteristics will be summarized descriptively by treatment group for the ITT, RFITT and RFPP analysis set. Continuous variables will be summarized by number of subjects, mean (M), standard deviation (SD), median, minimum, and maximum values. Categorical variables will be summarized by number and percentage of subjects in each category. Comparability of treatment groups will be assessed based on the review of statistical summaries, such as mean/median or percentages in each category, among baseline characteristics and statistical testing (Chi-Square/Fisher Exact test for categorical variables, t-Test or Mann-Whitney-U test for metric variables).

6.3. Prior/Concomitant Medication and Supplementation

Counting rules for prior, concomitant, and post-treatment medications or dietary supplements: prior medication/supplementation refers to all medications/supplements that were taken prior to randomization. Concomitant medication/supplementation refers to all medications taken during the double-blind trial supplementation period (*i.e.*, the duration when the medication/supplementation is taken overlaps the double-blind trial supplementation period at any single time), including those continued from pre-treatment. Post-treatment medications/supplements include medications/supplements that were started after the end of the double-blind trial supplementation period.

7. ANALYSIS METHODS

7.1. Pre-Processing

7.1.1. Interview Data

Differences between week 2, week 4 and week 6 to baseline (week 0) will be calculated as: week 2 – week 0, week 4 – week 0 and week 6 – week 0, respectively. For ordinal variables (*e.g.*, symptoms quantified by the complaint scale), the difference is a metric variable. For binary variables [symptom present (yes/no)], the change to baseline will be divided into the categories worsening (symptom not present at week 0 but at week x – relevant for safety / ITT population), persistence (symptom present at baseline and still present at week x) or resolution (symptom present at baseline and absent at week x).

The event „symptom-free after x weeks“ will be calculated separately for all recorded symptoms. Patients who show no symptom at week x will be considered as symptom-free after x weeks, even if new onsets of symptoms are recorded at later time points. Subsequently occurring new symptoms may be due to causes other than acute COVID-19 and are therefore not taken into account.

Time from baseline to resolution of individual symptoms will be calculated as difference between visit date (week) at the event symptom-free and baseline visit (week 0) in weeks.

Time from baseline to complete resolution of symptoms will be calculated as difference between the date of symptom resolution (as stated by the patient in the following interview) and the baseline visit (week 0) in days. If the exact date could not be given by the patients, the week or month will be recorded. Following a worst case scenario, the last day of the week or month will be imputed to calculate the difference to baseline visit.

For patients who did not reach the status symptom-free within the trial period, the time from baseline to resolution of individual symptoms is calculated as difference between the follow-up visit to baseline (maximal observation time for patients with censored events).

7.1.2. Daily Symptom Questionnaire

The event „symptom-free after x days“ will be calculated separately for all recorded symptoms of the daily symptom questionnaire. Patients who show no symptom at day x will be considered as symptom-free after x days. Time from baseline to resolution of individual or all symptoms will be obtained directly from the diary.

7.1.3. FACIT-F

For the FACIT-F questionnaire, the subscales physical well-being, social/familiar well-being, emotional well-being, functional well-being, fatigue subscale, FACIT-F TOI, FACIT-G total score and FACIT-F total score will be calculated after data cleaning within an external Excel scoring sheet as described in the manual (39).

7.1.4. SF-36

The scoring of the RAND 36-Item Health Survey will be performed after double data entry and cleaning using an external scoring scheme in MS Excel. Scale and subscales will be calculated and sent for data analyses. Items that are left blank (missing data) are not taken into account when calculating the scale scores. Hence, scale scores represent the average for all items in the scale that the respondent answered. Values of the reference population are given in Table 1 (40).

Table 1: Reference values for SF-36 supplied by the RAND corporation based on the Medical Outcomes Study (n=2471).

Scale	Items	Alpha	Mean	SD
Physical functioning	10	0.93	70.61	27.42
Role functioning/physical	4	0.84	52.97	40.78
Role functioning/emotional	3	0.83	65.78	40.71
Energy/fatigue	4	0.86	52.15	22.39
Emotional well-being	5	0.90	70.38	21.97
Social functioning	2	0.85	78.77	25.43
Pain	2	0.78	70.77	25.46
General health	5	0.78	56.99	21.11
Health change	1	—	59.14	23.12

7.2. Main Analysis

The standard summary statistics for continuous baseline and outcome variables will be: N, mean, standard deviation, 95% CI, median, minimum and maximum. The standard summary statistics for categorical baseline and outcome variables will be absolute and relative frequencies (expressed as percentage).

Prior to analysis, all metric variables will be checked with respect of the normality assumption to draw reliable interpretations and conclusions of the research. Skewness and kurtosis will be used to assess the distribution form. If the absolute

values are less than 1, the deviation from the normal distribution will be considered safe (41).

Analyses of primary and secondary endpoints will be conducted on the ITT Analysis Set and for the RFITT and RFPP populations. The primary analysis population for efficacy will be RFITT. Change from baseline values (difference between measurements at week x and week 0) will be analyzed for all continuous efficacy variables at each time point collected.

The primary efficacy variable symptom (yes/no) will be analyzed using change to baseline, considered as worsening (symptom present at week x but not at baseline; relevant for safety / ITT population), persistence (no change) or resolution (symptom present at baseline but not at week x). The frequency of patients with symptom worsening, persistence or resolution for all weeks by treatment group will be compared using the Cochran-Mantel-Haenszel test. Post-hoc tests for each week will be calculated using Fisher Exact test with Benjamini & Hochberg (56) adjustment for multiple testing. The Woolfe test will be performed to test for homogeneity of odds ratios across time. In case of significant p-values of the Woolfe test, the Cochran-Mantel-Haenszel test would not be appropriate. In this case, Fisher Exact tests for each timepoint will be used instead of the Cochran-Mantel-Haenszel test.

For evaluation of the four ordered primary endpoints of the futility population, the unadjusted p-values from the Fisher Exact test of the change from baseline to week 2 between the treatment groups will be extracted. These p-values will be used for the hierarchical testing procedure using a fallback method.

The two metric/ordinal primary key symptoms “ability to perform normal activities” and “cough” (measured on a scale) will be analyzed using a mixed effect model repeat measurement (MMRM) approach with baseline value as a covariate, fixed effect terms in the model group, week, and group-by-week interaction, and an unstructured covariance matrix. Using this model, the difference in change from baseline between the groups will be estimated based on Least Square (LS) mean difference with corresponding 95% CI and p-value. In the event that the unstructured covariance matrix is non-estimable (due to lack of convergence) when fitting the MMRM to the observed study data, an autoregression of lag 1(AR(1)) covariance matrix will be used instead. For the comparison of selected visits, contrasts will be used. The treatment effect will be evaluated using the contrast treatment group (A vs. B) of change from week 2 to week 0 (baseline). Other contrasts for other time points will be also reported,

but not used for evaluation of the primary study endpoint. Hierarchical multiple testing with fallback procedure will be applied to the unadjusted contrast of treatment group (A vs. B) for the change from week 2 to week 0 (baseline) (see section 7.6).

Other symptoms recorded at the interviews will be analyzed by the same models in an exploratively manner only. Continuous or ordinal secondary endpoints (severity of symptoms) measured at different time points will be analyzed using the same MMRM approach as used for the metric primary efficacy variables. For categorical secondary endpoints [other symptoms (yes/no) recorded within the interviews], Cochran-Mantel-Haenszel and post-hoc Fisher Exact tests for change of binary variables (symptom worsening, persistence or resolution) will be applied.

For every symptom in the efficacy analyses in the RFITT population, only patients suffering from the respective symptom at Baseline will be included into the analysis. For continuous or ordinal endpoints (*e.g.*, severity of symptoms), further subgroups may be formed according to levels of intensity or severity (*e.g.,* patients with at least moderate symptoms or patients with severe symptoms). The first level of subgroup analysis will focus on patients with substantial symptomatology. For example, the key outcomes *ability to perform normal activities (quantified by the complaint scale)* and *cough (quantified by the complaint scale)* will first be analyzed in patients with severe complaints indicated by a complaint scale score of >3, respectively (see section 6.1.2.), and analyses of the FACIT-F and SF-36 questionnaires will focus on patients with baseline outcome (scales and subscales) below or equal to the corresponding median of the RFITT population.

For time-to-event analyses, a Kaplan-Meier approach will be used. Log rank test will be performed to test if the time to event differs between treatment groups. A Cox-proportional hazards Model will be used to evaluate and estimate the impact of the treatment group. Time-dependent covariates may be included if necessary. For interviews, time is recorded in 2-week intervals. In the symptom questionnaires, symptoms are recorded daily during the dietary supplementation of 28 days.

For the patient questionnaires (FACIT-F and SF-36), data reliability will be assessed through internal consistency using Cronbach's alpha coefficient ($\alpha \geq 0.80$) and item to total correlation of more than 0.20. The average variance extracted will be calculated to assess discriminant validity.

For analysis of the safety of the supplementation, all symptoms reported during the course of the study which were not present at baseline or increased in severity will be

MedDRA®-coded, listed by system organ class and preferred term, and their frequency will be compared between the two groups using Chi-square or Fisher Exact test.

All statistical tests will be 2-tailed with a type I error of .05 unless otherwise stated.

7.3. Subgroups

Subgroups analyses will be performed on demand in order to further define responders. Possible subgroups might be defined in accordance with the futility analysis:

Risk groups as subgroups of the RFITT populations:

- age of ≥60 years;
- body mass index of ≥30.0 or type 2 diabetes;
- cardiovascular diseases;
- high blood pressure;
- stroke;
- asthma, chronic obstructive pulmonary disease or other chronic lung diseases;
- current or former smokers (the latter being defined as patients who smoked more than 100 cigarettes or other smoking products in total so far, but have not smoked for at least 4 weeks)

Further subgroups might be defined based on biomarkers.

Within subgroup analyses also complementary subgroups will be analyzed.

Responders to primary and key secondary endpoints will be considered as additional population/subgroups.

Responders are defined as patients of ITT or RFITT or RFPP who show improvement from week 2 to week 6 compared to baseline in the primary and/or at least one of the secondary key endpoints. Improvement will be considered as resolution of symptom (binary endpoint) or reduction of severity of at least 3 scalepoints (scale).

Subgroup analyses will be performed using methods described in section 7.2.

7.4. Final Analyses and Reporting

The final analyses described in this SAP will be performed after the database has been cleaned and locked for the data up to week 6. The final report will include all analyses including up to week 6.

7.5. Missing Data

All effort will be made to achieve complete capture of all data from all patients, including those patients who discontinue treatment. Data of primary and secondary endpoints will be captured by interviews and the interviewers are instructed to contact patients multiple times until they receive feedback from them. The frequency of missing data will be checked with respect to treatment differences in advance.

Within the dataset, symptoms are reported only in case they are present and could be observed. So, all patients with no reported symptoms and thus missing values are treated as patients with no symptoms.

Statistical methods that do not employ imputation like MMRM or Chi-Square Test will be used for analyses. The use of the MMRM model assumes implicitly that data are missing at random (MAR).

7.6. Multiple Testing Procedure

For testing several symptoms, hierarchical testing with a fallback procedure will be applied. For the primary endpoints of the futility population, the type I error rate (α) is partitioned among four hypotheses of interest. Testing hypotheses proceeds in the following order:

1. Performance drop (yes/no);
2. Ability to perform normal activities (quantified by the complaint scale);
3. Cough (quantified by the complaint scale);
4. Fatigue (yes/no).

As long as hypotheses are rejected, the Type I error rate can be accumulated, making tests of later hypotheses more powerful than under the Bonferroni procedure. Unlike the fixed sequence test, the fallback test allows consideration of all hypotheses even if one or more hypotheses are not rejected early in the process, thereby avoiding a common concern about the fixed sequence procedure.

The Type I error rate (α) is divided in the following proportions:

1. Performance drop (yes/no): 60% (.03);
2. Ability to perform normal activities (quantified by the complaint scale): 15% (.0075);
3. Cough (quantified by the complaint scale): 15% (.0075);
4. Fatigue (yes/no): 10% (.005).

In the primary analysis population RFITT, sequential hierarchical testing (α level .05) is applied to the three key secondary endpoints:

1. Ability to perform normal activities (quantified by the complaint scale);
2. Cough (quantified by the complaint scale);
3. Fatigue (yes/no).

7.7. Statistical Software

R version 3.6.3 using RStudio Version 1.1.463 or a more recent version at the time of the analysis will be used for all statistical analyses.

8. Safety

In the COVit trial, 1,000 mg of nicotinamide are administered, which is far below potentially harmful doses of several grams per day and very close to the acceptable daily intake of 900 mg/day (see section 1). Moreover, half of the dose is administered in a formulation targeting the ileum and colon, which additionally reduces the pulse of systemic exposure from immediate-release (conventional) nicotinamide that was considered when defining the acceptable daily intake (see section 1). At higher doses than those used in the COVit trial, nicotinamide can cause heartburn, nausea, diarrhoea and other gastrointestinal symptoms. Cases of liver toxicity were usually observed only after longer exposure to at least 3,000 mg of systemically available nicotinamide per day. Adverse events and possible side effects can easily be monitored and will be recorded in the symptom questionnaire of the trial, which therefore also serves a pharmacovigilance-like purpose far exceeding the usual practice with the administration of dietary supplements.

In addition to the general surveillance of adverse events and safety aspects described in section 7.2., frequencies of symptoms recorded in the patient interviews will be specifically used to investigate possible side effects during the course of the study at week 2 to week 6 (if these were not present already at week 0). Therefore, all symptoms recorded in the patient interviews (queried as present/absent or via a scale of complaints, or noted as further symptoms) will be reported. Adverse events are defined as symptoms that were not present at baseline but were recorded at week 2 to 6 (binary variables and free text notation) or showed an increase in a scale of complaints of at least one point compared to baseline or to the previous visit during the course of the trial.

Adverse events will be MedDRA®-coded and reported in tabulated form including system organ class, preferred term, description of the adverse event/complaint, group

allocation, number and frequency (in %) of affected individuals as well as the total number of events. The Fisher Exact Test will be used to compare the frequency of adverse events between treatment groups.

9. REFERENCES

1. Nikolaus, S., Schulte, B., Al-Massad, N., Thieme, F., Schulte, D. M., Bethge, J., Rehman, A., Tran, F., Aden, K., Häsler, R., Moll, N., Schütze, G., Schwarz, M. J., Waetzig, G. H., Rosenstiel, P., Krawczak, M., Szymczak, S., & Schreiber, S. (2017). Increased Tryptophan Metabolism Is Associated With Activity of Inflammatory Bowel Diseases. Gastroenterology, 153(6), 1504–1516.e2https://doi.org/10.1053/j.gastro.2017.08.028
2. Agus, A., Planchais, J., & Sokol, H. (2018). Gut Microbiota Regulation of Tryptophan Metabolism in Health and Disease. Cell host & microbe, 23(6), 716–724. https://doi.org/10.1016/j.chom.2018.05.003
3. Sorgdrager, F., Naudé, P., Kema, I. P., Nollen, E. A., & Deyn, P. P. (2019). Tryptophan Metabolism in Inflammaging: From Biomarker to Therapeutic Target. Frontiers in immunology, 10, 2565. https://doi.org/10.3389/fimmu.2019.02565
4. Taleb S. (2019). Tryptophan Dietary Impacts Gut Barrier and Metabolic Diseases. Frontiers in immunology, 10, 2113. https://doi.org/10.3389/fimmu.2019.02113
5. Pongratz, G., Lowin, T., Sewerin, P., Zaucke, F., Jenei-Lanzl, Z., Pauly, T., Ostendorf, B., & Schneider, M. (2019). Tryptophan metabolism in rheumatoid arthritis is associated with rheumatoid factor and predicts joint pathology evaluated by the Rheumatoid Arthritis MRI Score (RAMRIS). Clinical and experimental rheumatology, 37(3), 450–457.
6. Heseler, K., Spekker, K., Schmidt, S. K., MacKenzie, C. R., & Däubener, W. (2008). Antimicrobial and immunoregulatory effects mediated by human lung cells: role of IFN-gamma-induced tryptophan degradation. FEMS immunology and medical microbiology, 52(2), 273–281. https://doi.org/10.1111/j.1574-695X.2007.00374.x
7. Suzuki, Y., Suda, T., Yokomura, K., Suzuki, M., Fujie, M., Furuhashi, K., Hahimoto, D., Enomto, N., Fujisawa, T., Nakamura, Y., Inui, N., Nakano, Y., Nakamura, H., & Chida, K. (2011). Serum activity of indoleamine 2,3-dioxygenase predicts prognosis of community-acquired pneumonia. The Journal of infection, 63(3), 215–222. https://doi.org/10.1016/j.jinf.2011.07.003
8. Meier, M. A., Ottiger, M., Vögeli, A., Steuer, C., Bernasconi, L., Thomann, R., Christ-Crain, M., Henzen, C., Hoess, C., Zimmerli, W., Huber, A., Mueller, B., & Schuetz, P. (2017). Activation of the tryptophan/serotonin pathway is associated with severity and predicts outcomes in pneumonia: results of a long-term cohort study. Clinical chemistry and laboratory medicine, 55(7), 1060–1069. https://doi.org/10.1515/cclm-2016-0912
9. Tsay, T. B., Chen, P. H., & Chen, L. W. (2019). Aryl hydrocarbon receptor ligands enhance lung immunity through intestinal IKKβ pathways. Journal of translational medicine, 17(1), 304. https://doi.org/10.1186/s12967-019-2043-8
10. Michaudel, C., Bataille, F., Maillet, I., Fauconnier, L., Colas, C., Sokol, H., Straube, M., Couturier-Maillard, A., Dumoutier, L., van Snick, J., Quesniaux, V. F., Togbe, D., & Ryffel,

B. (2020). Ozone-Induced Aryl Hydrocarbon Receptor Activation Controls Lung Inflammation via Interleukin-22 Modulation. Frontiers in immunology, 11, 144. https://doi.org/10.3389/fimmu.2020.00144

11. Takei, H., Yasuoka, H., Yoshimoto, K., & Takeuchi, T. (2020). Aryl hydrocarbon receptor signals attenuate lung fibrosis in the bleomycin-induced mouse model for pulmonary fibrosis through increase of regulatory T cells. Arthritis research & therapy, 22(1), 20. https://doi.org/10.1186/s13075-020-2112-7
12. Zhou, B., Lou, B., Liu, J., & She, J. (2020). Serum metabolite profiles as potential biochemical markers in young adults with community-acquired pneumonia cured by moxifloxacin therapy. Scientific reports, 10(1), 4436. https://doi.org/10.1038/s41598-020-61290-x
13. Hashimoto, T., Perlot, T., Rehman, A., Trichereau, J., Ishiguro, H., Paolino, M., Sigl, V., Hanada, T., Hanada, R., Lipinski, S., Wild, B., Camargo, S. M., Singer, D., Richter, A., Kuba, K., Fukamizu, A., Schreiber, S., Clevers, H., Verrey, F., Rosenstiel, P., … Penninger, J. M. (2012). ACE2 links amino acid malnutrition to microbial ecology and intestinal inflammation. Nature, 487(7408), 477–481. https://doi.org/10.1038/nature11228
14. Waetzig G, Seegert D. A pharmaceutical composition containing nicotinic acid and/or nicotinamide and/or tryptophan for positively influencing the intestinal microbiota. PCT patent application no. PCT/EP2013/062363. Published as. 2013;WO2013/186355 (19 December 2013).
15. Bettenworth, D., Nowacki, T. M., Ross, M., Kyme, P., Schwammbach, D., Kerstiens, L., Thoennissen, G. B., Bokemeyer, C., Hengst, K., Berdel, W. E., Heidemann, J., & Thoennissen, N. H. (2014). Nicotinamide treatment ameliorates the course of experimental colitis mediated by enhanced neutrophil-specific antibacterial clearance. Molecular nutrition & food research, 58(7), 1474–1490. https://doi.org/10.1002/mnfr.201300818
16. Wätzig G, Seegert D. A pharmaceutical composition containing combinations of nicotinamide and 5-aminosalicylic acid for beneficially influencing the intestinal microbiota and/or treating gastrointestinal inflammation. PCT patent application no. PCT/EP2014/077637. Published as. 2015;WO2015/086838 (18 June 2015).
17. Heer, C. D., Sanderson, D. J., Voth, L. S., Alhammad, Y., Schmidt, M. S., Trammell, S., Perlman, S., Cohen, M. S., Fehr, A. R., & Brenner, C. (2020). Coronavirus infection and PARP expression dysregulate the NAD metabolome: An actionable component of innate immunity. The Journal of biological chemistry, 295(52), 17986–17996. https://doi.org/10.1074/jbc.RA120.015138

18. Boergeling, Y., & Ludwig, S. (2017). Targeting a metabolic pathway to fight the flu. The FEBS journal, 284(2), 218–221. https://doi.org/10.1111/febs.13997
19. Pizzini, A., Kurz, K., Santifaller, J., Tschurtschenthaler, C., Theurl, I., Fuchs, D., Weiss, G., & Bellmann-Weiler, R. (2019). Assessment of neopterin and indoleamine 2,3-dioxygenase activity in patients with seasonal influenza: A pilot study. Influenza and other respiratory viruses, 13(6), 603–609. https://doi.org/10.1111/irv.12677
20. Child, S. J., Franke, C. A., & Hruby, D. E. (1988). Inhibition of vaccinia virus replication by nicotinamide: evidence for ADP-ribosylation of viral proteins. Virus research, 9(2-3), 119–132. https://doi.org/10.1016/0168-1702(88)90027-5
21. Murray M. F. (2003). Nicotinamide: an oral antimicrobial agent with activity against both Mycobacterium tuberculosis and human immunodeficiency virus. Clinical infectious diseases : an official publication of the Infectious Diseases Society of America, 36(4), 453–460. https://doi.org/10.1086/367544
22. Li, W. Y., Ren, J. H., Tao, N. N., Ran, L. K., Chen, X., Zhou, H. Z., Liu, B., Li, X. S., Huang, A. L., & Chen, J. (2016). The SIRT1 inhibitor, nicotinamide, inhibits hepatitis B virus replication in vitro and in vivo. Archives of virology, 161(3), 621–630. https://doi.org/10.1007/s00705-015-2712-8
23. Zhang, L., & Liu, Y. (2020). Potential interventions for novel coronavirus in China: A systematic review. Journal of medical virology, 92(5), 479–490. https://doi.org/10.1002/jmv.25707
24. Gharote MA. Role of poly (ADP) ribose polymerase-1 inhibition by nicotinamide as a possible additive treatment to modulate host immune response and prevention of cytokine storm in COVID-19. Ind J Med Sci. 2020;72(1):25-8.
25. Shi, Y., Wang, Y., Shao, C., Huang, J., Gan, J., Huang, X., Bucci, E., Piacentini, M., Ippolito, G., & Melino, G. (2020). COVID-19 infection: the perspectives on immune responses. Cell death and differentiation, 27(5), 1451–1454. https://doi.org/10.1038/s41418-020-0530-3
26. Shakoor, H., Feehan, J., Mikkelsen, K., Al Dhaheri, A. S., Ali, H. I., Platat, C., Ismail, L. C., Stojanovska, L., & Apostolopoulos, V. (2021). Be well: A potential role for vitamin B in COVID-19. Maturitas, 144, 108–111. https://doi.org/10.1016/j.maturitas.2020.08.007
27. EFSA. Scientific Opinion on Dietary Reference Values for niacin (EFSA Panel on Dietetic Products, Nutrition and Allergies). EFSA Journal 2014;12(7):3759.
28. OECD-SIDS. 3-pyridinecarboxamide (nicotinamide). SIDS Initial Assessment Report for SIAM 15, Boston, Massachusetts, 22-25 October 2002. Website:. 2002; https://www.inchem.org/documents/sids/sids/98920.pdf.

29. Mehmel, M., Jovanović, N., & Spitz, U. (2020). Nicotinamide Riboside-The Current State of Research and Therapeutic Uses. Nutrients, 12(6), 1616. https://doi.org/10.3390/nu12061616
30. Tenforde, M. W., Kim, S. S., Lindsell, C. J., Billig Rose, E., Shapiro, N. I., Files, D. C., Gibbs, K. W., Erickson, H. L., Steingrub, J. S., Smithline, H. A., Gong, M. N., Aboodi, M. S., Exline, M. C., Henning, D. J., Wilson, J. G., Khan, A., Qadir, N., Brown, S. M., Peltan, I. D., Rice, T. W., … IVY Network Investigators (2020). Symptom Duration and Risk Factors for Delayed Return to Usual Health Among Outpatients with COVID-19 in a Multistate Health Care Systems Network - United States, March-June 2020. MMWR. Morbidity and mortality weekly report, 69(30), 993–998. https://doi.org/10.15585/mmwr.mm6930e1
31. Marshall M. (2020). The lasting misery of coronavirus long-haulers. Nature, 585(7825), 339–341. https://doi.org/10.1038/d41586-020-02598-6
32. Carvalho-Schneider, C., Laurent, E., Lemaignen, A., Beaufils, E., Bourbao-Tournois, C., Laribi, S., Flament, T., Ferreira-Maldent, N., Bruyère, F., Stefic, K., Gaudy-Graffin, C., Grammatico-Guillon, L., & Bernard, L. (2021). Follow-up of adults with noncritical COVID-19 two months after symptom onset. Clinical microbiology and infection : the official publication of the European Society of Clinical Microbiology and Infectious Diseases, 27(2), 258–263. https://doi.org/10.1016/j.cmi.2020.09.052
33. Carfì, A., Bernabei, R., Landi, F., & Gemelli Against COVID-19 Post-Acute Care Study Group (2020). Persistent Symptoms in Patients After Acute COVID-19. JAMA, 324(6), 603–605. https://doi.org/10.1001/jama.2020.12603
34. Mitsuyama, K., Tsuruta, K., Takedatsu, H., Yoshioka, S., Morita, M., Niwa, M., & Matsumoto, S. (2020). Clinical Features and Pathogenic Mechanisms of Gastrointestinal Injury in COVID-19. Journal of clinical medicine, 9(11), 3630. https://doi.org/10.3390/jcm9113630
35. Yeoh, Y. K., Zuo, T., Lui, G. C., Zhang, F., Liu, Q., Li, A. Y., Chung, A. C., Cheung, C. P., Tso, E. Y., Fung, K. S., Chan, V., Ling, L., Joynt, G., Hui, D. S., Chow, K. M., Ng, S., Li, T. C., Ng, R. W., Yip, T. C., Wong, G. L., … Ng, S. C. (2021). Gut microbiota composition reflects disease severity and dysfunctional immune responses in patients with COVID-19. Gut, 70(4), 698–706. https://doi.org/10.1136/gutjnl-2020-323020
36. Fangmann, D., Theismann, E. M., Türk, K., Schulte, D. M., Relling, I., Hartmann, K., Keppler, J. K., Knipp, J. R., Rehman, A., Heinsen, F. A., Franke, A., Lenk, L., Freitag-Wolf, S., Appel, E., Gorb, S., Brenner, C., Seegert, D., Waetzig, G. H., Rosenstiel, P., Schreiber, S., … Laudes, M. (2018). Targeted Microbiome Intervention by Microencapsulated Delayed-Release Niacin Beneficially Affects Insulin Sensitivity in Humans. Diabetes care, 41(3), 398–405. https://doi.org/10.2337/dc17-1967

37. World Health Organization W. WHO R&D Blueprint: informal consultation on prioritization of candidate therapeutic agents for use in novel coronavirus 2019 infection, Geneva, Switzerland. 2020
38. Yellen, S. B., Cella, D. F., Webster, K., Blendowski, C., & Kaplan, E. (1997). Measuring fatigue and other anemia-related symptoms with the Functional Assessment of Cancer Therapy (FACT) measurement system. Journal of pain and symptom management, 13(2), 63–74. https://doi.org/10.1016/s0885-3924(96)00274-6
39. Alcantara, J., Whetten, A., Zabriskie, C., & Jones, S. (2021). Exploratory factor analysis of PROMIS-29 V1.0, PROMIS Global Health and the RAND SF-36 from chiropractic responders attending care in a practice-based research network. Health and quality of life outcomes, 19(1), 82. https://doi.org/10.1186/s12955-021-01725-9
40. Miles, J. & Shevlin, M. 2001. Applying Regression and Correlation. London: Sage
41. Benjamini, Y., and Hochberg, Y. (1995). Controlling the false discovery rate: a practical and powerful approach to multiple testing. Journal of the Royal Statistical Society Series B, 57, 289–300. https://www.jstor.org/stable/2346101.

Appendix A: Summary of Analyses

Variable	Outcome	Population [a]	Statistical Method	Type
Frequency of individual COVID-19 symptoms at week 2 (primary time point), week 4 and week 6 compared to baseline (week 0) – worsening, persistence and resolution	Categorical (worsening / persistence / resolution)	ITT	Cochran-Mantel-Haenszel test, post-hoc Fisher Exact test	Longitudinal
Frequency of individual COVID-19 symptoms at week 2 (primary time point), week 4 and week 6 compared to baseline (week 0) – persistence and resolution	Binary (persistence / resolution)	ITT, **RFITT**, RFPP	Cochran-Mantel-Haenszel test, post-hoc Fisher Exact test	Longitudinal
Change of the severity of individual COVID-19 symptoms from week 2 (primary time point), week 4 and week 6 to baseline (week 0)	Metric	ITT, **RFITT**, RFPP	MMRM	Longitudinal
Frequency of being completely symptom-free after 2 weeks	Binary (yes/no)	ITT, **RFITT**, RFPP	Chi-Square test or Fisher Exact test	Longitudinal
Frequency of being completely symptom-free after 4 weeks	Binary (yes/no)	ITT, **RFITT**, RFPP	Chi-Square test or Fisher Exact test	Longitudinal
Frequency of being completely symptom-free after 6 weeks	Binary (yes/no)	ITT, **RFITT**, RFPP	Chi-Square test or Fisher Exact test	Longitudinal
Time from diagnosis to resolution of individual symptoms (in 2-week intervals, up to 6 weeks)	Time to event	ITT, **RFITT**, RFPP	Kaplan-Meier, Cox-PH	Longitudinal
Time from diagnosis to complete resolution of symptoms (in 2-week intervals, up to 6 weeks)	Time to event	ITT, **RFITT**, RFPP	Kaplan-Meier, Cox-PH	Longitudinal
Time from diagnosis to resolution of individual symptoms (in days, up to day 28 of the dietary intervention) as reported in the daily symptom questionnaire	Time to event	ITT, **RFITT**, RFPP	Kaplan-Meier, Cox-PH	Longitudinal
Time from diagnosis to complete resolution of symptoms (in days, up to day 28 of the dietary intervention) as reported in the daily symptom questionnaire	Time to event	ITT, **RFITT**, RFPP	Kaplan-Meier, Cox-PH	Longitudinal
Time from diagnosis to complete resolution of symptoms (in days, up to 6 weeks) as reported in patient interviews	Time to event	ITT, **RFITT**, RFPP	Kaplan-Meier, Cox-PH	Longitudinal

Change of scale and subscales of FACIT-F from week 2 (primary time point), week 4 and week 6 to baseline (week 0)	Metric	ITT, **RFITT**, RFPP	MMRM	Longitudinal
Change of scale and subscales of SF-36 from week 2 (primary time point), week 4 and week 6 to baseline (week 0)	Metric	ITT, **RFITT**, RFPP	MMRM	Longitudinal

Annotation: a) Primary analysis population: RFITT

7. Statistical analysis plan for the 6-month follow-up

This section contains the statistical analysis plan for the analyses at the 6-month follow-up in the final version 1.0 of 24 October 2022 (47 pp.).

STATISTICAL ANALYSIS PLAN

Improvement of the nutritional status regarding nicotinamide (vitamin B3) and the disease course of COVID-19

COVit-2

Follow-up at 6 months after COVID-19

Type of study: Interventional (Clinical Trial)

Biometrician: Regina Hollweck (Novustat)

Version number: 1.0

Date of issue: 24 October 2022

Type of report: Follow-up at 6 months after COVID-19

SAP Version History Summary

SAP Version	Date	Change	Rationale
1	24 October 2022	Not applicable	Initial release

Signature

24.Oct 22 R. Hollweck

(Date) (Regina Hollweck, Trial Statistician)

27.10.2022 [illegible]

(Date) (Prof. Dr. Dr. h.c. Stefan Schreiber, Principal Investigator)

Table of Contents

1. Introduction 10
2. Study Objectives of the Follow-up Study 12
2.1. Primary Objective of the Follow-up Study 12
2.2. Secondary Objectives of the Follow-up Study 12
2.3. Exploratory Objectives of the Follow-up Study 12
3. Trial Methods 12
3.1. Trial Design 12
3.2. Randomization and Blinding 12
3.3. Sample Size 13
3.4. Framework 14
3.5. Investigational Product 14
3.5.1. Nicotinamide Group 14
3.5.2. Control Group 14
3.6. Study Duration and Schedule 14
4. Statistical Principles 15
4.1. Confidence Intervals and P-Values 15
4.2. Adherence and Protocol Deviations 16
4.3. Analysis Populations 16
5. Trial population 18
5.1. Eligibility 18
5.1.1. Inclusion Criteria 18
5.1.2. Exclusion Criteria 18
5.2. Recruitment 18
5.3. Withdrawal/Follow-up 19
6. Analysis 20
6.1. Outcome Definition 20
6.1.1. Primary Outcomes 20
6.1.2. Secondary Outcomes 20
6.1.3. Exploratory Outcomes 21
6.2. Baseline Patient Characteristics 21
7. Analysis Methods 21
7.1. Pre-Processing 21

7.1.1. PCS Score 21
7.1.2. Self-reported Smelling and Tasting Abilities 23
7.1.3. Self-reported Mini Olfactory Questionnaire (Self-MOQ) 28
7.1.4. Questionnaire of Olfactory Disorders (QOD) 28
7.1.5. Multidimensional Dyspnea Profile (MDP) 29
7.1.6. Patient Health Questionnaire Depression (PHQ-8) 30
7.1.7. Generalized Anxiety Disorder 7 (GAD-7) 30
7.1.8. Perceived Stress Scale (PSS) 31
7.1.9. Brief Resilience Scale (BRS) 31
7.1.10. Pittsburgh Sleep Quality Index (PSQI) 32
7.1.11. Multidimensional Fatigue Inventory (MFI) 33
7.1.12. University of Pennsylvania Smell Identification Test (UPSIT) 34
7.1.13. T3MS Cognitive Test 34
7.1.14. Level of antibodies against the N or S proteins of SARS-CoV-2 23
7.1.15. FACIT-F 34
7.1.16. SF-36 35
7.2. Main Analysis 37
7.3. Subgroups 40
7.4. Final Analyses and Reporting 40
7.5. Missing Data 41
7.6. Multiple Testing Procedure 41
7.7. Statistical Software 41
8. References 42
Appendix A: Summary of Analyses 45

List of Abbreviations

BRS .... Brief Resilience Scale
CI .... Confidence Interval
CICR-NAM .... Controlled Ileocolonic Release of Nicotinamide
COVID-19 .... Coronavirus Disease 2019
DMB .... Data Management Board
FACIT-F .... Functional Assessment of Chronic Illness Therapy – Fatigue
GAD-7 .... Generalized Anxiety Disorder 7
ITT .... Intention-to-Treat
LOESS .... Locally-weighted Scatterplot Smoothing
M .... Mean
MDP .... Multidimensional Dyspnoea Profile
MFI .... Multidimensional Fatigue Inventory
MMRM .... Mixed Model Repeated Measures
N .... Number of Observations
N Protein .... Nucleocapsid Protein
NAD^+ .... Nicotinamide adenine dinucleotide
PCS .... Post-COVID Syndrome
PHQ-8 .... Patient Health Questionnaire Depression
PSQI .... Pittsburgh Sleep Quality Index
PSS .... Perceived Stress Scale
QOD .... Questionnaire of Olfactory Disorders
RFITT .... Risk Factor Intention-to-Treat
RFPP .... Risk Factor per Protocol
S Protein .... Spike Protein
SAP .... Statistical Analysis Plan
SARS-CoV-2 .... Severe Acute Respiratory Syndrome Coronavirus Type 2
SD .... Standard Deviation
Self-MOQ .... Self-reported Mini Olfactory Questionnaire

SF-36 .... RAND 36-Item Health Survey

T3MS .... Telephone Adaptation of the Modified Mini-Mental State Exam

UPSIT .... University of Pennsylvania Smell Identification Test

Trial Summary

**Principal Investigator**	Prof. Dr. Dr. h.c. Stefan Schreiber and Prof. Dr. Matthias Laudes<br>Department of Internal Medicine I<br>University Medical Center Schleswig-Holstein (UKSH), Campus Kiel<br>Arnold-Heller-Str. 3<br>24105 Kiel<br>Telephone: +49 431 500-22201<br>Fax: +49 431 500-22204<br>E-mail: s.schreiber@mucosa.de
**Sponsor**	University Medical Center Schleswig-Holstein (UKSH), Campus Kiel<br>Arnold-Heller-Str. 3<br>24105 Kiel
**Title**	Improvement of the nutritional status regarding nicotinamide (vitamin B3) and the disease course of COVID-19
**Protocol Title**	Verbesserung des Ernährungsstatus bezüglich Nicotinamid (Vitamin B3) und Verlauf der COVID-19-Erkrankung
**Acronym**	COVit-2
**Background**	Nicotinamide, a form of vitamin B3, plays an important role in chronic systemic inflammation and reduced circulating levels impede immune defense against coronaviruses. Results from the pilot study COVit-1 and from the evaluation of the acute phase of the COVit-2 trial suggest that nicotinamide is efficacious against the acute drop in physical performance and other acute symptoms of coronavirus disease 2019 (COVID-19). In the follow-up study after 6 months, the effect of nicotinamide on symptoms of the post-COVID syndrome (PCS) – grouped in a weighted PCS Score or analysed individually –

	and on levels of antibodies directed against SARS-CoV-2 shall be evaluated.
**Study Design**	Prospective, double-blind, randomized, placebo-controlled clinical trial.
**Intervention Group**	Nicotinamide, 1,000 mg/day p.o. (one each of 500-mg immediate-release and delayed-release nicotinamide tablets), for 4 weeks.
**Control Group**	Placebo, p.o. (2 tablets), for 4 weeks.
**Randomization**	Patients are randomized continuously in a 1:1 ratio according to a randomization scheme in which every second patient is randomized and the following one then receives the opposite allocation.
**Blinding**	Quadruple (Participant, Care Provider, Investigator, Outcomes Assessor)
**Primary Analysis**	The primary efficacy variable PCS Score will be compared between the treatment groups using the t-Test or the non-parametric Mann-Whitney U Test. Categorization of PCS Score (none, mild, moderate and severe PCS) between treatment groups will be analyzed using the Fisher-Exact Test. Predictor analysis will be performed to identify predictors for PCS Score at baseline and to define subgroups of patients at risk for PCS. Treatment comparison for this PCS risk subgroup will be performed.
**Primary Endpoint**	Difference between patients treated with nicotinamide and placebo in the PCS Score.

1. Introduction

The present document aims at detailing the statistical analyses that will be performed for the follow-up after 6 months of the COVit trial (DRKS00021214, NCT04751604) on the basis of the current study protocol (Version 2.3, dated 13 Dec 2021). This document has been written by the lead statistician and reviewed and agreed by the Principal Investigator of the trial.

In the third year of the SARS-CoV-2 pandemic, COVID-19 still is a large global disease burden resulting in a significant loss in work productivity by both acute and long COVID-19.

Increased catabolism of tryptophan with high levels of kynurenine and other metabolites is a hallmark of acute inflammation upon infection with SARS-CoV-2 (1-3). Degradation of tryptophan results from increased activity of tryptophan-catabolizing enzymes such as indoleamine 2,3-dioxygenase-1 and is associated with disease severity in COVID-19 (1-3), but has also been observed in other infectious diseases including community-acquired bacterial pneumonia (4) and viral infections (5-7). Interestingly, tryptophan absorption also depends on the presence of angiotensin-converting enzyme-2 on the intestinal epithelium, which is also the entry point for SARS-CoV-2 (2, 8). We have previously shown that tryptophan is important to maintain a non-inflammatory homeostasis of the gut microbiome (9) and that supplementation with the tryptophan metabolite nicotinamide conveys strong anti-inflammatory effects that – in animal models – can be transferred with the gut microbiome (9-11). The anti-inflammatory effect through gut-targeted delivery was larger than by systemic supplementation (10).

Infection with SARS-CoV-2 leads to changes of the gut microbiome (12), which are assumed to contribute to COVID-19 outcome, *e.g.* by licensing of immune responses via microbe-derived metabolites (13, 14). Since impaired tryptophan co-metabolism affects host-microbe homeostasis in COVID-19 patients, topical administration of nicotinamide as a key anti-inflammatory molecule of the pathway should have substantial impact on the COVID-19 microbiome and may serve to improve the course of SARS-CoV-2 infections. Nicotinamide is required for generation of NAD^+, a coenzyme central to cellular energy metabolism. The availability of NAD^+ is compromised in infections in general and COVID-19 in particular (15-18). An observational, small open label study has suggested

that COVID-19-related acute kidney injury may be reduced by a 7-day course with 1 g/d nicotinamide (19).

We therefore developed the pharmaceutical pH-dependent matrix tablet formulation CICR-NAM with ingredients approved for use in both food and pharmaceuticals that releases nicotinamide in the lower small intestine and colon to maximize effects on the gut microbiome (NCT05258474).

Following a pilot experiment (COVit-1; DRKS00021214), the prospective, double-blind, randomized, placebo-controlled trial COVit-2 targeted an outpatient population within 7 days after testing PCR-positive for SARS-CoV-2 infection and investigated the effects of one conventional 500-mg nicotinamide tablet plus one 500 mg CICR-NAM tablet administered daily for 4 weeks. Results from COVit-1 and from the evaluation of the acute phase of the COVit-2 trial (until week 6) suggest that nicotinamide is efficacious against the acute drop in physical performance and other acute symptoms of COVID-19.

Reduced organ functions after recovery from acute COVID-19 may persist for weeks to months after the acute phase of the infection in patients following both mild and severe disease courses. There are different terms for this syndrome, such as PCS, post-acute COVID syndrome, post-acute sequelae of SARS-CoV-2 infection or “long COVID”. In the present SAP, the term PCS is used. A large meta-analysis showed that more than half of COVID-19 survivors experienced PCS 6 months after recovery (20). Key symptoms of PCS are fatigue (44% of patients), sleep disorder (33%), dyspnoea (40%), cough (22%) as well as anxiety (34%), depression (32%) and cognitive impairments (15%), and decreased quality of life was reported by 57% of PCS patients (21). Other meta-analyses found that the most frequent persisting symptoms of COVID-19 were fatigue, dyspnoea, exercise intolerance, impaired sense of smell and taste, cognitive impairments, headache, and general pain (22). A recent large trial from Germany reported that “neurological ailments (61.5%), fatigue (57.1%), and sleep disturbance (57.0%) were the most frequent persisting symptoms at 6−12 months after infection” and developed a weighted PCS Score (22).

In the follow-up study of the COVit-2 trial after 6 months, the effect of nicotinamide on symptoms of PCS – as determined by the PCS Score (22) or analysed individually – and on levels of antibodies directed against SARS-CoV-2 shall be evaluated.

2. Study Objectives of the Follow-up Study

2.1. Primary Objective of the Follow-up Study

The primary objective of the follow-up study is to investigate the hypothesis that COVID-19 patients show a reduced intensity of PCS, as determined by the PCS Score (22), in an outpatient setting when supplemented with 1,000 mg of nicotinamide.

2.2. Secondary Objectives of the Follow-up Study

The secondary objectives of the follow-up study are to examine the following parameters: levels of antibodies against the nucleocapsid (N) protein or spike (S) protein of SARS-CoV-2 as well as the occurrence and severity of symptoms characteristic of COVID-19 and/or PCS at the 6-month follow-up, as queried in telephone interviews using the same questions as for the acute phase of COVID-19.

Self-reported Smelling and Tasting Abilities, self-reported Mini-Olfactory Questionnaire (Self-MOQ), Questionnaire of Olfactory Disorders (QOD), Multidimensional Dyspnoea Profile (MDP), Patient Health Questionnaire – Depression (PHQ-8), Generalized Anxiety Disorder 7 (GAD-7), Perceived Stress Scale (PSS), Brief Resilience Scale (BRS), Pittsburgh Sleep Quality Index (PSQI), Multidimensional Fatigue Inventory (MFI), University of Pennsylvania Smell Identification Test (UPSIT) and the T3MS cognitive test will be performed at Month 6 cross-sectionally to investigate different aspects of PCS.

2.3. Exploratory Objectives of the Follow-up Study

Changes in fatigue and quality of life will be exploratively assessed using the questionnaires FACIT-F and SF-36.

3. Trial Methods

3.1. Trial Design

This is a double-blinded, randomized, placebo-controlled parallel group trial.

3.2. Randomization and Blinding

Patients are randomized in one of both treatment groups (nicotinamide or placebo) continuously as they are enrolled. The study is blinded for participants, care providers, investigators and outcome assessors as well as for the statisticians. The staff distributing

the trial supplements is not blinded to ensure proper delivery. Trial supplements are provided in neutral containers. The blinding will not be violated by interim analyses as the allocation of randomization codes to treatment groups is not broken.

3.3. Sample Size

The data available at the beginning of the COVit-2 trial suggested that at most 40% of the already symptomatic COVID-19 patients would be completely symptom-free two weeks after the start of the intervention (primary endpoint defined at that time). This would likely have been the case in the placebo-treated patient population. The hypothesis was that this 40% event rate could be increased to 50% with the use of nicotinamide. Therefore, the following default values were assumed in the primary sample size calculation: α-error = 0.05; power = 0.8; the result is K = 7.85 (factor calculated from α-error and power). The following simplified formula was used:

$$n = \frac{K\,[(R+1) - p_2\,(R^2+1)]}{p_2\,(1-R)^2}$$

n = required number of cases

p1 = event rate in the treatment group (50%)

p2 = event rate in the control group (40%)

R = relative risk (p1/p2) = here: 50% / 40% = 1.25

As a result, 385 patients were needed per group. With an assumed drop-out rate of close to 10%, 420 patients per group were to be included. Based on the results of the futility analysis after n=400 patients, the Data Management Board (DMB) of the COVit-2 trial recommended to keep the previously planned sample size of approximately 840 patients and to select the subgroup of patients with at least one risk factor for severe COVID-19 as the primary clinical analysis population. To ensure that the subpopulation recruited after the futility analysis also included an adequate proportion of such patients, the frequencies of risk factors were monitored in a blinded manner and 900 patients were recruited as a safeguard against possibly increased dropout rates. All recruited patients were contacted 6 months after study inclusion.

3.4. Framework

This clinical investigation evaluates the efficacy of a 4-week dietary intervention with a combination of 500 mg immediate-release nicotinamide and 500 mg controlled-ileocolonic release nicotinamide (CICR-NAM) on the recovery from COVID-19 symptoms both in a short-term (up 6 weeks) and a long-term setting (at approximately 6 months). The present SAP describes the analyses pertaining to the long-term setting.

3.5. Investigational Product

The trial uses four investigational products: conventional tablets that start to release nicotinamide immediately after reaching the stomach (immediate-release formulation) as well as novel tablets for CICR-NAM plus a similarly constructed matching placebo. The reasons for applying the innovative tablets for release in the ileum and colon are the gastrointestinal infections with SARS-CoV-2 and recent data showing dysbalances in the intestinal microbiome of COVID-19 patients. CICR-NAM was designed to beneficially influence the intestinal microbiome by topically increasing nicotinamide availability.

3.5.1. Nicotinamide Group

Participants assigned to the nicotinamide group self-administer 2 nicotinamide tablets (1 conventional 500-mg tablet and 1 500-mg tablet with CICR-NAM) for 4 weeks. The overall dose strength of the verum tablets is 1,000 mg nicotinamide, taken once daily with breakfast.

3.5.2. Control Group

Participants randomized to the control group self-administer 2 matching placebo tablets with an appearance corresponding to the respective nicotinamide tablets in the same manner as described above for the nicotinamide group.

3.6. Study Duration and Schedule

The study duration for the individual patient (see schedule below) comprises the 4-week active treatment period, a safety follow-up at 6 weeks and a recall at 6 months with characterization for remaining COVID-19 symptoms (PCS). Recruitment started on 01 February 2021 and the last patient was randomized on 17 January 2022. Week 6 observations were complete in March 2022. After the last 6-month follow-up interview

data had been obtained on 27 July 2022, the last cleaned data from digitalized and double-checked paper questionnaires became available for analysis on 20 October 2022.

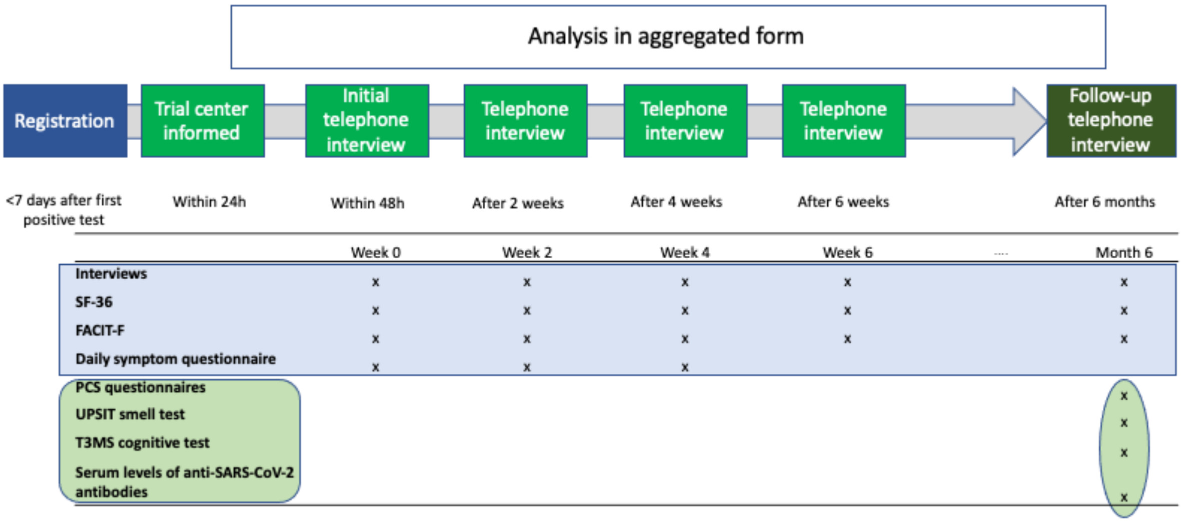

Figure 1: Scheme of visits during the trial.

4. STATISTICAL PRINCIPLES

4.1. Confidence Intervals and P-Values

For all analyses, two-sided 95% confidence intervals (CI) will be reported. Confidence intervals provide an adequately plausible range for the true value related to the measurement of the point estimate. Statements are possible on the direction of the effects, as well as their strength and the presence of a statistically significant result. For (approximatively) normally distributed variables, parametric 95% CI will be shown. If this assumption is violated, the corresponding CI will be estimated by bootstrapping methods.

In contrast to confidence intervals, p-values give the difference from a previously specified statistical level α. P-values will be calculated in case of 1) stated hypotheses or 2) in an exploratory fashion to evaluate the difference from a statistical level α. P-values will be shown with three decimal places, whereby p-values less than .05 will be considered as statistically significant. If not otherwise stated, p-values are two-tailed.

4.2. Adherence and Protocol Deviations

The planned analysis will be performed according to the clinical investigation plan, its amendments, and this SAP. If there are contradictions between the clinical investigation plan or its amendments and this SAP, this analysis plan will prevail.

If a protocol deviation or concurrent illness / adverse event occurs, which, according to the clinical judgment of the Investigator, may invalidate the study by pharmacokinetic or pharmacodynamic interference with the trial products, the subject will be withdrawn by the Investigator.

4.3. Analysis Populations

The safety population including all randomized patients has been addressed in the separate SAP for the final analyses of acute COVID-19 (baseline until week 6) dated 27 July 2022. In that SAP, the following three analysis populations were already defined.

The intention-to-treat (**ITT**) population includes all randomized subjects who have received at least one dose of any study treatment.

According to the recommendations of the DMB after the planned futility analysis, the primary analysis population for efficacy analysis in acute COVID-19 was the **RFITT** population. The RFITT population is defined as all subjects of the ITT population with a least one characteristic or underlying medical condition associated with an increased risk of developing severe illness from COVID-19, selected from the group consisting of

- an age of ≥60 years;
- a body mass index of ≥30.0;
- type 1 diabetes;
- type 2 diabetes;
- cardiovascular diseases;
- high blood pressure;
- stroke;
- asthma;
- chronic obstructive pulmonary disease;
- other chronic lung diseases;

- current or former smokers (the latter being defined as patients who smoked more than 100 cigarettes or other smoking products in total so far, but have not smoked for at least 4 weeks);
- chronic liver diseases;
- chronic kidney diseases;
- cancer;
- organ transplants;
- current immunosuppressive therapy;
- chronic neurological diseases (multiple sclerosis, Parkinson's disease);

The **RFPP** population (see separate SAP for the evaluation of the acute trial phase of COVit-2) is defined as all subjects of the RFITT population, excluding those patients who

- drop out or
- do not comply regarding investigational product intake for at least 80%, *i.e.* intake of investigational product for at least 11 of 14 days between the interventional study intervals (week 0 – week 2 and week 2 – week 4).

The ITT population will be the primary analysis population for efficacy in the 6-month follow-up described here, RFITT and RFPP will be secondary analysis populations.

At the follow-up after 6 months, the primary analysis will also focus on those patients who responded to nicotinamide or placebo treatment in any one of the primary and three key secondary endpoints during acute COVID-19 (until week 6) in order to investigate whether the acute treatment benefit also reduces the occurrence of PCS in these patients (Responder population).

Moreover, in order to identify patients at risk for developing PCS, baseline characteristics and symptoms that are significantly ($P<0.05$) correlated with the presence of PCS at the 6-month follow-up will be identified in the placebo group. Patients with at least 5 of these predictors will be included in PCS risk subgroups.

In all populations, subgroups of patients with a PCS score (22) of > 0 will be compared between nicotinamide- and placebo-treated patients in addition to the broader analysis of all patients including those with a PCS score of 0. Moreover, a subgroup of patients with a PCS score of ≥5 in the RFITT population will be analysed.

5. TRIAL POPULATION

5.1. Eligibility

5.1.1. Inclusion Criteria

- SARS-CoV-2 infection confirmed by laboratory findings; the positive test must not date back more than 7 days.
- Relevant symptoms of a SARS-CoV-2 infection, *e.g.* in the respiratory or gastrointestinal tract.
- The patient has been able to give written consent via a website before any trial procedure is performed and can comply with the trial-dependent prerequisites and requirements.
- The patient is 18 years or older.

5.1.2. Exclusion Criteria

Exclusion criterions are vaccination against SARS-CoV-2 before inclusion, current participation in another study and pregnancy or breast-feeding.

5.2. Recruitment

A flow diagram showing the progress through the phases of the trial (enrolment, intervention allocation, follow-up, and data analysis) will be produced for both treatment groups.

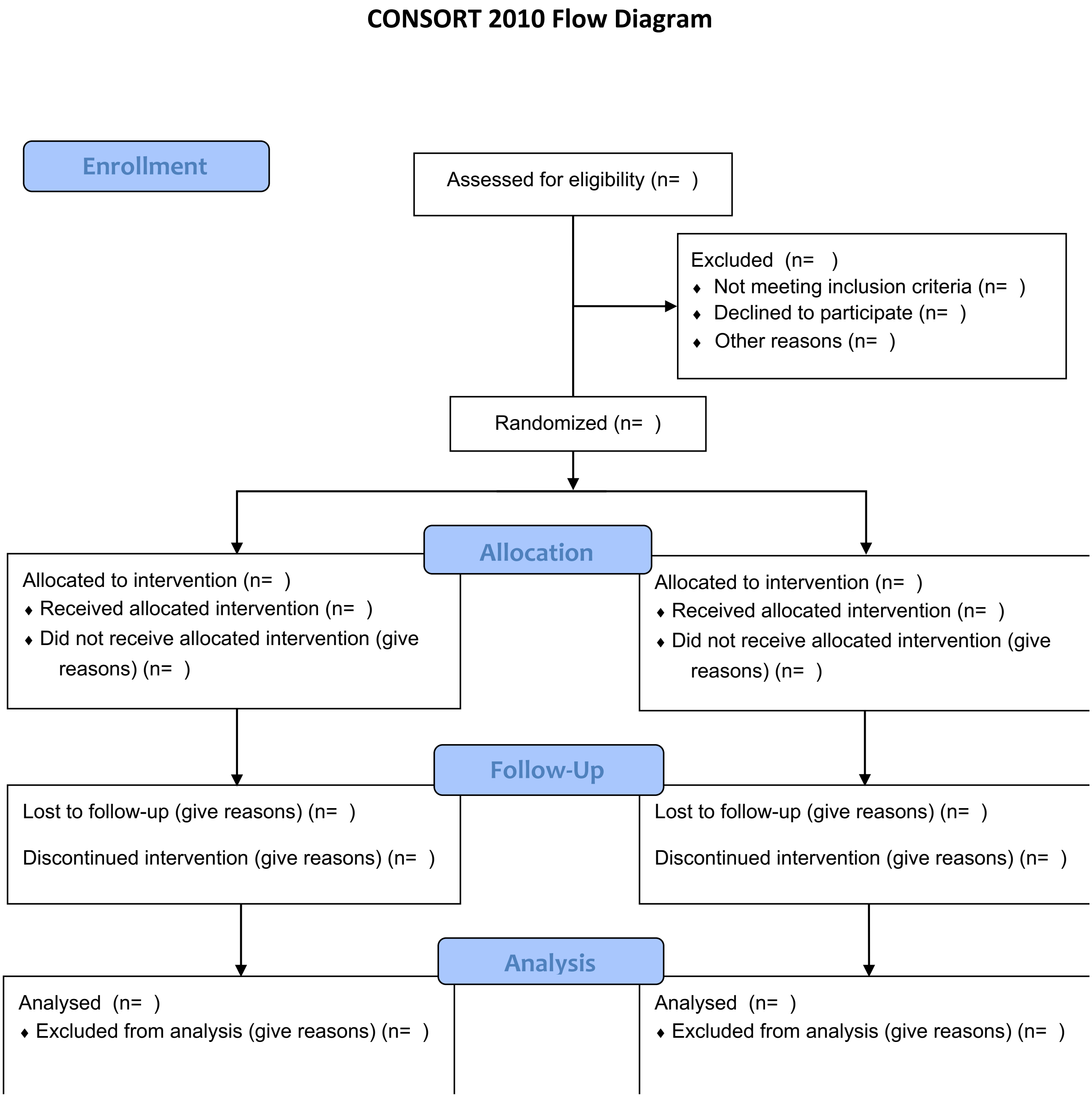

Figure 2 Flow Chart according to CONSORT Requirements.

5.3. Withdrawal/Follow-up

All subjects with evaluable data will be used for each analysis, irrespective of whether or not the subject subsequently dropped out.

The number of drop-outs and withdrawals will be reported as well as the time spent on the study (in days since enrolment) for those participants not completing the study protocol and full follow-up.

6. ANALYSIS

6.1. Outcome Definition

6.1.1. Primary Outcome at the 6-month follow-up

Occurrence and severity of symptoms of PCS measured by the PCS Score (22) between patients treated with placebo and nicotinamide.

6.1.2. Secondary Outcomes at the 6-month follow-up

Anti-SARS-CoV-2 antibodies:

The key secondary endpoint are the serum levels of antibodies directed against the N or S proteins of SARS-CoV-2.

Symptom occurrence and severity:

Symptoms characteristic of COVID-19 and/or PCS, as queried in telephone interviews at the 6-month follow-up using the same questions as for the acute phase of COVID-19.

Frequencies and severity of symptoms that are characteristic for PCS:

overall scale and subscales of

- Self-reported smelling and tasting abilities;
- Self-reported Mini Olfactory Questionnaire (Self-MOQ);
- Questionnaire of Olfactory Disorders (QOD);
- Multidimensional Dyspnoea Profile (MDP);
- Patient Health Questionnaire – Depression (PHQ-8);
- Generalized Anxiety Disorder 7 (GAD-7);
- Perceived Stress Scale (PSS);
- Brief Resilience Scale (BRS);
- Pittsburgh Sleep Quality Index (PSQI);
- Multidimensional Fatigue Inventory (MFI).

University of Pennsylvania Smell Identification Test (UPSIT): overall scale and subscales.

T3MS cognitive test: overall scale and subscales.

6.1.3. Exploratory Outcomes at the 6-month follow-up

Changes in fatigue and quality of life will be exploratively assessed using the following questionnaires:

- Description of the 40-item Functional Assessment of Chronic Illness Therapy – Fatigue (FACIT-F) questionnaire and its subscales;
- Description of the SF-36 questionnaire (RAND 36-Item Health Survey 1.0) and its subscales.

6.2. Baseline Patient Characteristics

Demographic characteristics of patients available at the follow-up phase will include

- Age;
- Gender;
- BMI (from height and weight);
- Ethnicity;
- Risk factors for severe COVID-19, as described in Section 4.3.

Demographic and baseline characteristics will be summarized descriptively by treatment group for the ITT, RFITT and RFPP analysis set. Continuous variables will be summarized by number of subjects, mean (M), standard deviation (SD), median, minimum, and maximum values. Categorical variables will be summarized by number and percentage of subjects in each category. Comparability of treatment groups will be assessed based on the review of statistical summaries, such as mean/median or percentages in each category, among baseline characteristics and statistical testing (Chi-Square/Fisher Exact test for categorical variables, t-Test or Mann-Whitney-U test for metric variables).

7. ANALYSIS METHODS

7.1. Pre-Processing

7.1.1. PCS Score

The PCS Score will be calculated from patient interview data according to Bahmer *et al.* (22). Long-term symptom complexes underlying the PCS Score definition are shown in Table 1.

Table 1 PCS Score development and weight (see Bahmer et al. (22))

**Symptom complex**	**Self-reported sub-symptoms at month 6 (present(1)/absent(0))**	**Variable from the COVit-2 eCRF**	**PCS Score weight**
Chemosensory deficits	Smelling disturbance, impaired sense of taste	covterma24, covterma25	3.5
Fatigue	Fatigue, performance drop	covterma1, covterma2	7
Exercise intolerance	Shortness of breath, reduced exercise capacity	covbreath >3, covterma5	4
Joint or muscle pain	Muscle pain, joint pain	covterma13, covterm14, covterma15	6.5
Ear-Nose-Throat (ENT ailments)	Hoarseness, sore throat, running nose	covterma9, covterma11, covterma12	5.5
Coughing, wheezing	Coughing, wheezing	covterma6, covterma7	7
Chest pain	Chest pain	covterma16	3.5
Gastrointestinal ailments	Stomach pain, diarrhoea, vomiting, nausea	covterma18, covterma19, covterma20, covterma21	5
Neurological ailments	Confusion, vertigo, headache, motor deficits, sensory deficits, numbness, tremor, deficits of concentration, cognition or speech	covterma17, covterma26, covterma27, covterma31	6.5
Dermatological ailments	Hair loss, rash, itchiness	covterma29, covterma30	2

Symptom complex	Self-reported sub-symptoms at month 6 (present(1)/absent(0))	Variable from the COVit-2 eCRF	PCS Score weight
Infection signs	Chills, fever, general sickness/flu-like symptoms	covterma3, covterma4, covterma34, covfeel >3	3.5
Sleep disturbance	Insomnia, unrestful sleep	covterma32, covterma33, covsleep >3	5

For each symptom complex, the presence of at least one of the patient-reported corresponding symptoms will be coded as value 1 (present) or 0 (absent) on the patient level.

The PCS Score will be calculated as weighted sum of the presence or absence of each symptom complex on the patient level using the weight shown in Table 1. The PCS Score ranges from 0 – 59 with higher values indicating more severe PCS.

Patients will be divided according to the PCS Score into 'none/mild', 'moderate', and 'severe' cases using the thresholds for the PCS Score of 10.75 and 26.25, respectively (22).

7.1.2. Level of antibodies directed against the N or S proteins of SARS-CoV-2

Prior to analyses, the time between the positive SARS-CoV-2 PCR test and the date of blood sampling for antibody analysis as well as, if applicable, the time(s) between one or more subsequent vaccinations against SARS-CoV-2 and the sampling date will be calculated in days. Antibody levels are measured using the AProof Duo Test (Adversis Pharma, Leipzig, Germany). In this test, dried blood spot samples are rehydrated and analysed by ELISAs detecting antibodies against the N protein (23) or S protein (24) of SARS-CoV-2. Anti-N antibody levels are quantified as percent of the signal of a positive control. The detection is considered negative if below 20% of the positive control, borderline for 20–30% and positive above 30%. Anti-S antibody levels are quantified as

binding antibody units per milliliter (BAU/mL; according to WHO International Standard for anti-SARS-CoV-2 immunoglobulin [human] [NIBSC code 20/136]). The detection is considered negative if below 22 BAU/mL, borderline for 22–44 BAU/mL and positive above 44 BAU/mL.

7.1.3. Interview Data

Differences between month 6 to baseline (week 0) will be calculated as: month 6 – week 0. For ordinal variables (*e.g.*, symptoms quantified by the complaint scale), the difference is a metric variable. For binary variables [symptom present (yes/no)], the change to baseline will be divided into the categories worsening (symptom not present at week 0 but at month 6), persistence (symptom present at baseline and still present at month 6) or resolution (symptom present at baseline and absent at month 6).

7.1.4. Self-reported Smelling and Tasting Abilities

For the self-reported smelling and tasting abilities, no formal score will be calculated. Changes between the status before, during and after infection will be described by combining items for smelling and tasting. The corresponding data will be exported directly from the database.

Variable	Scale	Variable name	Min	Max	Direction
Self-reported smelling before COVID-19	Scale (ordered)	riech_vor_covid	0	10	Higher score means better smelling
Self-reported smelling during COVID-19	Scale (ordered)	riech_bei_covid	0	10	Higher score means better smelling
Self-reported smelling after COVID-19	Scale (ordered)	riech_nach_covid	0	10	Higher score means better smelling

Variable	Scale	Variable name	Min	Max	Direction
Smelling difference after to before COVID-19	Scale (ordered)	DiffRiechen	-10	10	Negative values indicate worsening, positive values improvement
Self-reported nasal breathing before COVID-19	Scale (ordered)	nasenatm_vor_covid	0	10	Higher score means better nasal breathing
Self-reported nasal breathing during COVID-19	Scale (ordered)	nasenatm_bei_covid	0	10	Higher score means better nasal breathing
Self-reported nasal breathing since COVID-19	Scale (ordered)	nasenatm_nach_covid	0	10	Higher score means better nasal breathing
Nasal breathing difference after to before COVID-19	Scale (ordered)	DiffNasenatm	-10	10	Negative values indicate worsening, positive values improvement

Variable	Scale	Variable name	Min	Max	Direction
Self-reported smelling since COVID-19	Categorical	riech_seit_covid	0	1	0: no change, 1: change in smelling ability, -1 unknown
Change in smelling	categorical	gerueche	1	3	1: less than before, 2: different from before, 3: same as before, -1 unknown
Self-reported tasting before COVID-19	Scale (ordered)	schmecken_vor_covid	0	10	Higher score means better tasting
Self-reported tasting during COVID-19	Scale (ordered)	schmecken_bei_covid	0	10	Higher score means better tasting
Self-reported tasting after COVID-19	Scale (ordered)	schmecken_nach_covid	0	10	Higher score means better tasting
Tasting difference after to before COVID-19	Scale (ordered)	DiffSchmecken	-10	10	Negative values indicate worsening, positive values improvement

Variable	Scale	Variable name	Min	Max	Direction
Self-reported tasting since COVID-19	Categorical	schmecken_seit_covid	0	1	0: no change, 1 change in tasting ability, -1 unknown
Sweet taste	binary	schmecken_suess	0	1	0: no change, 1: Change in taste
Duration of change in sweet taste (days)	Scale (ordered)	schmecken_suess1	0	1000	
Salt taste	binary	schmecken_salzig	0	1	0: no change, 1: Change in taste
Duration of change in salt taste (days)	Scale (ordered)	schmecken_salzig1	0	1000	
Acid taste	binary	schmecken_sauer	0	1	0: no change, 1: Change in taste
Duration of change in acid taste (days)	Scale (ordered)	schmecken_sauer1	0	1000	
Bitter taste	binary	schmecken_bitter	0	1	0: no change, 1: Change in taste
Duration of change in bitter taste (days)	Scale (ordered)	schmecken_bitter1	0	1000	

Variable	Scale	Variable name	Min	Max	Direction
Pungent taste	binary	schmecken_scharf	0	1	0: no change, 1: Change in taste
Duration of change in pungent taste (days)	Scale (ordered)	schmecken_scharf1	0	1000	

7.1.5. Self-reported Mini Olfactory Questionnaire (Self-MOQ)

Five items of the Self-MOQ total score are recorded (s_moq1 to s_moq5). The total Self-MOQ Score will be calculated by summing up the number of items answered with yes (25).

Variable	Scale	Variable name	Min	Max	Direction
Self-MOQ score	Scale (count)	Self_MOQ	0	5	Lower score means better olfactory function

7.1.6. Questionnaire of Olfactory Disorders (QOD)

Seven key items of the QOD are answered by the patients. Patients who report disturbance of the sense of smell could agree (3 points), rather agree (2 point), rather disagree (1 point) or disagree (0 points) with each statement. The points of the 7 variables (variable names: qod1, qod13, qod27, qod33, qod37, qod42 and qod49) will be summed up to the total score (26). The initial question qod (“I have a disturbed sense of smell”) with the possible answers *yes*, *no* or *unknown* serves as a filter variable. Only those patients with an impaired sense of smell according to this variable are eligible to fill out the QOD questionnaire.

Variable	Scale	Variable name	Min	Max	Direction
QOD score	Scale (count)	QOD	0	21	Higher score means more limitations

7.1.7. Multidimensional Dyspnoea Profile (MDP)

Elements of the MDP (27, 28) are recorded by the patients to assess overall breathing discomfort (A1), intensity of five sensory qualities (physical breathing effort, air hunger, tightness, mental breathing effort and hyperpnoea) and intensity of emotional responses associated with dyspnoea (i.e. anxious, depressed, angry, frustrated, afraid) (in scales from 0 to 10, with higher values representing more discomfort).

The scores of the domains will be calculated using the arithmetic mean of the corresponding items within each domain. The immediate global unpleasantness item (A1) is used as the leading component of the definition of dyspnoea as “a subjective experience of breathing discomfort” (28).

Variable	Scale	Variable name	Min	Max	Direction
A1: Breathing discomfort	ordinal	mdp_a1	0	10	Higher values indicate higher discomfort
SQ: Physical breathing effort<br>SQ: Air hunger<br>SQ: Tightness<br>SQ: Mental breathing effort<br>SQ: Hyperpnoea	ordinal	mdp_sq_1 to mdp_sq_5	0	10	Higher values indicate higher discomfort
A2: Depressed<br>A2: Anxious<br>A2: Angry	ordinal	mdp_a2_1 to mdp_a2_5	0	10	Higher values indicate higher discomfort

Variable	Scale	Variable name	Min	Max	Direction
A2: Frustrated<br>A2: Afraid					
Immediate Perception Domain: Mean (mdp_sq_1 to mdp_sq_5, mdp_a1)	metric	mdp_sq	0	10	Higher values indicate higher discomfort
Emotional Response Domain: Mean (mdp_a2_1 to mdp_a2_5)	metric	mdp_a2	0	10	Higher values indicate higher discomfort

7.1.8. Patient Health Questionnaire – Depression (PHQ-8)

The PHQ-8 total score (29) will be calculated within the database system.

Variable	Scale	Variable name	Min	Max	Direction
PHQ-8 score	Scale (Sum)	phq8_total_score	0	24	Higher values indicate higher depression
Limitation of activities	Categorical	phq8_1_8	0	3	Higher values indicate higher limitation

7.1.9. Generalized Anxiety Disorder 7 (GAD-7)

The GAD-7 total score (30) will be calculated within the database system.

Variable	Scale	Variable name	Min	Max	Direction
GAD-7 score	Scale (Sum)	gad7_total_score	0	21	Higher values indicate higher anxiety

7.1.10. Perceived Stress Scale (PSS)

The PSS total score (31) will be calculated within the database system.

Variable	Scale	Variable name	Min	Max	Direction
PSS total score	Scale(sum)	pss_gesamt	10	50	Higher values indicate higher stress level
PSS score self-efficacy	Scale(sum)	pss_skala_selbstw	4	20	Higher values indicate lower self-efficacy
PSS score helplessness	Scale(sum)	pss_skala_hilfl	6	30	Higher values indicate higher helplessness

7.1.11. Brief Resilience Scale (BRS)

The BRS consists of 6 items (32). Response options are coded on a 5-level Likert scale from 1 (strongly disagree) to 5 (strongly agree) or vice versa, depending on the question. The total score will be calculated before data analysis as arithmetic mean of the 6 items (named brs1 to brs6).

Variable	Scale	Variable name	Min	Max	Direction
BRS score	Scale (arithmetic mean)	BRS	0	5	Higher values indicate better resilience

7.1.12. Pittsburgh Sleep Quality Index (PSQI)

The scale and four subscales of PSQI (sleep latency, sleep duration, sleep efficiency and sleep disturbance) will be calculated within the database system (33).

Variable	Scale	Variable name	Min	Max	Direction
PSQI score	Scale	psqi_total_score	0	21	Higher values indicate lower sleep quality
PSQI sleep latency	Scale	psqi_schlaflatenz	0	3	Higher values indicate higher sleep latency (more time)
PSQI sleep duration	Scale	psqi_schlafdauer	0	3	Higher values indicate less sleep
PSQI sleep efficiency (duration of sleep divided by duration of bed rest)	Scale	psqi_schlafeffizienz	0	3	Higher values indicate higher sleep efficacy

Variable	Scale	Variable name	Min	Max	Direction
PSQI sleep disturbance (sum of 9 reasons for sleep disturbance)	Scale	psqi_schlafstoer	0	3	Higher values indicate more sleep disturbance

7.1.13. Multidimensional Fatigue Inventory (MFI)

The scale and five subscales (general fatigue, physical fatigue, reduced activity, reduced motivation and mental fatigue) of the MFI will be calculated within the database system (34).

Variable	Scale	Variable name	Min	Max	Direction
MFI general fatigue score	Scale	mfi_general_fatigue	4	20	Low values indicate lower general fatigue
MFI physical fatigue score	Scale	mfi_physical_fatigue	4	20	Low values indicate lower physical fatigue
MFI reduced activity score	Scale	mfi_reduced_activity	4	20	Low values indicate higher physical activity
MFI reduced motivation score	Scale	mfi_reduced_motivation	4	20	Low values indicate higher motivation
MFI mental fatigue score	Scale	mfi_mental_fatigue	4	20	Low values indicate lower mental fatigue

Variable	Scale	Variable name	Min	Max	Direction
MFI total score	Scale	mfi_total_score	20	100	Low values indicate lower total fatigue

7.1.14. University of Pennsylvania Smell Identification Test (UPSIT)

The UPSIT Scale represents the number of correct identified smells with a maximum of 40 and a minimum of 0 (35). The scale will be calculated within the database system.

Variable	Scale	Variable name	Min	Max	Direction
Smell test score	Scale	Antwort Score.x	0	40	Higher values indicate higher smelling ability

7.1.15. T3MS Cognitive Test

The unadjusted scale of the T3MS (Telephone Adaptation of the Modified Mini-Mental State Exam) will be calculated within the database system (36, 37). Adjustments according to years of education may be made during further analyses, as appropriate.

Variable	Scale	Variable name	Min	Max	Direction
T3MS score	Scale	Antwort Score.y	0	100	Higher values indicate higher cognitive ability
3MS age adjusted score	Scale	Corrected Score	0	100	Higher values indicate higher cognitive ability

7.1.16. FACIT-F

For the FACIT-F questionnaire, the subscales physical well-being, social/familiar well-being, emotional well-being, functional well-being, fatigue subscale, FACIT-F Trial

Outcome Index (TOI), FACT-G total score and FACIT-F total score will be calculated after double data entry and cleaning within an external Microsoft Excel scoring sheet as described in the manual (38). Items that are left blank (missing data) are not taken into account when calculating the scale scores. Hence, scale scores represent the average for all items in the scale that the respondent answered. Analyses are performed in accordance with the separate SAP for the final analyses of acute COVID-19 (baseline until week 6) dated 27 July 2022.

Variable	Scale	Min	Max	Direction
Physical well-being	Scale(sum)	0	28	Higher values indicate greater well-being
Social/family well-being	Scale(sum)	0	28	Higher values indicate greater well-being
Emotional well-being	Scale(sum)	0	24	Higher values indicate greater well-being
Functional well-being	Scale(sum)	0	28	Higher values indicate greater well-being
Fatigue subscale	Scale(sum)	0	52	Higher values indicate greater well-being
FACIT-F TOI	Scale(sum)	0	108	Higher values indicate greater well-being
FACT-G total score	Scale(sum)	0	108	Higher values indicate greater well-being
FACIT-F total score	Scale(sum)	0	160	Higher values indicate greater well-being

7.1.17. SF-36

The scoring of the RAND 36-Item Health Survey 1.0 (https://www.rand.org/health-care/surveys_tools/mos/36-item-short-form/scoring.html) (39) will be performed after double data entry and cleaning using an external scoring scheme in Microsoft Excel.

Scale and subscales will be calculated and sent for data analyses. Items that are left blank (missing data) are not taken into account when calculating the scale scores. Hence, scale scores represent the average for all items in the scale that the respondent answered. Analyses are performed in accordance with the separate SAP for the final analyses of acute COVID-19 (baseline until week 6) dated 27 July 2022.

**Variable**	**Scale**	**Min**	**Max**	**Direction**
Physical functioning	Scale	0	100	Higher values indicate greater well-being
Role functioning/ physical	Scale	0	100	Higher values indicate greater well-being
Role functioning/ emotional	Scale	0	100	Higher values indicate greater well-being
Energy / fatigue	Scale	0	100	Higher values indicate greater well-being
Emotional well-being	Scale	0	100	Higher values indicate greater well-being
Social functioning	Scale	0	100	Higher values indicate greater well-being
Pain	Scale	0	100	Higher values indicate greater well-being
General health	Scale	0	100	Higher values indicate greater well-being
Health change	Scale	0	100	Higher values indicate greater well-being

7.2. Main Analysis

The standard summary statistics for continuous baseline and outcome variables will be: N, mean (M), standard deviation (SD), 95% CI, median, minimum and maximum. The standard summary statistics for categorical baseline and outcome variables will be absolute and relative frequencies (expressed as percentage).

Prior to analysis, all metric variables will be checked with respect of the normality assumption to draw reliable interpretations and conclusions of the research. Skewness and kurtosis are used to assess the distribution form. If the absolute values are less than 1, the deviation from the normal distribution is considered safe (40).

Analyses of primary and secondary endpoints will be conducted on the ITT population (primary analysis population, see section 4.3) as well as for the RFITT and RFPP populations. An overview of all analyses is provided in Appendix A.

Analyses of the PCS Score

The primary efficacy variable PCS Score will be compared between the treatment groups using the t-Test or the non-parametric Mann-Whitney U Test. Categorization of the PCS Score (none, mild, moderate and severe PCS) between treatment groups will be analyzed using the Fisher-Exact Test or the Chi-Square Test. All recorded symptoms at month 6 will be listed with absolute and relative frequencies and compared between the two treatment groups using the Fisher-Exact Test or the Chi-Square Test. In case that more than 20% of expected cell counts within the contingency tables are less than 5, the Fisher-Exact Test will be used instead of the Chi-Square Test. Responders of primary and key secondary endpoints within the first 6 weeks (acute COVID-19) will be considered as a subgroup (see section 4.3). The PCS formula will be applied to visits of the acute phase (week 0 to week 6) as a measure of overall COVID-19 symptom severity and will be analyzed descriptively using histograms. The PCS Score over time will be analyzed using a mixed model repeated measures (MMRM) approach with the baseline value as a covariate, fixed effect terms in the model group, week, and group-by-week interaction, and an unstructured covariance matrix. Using this model, the difference in change from baseline between the groups will be estimated based on least square mean difference with corresponding 95% CI and p-value. In the event that the unstructured covariance

matrix is non-estimable (due to lack of convergence) when fitting the MMRM to the observed study data, an autoregression of lag 1 covariance matrix will be used instead. For the comparison of selected visits, contrasts will be used. Individual courses of the PCS Score from baseline to month 6 will be drawn for patients with increased PCS at month 6. Tables with mean and SD will be shown and the t-Test or the non-parametric Mann-Whitney U Test will be applied to evaluate differences between treatment groups. The PCS Score in relation to time of recruitment will be analyzed exploratively to evaluate if severity of PCS is related to time of COVID-19 infection. Analyses will be performed in an exploratory fashion using scatterplots (time of recruitment vs. PCS score) and interpolation using smoothing procedures (locally-weighted scatterplot smoothing (LOESS)). Frequency and severity of PCS in patients with particularly mild progression (symptom-free after 2 weeks) compared to other patients will be performed using the t-Test or the non-parametric Mann-Whitney U Test.

Predictor analyses

Relevant predictors for PCS at month 6 will be obtained by screening baseline parameters (demographics, medical history, concomitant medication, risk factors for severe COVID-19 and symptoms present at baseline) as well as changes in the early stage of the disease (resolution/new onset/constant presence/constant absence of symptoms at week 2 compared to baseline). Screening will be applied within the placebo group comparing patients with a PCS score of ≥5 at month 6 with patients having a PCS score of <5. The t-test or the non-parametric Mann-Whitney-U test will be performed. Relevant predictors will be identified as parameters with p-values of <.05. Subgroups of patients at risk for developing an increased PCS Score will be defined using these relevant predictors. All patients belonging to PCR risk subgroups will be compared with regard to treatment group (nicotinamide or placebo) applying the t-test or the non-parametric Mann-Whitney-U test.

Comparison of symptoms to baseline

Change from baseline values will be analyzed for all symptoms (categorical and ordinal) at month 6. Comparison to baseline situation (symptom resolved at month 6, constant symptom present, symptom absent, symptom newly reported at month 6) will be

calculated and compared between treatment groups using the Fisher-Exact test or the Chi-Square test. For evaluation of symptoms measured on a scale of complaints, change to baseline between treatment groups will be analyzed using Mann-Whitney-U Test or t-test for independent samples.

Analyses of antibody levels

Analyses of antibodies directed against the N or S protein of SARS-CoV-2 will be performed using ANOVA. The level of antibodies against the N or S protein will be the dependent variables, group and number of vaccinations (none, one, multiple) will be independent factors. Post-hoc tests for vaccination will be performed using the Holm procedure to adjust for multiple testing. Correlation analysis showing the correlation coefficient, 95% CI and p-values will be applied to investigate the correlation of time difference between the positive test for SARS-CoV-2 (and/or) study inclusion (date of baseline visit at week 0) and the date of blood sampling for the antibody test. The time between sampling and baseline interview will be compared between the two treatment groups using the t-Test or the non-parametric Mann-Whitney U Test. Responders of primary and key secondary endpoints within the first 6 weeks (acute COVID-19) will be considered as a subgroup (see section 4.3). Correlation of the levels of antibodies against N or S protein and the PCS Score will be performed using the Pearson correlation coefficient or the Spearman correlation coefficient (in case of a violation of the normality assumption). Threshold values for negative, borderline or positive antibody detection as described in section 7.1.2 will be applied and treatment comparisons for these categorizations will be performed for all patients (ITT, RFITT, RFPP, Responder), as well as the subgroups of vaccinated and unvaccinated patients.

Analyses of questionnaires and tests

For the patient questionnaires (Self-MOQ, QOD, MDP, PHQ-8, GAD-7, PSS, BRS, PSQI, MFI, UPSIT, T3MS, FACIT-F and SF-36), the t-test or the Mann-Whitney-U test will be applied to test differences in total score or subscores between treatment groups.

Patients who reported impaired sense of smell or taste within the interviews will be compared with patients without impaired sense of smell or taste with respect to their

results in Self-MOQ, QOD, UPSIT and self-reported smelling/tasting using the t-test or the Mann-Whitney-U test.

All statistical tests will be 2-tailed with a type I error of .05 unless otherwise stated.

7.3. Subgroups

Subgroup analyses will be performed on demand in order to further define responders. Possible subgroups might be defined in accordance with the futility analysis and the analysis of acute COVID-19:

1) Risk groups as subgroups of the ITT, RFITT and RFPP populations:

- age of ≥60 years;
- body mass index of ≥30.0 or type 2 diabetes;
- cardiovascular diseases;
- high blood pressure;
- stroke;
- asthma, chronic obstructive pulmonary disease or other chronic lung diseases;
- current or former smokers (the latter being defined as patients who smoked more than 100 cigarettes or other smoking products in total so far, but have not smoked for at least 4 weeks).

2) Booster vaccination (yes/no, once/twice).

3) Responder in any one of the primary and key secondary endpoints during acute COVID-19 (until week 6; for details see the separate SAP for the final analyses of acute COVID-19 dated 27 July 2022).

4) Patients at risk for the development of PCS according to predictors identified in the placebo arm of the trial.

Subgroup analyses will be performed using the methods described in section 7.2.

7.4. Final Analyses and Reporting

The final analyses described in this SAP will be performed after the database has been cleaned and locked (database hard lock).

7.5. Missing Data

All efforts will be made to achieve complete capture of all data from all patients. Data of the primary outcome will be captured by interviews and the interviewers are instructed to contact patients multiple times until they receive feedback from them. Within the dataset, symptoms are reported only in case they are present and could be observed. So, all patients with no reported symptoms and thus missing values are treated as patients with no symptoms. Statistical methods that do not employ imputation like mixed model repeated measures (MMRM) or Chi-Square Test will be used for analyses. The use of the MMRM model assumes implicitly that data are missing at random.

7.6. Multiple Testing Procedure

To counteract the problem of multiple testing, the Bonferroni-Holm method will be applied where appropriate.

7.7. Statistical Software

R version 3.6.3 using RStudio Version 1.1.463 or a more recent version at the time of the analysis will be used for all statistical analyses.

8. REFERENCES

1. Anderson G, Carbone A, & Mazzoccoli G (2021) Tryptophan Metabolites and Aryl Hydrocarbon Receptor in Severe Acute Respiratory Syndrome, Coronavirus-2 (SARS-CoV-2) Pathophysiology. *Int J Mol Sci* 22(4):1597.
2. Sencio V, Machado MG, & Trottein F (2021) The lung-gut axis during viral respiratory infections: the impact of gut dysbiosis on secondary disease outcomes. *Mucosal Immunol* 14(2):296-304.
3. Chilosi M, Doglioni C, Ravaglia C, Martignoni G, Salvagno GL, Pizzolo G, Bronte V, & Poletti V (2022) Unbalanced IDO1/IDO2 Endothelial Expression and Skewed Keynurenine Pathway in the Pathogenesis of COVID-19 and Post-COVID-19 Pneumonia. *Biomedicines* 10(6):1332.
4. Meier MA, Ottiger M, Vogeli A, Steuer C, Bernasconi L, Thomann R, Christ-Crain M, Henzen C, Hoess C, Zimmerli W, Huber A, Mueller B, & Schuetz P (2017) Activation of the tryptophan/serotonin pathway is associated with severity and predicts outcomes in pneumonia: results of a long-term cohort study. *Clin Chem Lab Med* 55(7):1060-1069.
5. Mehraj V & Routy JP (2015) Tryptophan Catabolism in Chronic Viral Infections: Handling Uninvited Guests. *Int J Tryptophan Res* 8:41-48.
6. Wendt CH, Castro-Pearson S, Proper J, Pett S, Griffin TJ, Kan V, Carbone J, Koulouris N, Reilly C, Neaton JD, & Group IFPS (2021) Metabolite profiles associated with disease progression in influenza infection. *PLoS One* 16(4):e0247493.
7. Agus A, Planchais J, & Sokol H (2018) Gut Microbiota Regulation of Tryptophan Metabolism in Health and Disease. *Cell Host Microbe* 23(6):716-724.
8. Qin WH, Liu CL, Jiang YH, Hu B, Wang HY, & Fu J (2021) Gut ACE2 Expression, Tryptophan Deficiency, and Inflammatory Responses The Potential Connection That Should Not Be Ignored During SARS-CoV-2 Infection. *Cell Mol Gastroenterol Hepatol* 12(4):1514-1516 e1514.
9. Hashimoto T, Perlot T, Rehman A, Trichereau J, Ishiguro H, Paolino M, Sigl V, Hanada T, Hanada R, Lipinski S, Wild B, Camargo SM, Singer D, Richter A, Kuba K, Fukamizu A, Schreiber S, Clevers H, Verrey F, Rosenstiel P, & Penninger JM (2012) ACE2 links amino acid malnutrition to microbial ecology and intestinal inflammation. *Nature* 487(7408):477-481.
10. Waetzig G & Seegert D (2013) A pharmaceutical composition containing nicotinic acid and/or nicotinamide and/or tryptophan for positively influencing the intestinal microbiota. PCT patent application no. PCT/EP2013/062363. *Published as* WO2013/186355 (19 December 2013).
11. Wätzig G & Seegert D (2015) A pharmaceutical composition containing combinations of nicotinamide and 5-aminosalicylic acid for beneficially influencing the intestinal microbiota and/or treating gastrointestinal inflammation. PCT patent application no. PCT/EP2014/077637. *Published as* WO2015/086838 (18 June 2015).
12. Yeoh YK, Zuo T, Lui GC, Zhang F, Liu Q, Li AY, Chung AC, Cheung CP, Tso EY, Fung KS, Chan V, Ling L, Joynt G, Hui DS, Chow KM, Ng SSS, Li TC, Ng RW, Yip TC, Wong GL, Chan FK, Wong CK, Chan PK, & Ng SC (2021) Gut microbiota composition reflects disease severity and dysfunctional immune responses in patients with COVID-19. *Gut* 70(4):698-706.
13. Pickard JM, Zeng MY, Caruso R, & Nunez G (2017) Gut microbiota: Role in pathogen colonization, immune responses, and inflammatory disease. *Immunol Rev* 279(1):70-89.
14. Budden KF, Gellatly SL, Wood DL, Cooper MA, Morrison M, Hugenholtz P, & Hansbro PM (2017) Emerging pathogenic links between microbiota and the gut-lung axis. *Nat Rev Microbiol* 15(1):55-63.
15. Singhal A & Cheng CY (2019) Host NAD+ metabolism and infections: therapeutic implications. *Int Immunol* 31(2):59-67.
16. Tan A & Doig CL (2021) NAD(+) Degrading Enzymes, Evidence for Roles During Infection. *Front Mol Biosci* 8:697359.
17. Brenner C (2022) Viral infection as an NAD(+) battlefield. *Nat Metab* 4(1):2-3.
18. Zheng M, Schultz MB, & Sinclair DA (2022) NAD(+) in COVID-19 and viral infections. *Trends Immunol* 43(4):283-295.
19. Raines NH, Ganatra S, Nissaisorakarn P, Pandit A, Morales A, Asnani A, Sadrolashrafi M, Maheshwari R, Patel R, Bang V, Shreyder K, Brar S, Singh A, Dani SS, Knapp S, Poyan Mehr A, Brown RS, Zeidel ML, Bhargava R, Schlondorff J, Steinman TI, Mukamal KJ, & Parikh SM (2021) Niacinamide May Be Associated with Improved Outcomes in COVID-19-Related Acute Kidney Injury: An Observational Study. *Kidney360* 2(1):33-41.

20. Groff D, Sun A, Ssentongo AE, Ba DM, Parsons N, Poudel GR, Lekoubou A, Oh JS, Ericson JE, Ssentongo P, & Chinchilli VM (2021) Short-term and Long-term Rates of Postacute Sequelae of SARS-CoV-2 Infection: A Systematic Review. *JAMA Netw Open* 4(10):e2128568.
21. Jennings G, Monaghan A, Xue F, Mockler D, & Romero-Ortuno R (2021) A Systematic Review of Persistent Symptoms and Residual Abnormal Functioning following Acute COVID-19: Ongoing Symptomatic Phase vs. Post-COVID-19 Syndrome. *J Clin Med* 10(24).
22. Bahmer T, Borzikowsky C, Lieb W, Horn A, Krist L, Fricke J, Scheibenbogen C, Rabe KF, Maetzler W, Maetzler C, Laudien M, Frank D, Ballhausen S, Hermes A, Miljukov O, Haeusler KG, Mokhtari NEE, Witzenrath M, Vehreschild JJ, Krefting D, Pape D, Montellano FA, Kohls M, Morbach C, Stork S, Reese JP, Keil T, Heuschmann P, Krawczak M, Schreiber S, & group Ns (2022) Severity, predictors and clinical correlates of Post-COVID syndrome (PCS) in Germany: A prospective, multi-centre, population-based cohort study. *EClinicalMedicine* 51:101549.
23. Luo J, Brakel A, Krizsan A, Ludwig T, Motzing M, Volke D, Lakowa N, Grunewald T, Lehmann C, Wolf J, Borte S, Milkovska-Stamenova S, Gabert J, Fingas F, Scholz M, & Hoffmann R (2022) Sensitive and specific serological ELISA for the detection of SARS-CoV-2 infections. *Virol J* 19(1):50.
24. Luo J, Klett J, Gabert J, Lipp T, Karbach J, Jager E, Borte S, Hoffmann R, & Milkovska-Stamenova S (2022) A Quantitative ELISA to Detect Anti-SARS-CoV-2 Spike IgG Antibodies in Infected Patients and Vaccinated Individuals. *Microorganisms* 10(9).
25. Zou LQ, Linden L, Cuevas M, Metasch ML, Welge-Lussen A, Hahner A, & Hummel T (2020) Self-reported mini olfactory questionnaire (Self-MOQ): A simple and useful measurement for the screening of olfactory dysfunction. *Laryngoscope* 130(12):E786-E790.
26. Frasnelli J & Hummel T (2005) Olfactory dysfunction and daily life. *Eur Arch Otorhinolaryngol* 262(3):231-235.
27. Meek PM, Banzett R, Parsall MB, Gracely RH, Schwartzstein RM, & Lansing R (2012) Reliability and validity of the multidimensional dyspnea profile. *Chest* 141(6):1546-1553.
28. Banzett RB, O'Donnell CR, Guilfoyle TE, Parshall MB, Schwartzstein RM, Meek PM, Gracely RH, & Lansing RW (2015) Multidimensional Dyspnea Profile: an instrument for clinical and laboratory research. *Eur Respir J* 45(6):1681-1691.
29. Kroenke K, Strine TW, Spitzer RL, Williams JB, Berry JT, & Mokdad AH (2009) The PHQ-8 as a measure of current depression in the general population. *J Affect Disord* 114(1-3):163-173.
30. Spitzer RL, Kroenke K, Williams JB, & Lowe B (2006) A brief measure for assessing generalized anxiety disorder: the GAD-7. *Arch Intern Med* 166(10):1092-1097.
31. Schneider EE, Schonfelder S, Domke-Wolf M, & Wessa M (2020) Measuring stress in clinical and nonclinical subjects using a German adaptation of the Perceived Stress Scale. *Int J Clin Health Psychol* 20(2):173-181.
32. Smith BW, Dalen J, Wiggins K, Tooley E, Christopher P, & Bernard J (2008) The brief resilience scale: assessing the ability to bounce back. *Int J Behav Med* 15(3):194-200.
33. Buysse DJ, Reynolds CF, 3rd, Monk TH, Berman SR, & Kupfer DJ (1989) The Pittsburgh Sleep Quality Index: a new instrument for psychiatric practice and research. *Psychiatry Res* 28(2):193-213.
34. Smets EM, Garssen B, Bonke B, & De Haes JC (1995) The Multidimensional Fatigue Inventory (MFI) psychometric qualities of an instrument to assess fatigue. *J Psychosom Res* 39(3):315-325.
35. Doty RL, Frye RE, & Agrawal U (1989) Internal consistency reliability of the fractionated and whole University of Pennsylvania Smell Identification Test. *Percept Psychophys* 45(5):381-384.
36. Norton MC, Tschanz JA, Fan X, Plassman BL, Welsh-Bohmer KA, West N, Wyse BW, & Breitner JC (1999) Telephone adaptation of the Modified Mini-Mental State Exam (3MS). The Cache County Study. *Neuropsychiatry Neuropsychol Behav Neurol* 12(4):270-276.
37. Alexopoulos P, Perneczky R, Cramer B, Grimmer T, & Kurz A (2006) [Validation of a Short Telephone Test (T3MS) for the Diagnosis of Cognitive Impairment]. *Fortschr Neurol Psychiatr* 74(6):329-336.
38. Yellen SB, Cella DF, Webster K, Blendowski C, & Kaplan E (1997) Measuring fatigue and other anemia-related symptoms with the Functional Assessment of Cancer Therapy (FACT) measurement system. *J Pain Symptom Manage* 13(2):63-74.
39. Ware JE, Jr. & Sherbourne CD (1992) The MOS 36-item short-form health survey (SF-36). I. Conceptual framework and item selection. *Med Care* 30(6):473-483.

40. Miles J & Shevlin M (2001) *Applying regression and correlation : a guide for students and researchers* (Sage, London).

Appendix A: Summary of Analyses

Variable	Outcome	Population [a]	Statistical Method	Type
**Analyses of the PCS Score**				
Differences between treatment groups in the individual PCS Score at month 6	Metric	**ITT**, RFITT, RFPP, Responder	t-Test, Mann-Whitney-U	Cross sectional
PCS Score during the first 6 weeks of acute COVID-19 incl. comparison of treatment groups	Metric	**ITT**, RFITT, RFPP	t-Test, Mann-Whitney-U	Cross sectional
Time course of PCS Score during the trial incl. comparison of treatment groups	Metric	**ITT**, RFITT, RFPP, Responder	MMRM	Longitudinal
Individual course of PCS for patients with PCS Score >10 at month 6	Metric	**ITT**	Scatterplot	Longitudinal
Comparison of PCS Score between treatment groups for patients with PCS ≥5	Metric	**ITT**, RFITT, RFPP	t-Test, Mann-Whitney-U	Cross sectional
PCS Score in relation to time of recruitment (date baseline interview)	Metric	**ITT**	Scatterplot with LOESS interpolation	Cross sectional
PCS Score of patients with a mild disease course (symptom-free after 2 weeks) vs. others	Metric	**ITT**, RFITT, RFPP	t-Test, Mann-Whitney-U	Cross sectional
**Predictor analyses**				
Predictor analysis (2 Stages: a) Screening and extraction of relevant predictors for PCS, b) treatment comparison in the PCS risk subgroups)	Metric	**ITT** (a) only Placebo), RFITT, PCS Risk subgroups	t-Test, Mann-Whitney-U	Longitudinal using baseline parameters
Comparison of patients who reported impaired sense of smell or taste within the interviews with patients without impaired sense of smell or taste with respect to their results in QOD, MOQ, UPSIT and self-reported smelling/tasting	Metric	**ITT**	t-Test, Mann-Whitney-U	Cross sectional
**Comparison of symptoms to baseline**				
Differences in symptoms at month 6 compared to baseline	Categorical	**ITT**, RFITT, RFPP	Fisher-Exact Test or Chi-Square Test	Cross sectional
Differences in symptoms at month 6 compared to baseline (scale of complaints)	Metric	**ITT**, RFITT, RFPP	t-Test, Mann-Whitney-U	Cross sectional

**Analyses of antibody level**				
Level of antibodies against the N or S proteins of SARS-CoV-2	Metric	**ITT**, RFITT, RFPP	t-Test, Mann-Whitney-U	Cross sectional
Correlation of PCS Score and antibody levels	Metric	**ITT**	Pearson or Spearman correlation, Scatterplot, LOESS	Cross sectional
Treatment comparison of anti-N and anti-S antibody levels in patients with and without vaccination	Metric	**ITT**	t-Test, Mann-Whitney-U	Cross sectional
Time difference from positive SARS-CoV-2 test or first vaccination to time of sampling of antibodies between treatment groups	Metric	**ITT**	t-Test, Mann-Whitney-U	Cross sectional
Level of antibodies against the N or S proteins of SARS-CoV-2 between groups and vaccination level (none, one, multiple)	Metric	**ITT**, RFITT, RFPP	ANOVA	Cross sectional
Level of antibodies against the N or S proteins of SARS-CoV-2 between groups and vaccination level (none, one, multiple) applying threshold values	Categorical	**ITT**, RFITT, RFPP	Fisher-Exact Test or Chi-Square Test	Cross sectional
Correlation of time from positive SARS-CoV-2 test (or inclusion date) to date of laboratory testing of the level of antibodies against the N or S proteins of SARS-CoV-2	Metric	**ITT**, RFITT, RFPP	Correlation	Cross sectional
**Analyses of questionnaires and tests**				
Differences between treatment groups in the scale of the Self-MOQ	Metric	**ITT**, RFITT, RFPP	t-Test, Mann-Whitney-U	Cross sectional
Differences between treatment groups in the scale of the QOD	Metric	**ITT**, RFITT, RFPP	t-Test, Mann-Whitney-U	Cross sectional
Differences between treatment groups in the scale and subscales of the MDP	Metric	**ITT**, RFITT, RFPP	t-Test, Mann-Whitney-U	Cross sectional
Differences between treatment groups in the scale of the PHQ-8	Metric	**ITT**, RFITT, RFPP	t-Test, Mann-Whitney-U	Cross sectional
Differences between treatment groups in the scale of the GAD-7	Metric	**ITT**, RFITT, RFPP	t-Test, Mann-Whitney-U	Cross sectional
Differences between treatment groups in the scale of the PSS	Metric	**ITT**, RFITT, RFPP	t-Test, Mann-Whitney-U	Cross sectional

Differences between treatment groups in the scale of the BRS	Metric	**ITT**, RFITT, RFPP	t-Test, Mann-Whitney-U	Cross sectional
Differences between treatment groups in the scale and subscales of the PSQI	Metric	**ITT**, RFITT, RFPP	t-Test, Mann-Whitney-U	Cross sectional
Differences between treatment groups in the scale and subscales of the MFI	Metric	**ITT**, RFITT, RFPP	t-Test, Mann-Whitney-U	Cross sectional
Differences between treatment groups in the scale and subscales of the FACIT-F	Metric	**ITT**, RFITT, RFPP	t-Test, Mann-Whitney-U	Cross sectional
Differences between treatment groups in the scale and subscales of the SF-36	Metric	**ITT**, RFITT, RFPP	t-Test, Mann-Whitney-U	Cross sectional
Differences between treatment groups in the scale of the UPSIT	Metric	**ITT**, RFITT, RFPP	t-Test, Mann-Whitney-U	Cross sectional
Differences between treatment groups in the scale of the T3MS cognitive test	Metric	**ITT**, RFITT, RFPP	t-Test, Mann-Whitney-U	Cross sectional
Differences between treatment groups in the combination of items for smelling and tasting of self-reported smelling and tasting abilities	Metric	**ITT**, RFITT, RFPP	t-Test, Mann-Whitney-U	Cross sectional

Annotation: a) Primary analysis population: ITT
