# Supplementary material for: Nicotinamide modulates gut microbial metabolic potential and accelerates recovery in mild-to-moderate COVID-19
Source: Nat Metab. 2025 May 12;7(6):1136–49. doi: 10.1038/s42255-025-01290-1 (PMC12198012; doi:10.1038/s42255-025-01290-1)
Supplement: Supplementary file 2 — Reporting Summary [file 42255_2025_1290_MOESM2_ESM.pdf]

nature portfolio

Corresponding author(s): Stefan Schreiber

Last updated by author(s): Mar 17, 2025

Reporting Summary

Nature Portfolio wishes to improve the reproducibility of the work that we publish. This form provides structure for consistency and transparency in reporting. For further information on Nature Portfolio policies, see our Editorial Policies and the Editorial Policy Checklist.

Statistics

For all statistical analyses, confirm that the following items are present in the figure legend, table legend, main text, or Methods section.

n/a	Confirmed	
☐	☒	The exact sample size (*n*) for each experimental group/condition, given as a discrete number and unit of measurement
☐	☒	A statement on whether measurements were taken from distinct samples or whether the same sample was measured repeatedly
☐	☒	The statistical test(s) used AND whether they are one- or two-sided<br>*Only common tests should be described solely by name; describe more complex techniques in the Methods section.*
☐	☒	A description of all covariates tested
☐	☒	A description of any assumptions or corrections, such as tests of normality and adjustment for multiple comparisons
☐	☒	A full description of the statistical parameters including central tendency (e.g. means) or other basic estimates (e.g. regression coefficient) AND variation (e.g. standard deviation) or associated estimates of uncertainty (e.g. confidence intervals)
☐	☒	For null hypothesis testing, the test statistic (e.g. *F*, *t*, *r*) with confidence intervals, effect sizes, degrees of freedom and *P* value noted<br>*Give P values as exact values whenever suitable.*
☒	☐	For Bayesian analysis, information on the choice of priors and Markov chain Monte Carlo settings
☐	☒	For hierarchical and complex designs, identification of the appropriate level for tests and full reporting of outcomes
☐	☒	Estimates of effect sizes (e.g. Cohen's *d*, Pearson's *r*), indicating how they were calculated

Our web collection on statistics for biologists contains articles on many of the points above.

Software and code

Policy information about availability of computer code

Data collection	eCRF (IT's Ambio AG), MiSeq v3.0 software (Illumina)
Data analysis	R (v. 4.1.2, v. 4.2.1); QIIME2 environment6 (release 2021.4) with DADA2 plugin (v.2021.2); TOFU-MAaPO v1.2.2 (https://github.com/ikmb/TOFU-MAaPO; last accessed on 11-Mar-2025; relies on the bioBakery 3 environment9 [MetaPhlAn 3.0 and HUMAnN 3.0]); MetaCyc v26.1 (https://metacyc.org/; last accessed on 11-Mar-2025); phyloseq (v.1.40.0); vegan (v.2.6-2); MAasLin2 (v.1.10.0); lme4 package in R (v1.1-34); variancePartition15 v.1.26.0.

For manuscripts utilizing custom algorithms or software that are central to the research but not yet described in published literature, software must be made available to editors and reviewers. We strongly encourage code deposition in a community repository (e.g. GitHub). See the Nature Portfolio guidelines for submitting code & software for further information.

Data

Policy information about availability of data

All manuscripts must include a data availability statement. This statement should provide the following information, where applicable:

- Accession codes, unique identifiers, or web links for publicly available datasets
- A description of any restrictions on data availability
- For clinical datasets or third party data, please ensure that the statement adheres to our policy

The COVit-1 pilot trial and the COVit-2 main trial were preregistered with a data sharing statement at the WHO primary registry German Clinical Trials Register

(DRKS00021214). COVit-2 was additionally registered with ClinicalTrials.gov (NCT04751604). The trial protocol and statistical analysis plans are available in the Supplement with publication. The microbiome sequencing reads are deposited and available at ENA under the accession code PRJEB61276 (https://www.ebi.ac.uk/ena/browser/view/PRJEB61276; last accessed on 11-Mar-2025). The taxonomic classification of 16S rRNA data performed using the SILVA database (version 138) is publicly available at Zenodo: https://zenodo.org/records/6395539 (last accessed on 11-Mar-2025). Clinical data are not available for download due to privacy law according to the European Union's General Data Protection Regulation (EU GDPR) and due to ethical restrictions. Specific requests by academic researchers for access to clinical data can be addressed to the corresponding author. These data include individual de-identified participant data and data sorted by sex and diversity. Based on such a request including a detailed analysis plan, access may be provided subject to a decision of the Ethics Committee of the Medical Faculty of Kiel University (Kiel, Germany) to ensure compliance with privacy laws, data protection and requirements for consent and anonymization. Requests will be considered from the date of publication of this article. It is expected that data can be obtained within 90 days after the eventual ethics vote. Data will be available for 10 years.

Research involving human participants, their data, or biological material

Policy information about studies with human participants or human data. See also policy information about sex, gender (identity/presentation), and sexual orientation and race, ethnicity and racism.

Reporting on sex and gender

Demographics including sex assigned at birth (male/female) or the gender option 'diverse' (not selected by any patient) were collected at screening as reported by the patient. Due to the lack of evidence for a sex-specific effect of nicotinamide in COVID-19 or similar infections, neither sex nor gender were specifically considered in the design of the trial, but analysed in an exploratory fashion. Exploratory subgroup analyses of the primary and three key secondary endpoints showed no sex-dependent differences.

Reporting on race, ethnicity, or other socially relevant groupings

Race or ethnic group (white/other/not reported) were collected at screening as reported by the patient. No analyses based on this grouping were performed.

Population characteristics

Symptomatic outpatients with acute COVID-19 (inclusion within 7 days after testing PCR-positive for SARS-CoV-2 infection) were the intention-to-treat population (ITT). Among these, the primary efficacy population were patients with at least one risk factor for severe COVID-19 (RFITT). The randomized allocation of patients ensured a comparable influence of potential confounders in both groups. A comparison of all baseline characteristics between the two treatment groups revealed no relevant imbalances. Consequently, the models were analyzed without additional adjustment for confounders. Examined baseline characteristics included age, gender, pre-existing conditions, and medication use.

Recruitment

Patients were recruited through 24 independent diagnostic laboratory service providers with a total of 71 sites all over Germany. All patients who tested positive in the recruitment period at the laboratory sites were contacted together with the transmission of their laboratory results. Patients volunteered their identity to the recruitment center and after verification of key data including the referring laboratories and key inclusion criteria such as the test date, patients received the personalized patient information and a participation code, and could give informed consent on a secure trial website. Subsequently, they were contacted via telephone to check all inclusion and exclusion criteria and explain the informed consent. While a certain self-selection bias applied due to the ability of the subjects to swallow tablets, provide informed consent via the internet and talk to interviewers on the phone, the prospective, double-blind, randomized, placebo-controlled structure of the trial makes it highly unlikely that any bias should have significantly affected the results of the trial.

Ethics oversight

Ethics Committee of the Medical Faculty of Kiel University, Kiel, Germany (file reference A107/20).

Note that full information on the approval of the study protocol must also be provided in the manuscript.

Field-specific reporting

Please select the one below that is the best fit for your research. If you are not sure, read the appropriate sections before making your selection.

[x] Life sciences [ ] Behavioural & social sciences [ ] Ecological, evolutionary & environmental sciences

For a reference copy of the document with all sections, see nature.com/documents/nr-reporting-summary-flat.pdf

Life sciences study design

All studies must disclose on these points even when the disclosure is negative.

Sample size

Data available from the literature available at the beginning of the COVit-2 trial and results of the COVit-1 pilot trial (see section 1 of the Supplement) suggested that 40% of symptomatic patients with COVID-19 become symptom-free during the spontaneous course of the disease within two weeks. An increase of the event rate of becoming symptom-free to 50% (relative risk 1.25) through the use of nicotinamide at a 5% level of significance and 80% power resulted in a required sample size of at least 385 patients per group (for details, see section 6 of the Supplement). With an assumed drop-out rate of 10%, 420 patients per group (assigned in a 1:1 ratio) were to be included. Based on the results of the futility analysis after 400 patients, the Data Management Board (DMB) of the COVit-2 trial recommended (1) to keep the previously planned sample size of approximately 840 patients and (2) to base the efficacy analysis on patients with at least one risk factor for developing severe COVID-19 (RFITT, see section 3.4 of the Supplement). To ensure that the subpopulation recruited after the futility analysis also included an adequate proportion of such patients, the frequencies of risk factors were monitored in a blinded manner and 900 patients were recruited as a safeguard against possibly increased dropout rates. The Principal Investigator and the blinded trial team members remained blinded until the end of the trial and received only the recommendation of the DMB.

Data exclusions	All data were analysed according to the SAPs without exclusions.
Replication	The results of the large, powered COVit-2 trial reproduced the exploratory findings of the COVit-1 pilot trial. Experimental results for microbiome analyses were generated using established SOPs (see section 3.5 of the Supplement). There were no findings suggesting a lack of reproducibility.
Randomization	Eligible patients were randomly assigned in a 1:1 ratio, by means of a randomization code in the trial database.
Blinding	Blinded (recruiters, interviewers, study physicians, statisticians, technicians and scientists for microbiome analysis) and unblinded personnel (study material distribution, safety) were strictly separated.

Reporting for specific materials, systems and methods

We require information from authors about some types of materials, experimental systems and methods used in many studies. Here, indicate whether each material, system or method listed is relevant to your study. If you are not sure if a list item applies to your research, read the appropriate section before selecting a response.

Materials & experimental systems

n/a	Involved in the study
☒	☐ Antibodies
☒	☐ Eukaryotic cell lines
☒	☐ Palaeontology and archaeology
☒	☐ Animals and other organisms
☐	☒ Clinical data
☒	☐ Dual use research of concern
☒	☐ Plants

Methods

n/a	Involved in the study
☒	☐ ChIP-seq
☒	☐ Flow cytometry
☒	☐ MRI-based neuroimaging

Clinical data

Policy information about clinical studies

All manuscripts should comply with the ICMJE guidelines for publication of clinical research and a completed CONSORT checklist must be included with all submissions.

Clinical trial registration	The COVit-1 pilot trial and the COVit-2 main trial were preregistered with a data sharing statement at the WHO primary registry German Clinical Trials Register (DRKS00021214). COVit-2 was additionally registered with ClinicalTrials.gov (NCT04751604).
Study protocol	See section 5 of the Supplement.
Data collection	The trials were performed remotely due to the contract restrictions which were in place during this time period. For details on trial procedures and design, see section 3.1 of the Supplement. Patients in the COVit-1 pilot trial were recruited between 06 April 2020 and 28 January 2021, and patients in the main COVit-2 trial were recruited between February 1, 2021, and January 17, 2022. The molecular data were generated from stool samples received from trial participants at the Institute of Clinical Molecular Biology (Kiel University and University Hospital Schleswig-Holstein, Kiel, Germany).
Outcomes	The original primary clinical outcome of COVit-1 was the frequency of hospital admission in order to receive at least 24 h of continuous oxygen therapy, and secondary endpoints included frequencies of machine ventilation, intensive care, death as well as time to resolution of symptoms (for details, see section 1 of the Supplement). Due to the results of the pilot experiment, COVit-2 focused on the COVID-19-related patient-reported symptom burden in the acute primary analysis population RFITT (intention-to-treat patients with at least one risk factor for severe COVID-19; section 3.4 of the Supplement). The primary endpoint was restoration of physical performance at week 2. Key secondary endpoints were an improvement of the ability to perform normal activities, resolution of cough, and resolution of fatigue at week 2. All endpoints were tested in patients suffering from the respective symptoms at baseline. Prespecified subgroup analyses were performed for key risk factors. At the 6-month follow-up, the main outcome was post-COVID syndrome (PCS) determined by a previously established PCS score, which was derived and validated in a large and prospective German cohort. For the complete list of outcomes and for details on trial populations, see sections 3.2 and 3.4 of the Supplement, respectively.

Plants

Seed stocks	*Report on the source of all seed stocks or other plant material used. If applicable, state the seed stock centre and catalogue number. If plant specimens were collected from the field, describe the collection location, date and sampling procedures.*
Novel plant genotypes	*Describe the methods by which all novel plant genotypes were produced. This includes those generated by transgenic approaches, gene editing, chemical/radiation-based mutagenesis and hybridization. For transgenic lines, describe the transformation method, the number of independent lines analyzed and the generation upon which experiments were performed. For gene-edited lines, describe the editor used, the endogenous sequence targeted for editing, the targeting guide RNA sequence (if applicable) and how the editor was applied.*
Authentication	*Describe any authentication procedures for each seed stock used or novel genotype generated. Describe any experiments used to assess the effect of a mutation and, where applicable, how potential secondary effects (e.g. second site T-DNA insertions, mosiacism, off-target gene editing) were examined.*
